# A modular DNA origami nanocompartment for engineering a cell-free, protein unfolding and degradation pathway

**DOI:** 10.1038/s41565-024-01738-7

**Published:** 2024-07-29

**Authors:** J. Huang, A. Jaekel, J. van den Boom, D. Podlesainski, M. Elnaggar, A. Heuer-Jungemann, M. Kaiser, H. Meyer, B. Saccà

**Affiliations:** 1 Bionanotechnology, CENIDE and ZMB, University of Duisburg-Essen, Essen, Germany; 2 Molecular Biology, ZMB, University of Duisburg-Essen, Essen, Germany; 3 Chemical Biology, ZMB, University of Duisburg-Essen, Essen, Germany; 4 Max Planck Institute of Biochemistry, Martinsried, Germany

**Keywords:** Nanostructures, Organizing materials with DNA, DNA nanomachines

## Abstract

Within the cell, chemical reactions are often confined and organized through a modular architecture. This facilitates the targeted localization of molecular species and their efficient translocation to subsequent sites. Here we present a cell-free nanoscale model that exploits compartmentalization strategies to carry out regulated protein unfolding and degradation. Our synthetic model comprises two connected DNA origami nanocompartments (each measuring 25 nm × 41 nm × 53 nm): one containing the protein unfolding machine, p97, and the other housing the protease chymotrypsin. We achieve the unidirectional immobilization of p97 within the first compartment, establishing a gateway mechanism that controls substrate recruitment, translocation and processing within the second compartment. Our data show that, whereas spatial confinement increases the rate of the individual reactions by up to tenfold, the physical connection of the compartmentalized enzymes into a chimera efficiently couples the two reactions and reduces off-target proteolysis by almost sixfold. Hence, our modular approach may serve as a blueprint for engineering artificial nanofactories with reshaped catalytic performance and functionalities beyond those observed in natural systems.

## Abstract (web summary)

This study presents DNA-origami biocatalytic modular nanocompartments for programmed regulation of protein unfolding and degradation. These artificial nanofactories augment reaction kinetics, improve enzyme performance and reduce off-target effects.

## Main

The physical separation of chemical reactions in specialized compartments is a hallmark of cellular metabolism^1,2^. Modular enzymes, such as the proteasome^3–5^, execute complex tasks by processing the substrate in specialized catalytic domains organized in a sequential order. Inspired by these natural machines, man-made biological compartments have been realized to reconstitute and manipulate metabolic pathways in both cellular and cell-free settings^6–10^. While most systems rely on the periodic self-assembly of protein-^11^ and lipid-based^12^ building blocks, approaches based on nucleic acids, particularly the DNA origami method^13^, enable the fabrication of three-dimensional (3D) architectures of programmable shape and known spatial coordinates for each nucleobase. This allows to functionalize origami surfaces with subnanometre accuracy^14^. Furthermore, modular assembly procedures have been developed to guide the ordered association of multiple structures into micrometre-large assemblies^15–18^. Altogether, these features have been used, for example, to investigate the effect of spatial confinement and inter-molecular distance on the activity of individual enzymes^19–21^ or enzyme pairs^22–26^. In the quest to mimic the complexity of natural modular enzymes, an important challenge is to control the sequential order of multiple reactions.

In this Article, we harness DNA programmability to engineer a modular and compartmentalized construct with multi-catalytic function. Our artificial chimera couples a protein segregation and unfolding process to a downstream proteolytic reaction, resulting in a semisynthetic prototype of the 26S proteasome: a self-compartmentalized and unfoldase-assisted protein degradation machine^3^. Through precise control of stoichiometry, spatial arrangement and unidirectional orientation of the unfolding machine, our design provides a substrate-specific gateway mechanism for regulating substrate entry, unfolding and processing into the downstream proteolytic module. We show that, whereas spatial confinement enhances the rate of each catalytic step, physical connection of the modules further enhances the global performance of the cascade and minimizes off-target interactions. Finally, by modifying the activity of the downstream module, we reprogram the chimera for a distinct task, showcasing the potential use of our approach for engineering biocatalytic pathways on selected substrates.

## A nanoscale model of a modular enzyme

**Fig. 1:**
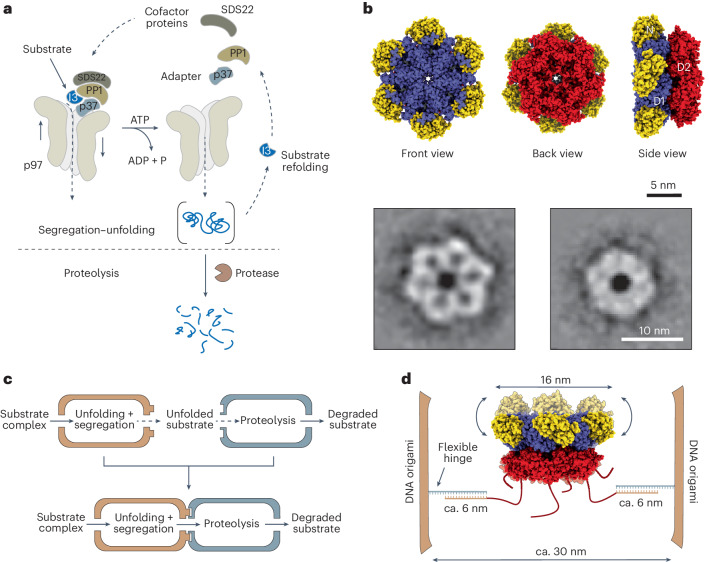
A modular nanoscale model of compartmentalization. **a**, The biocatalytic reactions coupled in this work: substrate segregation–unfolding and substrate proteolysis. In the first reaction, p97 binds the substrate protein I3 (25 kDa), trapped in a complex with PP1 (37 kDa) and SDS22 (50 kDa), via the adapter protein p37 (ca. 40 kDa). Binding occurs at the N-terminal domains of p97. Addition of ATP drives the unidirectional threading of I3 through the central p97 channel and its ejection at the C-terminal tails, resulting in the unfolding of I3 and segregation from PP1 and SDS22. In the second reaction, the proteolytic degradation of the substrate is catalysed by aCt (25 kDa). All proteins involved in the modular chimera and their complexes are reported in Supplementary Table 1. **b**, Three-dimensional surface model of the p97 hexamer in the resting state (PDB 5FTK). N domains are indicated in yellow, D1 domains in violet and D2/C terminal domains in red. The molecule is viewed from the front (left), back (middle) and side (right). Front and back views correspond well to the p97 structures observed by negative stain TEM (*n* = 13,819 particles, 2.39 Å per pixel, box size 120 pixels, particles were sorted and averaged in 100 classes). Side views were not found in class averages. When viewed from the side, the global shape of p97 can be inscribed into a truncated cone with the larger base corresponding to the D1 ring plus the peripheral N domains and the smaller base corresponding to the D2 ring. **c**, Schematic representation of the programmable modular enzyme: each biocatalytic reaction is confined within a DNA compartment and the compartments are later joined in a programmable order to perform a defined reaction cascade. **d**, Schematic representation of the design of the DNA origami compartment hosting one p97 protein (side view). The design must enable protein immobilization, while permitting active protein movement and sufficient room for binding partners. Source data

The first reaction of our modular chimera is catalysed by the valosin-containing protein (VCP)/p97: a protein unfolding machine with a major role in cell homeostasis, proliferation and signalling^27,28^. The second reaction is catalysed by α-chymotrypsin (aCt): a well-studied and robust serine protease of the S1 family (Fig. 1a)^29^. As a type II AAA ATPase^30^, p97 is composed of two hexameric stacked rings (D1 and D2) forming a central narrow channel, with N-terminal domains attached to the D1 ring and flexible C-terminal tails attached to the D2 ring (Fig. 1b). In the cell, p97 recruits ubiquitylated substrate proteins to prepare them for degradation in the proteasome^31,32^. Alternatively, p97 can target substrate proteins in a ubiquitin-independent manner for recycling^33^. Both pathways require specific adapter proteins but rely on the same unfolding mechanism. This starts with recruitment of the substrate to the N-terminal domains of p97. Afterwards, ATP hydrolysis drives the insertion of the substrate into the D1 pore, its threading through the channel and final ejection from the D2 pore. The mechanical forces applied to the substrate lead to substrate unfolding and, in some cases, to its concomitant separation from the binding partners. Here, we in vitro reconstituted a ubiquitin-independent segregation and unfolding pathway and used it as the upstream reaction of our model. Specifically, the inhibitor-3 (I3) substrate, in complex with PP1 and SDS22 cofactors (forming an SP–I3 complex), is directly recruited to p97 by the adapter protein, p37. Addition of ATP results in the unfolding of I3 and concomitant segregation of I3 from its binding partners (Fig. 1a and Supplementary Table 1).

To efficiently couple substrate unfolding to its proteolytic digestion, the p97 machine must be close enough to aCt but physically separated from it to prevent off-target proteolysis. This may be realized by first immobilizing each enzyme in a suitable compartment and, subsequently, linking the two differently loaded compartments into a chimera (Fig. 1c). The compartment should allow for the encapsulation of a single p97 protein while permitting its mechanical motion and sufficient room for binding partners, both essential for correct p97 functioning (Fig. 1d). The compartment should also enable controlled loading of chymotrypsin and the implementation of modular assembly strategies. Finally, since the substrate threading reaction is strictly unidirectional, namely from the N- to the C-terminal domains of p97, two critical conditions need to be satisfied: (1) the p97 pore must be aligned with the central axis of the DNA compartment to guarantee unhindered translocation of the unfolded substrate to the proteolytic module; (2) the relative orientation of p97 inside the DNA compartment must be known and uniquely defined to ensure that all constructs feature the same N-to-C direction of substrate translocation.

## Design and assembly of the modular compartment

**Fig. 2:**
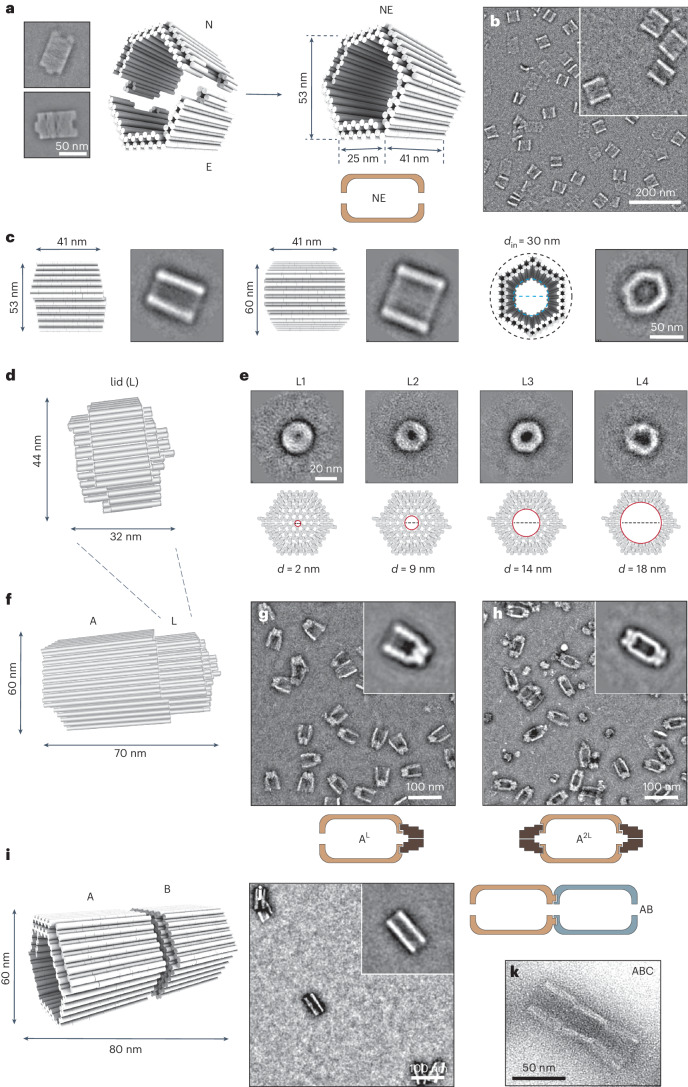
Design and characterization of the DNA origami compartment. **a**, The NE compartment was obtained by hybridization of two halves (N and E) that are mutually complementary in shape. The front and back edges of NE correspond to the left and right side of the design chart (Supplementary Figs. 1 and 2). **b**, Successful formation of NE was confirmed by negative-stain TEM (yield 98%). **c**, Molecular models and sizes of NE in different views well match with the structures observed by TEM. Class averages are shown (*n* = 3,065; 3.83 Å per pixel, box size 384 pixels). **d**, NE was modified at the edges to enable connection with a lid (L). **e**, The lid displays a central pore with variable diameters (2 nm to 18 nm: L1 to L4, respectively). Corresponding class averages are shown (*n* = 898, 959, 1,775 and 591; yields >95% for each structure). **f**–**h**, The lid can be linked either at one (**f** and **g**; A^L^) or both sides of the compartment (**h**; A^2L^). In **f**, the molecular model of the structure visualized in **g** is shown. Class averages are shown in the insets (*n* = 1,477 for A^L^; *n* = 358 for A^2L^; yields >96%). **i**–**k**, Two-compartment constructs (**i** and **j**; AB, class average from *n* = 354 is shown in the inset; yield 70%) and three-compartment constructs (**k**; ABC) were also fabricated. In **i**, the molecular model of the structure visualized in **j** is shown. Scale bars are reported for all DNA origami constructs in representative TEM images. If not stated differently, box size was 200 pixels and particles were picked at 7.42 Å per pixel resolution, sorted and averaged in 32 classes. The assembly yields of all constructs are reported in Supplementary Table 2. Source data

To meet the criteria described above, we designed the DNA origami compartment illustrated in Fig. 2. The structure (Nemesis, NE) is a hollow prism of hexagonal shape (25 nm × 41 nm × 53 nm), composed of two identical halves (Narcissus (N) and Echo (E)), linked together by nucleobase hybridization at shape-complementary edges (Fig. 2a and Supplementary Figs. 1–3). The compartment was designed to match the size and shape of p97 while ensuring enough room for its binding to protein partners and unhindered mechanical movement (Fig. 1d).

Individual N, E and NE origami structures were assembled, purified and analysed by atomic force microscopy (AFM), transmission electron microscopy (TEM), dynamic light scattering and agarose gel electrophoresis (AGE). All structures formed with at least 98% yield (Fig. 2b,c, Supplementary Figs. 4–10 and Supplementary Table 2). The front and back edges of NE were modified with extrusion and intrusion features to implement modularity. This allowed attaching one or two DNA origami lids at the extremities of NE (Fig. 2d–h and Supplementary Figs. 11–13) or linking two or three NE structures along their longitudinal axes to form linear multi-compartment constructs (Fig. 2i–k and Supplementary Figs. 14–17). For simplicity, modified NE compartments will be hereafter referred to as ‘A’ or ‘B’ followed by a superscript specifying the presence of one or two lids (A^L^ and A^2L^). Functionalization of the inner cavity of the compartment with single-stranded protruding arms (PA) will be instead indicated by a subscript (A_PA_). The chimera will be named as AB, with A and B being, respectively, the upstream and downstream module.

## Spatial confinement of the unfolding machine

**Fig. 3:**
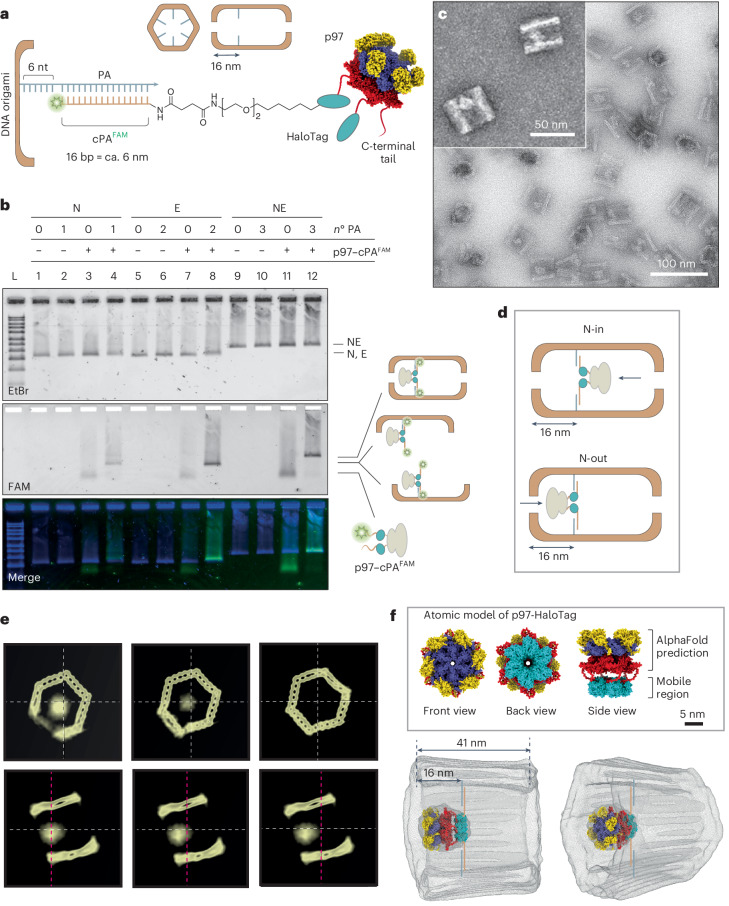
Encapsulation of the p97 machine. **a**, The C-terminal tails of p97 were fused to HaloTag domains and linked to cPA^FAM^ strands. The hexameric p97–cPA^FAM^ conjugate (780 kDa) was encapsulated into the NE compartment via hybridization to six PA strands extending from the inner surface of the origami, leaving 6-nt-long toeholds to facilitate protein movement and permit subsequent protein displacement. **b**, Representative AGE characterization of p97 binding to half (lanes 3–4 and 7–8) and full compartments (lanes 11 and 12), with 0, 1, 2 or 3 PA (the number of PA is indicated by *n*°): co-migration of DNA origami constructs and FAM-labelled p97 occurred only in the presence of PAs (lanes 4, 8 and 12). Gel running conditions: 0.75 % agarose in 1× TBEMg, 80 V for 2.5 h at 4 °C. The gel was scanned with a Typhoon FLA9000 (GE Healthcare Life Sciences) to monitor FAM emission and finally stained with EtBr. **c**, Representative TEM image of the encapsulated p97 (10 MDa) showing the expected positioning of the protein at one side of the compartment, with the long axis of the DNA compartment and the central pore of p97 being aligned (*n* = 758; yield 75%). A detailed view is shown in the inset. **d**, Two possible co-axial orientations of p97 after encapsulation inside the DNA origami chamber (N-in and N-out); these configurations are not superimposable and are therefore clearly distinguishable. **e**, Orthogonal slices of the refined and averaged 3D electron density map (from *n* = 14,400 particles) of A(p97) at different positions across the structure. Front (top) and side (bottom) views highlight the asymmetric shape of the protein. **f**, Top: atomic model of p97-HaloTag predicted by AlphaFold2. The relative position of the HaloTag-cPA domains (cyan) cannot be predicted accurately due to the flexibility of the C-terminal tails of p97 (red); nevertheless, they are aligned with the PAs located 16 nm away from one edge of the compartment. Bottom: the electron density map of A(p97) shows an asymmetric protein signal localized at one side of the chamber. The atomic model of p97-HaloTag fits into the protein density map only in the N-out configuration. Source data

Next, self-labelling HaloTag subunits were genetically fused to the C-terminus of p97 and covalently linked to chlorohexane-modified DNA handles, further equipped with a fluorescein label (FAM) for tracking purposes (cPA^FAM^ in Fig. 3a; Supplementary Figs. 18 and 19). The resulting p97–cPA^FAM^ conjugate was purified and bound to the inner surface of the DNA compartment through hybridization to six complementary PAs: one for each face of the hexagonal prism and all positioned ca. 16 nm away from the front opening (Fig. 3a, top scheme). The PA/cPA^FAM^ sequences formed 16-bp-long duplexes flanked by 6-nt-long toeholds (Fig. 3a, bottom scheme). This design was conceived to favour the accommodation of a single p97 guest into the cavity of the DNA host and align their central axes. The toeholds were introduced to facilitate protein motion and allow subsequent detachment of the protein via strand displacement mechanisms.

To test our design, halves and full compartments were incubated with an excess of p97–cPA^FAM^ conjugate and the products of the reaction were analysed by AGE. The results showed that co-localization of the p97 and DNA origami bands occurred only in presence of PA handles (Fig. 3b and Supplementary Fig. 20). This indicates that binding of the protein to the DNA compartment is specifically driven by the intended PA/cPA^FAM^ hybridization. The purified DNA origami–protein complex, in the following indicated as A(p97), was further analysed by TEM (Fig. 3c and Supplementary Figs. 21–23). Single particle counting revealed successful protein encapsulation in about 75% of the structures (Supplementary Table 2). In the TEM images, p97 appeared as an oval shape at one end of the compartment with the long axis of p97 being parallel to the chamber’s aperture. This suggests that the protein is trapped in the correct location of the chamber and with its pore being co-axially aligned with the DNA cavity (Fig. 3c, inset).

Once aligned, the p97 protein can theoretically assume two distinct orientations with respect to the DNA compartment (Fig. 3d): in one orientation, the N-terminal domains of the protein point towards the inner cavity of the compartment (N-in); in the other orientation, the same domains point outside (N-out). Both orientations enable the recruitment of the SP–I3 complex and the unfolding of I3 through the p97 pore. However, the net flow of substrate unfolding is expected to be opposite in the two orientations.

Cryo-electron microscopy (cryo-EM) data, though at low resolution, revealed the prevalent formation of the N-out species (Fig. 3e,f and Supplementary Fig. 24). Unambiguous assignment was possible because of two asymmetric structural features in our system: the shape of the p97 protein (Fig. 1b) and the positioning of the protruding arms on one side of the DNA compartment (Fig. 3a). These features break the symmetry of the A(p97) construct and allow to distinguish the N-in from the N-out orientation, since these configurations are not superimposable (Fig. 3d). The averaged 3D cryo-EM density map revealed an asymmetric protein signal co-axially aligned with the central axis of the DNA compartment and mostly located on one side, with the largest portion of the signal (due to the D1 ring and the peripheral N domains) being near the chamber aperture (Fig. 3f, bottom, and Supplementary Video 1). Orthogonal slices of the density map further highlighted the asymmetric shape of the protein, tapering down from one extremity of the chamber towards its interior (Fig. 3e). We also generated an atomic model of p97-HaloTag in its apo state (Fig. 3f, top). The model fitted well into the 3D protein density map, albeit only in the N-out configuration (Fig. 3f, bottom, and Supplementary Fig. 24).

Altogether, our structural data indicate that p97 is co-axially aligned with the central axis of the DNA chamber and that, while exhibiting limited mobility, it predominantly localizes at one end of the compartment with the larger N-terminal subunits pointing outside. Although the reason for this biased alignment is not yet clear, its occurrence elegantly solves the main structural challenge of our system: the achievement of a well-defined and co-axial orientation of the unfolding machine within the compartment. This arrangement provides a substrate-specific gateway mechanism that controls accessibility to the compartment and imposes a unidirectional flow of substrate translocation.

## Substrate unfolding in the upstream module

**Fig. 4:**
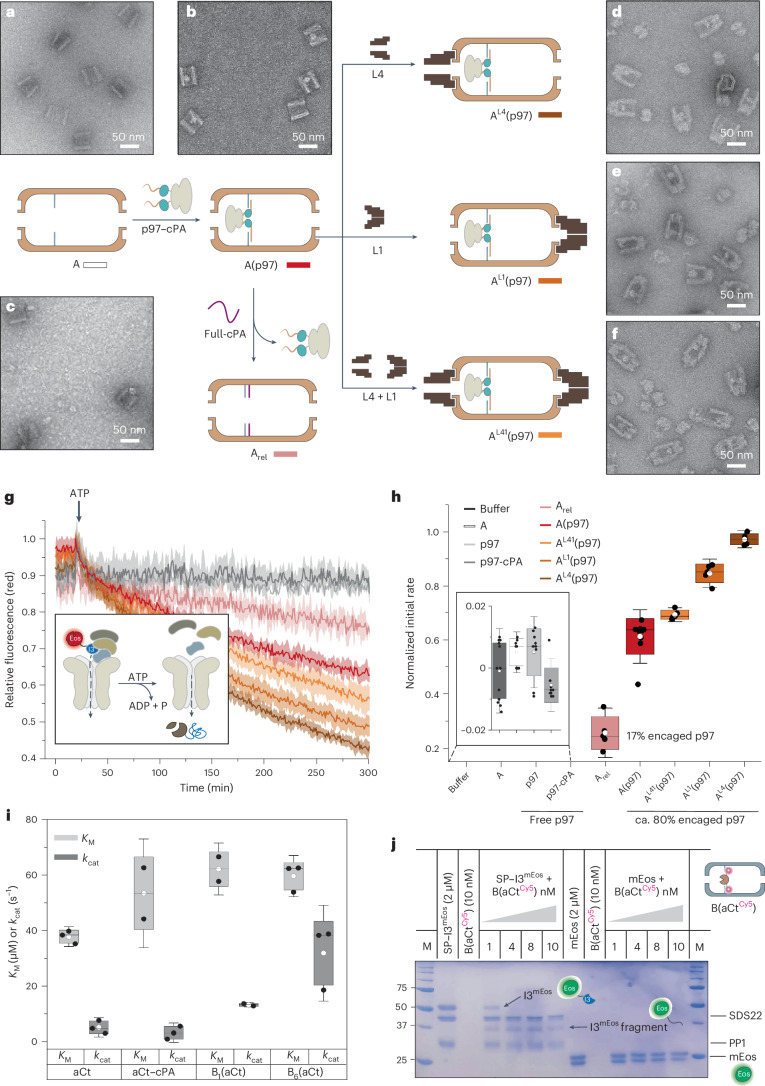
Effect of DNA compartmentalization on enzyme activity. **a**–**f**, Schematic representation of the experimental procedure applied to construct A(p97) variants. The p97-cPA conjugate was immobilized into the A compartment decorated with inner-pointing PA handles (**a**), leading to A(p97) (**b**). Addition of full-cPA strands to A(p97) displaced the protein from the compartment, yielding A_rel_ (**c**). Subsequent addition of lids at one or both sides of A(p97) allowed to assess the effect of spatial confinement upstream of p97 (**d**; A^L4^), restricted substrate escape (**e**; A^L1^) or both (**f**; A^L41^). Representative TEM images for each construct are reported. **g**, The unfolding activity of p97 was measured by following the decrease of the fluorescence signal at 540/580 nm (ex/em) of a photoactivated I3^mEos^ substrate, upon addition of ATP. Average time-course profiles from *n* = 3 technical replicates are reported (A_rel_, pink; A(p97), red; A^L41^, orange; A^L1^, light brown, A^L4^, dark brown; p97, light grey; p97–cPA, dark grey). Curves from buffer (black) and A (white) were excluded from the plot for clarity. **h**, Normalized initial rates for the constructs obtained as in **a**–**f** were calculated by the slopes of the curves obtained as in **g** in the interval between 50 min and 200 min. The data are presented as mean ± s.d. from at least *n* = 3 technical replicates and up to *n* = 4 biological replicates for each sample. **i**, Michaelis–Menten parameters (*K*_M_ and *k*_cat_; light and dark grey) for the proteolytic degradation of Suc-AAPF-pNA catalysed by aCt, aCt–cPA conjugate, B_1_(aCt) or B_6_(aCt). The data are presented as mean ± s.d. from *n* = 2 or 3 biological replicates (Supplementary Table 3). The whiskers (in **h** and **i**) are 1.5 s.d.; mean (white dots) and median (black lines) are indicated for each data set. No outliers have been excluded. **j**, SDS PAGE of the proteolytic digestion of green mEos, either alone (right lanes) or fused to I3 (SP–I3^mEos^; left lanes) and at increasing concentrations of B(aCt^Cy5^) (1–10 nM). mEos is stable to proteolysis. Lack of aCt bands in B(aCt^Cy5^) lanes indicates that immobilized aCt is protected from autoproteolysis. The gel was stained with Coomassie and scanned with a Typhoon FLA9000 (GE Healthcare Life Sciences). Source data

To investigate the effect of compartmentalization on p97 activity, we prepared different types of DNA compartments (Fig. 4a–f). Substrate unfolding by p97 was monitored by fusing I3 to mEos (forming I3^mEos^)^33^. mEos is a photoactivable protein that shifts its fluorescence emission from green to red upon the ultraviolet (UV)-induced breakage of a peptide backbone near the chromophore^34^. This prevents mEos refolding, enabling to associate a loss in red fluorescence signal to substrate unfolding (schematics in Fig. 4g). Once I3^mEos^ in complex with SDS22 and PP1 (called SP–I3^mEos^) is recruited to p97 by p37, addition of ATP initiates the unfolding of I3^mEos^. This process can be directly monitored by the decrease in the red fluorescence emission from the mEos tag.

**Extended Data Fig. 1:**
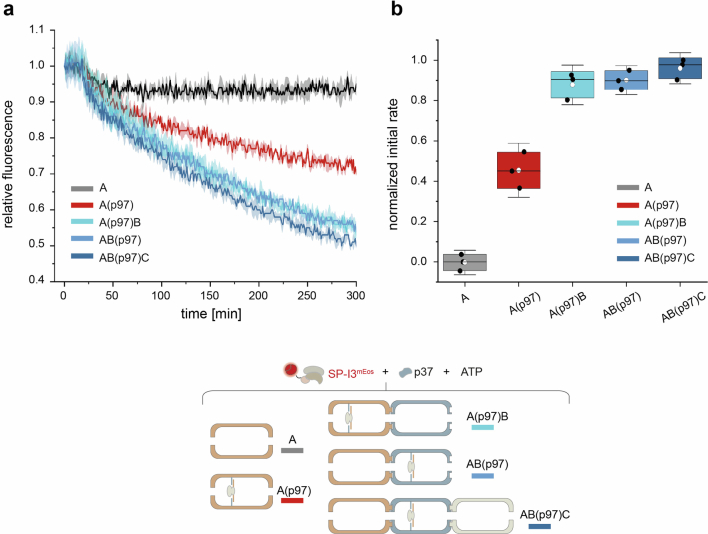
Substrate unfolding activity of p97 in multi-compartment DNA origami constructs. **(a)** The unfolding activity of p97 was monitored by following the decrease in the red fluorescence signal at 540/580 nm (ex/em) of a photoactivated I3^mEos^ substrate in complex with SDS22-PP1 and in presence of p37 adapter, upon addition of a prewarmed E-Mix (ATP and creatine phosphate-based enzymatic recycling system). The A(p97) construct (red) was compared to two- and three-compartment structures bearing a void chamber either downstream, upstream or on both sides. These are respectively indicated as A(p97)B (light blue), AB(p97) (middle blue) and AB(p97)C (dark blue). A void DNA compartment (A, grey) was used as reference. Average time- course profiles from *n* = 3 technical replicates are shown. **(b)** Normalized initial rates calculated from the slopes of the profiles in (a) in the interval between 50 min and 200 min. Data are presented as mean ± s.d. from *n* = 3 technical replicates per sample. Whiskers are 1.5 s.d.; mean (white dots) and median (black lines) are indicated for each data set. No outliers have been excluded. Source data

We then prepared two identical solutions of A(p97): one was treated with DNA strands fully complementary to the PA sequence (full-cPA); the other was treated with an equal amount of buffer. Addition of full-cPA led to the detachment of p97–cPA from the compartment with concurrent formation of a full PA/cPA duplex at the inner DNA walls (Fig. 4a–c and Supplementary Fig. 25). The kinetic assays performed on both solutions indicated a strong impact of compartmentalization on the rate of substrate unfolding, with A(p97) being about threefold faster than the displaced p97–cPA in presence of empty chambers (Fig. 4g,h, red versus pink). Control experiments using the same concentration of p97 and p97–cPA showed a negligible effect of the cPA strands and fused HaloTag domains on p97 activity (Fig. 4g,h, light versus dark grey). No substantial effect was observed when using a DNA origami compartment devoid of inner PAs, further confirming that the p97 machine must be truly confined within the DNA host to work at higher speed (Fig. 4h, white, and Supplementary Fig. 26). Interestingly, A(p97) constructs equipped at both entries with one or two lids of different pore sizes (Fig. 4d–f) showed further enhanced rates of substrate unfolding, especially A^L4^(p97), which featured a constrained room upstream of p97 (Fig. 4h, brown). The same outcome was observed in multi-compartment constructs, where the attachment of one or two void chambers next to A(p97) accelerated the unfolding reaction by about twofold (Extended Data Fig. 1).

Altogether, these results indicate that the spatial confinement of p97 increases the rate of substrate unfolding and that this rate scales with the size of the compartment, particularly upstream of p97. As suggested for other DNA-scaffolded enzyme systems, spatial confinement may enhance the reaction rate by favouring the conformational stability of the bound protein^35–37^ or increasing the local substrate concentration^38,39^. Our findings indicate that this effect can be boosted by enlarging the confined space around the protein, especially near the substrate recruitment domains, probably further reducing substrate escape to the bulk solution.

## Proteolytic degradation in the downstream module

aCt was covalently modified at lysine residues with a fluorescently labelled and thiol-modified single-stranded DNA (cPA^FAM^ or cPA^Cy5^) (Supplementary Fig. 27). The 1:1 enzyme:DNA conjugate (aCt–cPA) was successfully isolated and immobilized within a DNA origami compartment (called B) modified with 6PAs, resulting in 65% yield of B(aCt) complex (Supplementary Figs. 28–30).

Enzymatic assays using a chromogenic substrate showed that the *K*_M_ value of aCt increased by ca. 1.5-fold upon its covalent conjugation to DNA but was not further affected by encapsulation of the conjugate within the DNA compartment (Fig. 4i). Conversely, the turnover number (*k*_cat_) was largely affected by spatial confinement: B(aCt) performed about 5-fold faster than unbound aCt at the same concentration and up to 13-fold faster when containing 6PAs due to the higher number of immobilized aCt molecules (Fig. 4i, Supplementary Figs. 31–33 and Supplementary Table 3). These findings further confirm that immobilization within the cavity of a DNA compartment increases the catalytic efficiency of an enzyme, in agreement with previous reports^21,40,41^.

We then performed a proteolytic digestion of the SP–I3^mEos^ complex, using either native aCt, aCt–cPA or B(aCt), and analysed the products of enzymatic fragmentation by gel electrophoresis (Fig. 4j and Supplementary Fig. 34). The data showed that, in all cases, the I3 portion of the complex was rapidly and almost completely degraded, probably due to the intrinsically disordered conformation of this small protein. In contrast, the more compact mEos moiety displayed greater resistance to proteolysis, an issue that we tried to overcome by coupling the proteolytic digestion of I3^mEos^ directly after its unfolding by p97.

## Substrate unfolding and degradation by the modular chimera

We first tested the permeability of our DNA compartment to various molecular cargos (Supplementary Figs. 35–37). The data showed that, whereas short oligonucleotides and aCt could enter in a DNA compartment equipped with front and back lids, protein cargos larger than 45 kDa could not, confirming also recent findings^42^. Hence, we deduce that the DNA layers of our compartment are not permeable to the SP–I3^mEos^ complex, which is ca. 140 kDa in size, and that, although this complex can enter the chamber from either the front or back aperture, the biased p97 orientation will favour the accumulation of unfolded substrate downstream of p97.

**Fig. 5:**
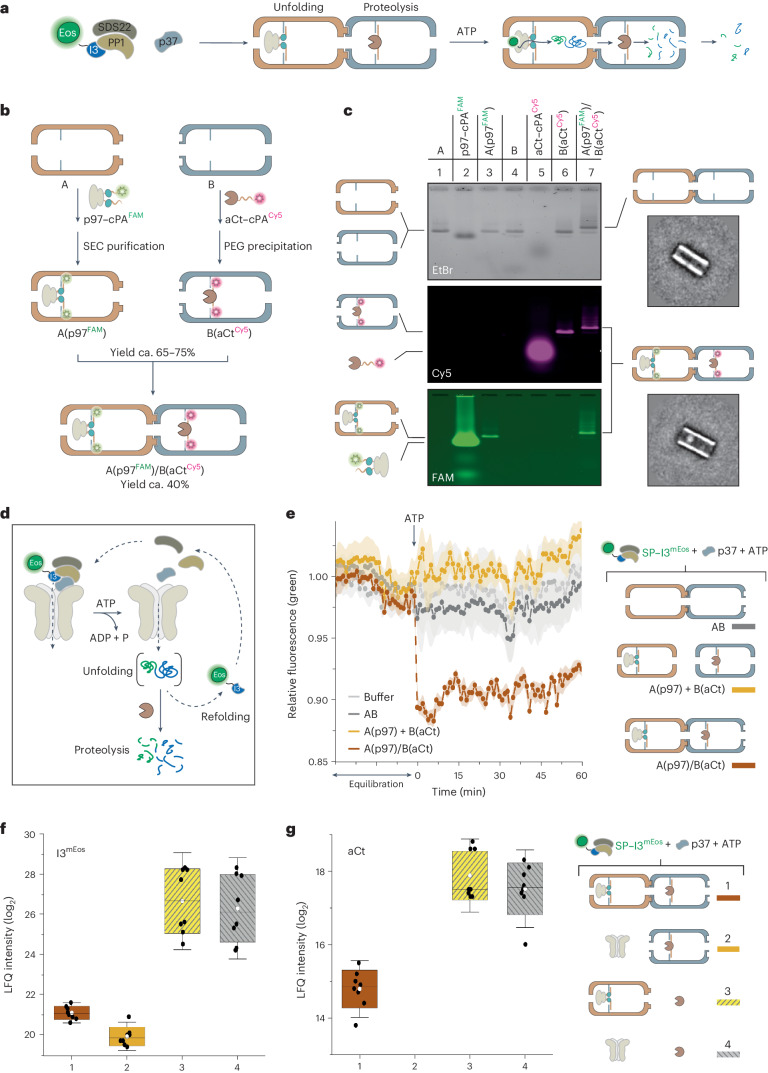
Assembly and catalytic function of the DNA origami chimera. **a**, Schematic representation of the modular chimera and its functioning. **b**, The individual proteins were encapsulated in distinct compartments, previously purified by ultrafiltration, and the resulting DNA-protein complexes were isolated by SEC or PEG-assisted precipitation. Finally, the two modules were mixed in equimolar amount and linked in a pre-defined order through hybridization at shape-complementary edges (more details in the Methods section). **c**, p97 and aCt conjugates were fluorescently labelled to favour identification of the species by AGE (lanes 2 and 5). Individual compartments, each loaded with its corresponding protein (A(p97^FAM^), lane 3 and B(aCt^Cy5^), lane 6), were purified and combined into A(p97^FAM^)/B(aCt^Cy5^) (yields in Supplementary Table 2). Successful formation of the target construct was verified by the co-localization of the fluorescence signals associated to the proteins and their co-migration with the AB origami structure (lane 7). TEM imaging of the protein-loaded structures (right) showed correct location of p97 (aCt was not visible due to the limited resolution of the microscope). Class averages are shown in the insets (*n* = 354 and *n* = 680, for AB and A(p97)/B(aCt), respectively). **d**, Substrate unfolding assays were performed by monitoring the decrease of the fluorescence signal of green SP–I3^mEos^ (490/520 nm ex/em), upon addition of ATP. Sustained loss of green fluorescence indicated proteolysis. **e**, Average time-course profiles from *n* = 2 biological replicates, with *n* = 3 technical replicates per sample. The p97/aCt-loaded chimera (brown) was compared with an equimolar mixture of the two isolated compartments (yellow). AB and buffer were used as reference (dark and light grey). **f**,**g**, Proteomic analysis of the products of proteolytic digestion of I3^mEos^ (**f**) and autodigestion of aCt (**g**) generated by the modular chimera construct (1) compared with control samples, containing only one of the two proteins in the encapsulated form (2 and 3) or both proteins in the unbound form (4). The data were acquired after 10 min incubation at 37 °C (aCt autodigestion in 2 was below the detection limit). LFQ intensity is reported in a log_2_ scale (one LFQ unit equals a twofold intensity change). The data are presented as mean ± s.d. from *n* = 2 biological replicates, with *n* = 4 technical replicates per sample. The whiskers are 1.5 s.d.; mean (white dots) and median (black lines) are reported for each data set. No outliers have been excluded. Source data

We then linked the segregation and unfolding module, A(p97), to the downstream proteolytic module, B(aCt), to reconstitute a substrate unfolding and degradation pathway (Fig. 5a). Stepwise assembly and purification of the full construct, A(p97)/B(aCt), was performed as illustrated in Fig. 5b. Protein-loaded compartments were characterized by AGE, using fluorescently labelled proteins (p97–cPA^FAM^ and aCt–cPA^Cy5^) (Fig. 5c). The results confirmed successful attainment of the individual modules and their further binding into the desired 19 MDa chimera (A(p97^FAM^)/B(aCt^Cy5^); Fig. 5c, lane 7). TEM imaging of the final product showed about 56% yield of AB, with 40% of the structures containing one p97 protein at the expected position (Fig. 5c, bottom inset, and Supplementary Fig. 38). The presence of aCt in compartment B was more difficult to observe due to the limited resolution of our TEM microscope. Notably, any attempt to immobilize both enzymes within the same compartment failed because of the concurrent proteolytic degradation of p97. This pinpoints the advantage of a modular strategy to couple enzymatic reactions while minimizing undesired off-target interactions.

**Extended Data Fig. 2:**
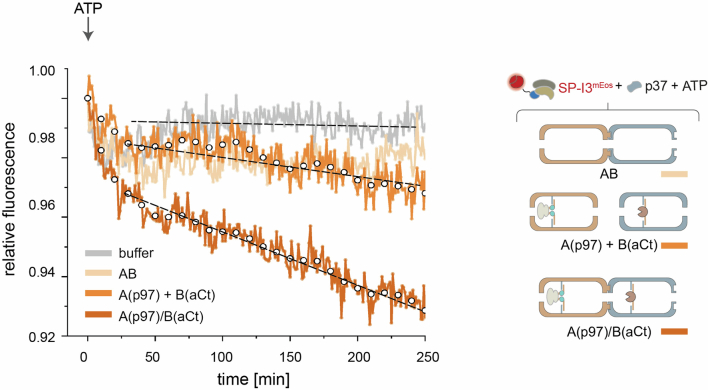
Substrate unfolding activity of p97 in presence of proteolysis. The unfolding activity of p97 was monitored by following the decrease in the red fluorescence signal at 540/580 nm (ex/em) of a photoactivated I3^mEos^ substrate in complex with SDS22-PP1 and in presence of p37 adapter, upon addition of ATP. The modular chimera A(p97)/B(aCt), bearing p97 in the first chamber and aCt in the second chamber (brown), was compared to an equimolar mixture of the two individual compartments, A(p97) + B(aCt) (orange). A void two-compartments construct AB (light orange) and a buffer solution (grey) were used as reference. The presence of an additional confined space in the vicinity of p97 increases the rate of p97- mediated substrate unfolding, as observed for multi- compartment structures devoid of aCt (Extended Data Fig. 1). The initial rate of substrate unfolding in the chimera construct was roughly 2-fold the rate exhibited by an equimolar mixture of individual modules, matching the same results observed in absence of aCt. Initial rates were estimated in the interval between 50 min and 200 min, using the profile of a buffer solution as reference. This was done to visualize, and exclude from analysis, the contribution to fluorescence decrease caused by dilution. The red-based assay is not sensitive to the presence of aCt because (i) aCt cannot degrade the folded form of the substrate (see also gels in Supplementary Fig. 34) and (ii) the degradation of the unfolded form of the cleaved substrate, even if happening, is not visible. The time-course profiles are the result of *n* = 4 technical replicates. Source data

To characterize the unfolding activity of the A(p97)/B(aCt) modular chimera, we utilized the native ‘green’ form of the I3^mEos^ substrate, which, in contrast to the ‘red’ mEos variant, refolds upon exiting the p97 pore. Thus, if visible, loss of green fluorescence is a direct readout for unfolding-coupled proteolysis (Fig. 5d)^32^. We observed ca. 10% loss of fluorescence signal immediately after addition of ATP, but only in the A(p97)/B(aCt) construct, that is, in the sample where the two biocatalytic modules were physically connected (Fig. 5e, brown). Contrarily, no appreciable change was visible for an equimolar mixture of individual modules at the same concentration (A(p97) + B(aCt); Fig. 5e, yellow). This indicates that the proteolytic digestion of the substrate increases when coupled directly after the unfolding process. Conversely, the simultaneous occurrence of the two processes in distinct and physically separated modules results in rapid refolding of the substrate and negligible proteolysis, in agreement with the sodium dodecyl sulfate (SDS) assays (Supplementary Fig. 34). The same experiment carried out on the red form of the substrate further proved the effect of spatial confinement on the rate of substrate unfolding (Extended Data Fig. 2).

Finally, we used mass spectrometry to analyse the products of substrate degradation by the A(p97)/B(aCt) chimera (Fig. 5f, sample 1 and Supplementary Figs. 39–41). Control samples containing either aCt, p97 or both enzymes as unbound species were also prepared (samples 2–4). The data showed that connecting the aCt module downstream of the p97 module almost doubled the efficiency of I3^mEos^ degradation (1 versus 2). Moreover, immobilizing aCt within the compartment shielded the protease from autoproteolysis (Fig. 5g, 1–2 versus 3–4), as also observed by AGE (Supplementary Fig. 30).

## Engineering a substrate-specific biocatalytic pathway

**Extended Data Fig. 3:**
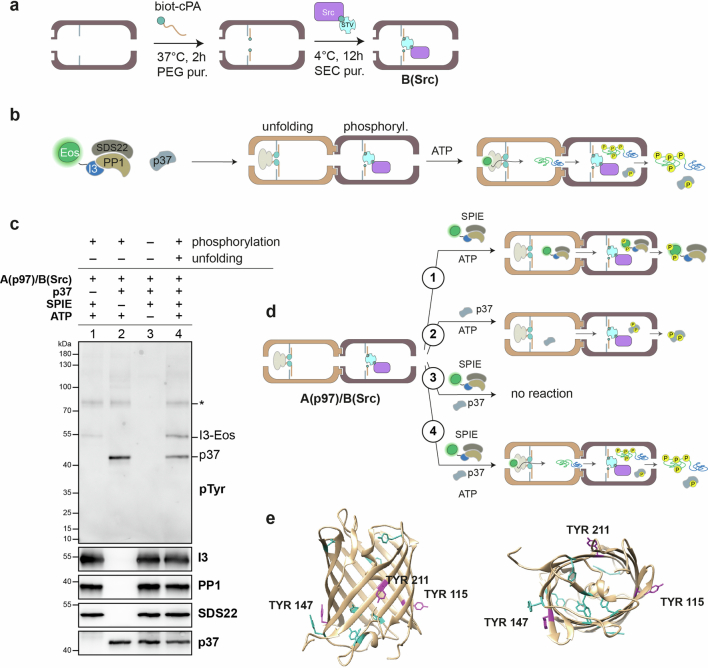
Enhanced unfolding- assisted phosphorylation of mEos at tyrosine residues. **(a)** A DNA origami cage, previously functionalized with biot- modified cPA strands, was bound to biotin-modified Src kinase through streptavidin. **(b)** The resulting encaged kinase (B(Src)) was linked downstream of a DNA-encaged p97, leading to a chimera construct, A(p97)/B(Src), that performs a phosphorylation reaction at the tyrosine residues of a previously unfolded substrate. (**c, d**) The extent of mEos phosphorylation was analyzed in absence (1) and presence (4) of previous p97-assisted mEos unfolding and control samples were prepared to verify correct kinase functioning and the extent of background phosphorylation (2 and 3). Proteins were separated by SDS-PAGE, blotted onto a nitrocellulose membrane, and probed with indicated antibodies (in bold). The asterisk denotes binding of pTyr antibody to a trace of contaminant protein. The data show that phosphorylation of mEos at tyrosine residues is enhanced by previous unfolding of the substrate by p97 (cfr. intensity of I3^mEos^ band in lane 4 with the same band in lane 1). In addition, while p37 is largely phosphorylated by Src, independently of p97 activity, SDS22 and PP1 are not phosphorylated. **(e)** Crystal structure of the mEos2 substrate (PDB 3S05), viewed along a direction perpendicular to the central axis (left panel) and along the central axis (right panel). TYR 115, TYR 147 and TYR 211 (in magenta) are predicted to be phosphorylated by the Src kinase. Most of the other TYR residues (in cyan) are buried within the protein barrel and might become more accessible to phosphorylation upon substrate unfolding.

To extend the applicability of our approach, we coupled the p97-driven unfolding process to a phosphorylation process catalysed by Src, a tyrosine kinase that plays a role in the regulation of embryonic development and cell growth^43^. Using this artificially engineered biocatalytic cascade, we expected to increase the extent of phosphorylation at tyrosine residues that were masked in the folded form of the substrate and would become more accessible only after substrate unfolding (Extended Data Fig. 3).

We obtained the compartmentalized chimera, A(p97)/B(Src), using modular assembly and analysed the extent of substrate phosphorylation in absence and presence of previous unfolding. Our results showed that, upon addition of ATP, phosphorylation of the substrate occurred in both scenarios. However, physical coupling of the two enzymatic modules in the intended sequential order notably enhanced the amount of phosphorylated tyrosine residues in I3^mEos^ only. This proves the occurrence of unfolding-assisted phosphorylation in a substrate-specific manner and demonstrates the general applicability of our approach for executing post-translational modifications on virtually any I3-tagged protein.

## Conclusions

Our nanoscale model merges two fundamental aspects of modular enzymes: the spatial confinement of biocatalytic reactions within specialized units and the spatial proximity of these units in a defined sequential order. Using this design principle, we created a prototype of a modular enzyme capable of recognizing, unfolding and digesting a specific substrate, thus mimicking the structural organization and functioning of the 26S proteasome. We also demonstrated that this principle can be extended to reaction pathways not yet found in cells, setting the bases for engineering potentially any type of biocatalytic cascade on a selected substrate.

Compared with previous DNA-confined enzymes, our recognition and unfolding module tackles numerous structural and functional challenges. The encaged p97 machine (ca. 10 MDa in size) demands active movement, multiple binding partners and precise orientation for functionality. We achieve substrate specificity via the I3 domain: the key to access the nanochannel through the p97 gateway. By fusing the I3 domain to other molecules, including DNA, our approach may permit customized modifications on different substrates besides peptides or small proteins. Although only specific substrates follow the intended unfolded-assisted pathway, our construct is still permeable to molecules <45 kDa in size, which may potentially interfere with the reaction cascade or reduce its efficacy. Attaching a lid at the channel’s terminal end can mitigate this problem, but assembly yields of multi-compartment constructs may become too low for further manipulation. We are currently exploring silica and co-polymers coating procedures to reduce the permeability of the DNA compartments.

Future enhancements of our modular strategy may involve incorporating additional structural and functional features such as semipermeable barriers and stimuli-responsive motifs. These additional elements may help regulate the translocation of intermediate species between modules or mediate the entry/exit of molecules in/from channels via light, pH or ligand interactions. Hence, by refining control of reaction flow and endowing compartments with advanced capabilities, our approach can inspire the development of new pathways, fostering the advancement of miniature laboratories with capabilities not found in natural systems.

## Methods

### Chemicals and DNA origami assembly

Unless stated differently, all chemicals were purchased from Merck or Thermo Fisher; consumables were from VWR, Eppendorf or Millipore. DNA sequences were purchased from Merck or IDT. cPA sequence for protein conjugation was GTGGAAAGTGGCAATC; PA sequence for protein immobilization was CTTCACGATTGCCACTT TCCAC (the underlined bases represent the toehold region). Full-cPA sequence for protein displacement from the compartment was GTGGAAAGTGGCAATCGTGAAG. All DNA sequences for origami assembly are reported as Supplementary Data 2. Buffers were TEMgX (5 mM Tris base, 1 mM EDTA, *X* mM MgCl_2_, pH 8.0; *X* = 12.5, 16 or 20) and TBEMg (40 mM Tris base, 20 mM boric acid, 2 mM EDTA and 12.5 mM MgCl_2_). DNA origami structures were designed with caDNAno (https://cadnano.org/). The scaffold sequence p7560 was purchased from tilibit nanosystems and amplified as previously described^44^. Assembly of N and E origami structures was performed by mixing the scaffold strand with fivefold staple strands in 1× TEMg20 (65 °C for 10 min, 52 °C for 3 h). Pre-assembled N and E monomers were mixed in equimolar amounts and incubated for 4 h, at 40 °C to obtain NE. Individual NE structures were pre-activated at their edges to enable multimerization. Upon ultrafiltration (100 kDa molecular weight cut-off (MWCO), seven times in 1× TEMg5), the monomers were mixed in equimolar amounts and incubated at 4 °C overnight. Assembly of the lid was done by mixing the scaffold with tenfold staple strands in 1× TEMg16 (75 °C to 40 °C, −1 °C per 30 min; hold at 21 °C). Attachment of one or two lids to the origami compartments was done by mixing filter-purified structures in equimolar amounts and adjusting the magnesium ions concentration to 20 mM (37 °C for 12 h, 8 °C overnight). Thermal assembly was carried out in a Thermocycler Mastercycler nexus gradient (Eppendorf), using a lid temperature 10 °C above the highest assembly temperature. DNA concentration was measured by recording the absorption at 260 nm using a DS11 spectrophotometer (De Novix). The signal from the buffer was subtracted, and the concentration was calculated via the extinction factor provided by the vendor. DNA origami concentration was estimated using a molar extinction coefficient of 9.32 × 10^6^ cm^−1^ M^−1^. DNA origami structures were purified from excess staple strands by polyethylenglycol (PEG)-assisted precipitation^44^, or by ultrafiltration, using 100 kDa or 50 kDa MWCO centrifugal devices. Samples were washed up to seven times and centrifuged at 8,000*g* for 5 min at 21 °C.

### Proteins

All proteins used for in vitro reconstitution of the segregation–unfolding pathway were obtained as previously described^33^. Briefly, human p97 constructs were expressed with a His_6_ tag and purified by affinity chromatography and size-exclusion chromatography (SEC, using a Superose 6 10/300 column). Human p37 was expressed in *Escherichia coli* with an N-terminal glutathione-S-transferase (GST)-tag. The protein was purified by GST-tag affinity chromatography with subsequent GST-tag removal and further purified by SEC. The complex of SDS22, PP1 and His_6_-I3-mEos3.2 was expressed in High Five insect cells and purified via NiNTA affinity chromatography, ion exchange chromatography and SEC. For conversion of green mEos to the red form, SP–I3^mEos^ was irradiated at 365 nm wavelength for 120 min on ice using a 100 W Blak-Ray, B-100AP UV lamp (Fisher Scientific). aCt (from bovine pancreas, 350 U mg^−1^, #1.02307) was purchased from Sigma-Aldrich. A list of proteins used in this study, including their molecular weights and the size of their complexes, is reported in Supplementary Table 1.

### Synthesis of the p97–cPA conjugate

The chloroalkane (CH)-modified cPA oligonucleotide was obtained by incubating the amino-modified cPA with tenfold excess *N*-hydroxysuccinimidyl-modified CH for at least 2 h, at 21 °C. The CH-cPA was isolated either by precipitation in a 1:8 (v:v) solution of 5 M ammonium acetate/isopropanol or by gel extraction, followed by ultrafiltration (3 kDa MWCO). Successful attainment of the product was confirmed by matrix-assisted laser desorption/ionization, using the oligonucleotide solution (10 µM to 100 µM) mixed with an equimolar amount of matrix. Up to four different oligonucleotides were used as mass standard. Laser power, spot size and frequency were adjusted to allow detection of the heaviest oligonucleotide; mass spectra were baseline subtracted. Then, the cPA-CH conjugate was mixed to purified HaloTag-fused p97 (10:1) in storage buffer at 8 °C overnight. The reaction mixture was purified by SEC. Fractions of interest were pooled and concentrated by ultrafiltration (100 kDa MWCO). Successful conjugation was verified by SDS polyacrylamide gel electrophoresis (PAGE), and p97–cPA concentration was estimated using a standard curve.

### Synthesis of the aCt–cPA conjugate

*N*-[α-maleimidoacetoxy] succinimide ester (AMAS, 20 mM, in dimethyl sulfoxide) was mixed with aCt (4 mM) at a molar ratio of 28:1 and incubated at room temperature for 2 h. Thiol-modified oligonucleotides were treated with Tris(2-carboxyethyl)phosphine (TCEP) at room temperature for 20 min, and the excess of TCEP was then removed by ultrafiltration (3 kDa MWCO) or isopropanol precipitation. The reduced oligonucleotides were added to the AMAS-coupled aCt in a molar ratio of 1:5, and the mixture was kept at 4 °C overnight. The aCt–cPA conjugate (1:1) was isolated by ion-exchange chromatography (proFIRE, Dynamic Biosensors) and concentrated by ultrafiltration (20 kDa MWCO).

### Binding of proteins in the DNA origami compartment

The p97–cPA conjugate was mixed with the DNA origami compartment in tenfold molar excess over the protruding arms, and the solution was incubated in 1× TEMg20 at 8 °C overnight. The DNA-encaged p97 was isolated by SEC, and fractions of interest were pooled and concentrated by ultrafiltration (100 kDa MWCO). Alternatively, streptavidin-coated magnetic beads (35 µl) were washed four times with 400 µl TEN100 buffer (10 mM Tris-HCl, 1 mM Na_2_EDTA, 100 mM NaCl, pH 7.5) and incubated with 0.5 nmol of biotin-modified T_10_-PA strand at 8 °C overnight. The beads were washed three times with 400 µl storage buffer and incubated with 20 µl reaction mixture for at least 2 h, at 8 °C. DNA origami samples were recovered by washing off the beads. Alternatively, DNA origami samples were first immobilized onto the beads and pulled down by strand-displacement. Successful encapsulation of p97 was verified by TEM and sample concentration was estimated by absorbance at 260 nm. The aCt–cPA conjugate was mixed with the DNA origami compartment in twofold molar excess over the protruding arms and incubated in 1× TEMg20 (37 °C for 10 min; 36 °C to 10 °C, at −1 °C per 3 min). Unbound protein was removed by PEG-assisted precipitation. The DNA origami pellet was redissolved in 1× TEMg20 and stored at 4 °C until usage. Sample concentration was estimated by gel using a standard curve.

### Dynamic light scattering

Unpurified N and E structures were mixed in equimolar amount (10 nM), and the change in the hydrodynamic radius was measured over time (Zetasizer, Malvern Analytics). Different temperatures and Mg concentrations were used in distinct experiments. The refractive index for the buffer was obtained by the Zeta Analyzer Software built-in calculator. Attenuator 11 was chosen to keep the particle count approximately constant.

### Gel electrophoresis analysis

For AGE, a solution of 1% agarose (Lonza) in 1× TBEMg was used for casting the gels. Gels were run at 80 V for 1.5 h in ice bath and stained with ethidium bromide (EtBr) or SYBR Green I. For SDS PAGE, a 15% polyacrylamide solution was used. Running conditions: 1 h at 180 V. The gels were first stained with SYBR Gold and then with Coomassie R250 staining solution (0.06%). Running buffer (1.5 M Tris–HCl, pH 8.8), stacking gel buffer (1 M Tris–HCl, pH 6.8), electrophoretic buffer (25 mM Tris, 250 mM Gly and 10% SDS), and loading dye (0.6 M Tris–HCl, 40% glycerol, 8% SDS and 0.4 mg ml^−1^ bromophenol blue) were used. Before staining, fluorescently labelled samples were visualized by scanning the gel with a Typhoon FLA9000 (GE Healthcare Life Sciences) at different wavelengths. Stained gels were also scanned with a Typhoon FLA9000 and visualized upon UV illumination.

### AFM

Five microlitres of sample was deposited on a freshly cleaved mica surface (Plano GmbH) and adsorbed for 3 min at room temperature. After washing with ddH_2_O, the sample was dried under gentle argon flow. Samples were scanned in ScanAsyst Mode using a MultiMode microscope (Bruker) equipped with a Nanoscope V controller, using cantilevers with sharpened pyramidal tips (ScanAsyst-Air or ScanAsyst-Fluid+ tips, Bruker). Several AFM images were acquired from different locations of the mica surface to ensure reproducibility of the results. All images were analysed by using the NanoScope Analysis 1.5 software.

### Negative-stain TEM

Carbon-coated copper grids (400 mesh, Quantifoil) were glow charged at 10 mA for 30 s and coated with 5 µl sample for 2 min. For DNA origami, 10 nM solution was applied to the grid, dried off with a filter paper and washed with 5 µl of a 1% uranyl formate staining solution. Five microlitres of staining solution was then added, incubated for 2 min and dried with a filter paper. For protein samples, 200 nM solution was incubated for 2 min on the grid and dried off with a filter paper, then washed two times with 5 µl water and two times with staining solution before applying 5 µl staining solution for 2 min. Grids were further dried in air for a few minutes before investigation with TEM (JEOL JEM 1400Plus equipped with a 120 kV beam from a LaB_6_ or tungsten filament). Images were manually obtained near the Scherzer defocus (highest contrast near the focus). The structures of interest were identified using the cell counter plugin for the software FiJi. The number of individual DNA origami structures imaged was evaluated to ensure statistical relevance. Class averages were obtained by Eman2, and the images were checked for absence of drift or stigmatism using the contrast transfer function (CTF). A spherical aberration of 3.4 was used and CTF was fitted as recommended for negative-stain TEM. If not stated differently, box size was 200 pixels and particles were picked manually at 7.42 Å per pixel resolution, sorted and averaged in 32 classes. Contrast was set at 60–80, and a low-pass filter of 20 Å was used to obtain two-dimensional (2D) class averages.

### Cryo-EM and protein prediction model

Ten microlitres of concentrated sample at 200 nM were applied to plasma cleaned Quantifoil R1.2/1.3 holey carbon grids. Loaded grids were plunge-frozen using a Vitrobot (FEI, Thermo Scientific) at the following settings: humidity 95%, temperature 20 °C, wait time 0 s, blot time 3 s, drain time 0 s, blot force 3. Data were acquired on a Talos Arctica electron microscope operating at 200 kV. The equipment included a Falcon 3EC detector and SerialEM software. Micrographs were recorded with an electron dose of ∼23 e Å^−2^ s^−1^ and later adjusted to a pixel size of 1.997 Å in RELION 4.1. Recorded micrographs were motion-corrected using MotionCor2, and the CTF was estimated using CTFFIND-4.1. Micrographs were manually assessed for astigmatism, and 2,486 micrographs were selected for further analysis. A total of 57,000 particles were manually picked, extracted and subjected to reference-free 2D classification. Two-dimensional classes were further used in multiple cycles of 2D and 3D classifications to remove falsely aligned particles and to investigate intrinsic heterogeneity. The refined 3D map was generated using an initial low-resolution 3D model, with a total of ca. 14,400 particles. Finally, post-processing of the map was done using a low-pass-filtered mask to calculate the Fourier shell correlation and to estimate the final resolution, which was 16 Å. The 2D slice side view was generated from a larger dataset and included ca. 27,000 particles. The experimental parameters were slightly modified during grid preparation to blot time 6 s and blot force 2. Cryo-EM density maps were analysed using the ResolutionMap algorithm^45^. The atomic model of the apo p97-HaloTag protein was generated using AlphaFold2 (ref. ^46^) embedded into the UCSF ChimeraX software package^47^ and run through the ColabFold interface^48^. Fitting into the cryo-EM density map and generation of the 3D model of the DNA-protein construct was done using ChimeraX.

### Substrate unfolding assay

A master mix containing SP–I3^mEos^ (35 nM) and p37 (500 nM) in unfolding buffer (25 mM HEPES, 100 mM KCl and 5 mM MgCl_2_, pH 7.4) was prepared before each experiment and aliquoted for technical replica. Samples were placed on 384 well-plates; p97 or A(p97) (2 nM) was added, and the reaction mixtures were equilibrated for 20 min at 37 °C. Pre-warmed ATP (2 mM) was quickly added, and the solutions were mixed by pipetting; final volume was 50 μl per sample. The fluorescence signal was monitored over time using a Tecan Spark 10 and collecting one data point per minute for approximately 5 h (540 nm/580 nm, ex/em for red I3^mEos^; 490 nm/520 nm for green I3^mEos^). Fluorescence values relative to the equilibration phase were considered for analysis. The initial rates were calculated from the slopes of the curves between 50 min and 200 min, comparing each curve with a reference signal from a buffer solution; this was done to distinguish the fluorescence changes due to effective substrate unfolding from those caused by dilution of the sample after addition of ATP. The average values obtained within the same experiment were normalized to the highest rate observed.

### Chymotrypsin enzymatic activity

Chromogenic substrate Suc-AAPF-pNA (Sigma-Aldrich, #S7388) was resuspended in dimethyl sulfoxide to reach a concentration of 20 mM. Enzymatic activity assays were performed by incubating aCt with different concentrations of substrate (from 0 to 400 or 1,000 µM) in activity buffer (20 mM Tris base with 5 mM MgCl_2_ and 3 mM CaCl_2_, pH 8) at 30 °C for 2 h. The progress of the catalytic reaction was monitored over time by measuring the absorbance of each sample at 410 nm.

### Proteomics analysis

SP–I3^mEos^ (2.1 μM) was mixed with p37 (2.1 μM) in unfolding buffer. Afterwards, chymotrypsin (unbound: 12 nM, encaged: 2 nM) and/or p97 (unbound 2 nM, encaged 2 nM) were added to reach a final volume of 18 μl. The pre-warmed ATP was added to a final concentration of 2 mM, and the reaction mixture was incubated at 37 °C for 5 min, 10 min, 30 min or 60 min. Identical samples were prepared to analyse the progress of the reaction at distinct time points. The enzymatic reaction was stopped by adding a fourfold excess of ice-cold acetone (80 µl; Sigma-Aldrich, 270725) to induce protein precipitation. The samples were incubated overnight at −20 °C. Proteins were sedimented by centrifugation at 18,213*g* at 4 °C for 30 min, and the supernatant of each sample was transferred to a fresh vessel. The solvent was removed under reduced pressure at 30 °C in a centrifugal vacuum concentrator (Eppendorf), and the peptides were dissolved in 50 µl benzonase-containing buffer (10 u benzonase per sample; EMD Millipore, 71206), followed by incubation at 37 °C for 2 h. Each sample was acidified with 1 µl formic acid (FA; Fisher Chemical, A117-50) and peptides were loaded onto EvoTips (Evosep, EV2001). Samples were then separated using the Evosep One UPLC system equipped with an Evosep, EV1064 analytical column (60/100 samples per day; 21 min proprietary preformed gradient; solvent A: 0.1% FA; solvent B: 0.1% FA, 99.9% acetonitrile; variable flow set by Evosep One). Mass spectra were acquired on an Orbitrap Elite mass spectrometer (Thermo Fisher Scientific). MS1 data acquisition was done in a *m*/*z* range of 300–1,500 at a resolution of 60,000 (*m*/*z* = 400). Data-dependent MS2 spectra were acquired in the Iontrap at rapid scan range using a topN = 15 loop with a dynamic exclusion time set to 30 s. Fragmentation by collision-induced dissociation was performed at a normalized collision energy of 35. Data processing was done in Proteome Discoverer 2.5 (Thermo Fisher Scientific) using the SequestHT search engine. Statistical analysis was done using Perseus 2.0.7.0. Data are given in label-free quantification (LFQ) intensity units and represented in a log_2_ scale (one LFQ is a twofold change in relative intensity).

### Unfolding-assisted phosphorylation of mEos at tyrosine residues

The GPS software tool^49^ was used to select the most suitable kinase candidate for phosphorylation of mEos. Highest scores were found for the Src tyrosine kinase and Tyr 115, Tyr 147 and Tyr 211 of mEos. Biotinylated tyrosine kinase Src (Src*biotin, Carna Biosciences, 08-473-20N) was mixed with streptavidin in equimolar amount and incubated on ice for 2 h. Biotin-modified cPA strand (biot-cPA) was mixed with B_6_ in a 12-fold excess and incubated at 37 °C for 2 h. Excess biot-cPA was removed by PEG precipitation, and the purified biotin-functionalized chamber was incubated with the Src mixture. The so-obtained B(Src) was purified by SEC and linked downstream of A(p97) to form the chimera structure A(p97)/B(Src). This construct (2 nM) was mixed with green SP–I3^mEos^ (35 nM), p37 (500 nM) and ATP (2 mM) and incubated at 37 °C for 2 h. Control samples were prepared similarly and used to verify the extent of off-target phosphorylation and on-target phosphorylation without prior substrate unfolding. Samples were analysed by western blotting using antibodies specific to phosphorylated tyrosine. SDS22 antibody B-6 (Santa Cruz Biotechnologies), UBXN-2 antibody (PMID 23649807), PP1γ antibody (E-9; Santa Cruz Biotechnology) were used at a dilution of 1:1,000. Anti-I3 (PMID 25298395) was used at a dilution of 1:200. Western blots were developed with a ChemoStar TS digital ECL imager (Intas) using ChemoStar software v.0.5.67 (Intas). Alexa Fluor 647-modified phosphotyrosine antibody (P-Tyr-01; ThermoFisher) was used at a dilution of 1:1,000, and fluorescence was recorded using a Typhoon FLA9000 scanner.

### Statistics and reproducibility

Information about the statistical relevance of the data is reported in the legends as number of data points collected (*n*), for a given number of technical or biological replicates. Box plots include data from mean ± standard deviation (s.d.); whiskers are 1.5-fold s.d.; white dots and black lines indicate, respectively, the mean and median value of each data set. No outliers have been excluded from analysis. AFM and TEM images shown in the figures are representative examples of wide-field views. TEM data were interpreted by resampling the images to obtain around 200 structures per subset. Alternatively, image to image variance was calculated. AGE and SDS gels shown in the figures are representative examples of similar experiments that yielded the same result. Reported kinetic assays are the averaged result of experiments conducted by different investigators. In all cases, reproducibility was observed. No data were excluded from the analyses, and the investigators were not blinded to allocation during experiments and outcome assessment.

### Reporting summary

Further information on research design is available in the Nature Portfolio Reporting Summary linked to this article.

## Online content

Any methods, additional references, Nature Portfolio reporting summaries, source data, extended data, supplementary information, acknowledgements, peer review information; details of author contributions and competing interests; and statements of data and code availability are available at 10.1038/s41565-024-01738-7.

## Supplementary information

Supplementary Information Supplementary Figs. 1–41 and Tables 1–3.

Reporting Summary

Supplementary Video 1 Video of the 3D cryo-EM reconstruction of A(p97).

Supplementary Data 1 Statistical source data for supplementary figures.

Supplementary Data 2 List of DNA sequences.

## Source data

Source Data Figs. 1–5 and Extended Data Fig. 3 Figure 1b: wide-field TEM images of p97. Figure 2a: TEM-averaged classes of N and E. Figure 2b: wide-field TEM images of NE. Figure 2c: TEM-averaged classes of NE. Figure 2e: TEM images of L. Figure 2g: wide-field TEM images of A^L^. Figure 2h: wide-field TEM images and class averages of A^2L^. Figure 2j: wide-field TEM images and class averages of AB. Figure 2k: TEM images of ABC. Figure 3b: unprocessed AGE of A(p97). Figure 3c: wide-field TEM images of A(p97). Figure 3e: slices of the 3D cryo-EM density map. Figure 4a: wide-field TEM images of A. Figure 4b: wide-field TEM images of A(p97). Figure 4c: wide-field TEM images of A_rel_. Figure 4d: wide-field TEM images of A^L4^(p97). Figure 4e: wide-field TEM images of A^L1^(p97). Figure 4f: wide-field TEM images of A^L41^(p97). Figure 4j: SDS gel B(aCt). Figure 5c: unprocessed gel A(p97)/B(aCt) chimera. Figure 5c (inset) TEM class average of AB. Extended Data Fig. 3c: unprocessed SDS gel and blots A(p97)/B(Src).

Source Data Figs. 4 and 5 and Extended Data Figs. 1 and 2 Figure 4g: relative fluorescence red versus time (single comp.). Figure 4h: normalized initial rates red (single comp.). Figure 4i: Michaelis–Menten (MM) parameters aCt. Figure 5e: relative fluorescence green versus time (chimera). Figure 5f: proteomics profile I3^mEos^. Figure 5g: proteomics profile aCt. Extended Data Fig. 1a: relative fluorescence red versus time (multi). Extended Data Fig. 1b: normalized initial rates red (multi). Extended Data Fig. 2: relative fluorescence red versus time (chimera).

## Data Availability

All data generated or analysed during this study are included in this published article and its Supplementary Information files, Supplementary Figs. 1–41 and Supplementary Tables 1–3. Cryo-EM maps and atomic models reported in this study have been deposited in the Electron Microscopy Data Bank (EMDB) under accession codes EMD-18538. The mass spectrometry proteomics data have been deposited to the ProteomeXchange Consortium via the PRIDE partner repository (https://www.ebi.ac.uk/pride/archive/) with the dataset identifiers PXD045825 and PXD050816. All other data can be provided by the corresponding authors on request. Source data are provided with this paper.

## References

[1] Chen, A. H. & Silver, P. A. Designing biological compartmentalization. *Trends Cell Biol.* **22**, 662–670 (2012). 22841504 10.1016/j.tcb.2012.07.002

[2] Urban, P. L. Compartmentalised chemistry: from studies on the origin of life to engineered biochemical systems. *N. J. Chem.* **38**, 5135–5141 (2014).

[3] Baumeister, W., Walz, J., Zuhl, F. & Seemuller, E. The proteasome: paradigm of a self-compartmentalizing protease. *Cell* **92**, 367–380 (1998). 9476896 10.1016/s0092-8674(00)80929-0

[4] Khosla, C. & Harbury, P. B. Modular enzymes. *Nature* **409**, 247–252 (2001). 11196653 10.1038/35051723

[5] Majumder, P. & Baumeister, W. Proteasomes: unfoldase-assisted protein degradation machines. *Biol. Chem.* **401**, 183–199 (2019). 31665105 10.1515/hsz-2019-0344

[6] Agapakis, C. M., Boyle, P. M. & Silver, P. A. Natural strategies for the spatial optimization of metabolism in synthetic biology. *Nat. Chem. Biol.* **8**, 527–535 (2012). 22596204 10.1038/nchembio.975

[7] Ren, H., Zhu, S. & Zheng, G. Nanoreactor design based on self-assembling protein nanocages. *Int J. Mol. Sci.* **20**, 592 (2019). 30704048 10.3390/ijms20030592 PMC6387247

[8] McConnell, S. A. et al. Designed protein cages as scaffolds for building multienzyme materials. *ACS Synth. Biol.* **9**, 381–391 (2020). 31922719 10.1021/acssynbio.9b00407

[9] Kramer, R. M., Li, C., Carter, D. C., Stone, M. O. & Naik, R. R. Engineered protein cages for nanomaterial synthesis. *J. Am. Chem. Soc.* **126**, 13282–13286 (2004). 15479082 10.1021/ja046735b

[10] Comellas-Aragones, M. et al. A virus-based single-enzyme nanoreactor. *Nat. Nanotechnol.* **2**, 635–639 (2007). 18654389 10.1038/nnano.2007.299

[11] Edwardson, T. G. W. et al. Protein cages: from fundamentals to advanced applications. *Chem. Rev.* **122**, 9145–9197 (2022). 35394752 10.1021/acs.chemrev.1c00877

[12] Rideau, E., Dimova, R., Schwille, P., Wurm, F. R. & Landfester, K. Liposomes and polymersomes: a comparative review towards cell mimicking. *Chem. Soc. Rev.* **47**, 8572–8610 (2018). 30177983 10.1039/c8cs00162f

[13] Dey, S. et al. DNA origami. *Nat. Rev. Methods Prim.* **1**, 13 (2021).

[14] Madsen, M. & Gothelf, K. V. Chemistries for DNA Nanotechnology. *Chem. Rev.* **119**, 6384–6458 (2019). 30714731 10.1021/acs.chemrev.8b00570

[15] Pfeifer, W. & Sacca, B. From nano to macro through hierarchical self-assembly: the DNA paradigm. *Chembiochem* **17**, 1063–1080 (2016). 27186937 10.1002/cbic.201600034

[16] Wagenbauer, K. F., Sigl, C. & Dietz, H. Gigadalton-scale shape-programmable DNA assemblies. *Nature* **552**, 78–83 (2017). 29219966 10.1038/nature24651

[17] Li, Y. et al. Hierarchical assembly of super-DNA origami based on a flexible and covalent-bound branched DNA structure. *J. Am. Chem. Soc.* **143**, 19893–19900 (2021). 34783532 10.1021/jacs.1c09472

[18] Zhou, Y., Dong, J., Zhou, C. & Wang, Q. Finite assembly of three-dimensional DNA hierarchical nanoarchitectures through orthogonal and directional bonding. *Angew. Chem. Int Ed. Engl.* **61**, e202116416 (2022). 35147275 10.1002/anie.202116416

[19] Grossi, G., Dalgaard Ebbesen Jepsen, M., Kjems, J. & Andersen, E. S. Control of enzyme reactions by a reconfigurable DNA nanovault. *Nat. Commun.* **8**, 992 (2017). 29051565 10.1038/s41467-017-01072-8 PMC5648847

[20] Hahn, J., Chou, L. Y. T., Sørensen, R. S., Guerra, R. M. & Shih, W. M. Extrusion of RNA from a DNA-origami-based nanofactory. *ACS Nano* **14**, 1550–1559 (2020). 31922721 10.1021/acsnano.9b06466

[21] Kosinski, R. et al. The role of DNA nanostructures in the catalytic properties of an allosterically regulated protease. *Sci. Adv.* **8**, eabk0425 (2022). 34985948 10.1126/sciadv.abk0425 PMC8730604

[22] Rabe, K. S., Muller, J., Skoupi, M. & Niemeyer, C. M. Cascades in compartments: en route to machine-assisted biotechnology. *Angew. Chem. Int. Ed. Engl.* **56**, 13574 (2017). 28691387 10.1002/anie.201703806

[23] Wilner, O. I. et al. Enzyme cascades activated on topologically programmed DNA scaffolds. *Nat. Nanotechnol.* **4**, 249–254 (2009). 19350036 10.1038/nnano.2009.50

[24] Linko, V., Eerikainen, M. & Kostiainen, M. A. A modular DNA origami-based enzyme cascade nanoreactor. *Chem. Commun.* **51**, 5351–5354 (2015). 10.1039/c4cc08472a 25594847

[25] Zhao, Z. et al. Nanocaged enzymes with enhanced catalytic activity and increased stability against protease digestion. *Nat. Commun.* **7**, 10619 (2016). 26861509 10.1038/ncomms10619 PMC4749968

[26] Kahn, J. S., Xiong, Y., Huang, J. & Gang, O. Cascaded enzyme reactions over a three-dimensional, wireframe DNA origami Scaffold. *J. Am. Chem. Soc.* **2**, 357–366 (2022). 10.1021/jacsau.1c00387 PMC8889550 35252986

[27] van den Boom, J. & Meyer, H. VCP/p97-mediated unfolding as a principle in protein homeostasis and signaling. *Mol. Cell* **69**, 182–194 (2018). 29153394 10.1016/j.molcel.2017.10.028

[28] Meyer, H. & van den Boom, J. Targeting of client proteins to the VCP/p97/Cdc48 unfolding machine. *Front. Mol. Biosci.* **10**, 1142989 (2023). 36825201 10.3389/fmolb.2023.1142989 PMC9941556

[29] Appel, W. Chymotrypsin: molecular and catalytic properties. *Clin. Biochem* **19**, 317–322 (1986). 3555886 10.1016/s0009-9120(86)80002-9

[30] Khan, Y. A., White, K. I. & Brunger, A. T. The AAA+ superfamily: a review of the structural and mechanistic principles of these molecular machines. *Crit. Rev. Biochem Mol. Biol.* **57**, 156–187 (2022). 34632886 10.1080/10409238.2021.1979460

[31] Twomey, E. C. et al. Substrate processing by the Cdc48 ATPase complex is initiated by ubiquitin unfolding. *Science* **365**, eaax1033 (2019). 31249135 10.1126/science.aax1033 PMC6980381

[32] Olszewski, M. M., Williams, C., Dong, K. C. & Martin, A. The Cdc48 unfoldase prepares well-folded protein substrates for degradation by the 26S proteasome. *Commun. Biol.* **2**, 29 (2019). 30675527 10.1038/s42003-019-0283-z PMC6340886

[33] Weith, M. et al. Ubiquitin-Independent Disassembly by a p97 AAA-ATPase Complex Drives PP1 Holoenzyme Formation. *Mol. Cell* **72**, 766–777.e6 (2018). 30344098 10.1016/j.molcel.2018.09.020

[34] Zhang, M. et al. Rational design of true monomeric and bright photoactivatable fluorescent proteins. *Nat. Methods* **9**, 727–729 (2012). 22581370 10.1038/nmeth.2021

[35] Zhou, H. X., Rivas, G. & Minton, A. P. Macromolecular crowding and confinement: biochemical, biophysical, and potential physiological consequences. *Annu. Rev. Biophys.* **37**, 375–397 (2008). 18573087 10.1146/annurev.biophys.37.032807.125817 PMC2826134

[36] Baumketner, A., Jewett, A. & Shea, J. E. Effects of confinement in chaperonin assisted protein folding: rate enhancement by decreasing the roughness of the folding energy landscape. *J. Mol. Biol.* **332**, 701–713 (2003). 12963377 10.1016/s0022-2836(03)00929-x

[37] Idan, O. & Hess, H. Origins of activity enhancement in enzyme cascades on scaffolds. *ACS Nano* **7**, 8658–8665 (2013). 24007359 10.1021/nn402823k

[38] Tagliazucchi, M. & Szleifer, I. How does confinement change ligand-receptor binding equilibrium? Protein binding in nanopores and nanochannels. *J. Am. Chem. Soc.* **137**, 12539–12551 (2015). 26368839 10.1021/jacs.5b05032

[39] Rubinovich, L. & Polak, M. The intrinsic role of nanoconfinement in chemical equilibrium: evidence from DNA hybridization. *Nano Lett.* **13**, 2247–2251 (2013). 23600497 10.1021/nl4008198

[40] Kuchler, A., Yoshimoto, M., Luginbuhl, S., Mavelli, F. & Walde, P. Enzymatic reactions in confined environments. *Nat. Nanotechnol.* **11**, 409–420 (2016). 27146955 10.1038/nnano.2016.54

[41] Xiong, Y., Huang, J., Wang, S.-T., Zafar, S. & Gang, O. Local environment affects the activity of enzymes on a 3D molecular scaffold. *ACS Nano* **14**, 14646–14654 (2020). 32880434 10.1021/acsnano.0c03962

[42] Scherf, M. et al. Trapping of protein cargo molecules inside DNA origami nanocages. *Nanoscale* **14**, 18041–18050 (2022). 36445741 10.1039/d2nr05356j

[43] Segawa, Y. et al. Functional development of Src tyrosine kinases during evolution from a unicellular ancestor to multicellular animals. *Proc. Natl Acad. Sci. USA* **103**, 12021–12026 (2006). 16873552 10.1073/pnas.0600021103 PMC1567691

[44] Castro, C. E. et al. A primer to scaffolded DNA origami. *Nat. Methods* **8**, 221–229 (2011). 21358626 10.1038/nmeth.1570

[45] Kucukelbir, A., Sigworth, F. J. & Tagare, H. D. Quantifying the local resolution of cryo-EM density maps. *Nat. Methods* **11**, 63–65 (2014). 24213166 10.1038/nmeth.2727 PMC3903095

[46] Jumper, J. et al. Highly accurate protein structure prediction with AlphaFold. *Nature* **596**, 583–589 (2021). 34265844 10.1038/s41586-021-03819-2 PMC8371605

[47] Pettersen, E. F. et al. UCSF Chimera—a visualization system for exploratory research and analysis. *J. Comput Chem.* **25**, 1605–1612 (2004). 15264254 10.1002/jcc.20084

[48] Mirdita, M. et al. ColabFold: making protein folding accessible to all. *Nat. Methods* **19**, 679–682 (2022). 35637307 10.1038/s41592-022-01488-1 PMC9184281

[49] Zhou, F. F., Xue, Y., Chen, G. L. & Yao, X. GPS: a novel group-based phosphorylation predicting and scoring method. *Biochem. Biophys. Res. Commun.* **325**, 1443–1448 (2004). 15555589 10.1016/j.bbrc.2004.11.001

