# Supplementary material for: A modular DNA origami nanocompartment for engineering a cell-free, protein unfolding and degradation pathway
Source: Nat Nanotechnol. 2024 Jul 29;19(10):1521–31. doi: 10.1038/s41565-024-01738-7 (PMC11486656; doi:10.1038/s41565-024-01738-7)

nature nanotechnology

Supplementary information https://doi.org/10.1038/s41565-024-01738-7

A modular DNA origami nanocompartment for engineering a cell-free, protein unfolding and degradation pathway

In the format provided by the authors and unedited

Table of Content

Supplementary Figures 1-41
Supplementary Tables 1-3
Supplementary References
Supplementary Source Data

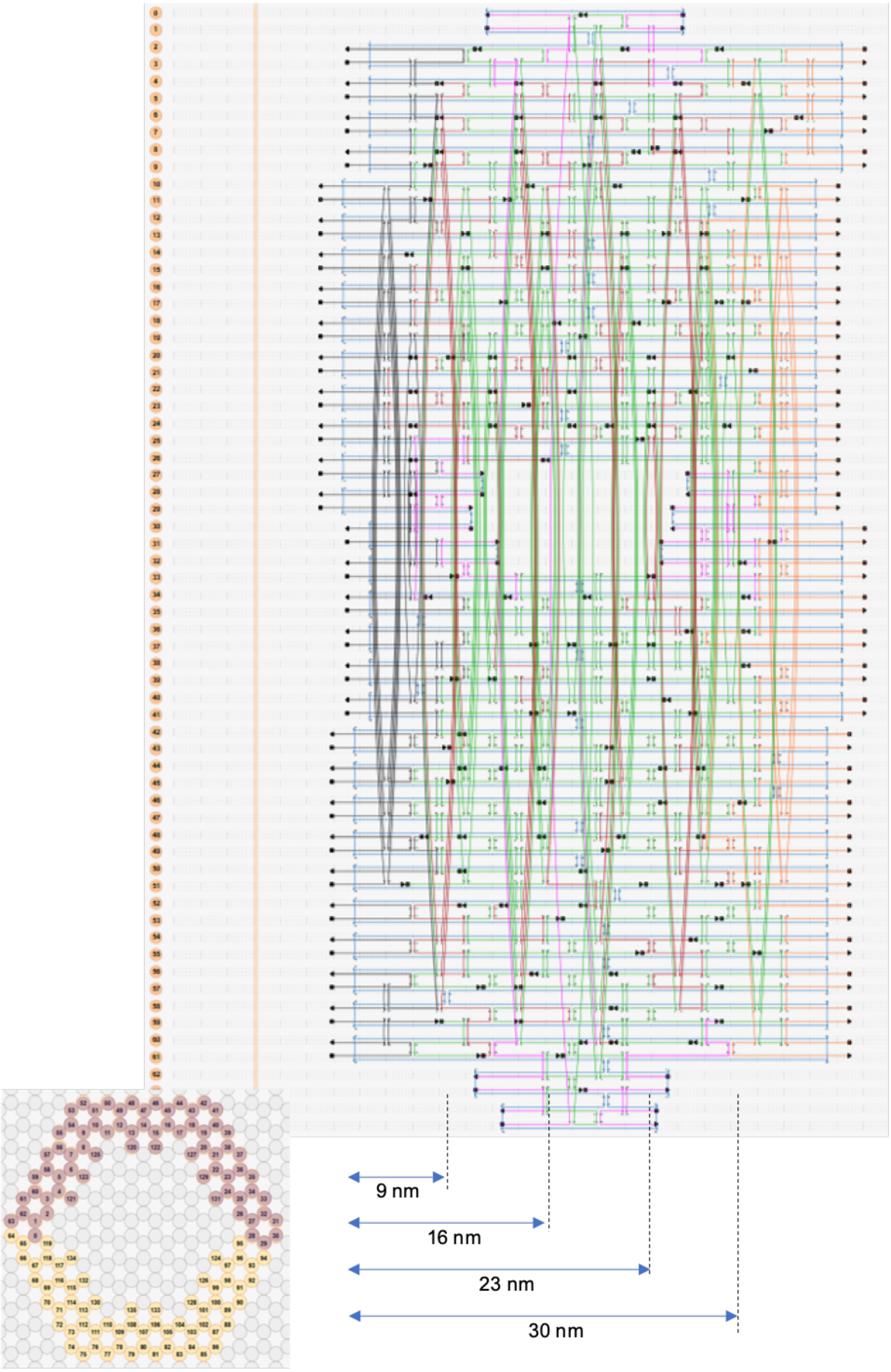

Supplementary Figure 1. Design chart of the *Narcissus* (N) DNA origami compartment. Staple sets are color coded as follows: core of the structure (green), inner pointing protruding arms (red), left edge (black), right edge (orange) and interaction sites for origami-to-origami binding (pink). DNA helices are crosslinked into a hexagonal lattice. The front view of the full NE structure is schematically represented in the lower panel (*Narcissus* half cage is highlighted in pink). The left and right side of the design map correspond, respectively, to the front and back edges of the origami structure. Full list of DNA sequences is provided in Suppl. Data 2.

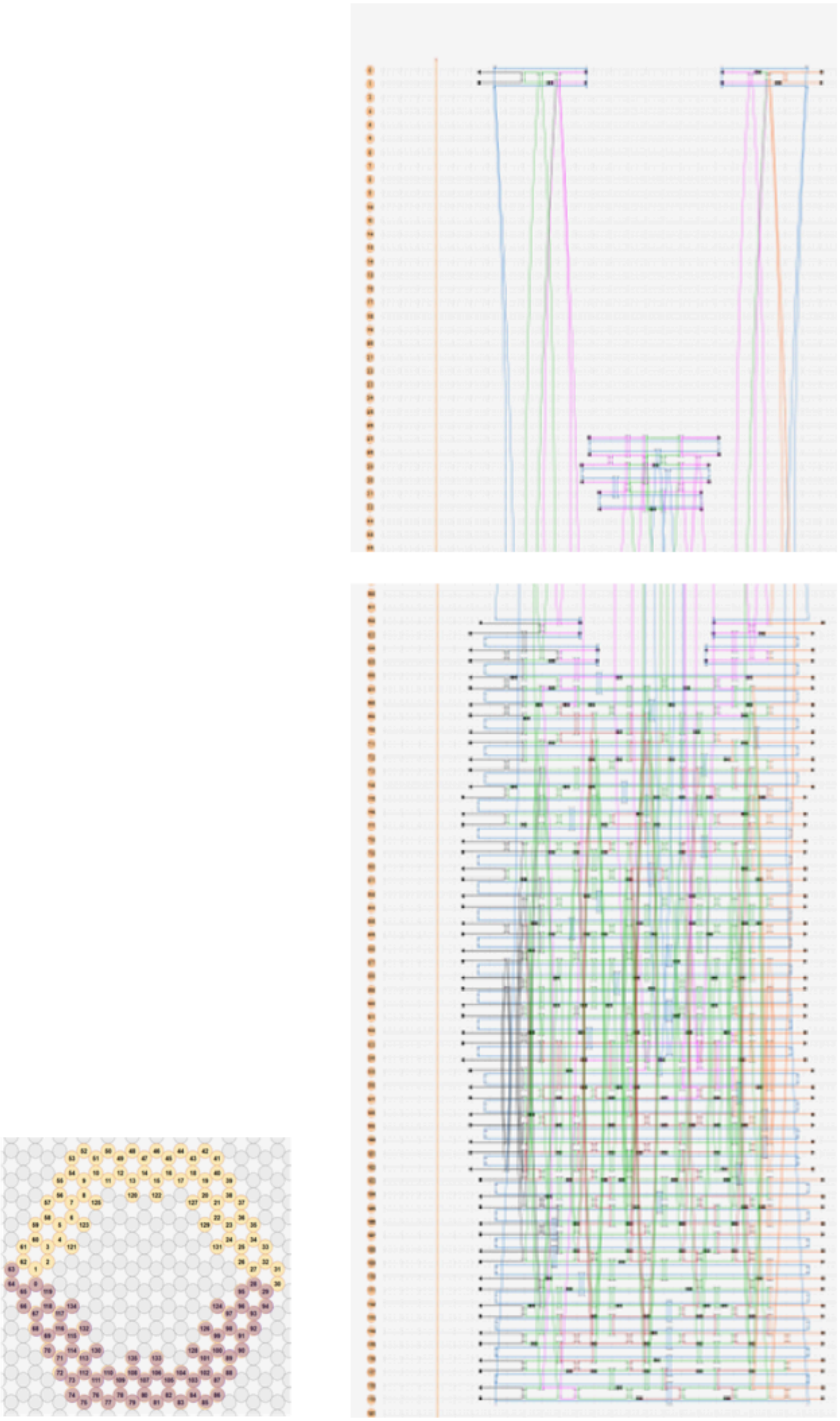

Supplementary Figure 2. Design chart of the *Echo* (E) DNA origami compartment. Staple sets are color coded as follows: core of the structure (green), inner pointing protruding arms (red, same distribution as in N, Suppl. Fig. 1), left edge (black), right edge (orange) and interaction sites for origami-to-origami binding (pink). DNA helices are crosslinked into a hexagonal lattice. The front view of the full NE structure is schematically represented in the lower panel (*Echo* half cage is highlighted in pink). The left and right side of the design map correspond, respectively, to the front and back edges of the origami structure. Full list of DNA sequences is provided in Suppl. Data 2.

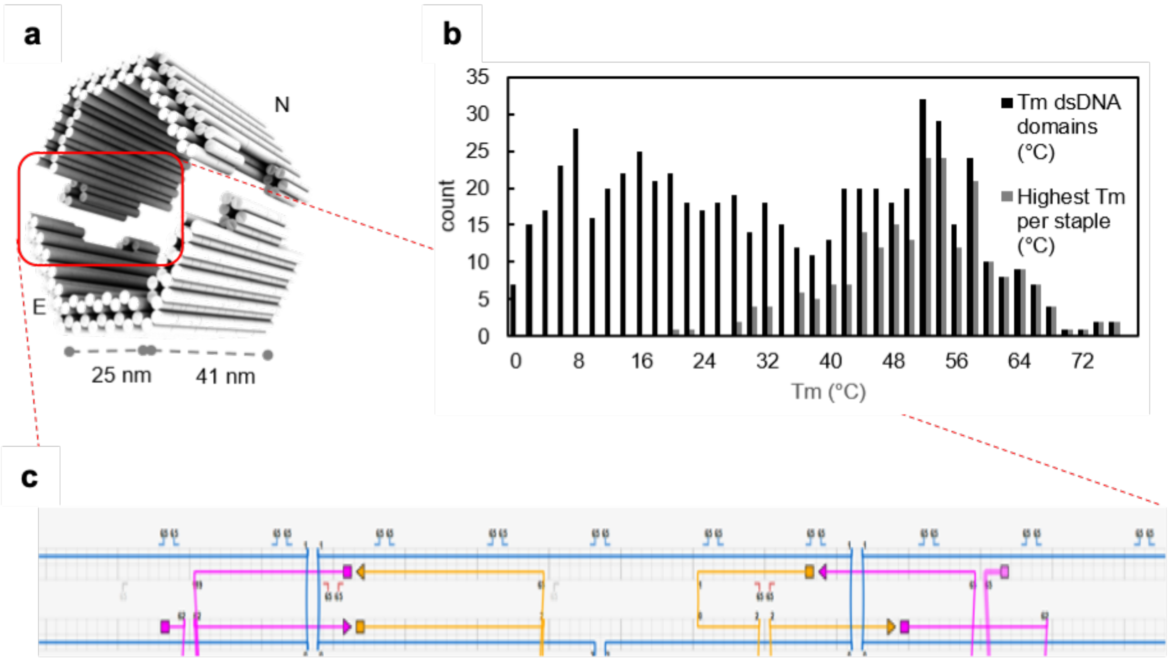

Supplementary Figure 3. Design of the *Nemesis* (NE) full compartment. (a) Three-dimensional model of upper half-prism (N) and lower half-prism (E). (**b**) Identical scaffold sequence permutations were analyzed using the GUI designer_analzer.app[1] and the optimal (fictitious) backbone nick was chosen such to result in a reasonable narrow distribution of melting temperatures for the longest and continuous dsDNA segments. This led to the set of staple strands chosen for the designed scaffold routing. The results obtained for the *Echo* structure are reported here. Hierarchical assembly of N and E into NE was done by hybridization of shape complementary features through 4-nucleotides long stretches. (**c**) An exemplary region is shown for hybridizing staples that connect N (yellow) and E (pink). Full list of DNA sequences is provided in Suppl. Data 2.

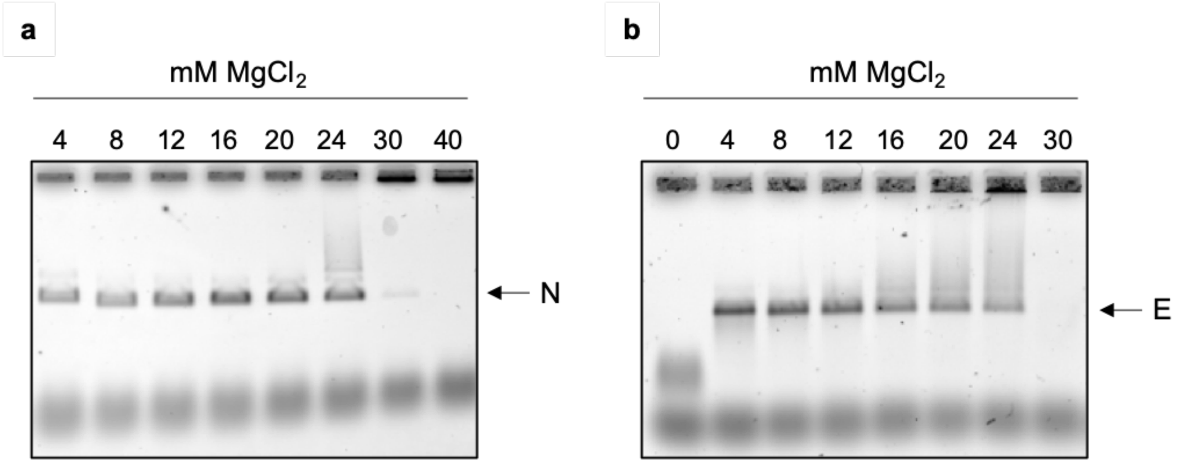

Supplementary Figure 4. AGE analysis of DNA origami assembly at different magnesium ions concentrations. The self-assembly of N (**a**) and E (**b**) was analyzed by AGE at different magnesium ions concentrations in the range between 4 mM and 40 mM. Optimal Mg^{2+} concentration was chosen to be 20 mM for both structures. Gel running conditions: 0.75 % agarose in 1x TBEMg at 80 V, for 2.5 h, at 4 °C. The gel was stained by ethidium bromide. Indicated are the bands associated to the DNA origami structures. Excess staple strands migrate at the bottom of the gel.

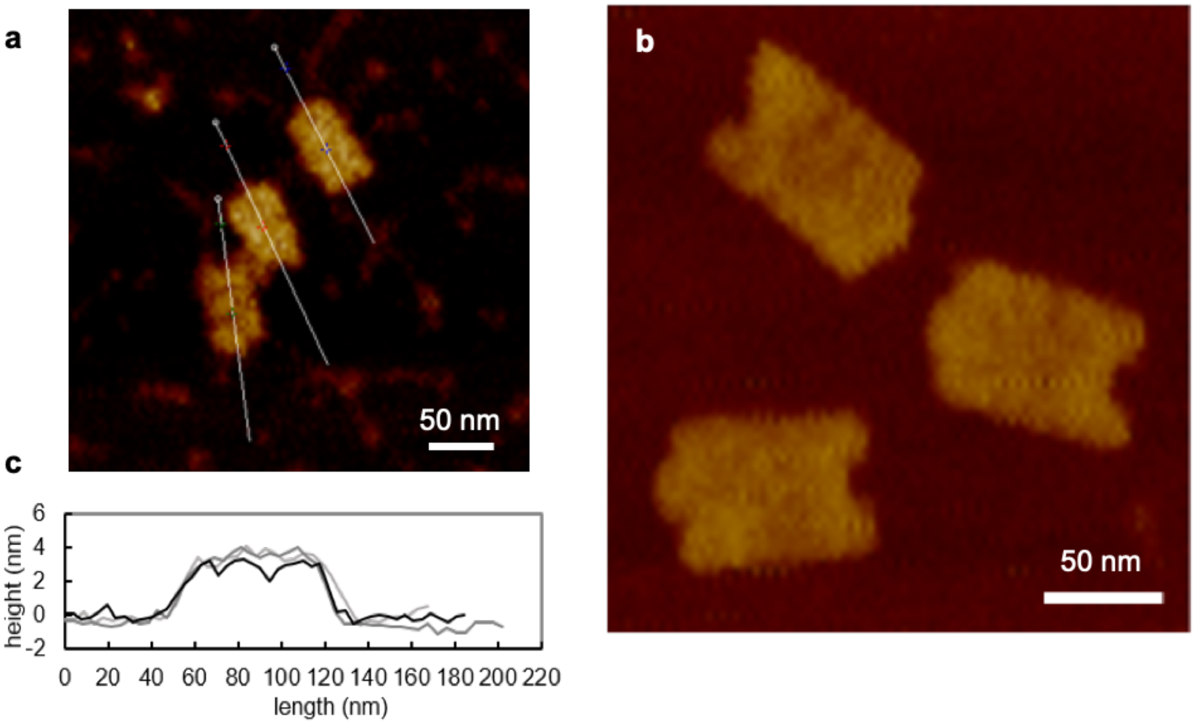

Supplementary Figure 5. AFM characterization of DNA origami half compartments. Successful formation of N (**a**) and E (**b**) structures was confirmed by AFM. Half chambers collapsed on the mica surface, thus showing the complementary shaped features necessary for N to E assembly. Height profiles (**c**) along the depicted lines in (a) revealed a plateau at about 4 nm from the baseline, as expected for a double layered origami. Scale bars are 50 nm.

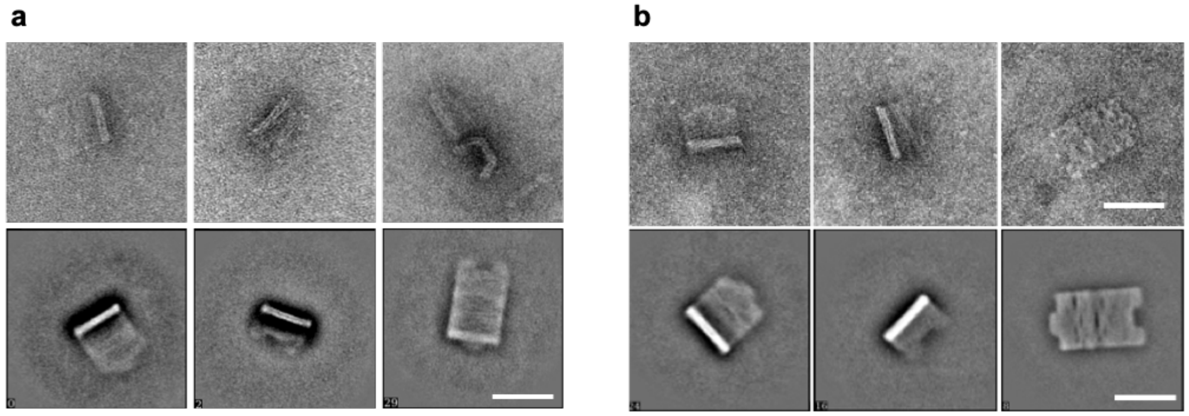

Supplementary Figure 6. TEM characterization of DNA origami half compartments. Successful formation of N (**a**) and E (**b**) structures was confirmed by negative stain TEM. Raw images (top rows) and corresponding class averages (bottom rows) are shown for 4,641 particles averaged in 30 classes (a) and 2,973 particles averaged in 32 classes (b). Scale bars are 50 nm.

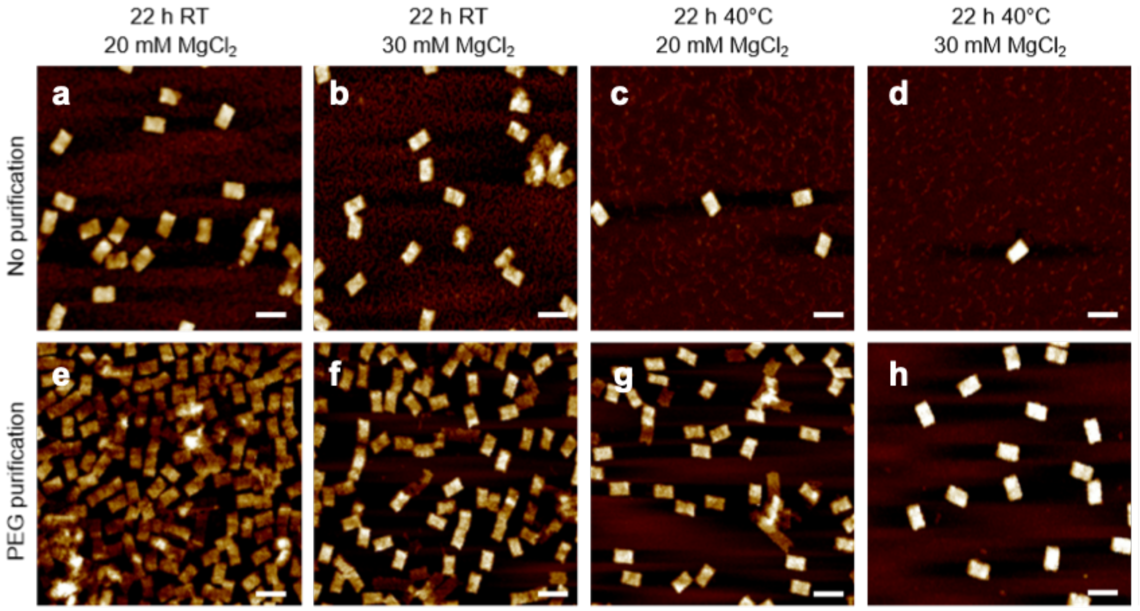

Supplementary Figure 7. AFM characterization of DNA origami full compartments. Hierarchical assembly of N and E into NE was confirmed by AFM imaging. Full structures appeared to be intact and in the expected dimensions, either before (**a-d**) or after (**e-h**) purification by PEG-induced precipitation. The quality of the self-assembly was evaluated at two temperatures, room temperature (RT, ca. 21 °C, a-b, e-f) and 40 °C (c-d, g-h), as well as at two different magnesium ions concentrations, 20 mM (a, c, e, f) and 30 mM (b, d, f, h). Scale bars are 100 nm.

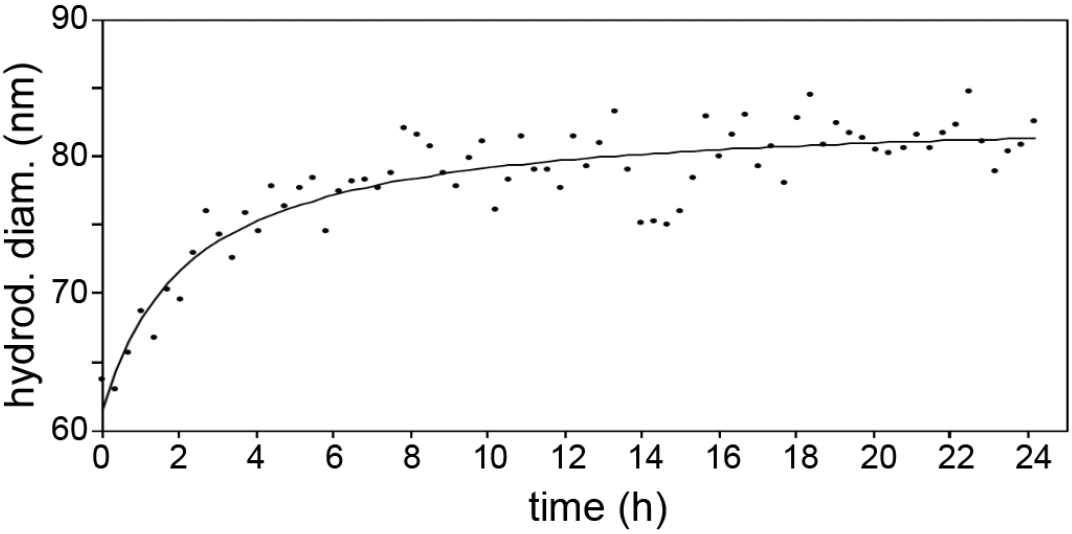

Supplementary Figure 8. Dynamic light scattering (DLS) characterization of NE assembly. Time-course formation of the full compartment (NE), starting from a solution of unpurified N and E in equimolar amount, was monitored by DLS. The assembly terminated in about 10 h at 21 °C. Further optimization of the assembly protocol was achieved varying the concentration of magnesium ions as well as the assembly temperature and the purification state of the starting half cages. The products of the assembly were analyzed by AGE, TEM, and AFM (see Suppl. Fig. 7, 9 and 10). The optimal assembly conditions for formation of the NE cage required equimolar amounts of unpurified N and E incubated at 40 °C for 4 h. Each data point is the mean value of four instrumental measurements, with each measurement taken over one minute.

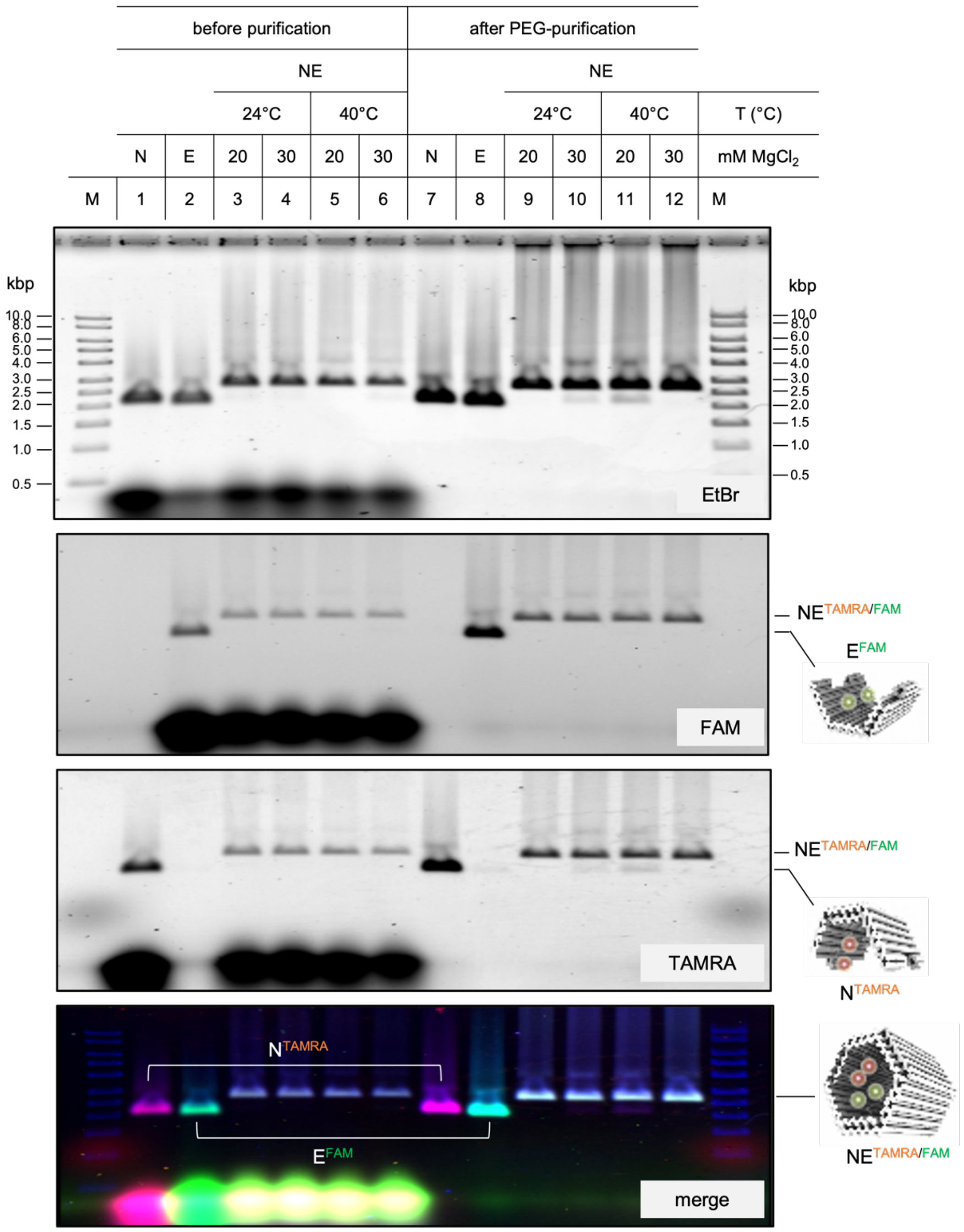

Supplementary Figure 9. AGE characterization of DNA origami compartments. Correct formation of NE was characterized by AGE using fluorescently labelled N and E precursors. Specifically, the inner cavities of the N and E structures were functionalized with three handles each (PA) and these latter were hybridized to complementary strands (cPA), modified at their 3'-terminus, respectively, with TAMRA (red) and FAM (green) dyes. N (lanes 1, 7) and E (lanes 2, 8) cages were incubated at 24 °C (lanes 3, 4 and 9, 10) or 40 °C (lanes 5, 6 and 11, 12) for 3 h in TEMg buffer containing either 20 mM (lanes 3, 5, 9, 11) or 30 mM $MgCl_2$ (lanes 4, 6, 10, 12) and the assembly products were analyzed by AGE, either before (lanes 1-6) or after (lanes 7-12) purification by PEG-induced precipitation. Band shifts at a lower migration rate and superimposition of the TAMRA and FAM fluorescence signals (blue, lanes 3-6 and 9-12) indicated successful achievement of the desired NE structure in all conditions analyzed. Gel running conditions: 0.75 % agarose in 1x TBEMg at 80 V, for 2.5 h, at 4 °C. Lane M contains a 1 kbp DNA ladder (ROTH). DNA origami half chambers N and E migrate between 2.5 kbp and 2.0 kbp. DNA origami full chambers NE migrate at ca. 3.0 kbp. The gel was scanned with a Typhoon FLA9000 (GE healthcare Life Sciences) at different wavelengths and finally stained with ethidium bromide.

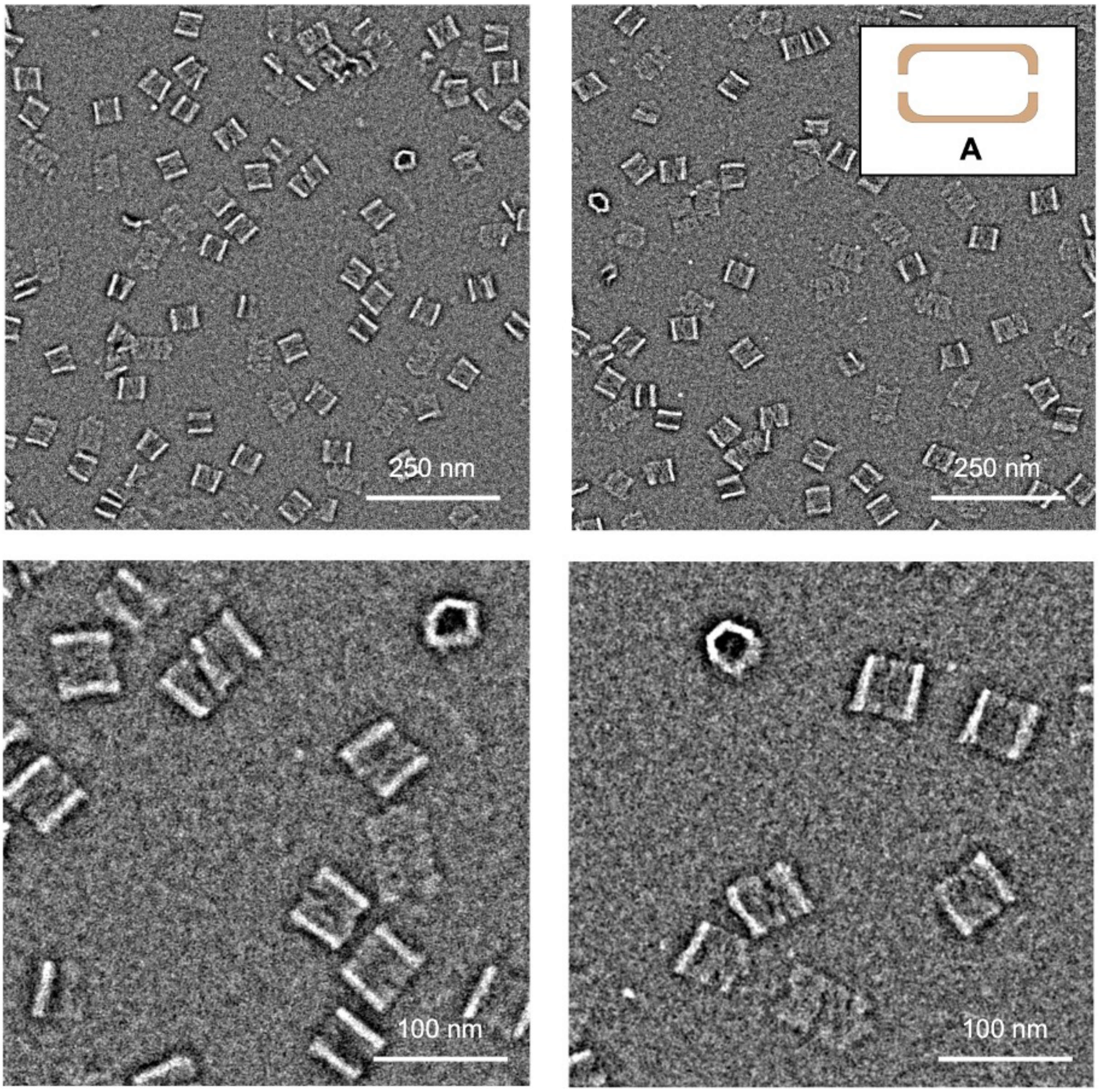

Supplementary Figure 10. TEM characterization of the full DNA origami compartment (also called NE or "A" construct). Successful formation of NE was confirmed by negative stain TEM (98 % yield). Raw images (top rows) and enlarged views (bottom rows) are shown.

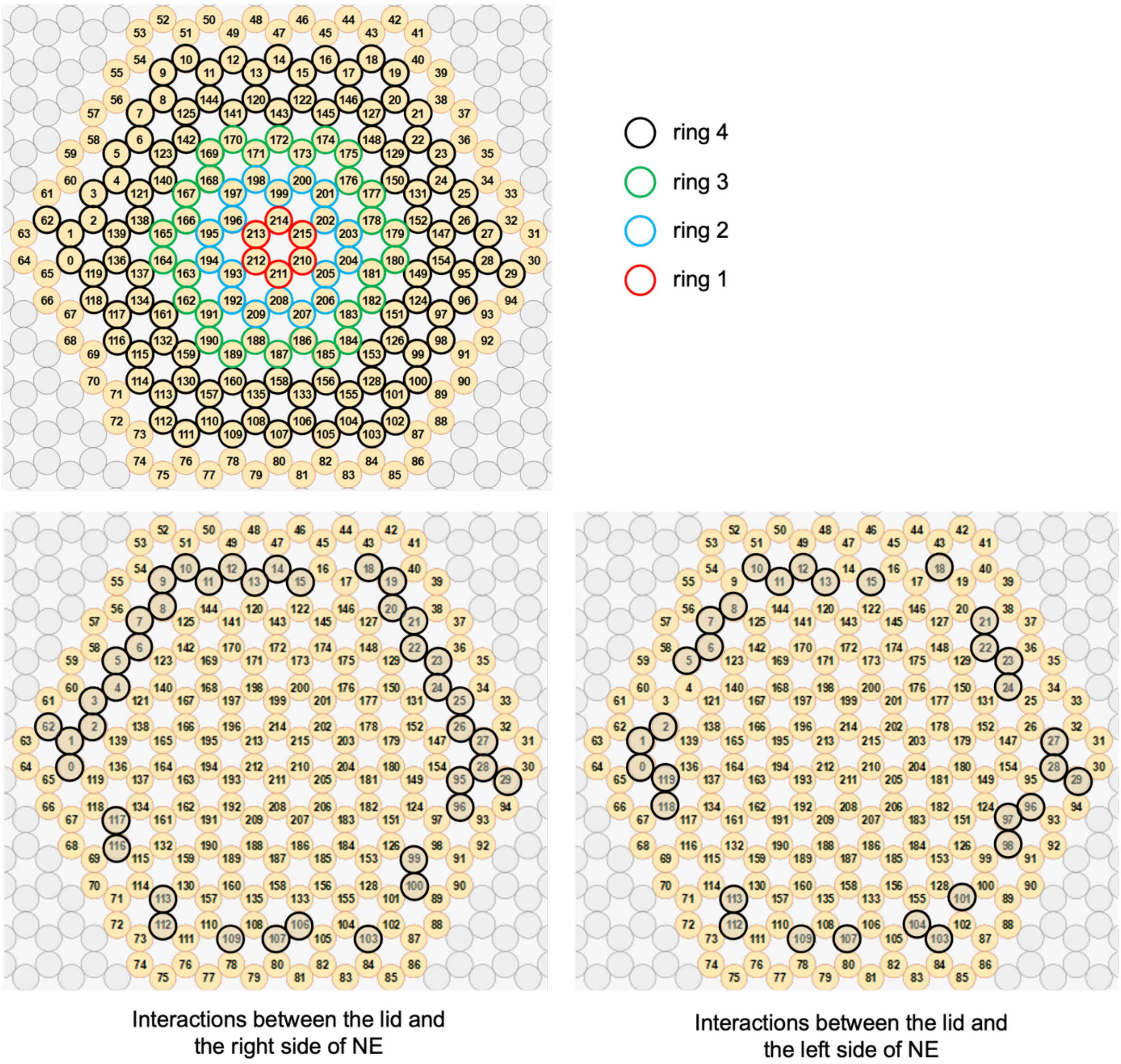

Supplementary Figure 11. Schematic representation of the layout of helices in the DNA origami lid. The lid is composed of helices organized into 4 concentric rings of hexagonal cross-section (from 1 to 4, indicated in different colors). Assembly of a lid composed solely of ring 4 leads to a pore size of ca. 18 nm. Assembly of ring 4 and 3 leads to a pore size of ca. 14 nm. Ring 4 to 2 result in a lid with a pore size of about 9 nm, while full assembly of all rings (from 4 to 1) leads to a lid with an inner pore size equivalent to the spacing between the helices of a honeycomb lattice (ca. 2 nm). Note that all four types of lids insert into the NE through staple crossovers that link the outer helices of layer 4 of the lid (black circles) with the outer layer of helices of the NE compartment. The pattern of interactions is therefore different for the lid bound on the right side or the left side of NE (bottom panels). Moreover, staple crossovers in between the four rings are reduced in number, in order to allow the formation of lids with different apertures. Finally, the edges of the lid which are not used for hybridization were passivated by 6T overhangs. Full list of DNA sequences for assembly of the lid is reported in Suppl. Data 2.

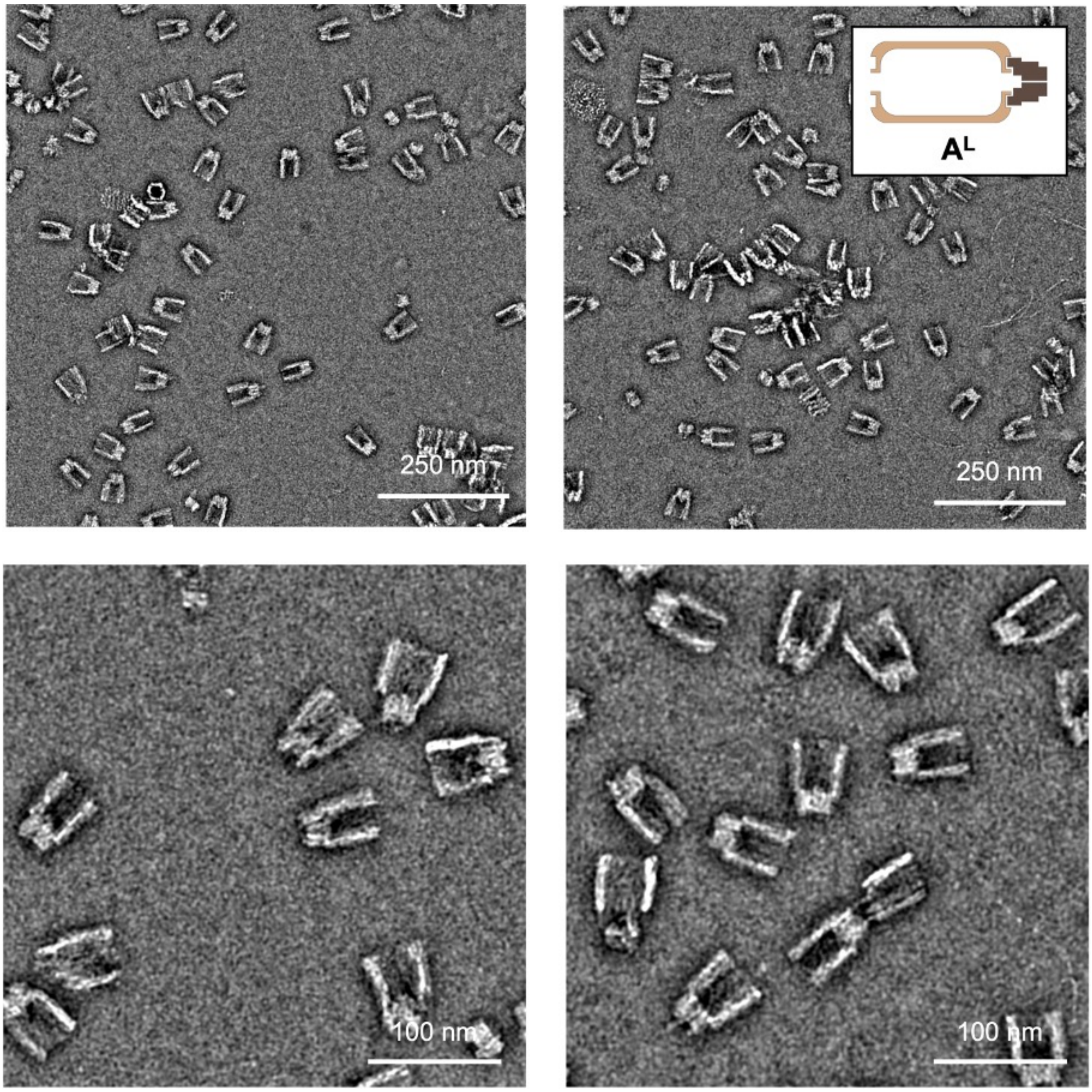

Supplementary Figure 12. TEM characterization of the A^L construct. Successful formation of the A construct with one lid on its right-side edge was confirmed by negative stain TEM (96 % yield). Raw images (top rows) and enlarged views (bottom rows) are shown.

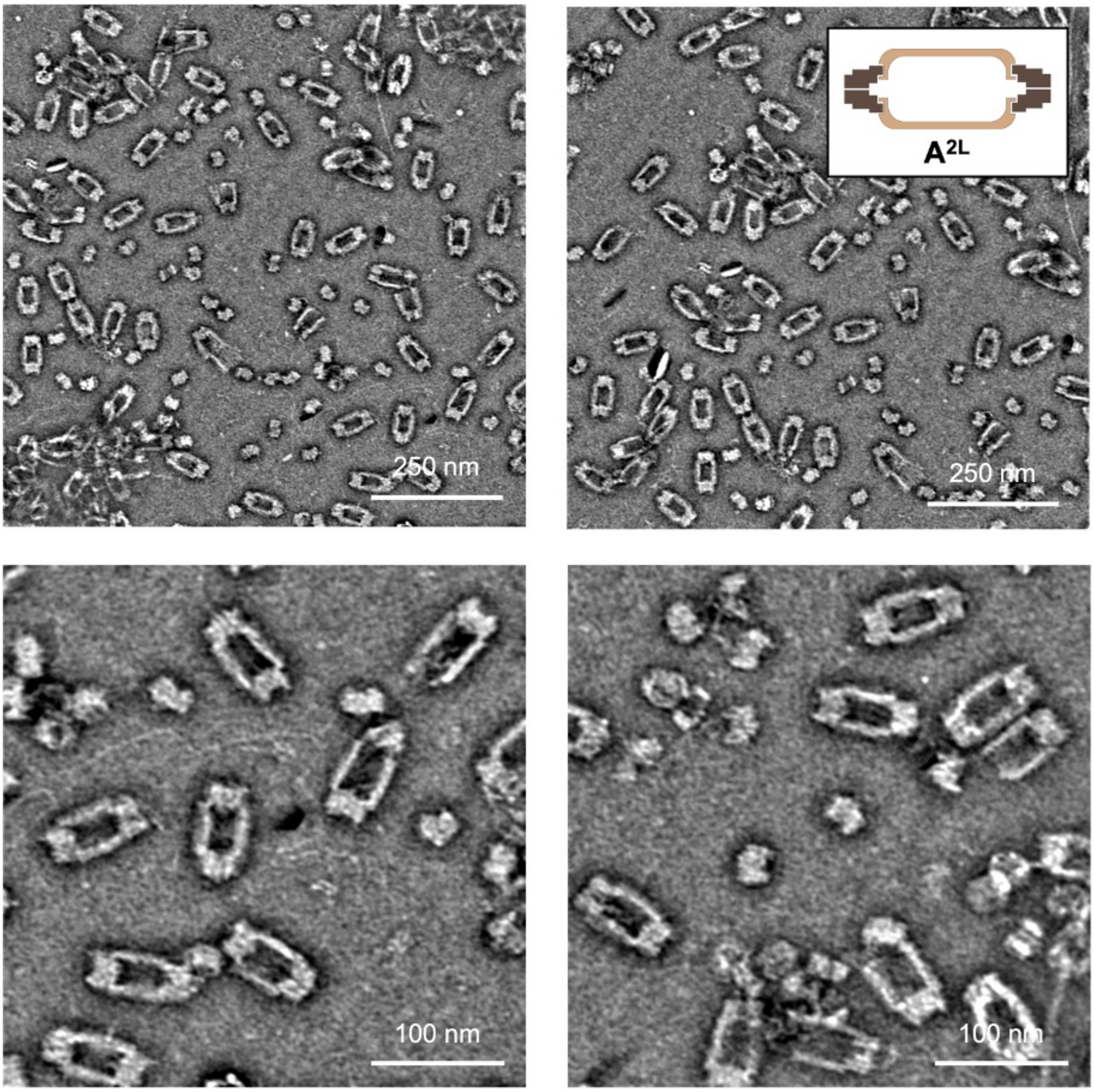

Supplementary Figure 13. TEM characterization of the A^{2L} construct. Successful formation of the A construct with one lid on either side of the compartment was confirmed by negative stain TEM (98 % yield). Raw images (top rows) and enlarged views (bottom rows) are shown.

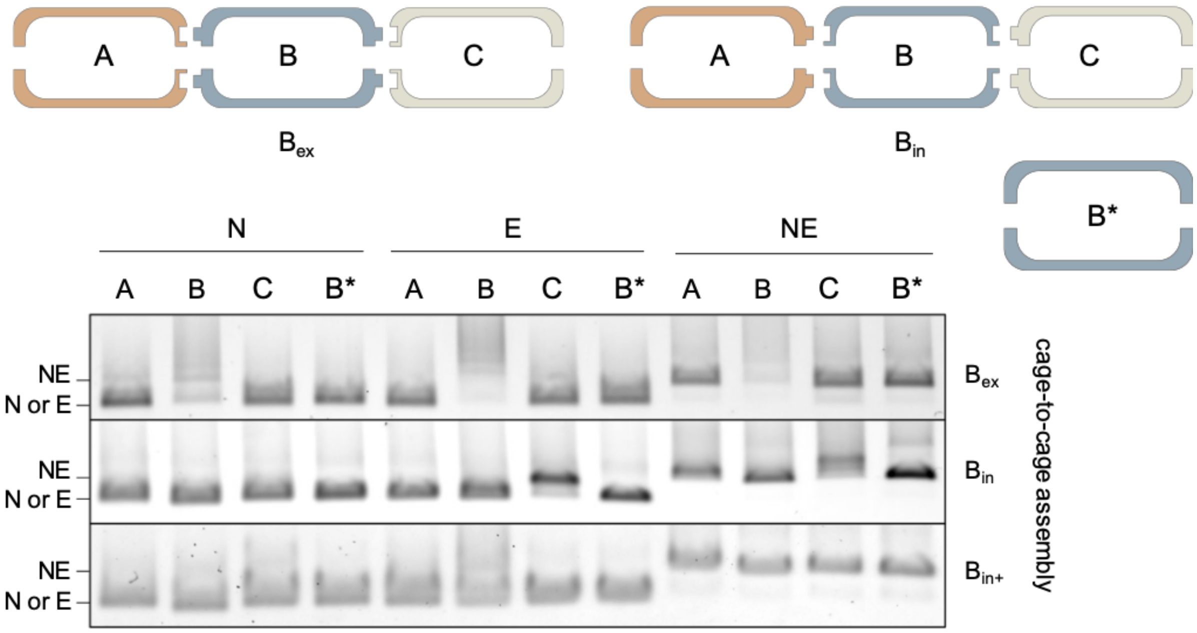

Supplementary Figure 14. AGE characterization of different hierarchical assembly strategies. Two possible design strategies were explored to achieve controlled multimerization of a single NE compartment (upper panel). In the first set, the middle chamber (referred to as B) displays left and right extrusions (B_{ex}) that are complementary in shape and sequence to intrusions, respectively, on the right side of A and left side of C. In a second design, the opposite configuration is adopted, with intrusions on both the left and right side of the central cage (B_{in}). These intrusions are shape and sequence complementary to extrusions on A and C, respectively. A control compartment was constructed with passivated edges that are not suitable for multimerization (B*). The two sets of staple designs were analyzed by AGE (lower panel) and checked for their effect on the formation of both the N and E halves, as well as on the formation of the full NE. The set B_{in} gave generally better results, with only the E half of C being unproperly folded. This set was then further improved, leading to a new B_{in+} design (lower gel panel). Gel running conditions: 0.75 % agarose in 1x TBEMg at 80 V, for 2.5 h, at 4 °C (ethidium bromide staining).

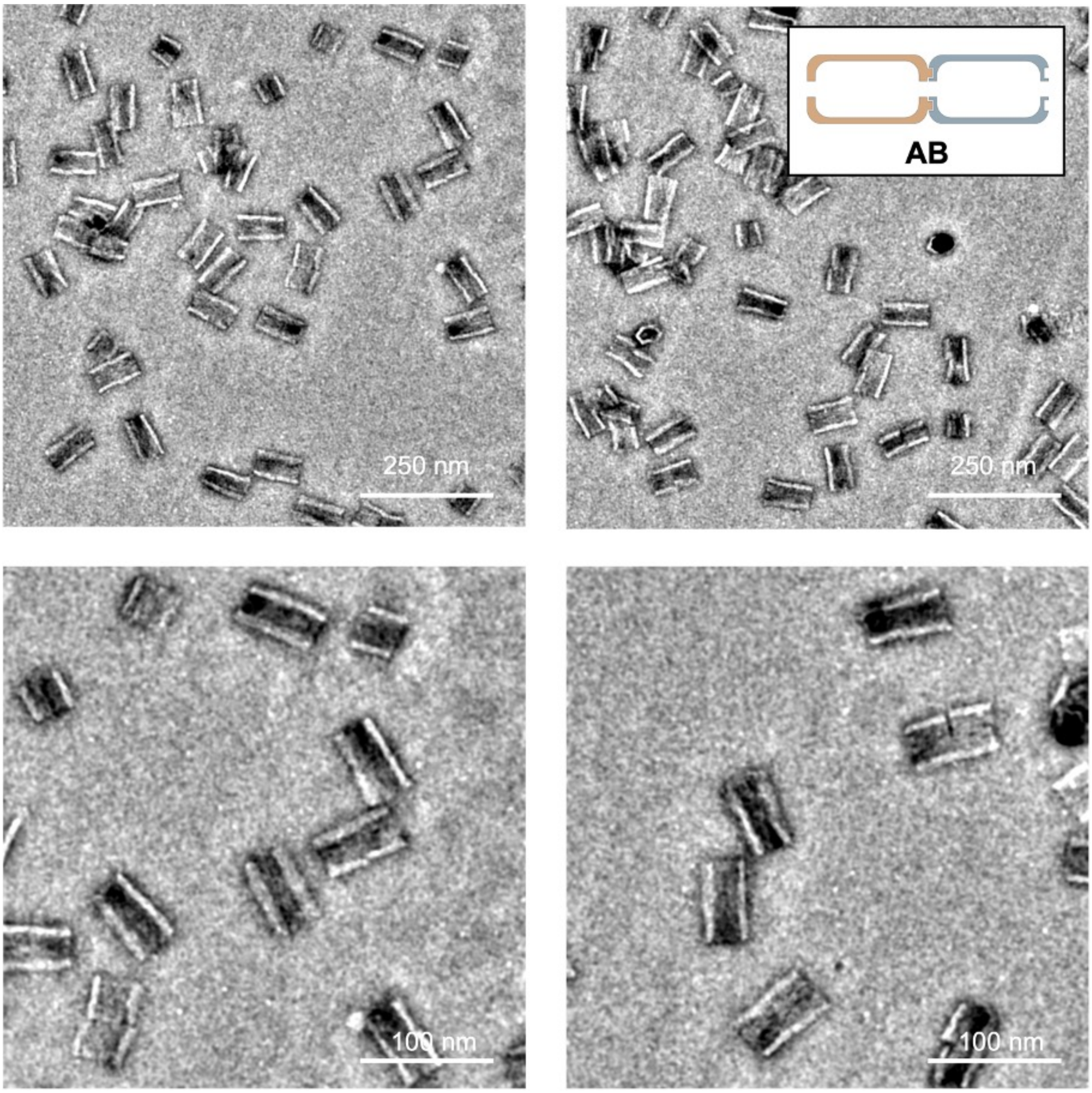

Supplementary Figure 15. TEM characterization of the AB construct. Successful formation of the two-chamber compartment (AB) was confirmed by negative stain TEM. Raw images (top rows) and enlarged views (bottom rows) are shown. The structures formed with a yield of ca. 70 %.

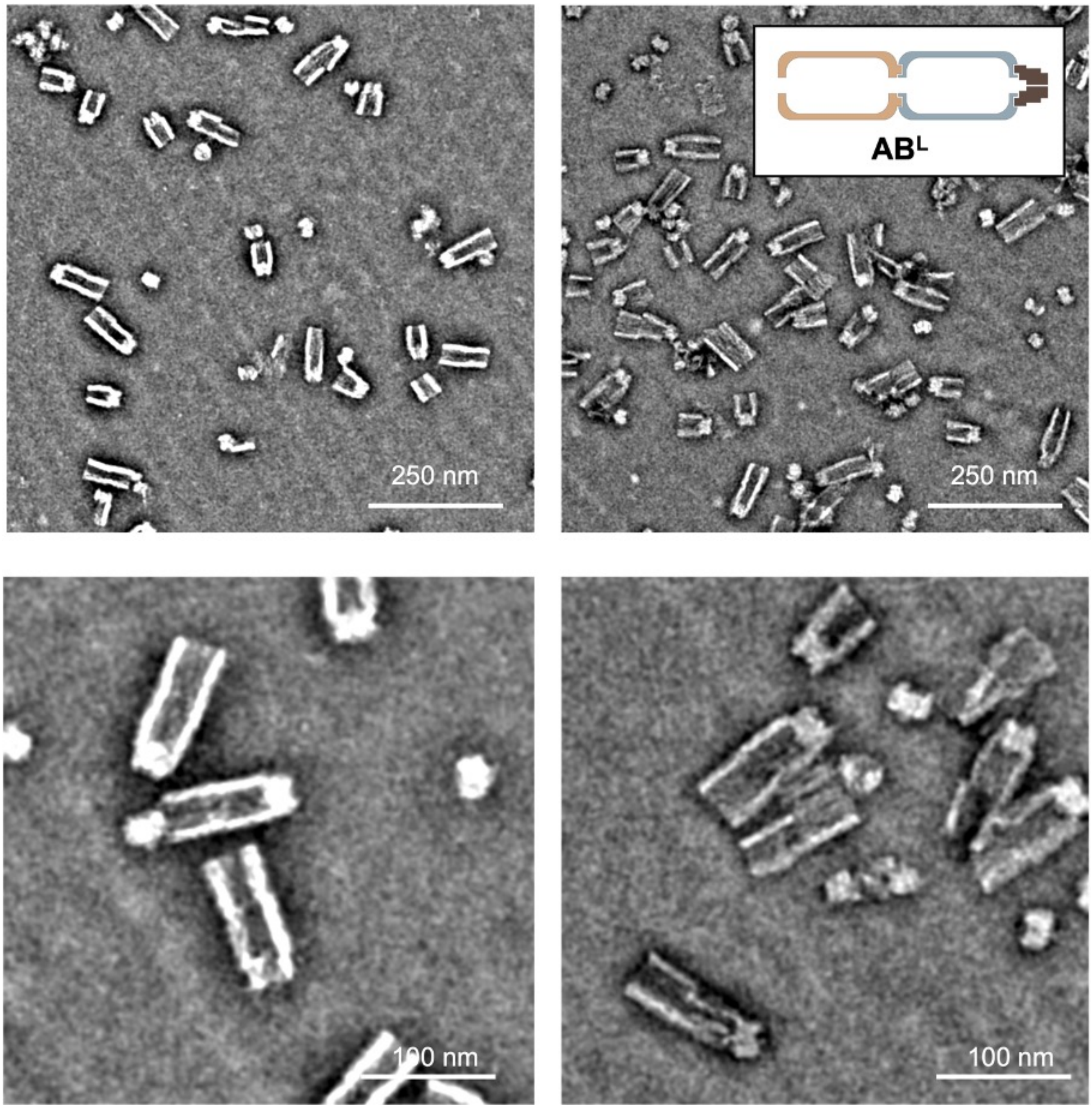

Supplementary Figure 16. TEM characterization of the AB^L construct. Successful formation of the two-chamber compartment bearing one lid on the right-side edge of B was confirmed by negative stain TEM. Raw images (top rows) and enlarged views (bottom rows) are shown. The structures formed with a yield of ca. 38 %.

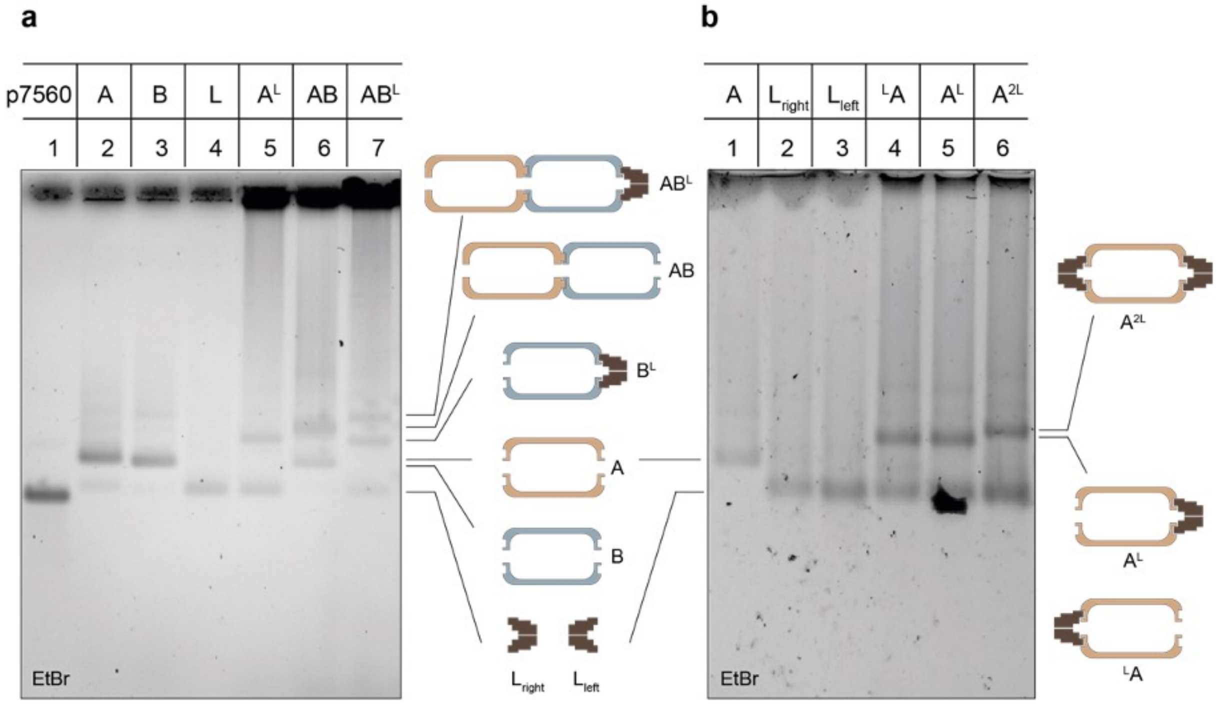

Supplementary Figure 17. AGE characterization of the DNA origami constructs used in this study. One-chamber and two-chambers DNA origami compartments and lids have been assembled as described in the Materials and Methods section and the products of the assembly mixture have been analyzed by gel electrophoresis and TEM. AGE data showed that individual DNA origami constructs (A, B and L) formed as homogenous structures with the expected MW (lanes 2-4 in **a** and lanes 1-3 in **b**). Conversely, multimeric structures did not assemble completely, and unbound monomers were still present in the reaction mixture (lanes 5-7 in **a**; lanes 4-6 in **b**). The excess of unbound lid is not expected to affect the results obtained from the unfolding assays performed on the single cages. However, excess of monomeric chambers may lower the global effect observed in the modular chimera, meaning that a pure solution of two-chambers constructs would result in a higher performance of the system. Gel running conditions: 1% agarose in 1x TBEMg at 80 V, for 2.5 h, at 4 °C (ethidium bromide staining).

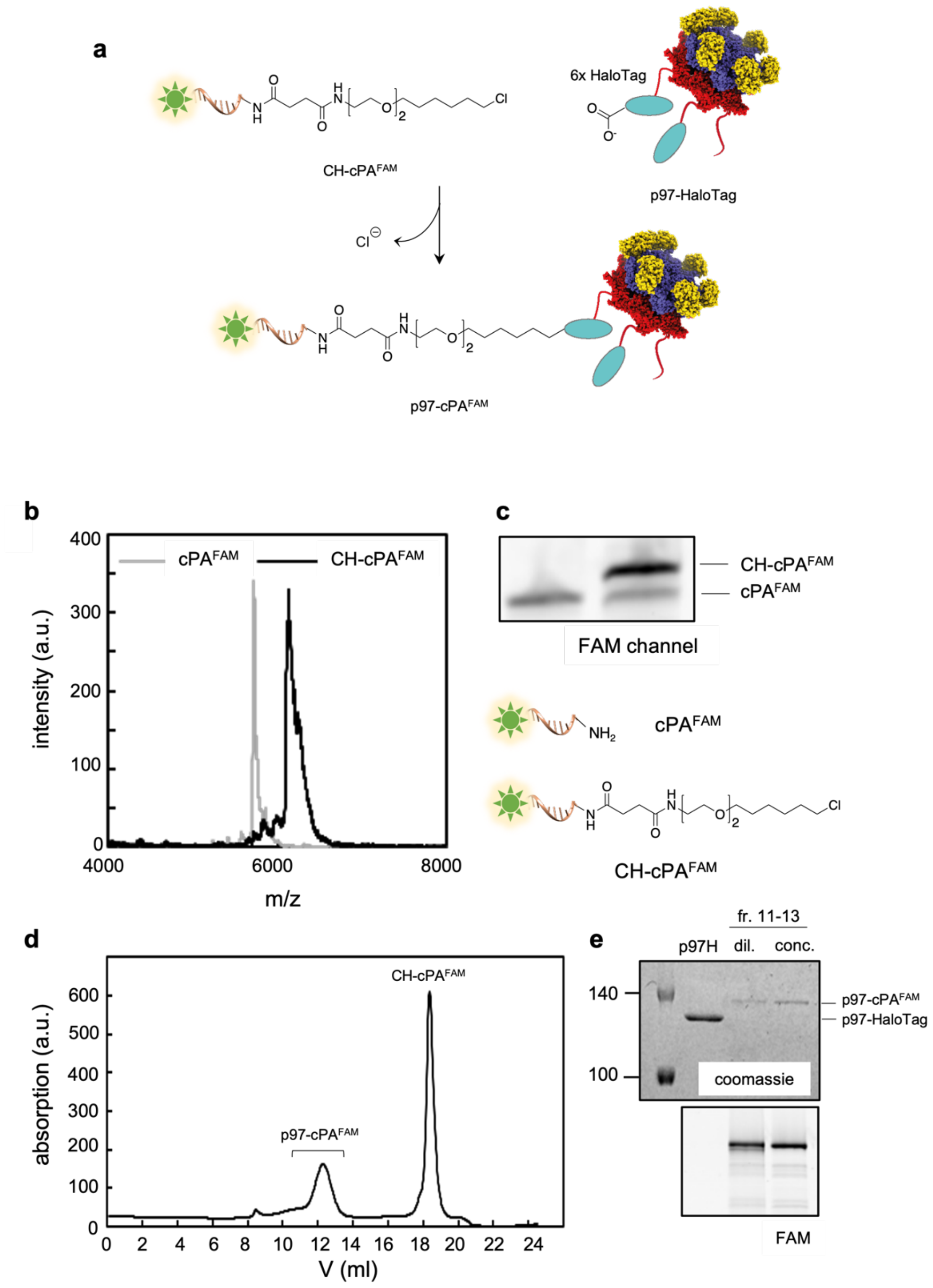

Supplementary Figure 18. Synthesis and purification of the p97-DNA conjugate. (**a**) Schematic representation of the bioconjugation reaction. The C-terminal tails of p97 were genetically fused to HaloTag subunits and covalently linked to chlorohexane-modified DNA handles (CH-cPA^{FAM}), further equipped with a fluorescein label for tracking purposes. (**b**) Overlap of the MALDI spectra for the cPA^{FAM} oligonucleotide and its chlorohexane derivative (CH-cPA^{FAM}). The measured mass difference is 390 Da (expected is 393 Da). The same samples as in (b) were investigated by denaturing PAGE (**c**). Gel scanning upon irradiation in the FAM channel revealed formation of the target product in high yields (ca. 80 %). The reaction mixture containing the CH-cPA^{FAM} and the Halo-tagged p97 was separated by size exclusion chromatography (**d**). The desired p97-DNA conjugate (p97-cPA^{FAM}) eluted between 11 and 13 ml, whereas excess oligonucleotide eluted after 17 ml. Collected fractions were examined by SDS-PAGE (**e**), before and after sample concentration via 100 kDa MWCO ultrafiltration devices and compared with unconjugated p97-HaloTag (expected MW is ca. 130 kDa per monomer; Suppl. Table 1). Yields per p97 monomer were regularly between 70-100 % (calculated from gel band intensity).

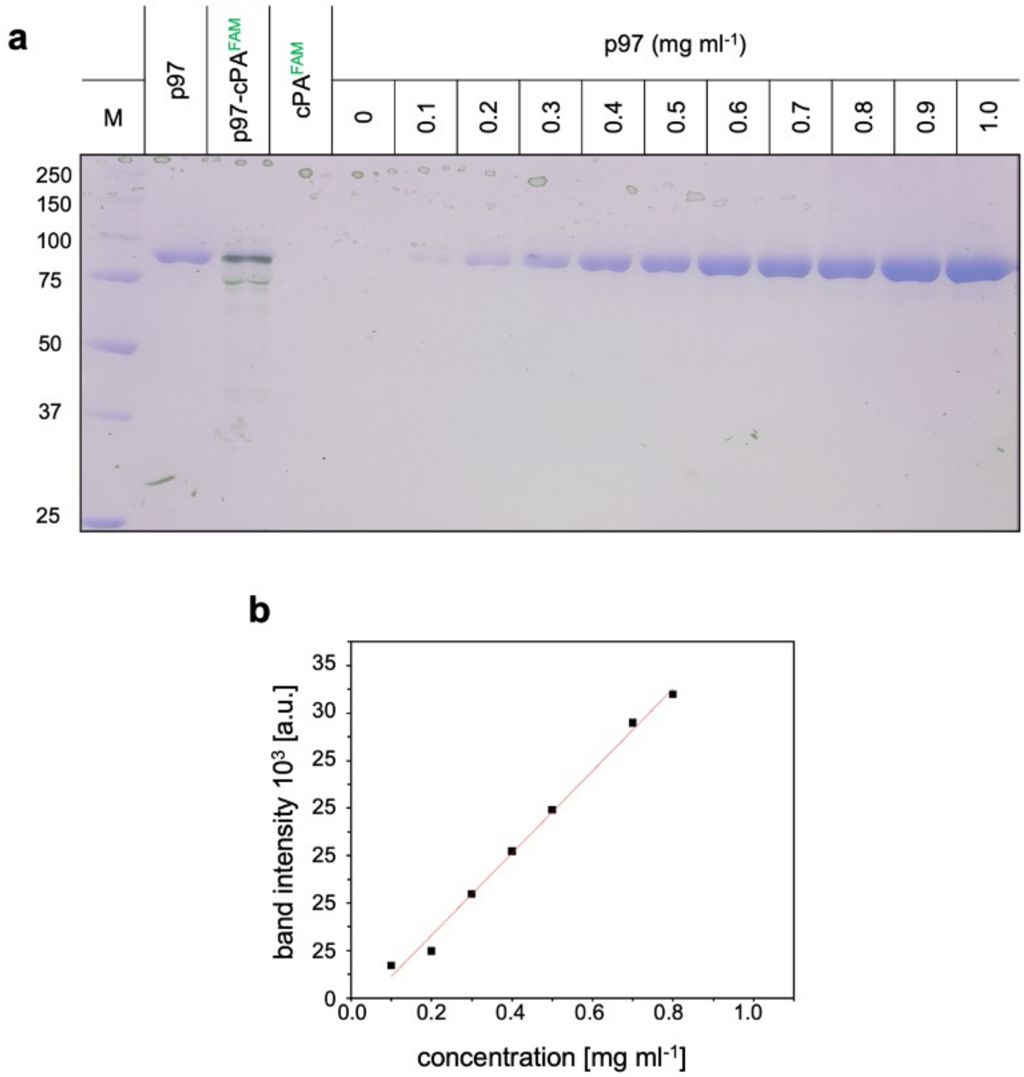

Supplementary Figure 19. Determination of p97-cPA conjugate concentration. (**a**) A solution of the p97-cPA conjugate obtained upon SEC purification (p97-cPA^{FAM}; Suppl. Fig. 18) was loaded in a SDS gel together with a known concentration of unmodified p97 and fluorescently labelled DNA strand (cPA^{FAM}). Various diluted solutions of unmodified p97 were prepared (from 0 to 1.0 mg ml^{-1}) to construct the standard curve (**b**), which allowed to relate the intensity of the p97-cPA band to a known concentration of p97. The intensity of the band corresponding to the p97-cPA conjugate was then used to calculate the concentration of the sample (in this example, p97-cPA^{FAM} concentration is about 0.43 mg ml^{-1}). Gel running conditions: 8 % SDS PAGE at 170 V for 1 h. The gel was first scanned for visualization of FAM emission, then stained with Coomassie and finally scanned again at a Typhoon under illumination with visible light. The gel image is an overlay of the two scans.

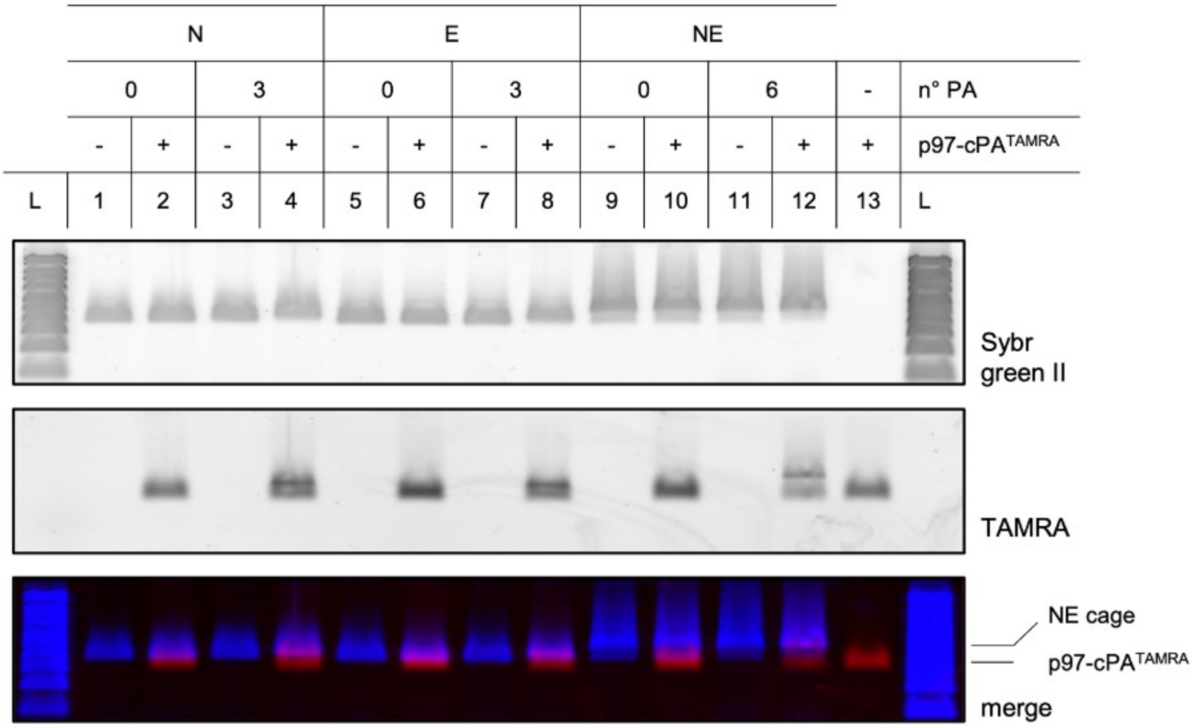

Supplementary Figure 20. AGE characterization of p97 binding to half and full DNA origami compartments. Specific interaction between a p97-cPA^{TAMRA} conjugate and the DNA origami half (N and E) and full (NE) compartments modified in their inner cavity with 0, 3 or a total number of 6 PA handles. Colocalization of the TAMRA (red) and Sybr green II (blue) signals proved successful encapsulation of the p97-cPA^{TAMRA} conjugate within the DNA compartment. Gel running conditions: 0.75 % agarose in 1x TBEMg at 80 V, for 2.5 h, at 4 °C. Lane L contains a 1 kbp DNA ladder. DNA origami half chambers N and E migrate between 2.5 kbp and 2.0 kbp. DNA origami full chambers NE migrate at ca. 3.0 kbp. The gel was scanned with a Typhoon FLA9000 (GE healthcare Life Sciences) at different wavelengths and finally stained with Sybr green II.

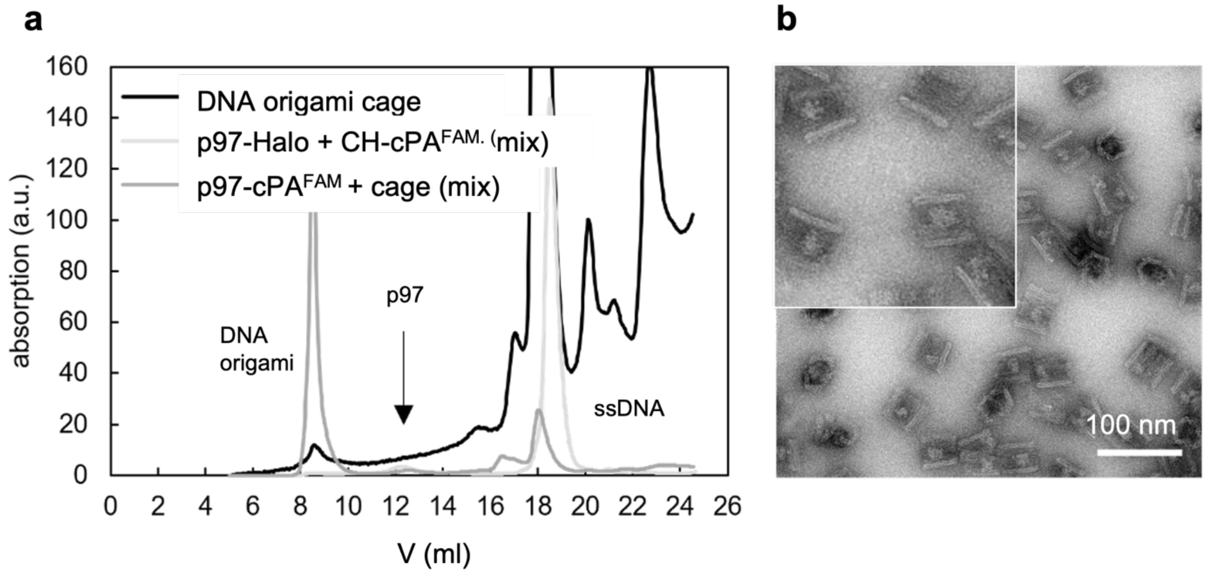

Supplementary Figure 21. Purification of the A(p97) complex by size exclusion chromatography (SEC). (**a**) SEC profiles of a 20 nM solution of assembled DNA origami compartments (black), a reaction mixture containing p97-HaloTag and CH-cPA^{FAM} (light grey) and a mixture of p97-cPA^{FAM} conjugate incubated with an excess of DNA origami compartments (dark grey). DNA origami structures eluted around 9 ml. p97 (with or without the attached oligonucleotide) eluted between 11 and 13 ml, whereas excess DNA oligonucleotides eluted after 13 ml. Absorption values of the three curves were manually amplified for comparative purposes. (**b**) Negative stain TEM imaging of the elution fraction corresponding to the DNA origami containing sample shows that the p97-cPA^{FAM} conjugate is encapsulated within the DNA origami compartment and is mostly near one edge of the structure, as expected by the positions of the complementary PA handles. Scale bar is 100 nm.

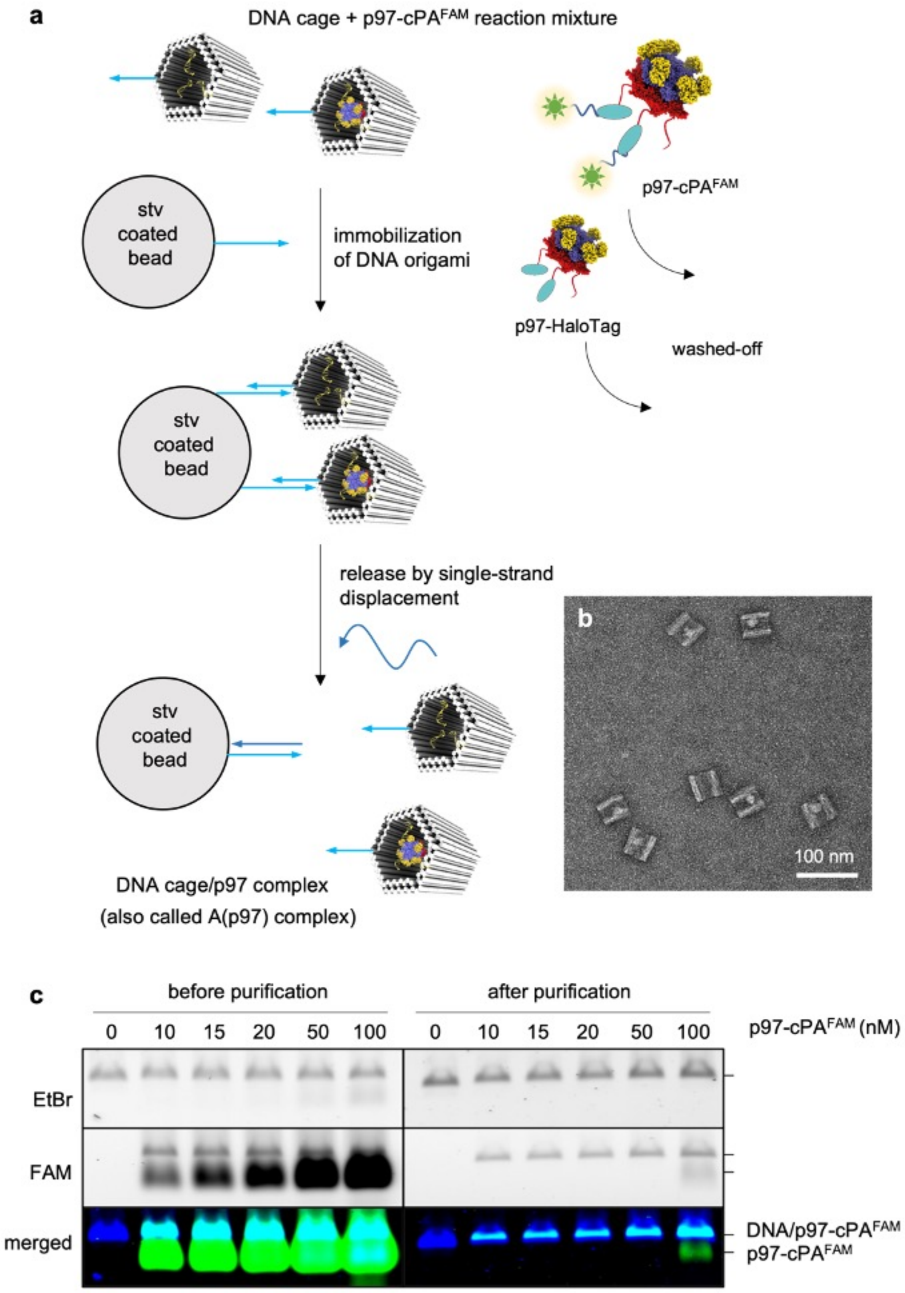

Supplementary Figure 22. Magnetic beads purification of the DNA-encaged p97. (**a**) A mixture containing the NE compartment functionalized in its cavity with 3 or 6 PAs and the p97-cPA^{FAM} conjugate (possibly containing residuals of p97-HaloTag) was purified by streptavidin-coated beads. The NE origami displays a single-stranded extension at one edge of the structure that is partially complementary to a biotin-tagged sequence previously used to coat the beads. In this way, DNA origami (either unloaded or loaded with p97) can be immobilized on the beads and the excess of unbound protein can be washed off. Release of the DNA compartments is done by adding a fully complementary strand (dark blue) according to a single-strand displacement mechanism. Successful purification was confirmed by TEM (**b**, scale bar is 100 nm) as well as by AGE (**c**). Here, 10 nM NE was loaded with increasing concentrations of p97-cPA^{FAM} and analyzed before (left panel) and after (right panel) purification on streptavidin-coated magnetic beads. Comigration of FAM-labelled protein and DNA origami compartments (merged signal in cyan) and simultaneous disappearance of the FAM-signal belonging to the unbound protein (green), confirmed the successful binding and purification of the target complex. Gel running conditions: 0.75 % agarose in 1x TBEMg at 80 V, for 2.5 h, at 4 °C. The gel was scanned with a Typhoon FLA9000 (GE healthcare Life Sciences) at different wavelengths and finally stained with ethidium bromide.

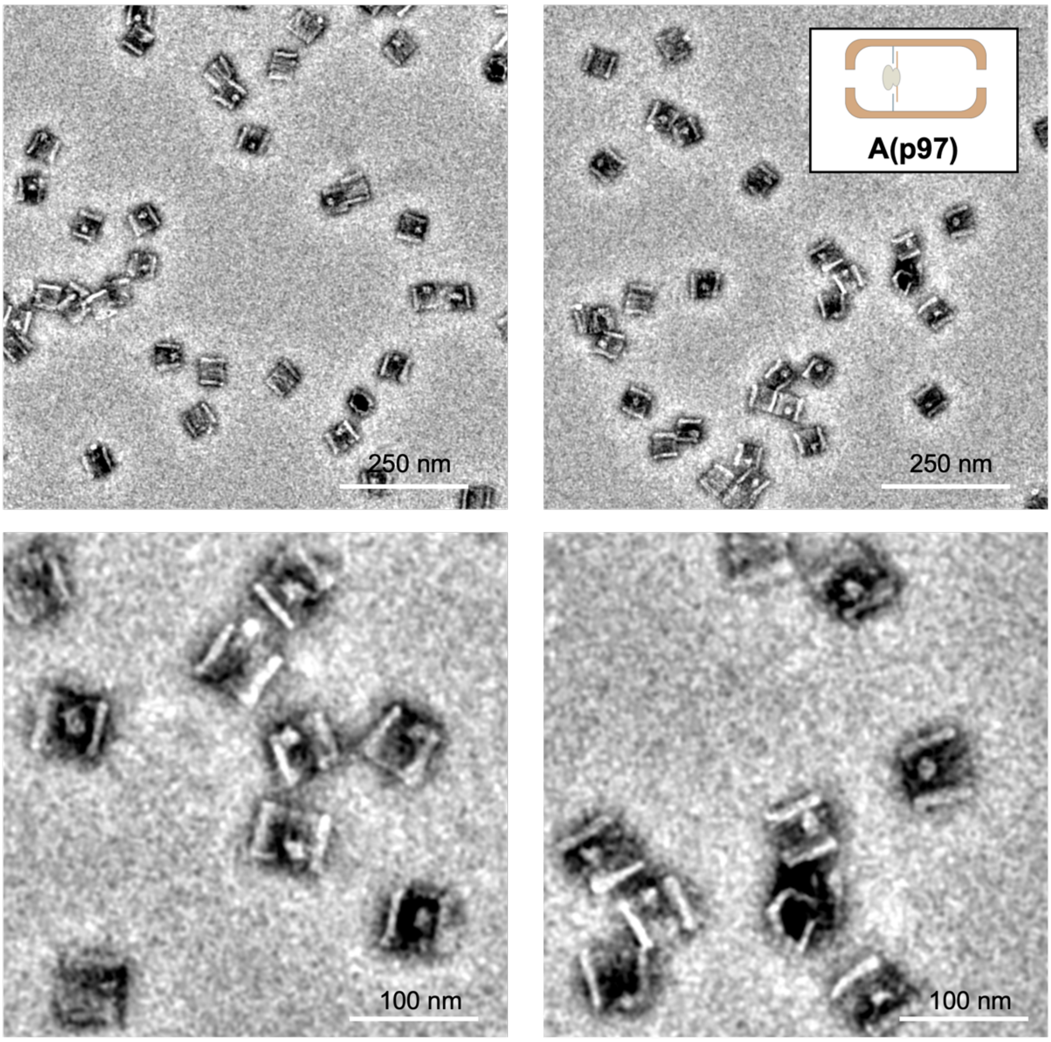

Supplementary Figure 23. TEM characterization of the A(p97) construct. Successful encapsulation of the p97-cPA conjugate into the DNA origami chamber containing 6 PAs led to formation of the A(p97) complex, as confirmed by negative stain TEM. Raw images (top rows) and enlarged views (bottom rows) are shown. Average TEM yield was ca. 75 % (in some cases, up to 95 %).

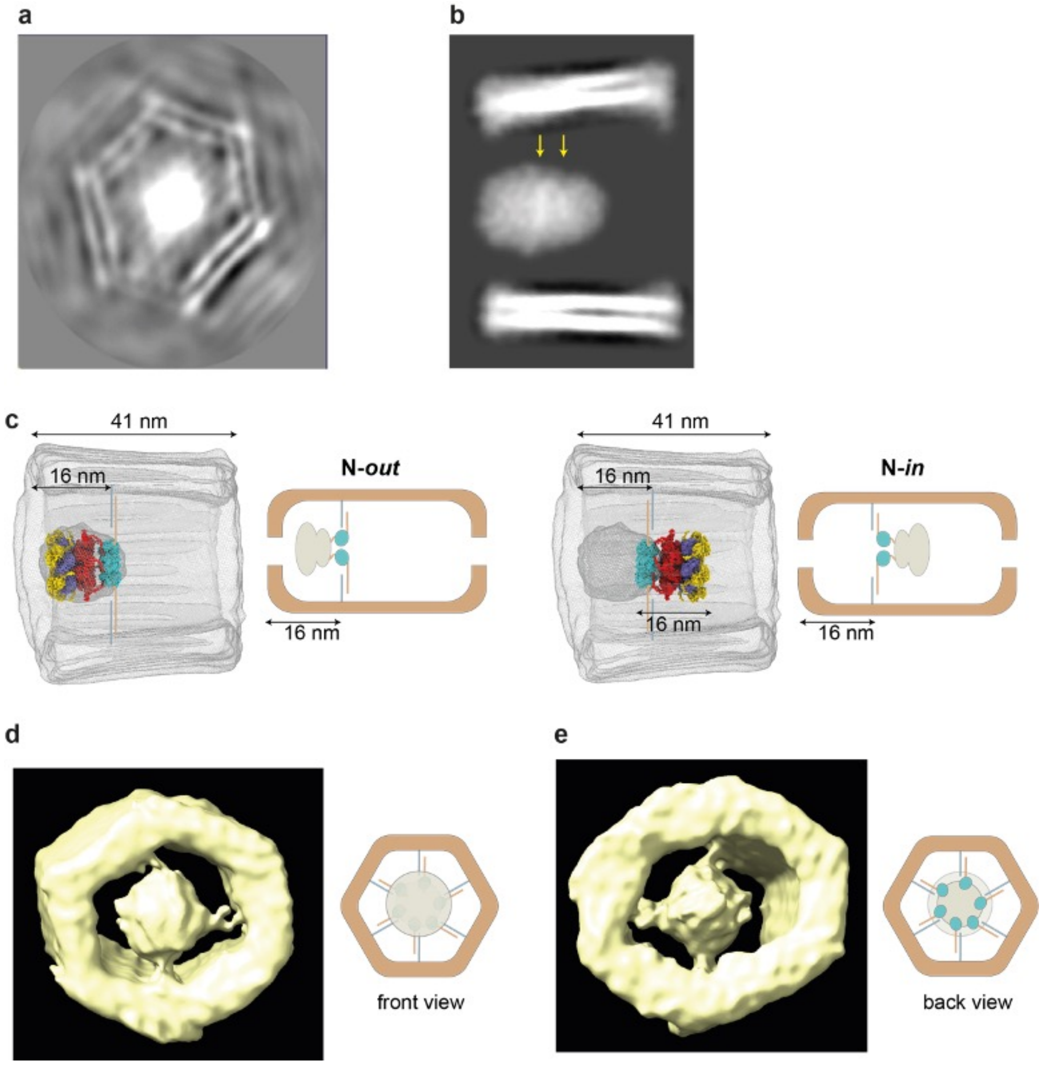

Supplementary Figure 24. Cryo-EM characterization of the A(p97) construct. (**a**) Particle subtraction by protein masking still showed remnants of the DNA cage. Complete subtraction was not possible due to the strong DNA signal and its close proximity to the protein (image collected from ~278 K initial particles). (**b**) The side-view of a 2D visualization of the 3D-refined average map from 27 K particles showed a larger, asymmetric protein density map, clearly indicating that the larger part of the protein points outwards, while the smaller part points inwards. Additionally, higher density features of the protein were observed as two parallel lines of different length, with the longer line nearer to the cage aperture. This strongly implies that the larger subunit (attributed to the N-terminal domains and the D1 ring) is indeed pointing outwards. (**c**) The atomic model of the p97-HaloTag protein in the apo form was obtained by AlphaFold2 and fitted into the density map corresponding to the protein. Fit was possible only with the protein oriented in the N-*out* configuration, with the HaloTag domains aligned to the PAs and the N-terminal domains facing the chamber aperture. The opposite orientation of the protein, with the HaloTag domains still anchored to the PAs and the N-terminal domains faced towards the interior of the DNA chamber, did not fit with the observed density map. This N-*in* configuration would instead lead to a signal distribution located more centrally with respect to the chamber (approximatively between 16 nm and 32 nm from one aperture), with the larger region of the density map farther away from the cage aperture. Note that flipping the N-*in* configuration by 180° around its y-axis does not lead to the N-*out* configuration, as the PAs are not located in the middle of the chamber, rather on one side. Therefore, the two possible orientations are not superimposable and are clearly distinguishable. Finally, the presence of both configurations would lead to an extended electron density map of dumbbell shape. (**d, e**) Snapshots of reconstructed cryo-EM models before noise removal showed the presence of at least three PAs connecting the inner side of the compartment with the protein. Front (d) and back (e) views are shown.

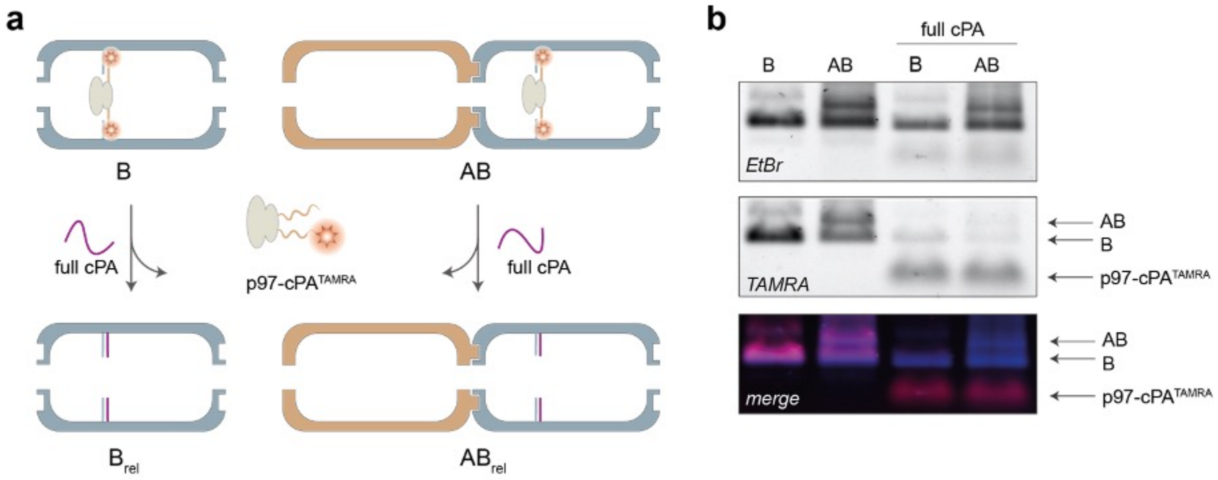

Supplementary Figure 25. Displacement of p97-cPA from the DNA compartment. (**a**) A TAMRA-labelled p97-DNA conjugate (p97-cPA^{TAMRA}) was encapsulated into a B compartment and upon purification an AB construct was prepared using a slight excess of compartment A. Addition of a release strand (full-cPA) which is fully complementary to the PA within the DNA compartment led to displacement of the protein from the compartment and formation of a full duplex on the inner DNA origami walls (B_{rel} or AB_{rel}). (**b**) Both complexes were analyzed by AGE before and after addition of full-cPA, which sets the protein free in solution (see separation of the red and blue colored bands in the last two lanes). The yield of p97 encapsulation estimated by TEM imaging was, for this experiment, about 95 % for B and AB and dropped to about 17 % upon addition of the release strand (full-cPA). Thus, the displacement of p97 upon addition of full-cPA occurred with a yield of ca. 83 %.

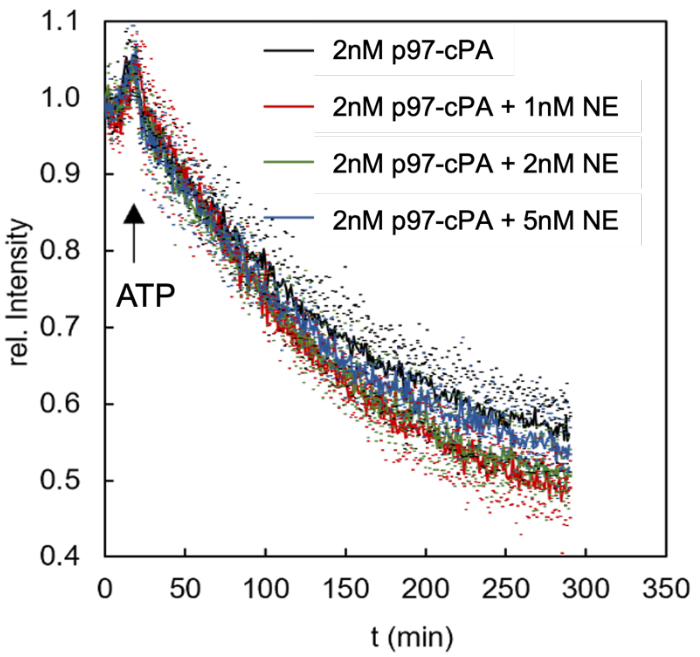

Supplementary Figure 26. Substrate unfolding activity of p97 in presence of unbound DNA origami compartments. Upon photoactivation, the fluorescence emission red signal of the $I3^{mEos}$ substrate was monitored over time in presence of 2 nM p97-cPA conjugate and various concentrations (1 nM to 5 nM) of a DNA origami compartment devoid of inner PA handles and therefore uncapable to bind to the protein. The data showed no significant change in the unfolding rate of p97 and suggest that encapsulation of the protein within the DNA origami chamber is responsible for the increased rate observed in the A(p97) complexes. Data points indicate n = 3 technical replicates for each sample.

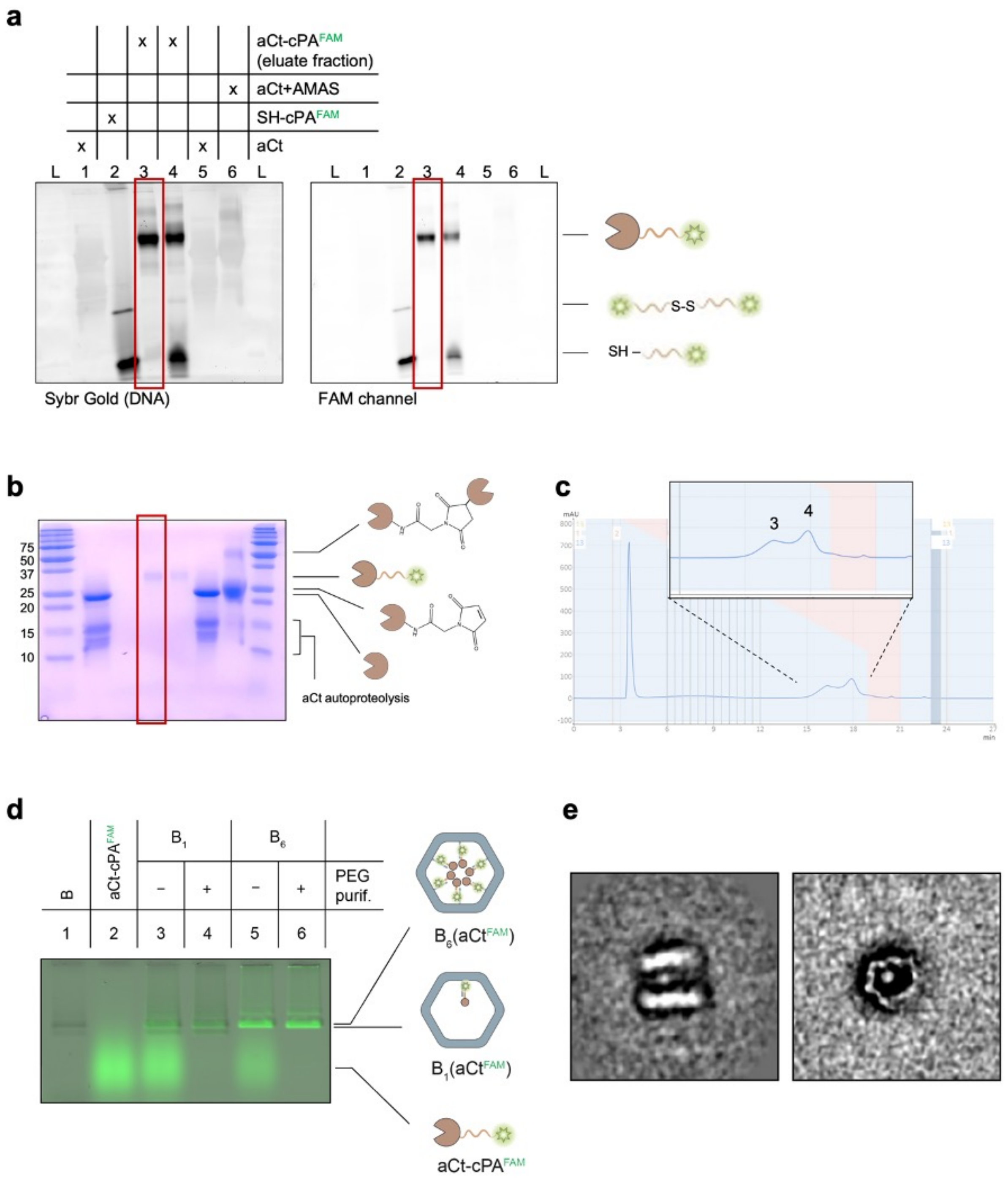

Supplementary Figure 27. Synthesis, purification and characterization of aCt-cPA conjugate and its DNA-encaged form. (**a** and **b**) alpha-Chymotrypsin was modified at lysine residues with a FAM-labelled DNA strand (cPA^{FAM}) using standard chemical conjugation procedures, as described in the Methods section of the main manuscript. The resulting aCt-cPA^{FAM} conjugate was obtained in a 1:1 (protein:DNA) form and successfully purified by ion-exchange chromatography (**c**). Expected MW of aCt-cPA is ca. 30 kDa (see lane 3 in a and b, which is visible upon DNA staining, FAM illumination and Coomassie staining). Gel running conditions:15 % SDS PAGE in 1x SDS running buffer at 180 V for 1 h. (**d**) The resulting aCt-cPA conjugate was immobilized within the cavity of a DNA compartment, bearing either 1 or 6 PAs (B_1 and B_6). The excess of unbound protein was successfully removed by PEG-induced precipitation of the DNA origami. Gel scanned under FAM illumination and after ethidium bromide staining (merged image is shown). (**e**) The final complex was characterized by negative stain TEM; however, the resolution was too low for clear visualization of the protein.

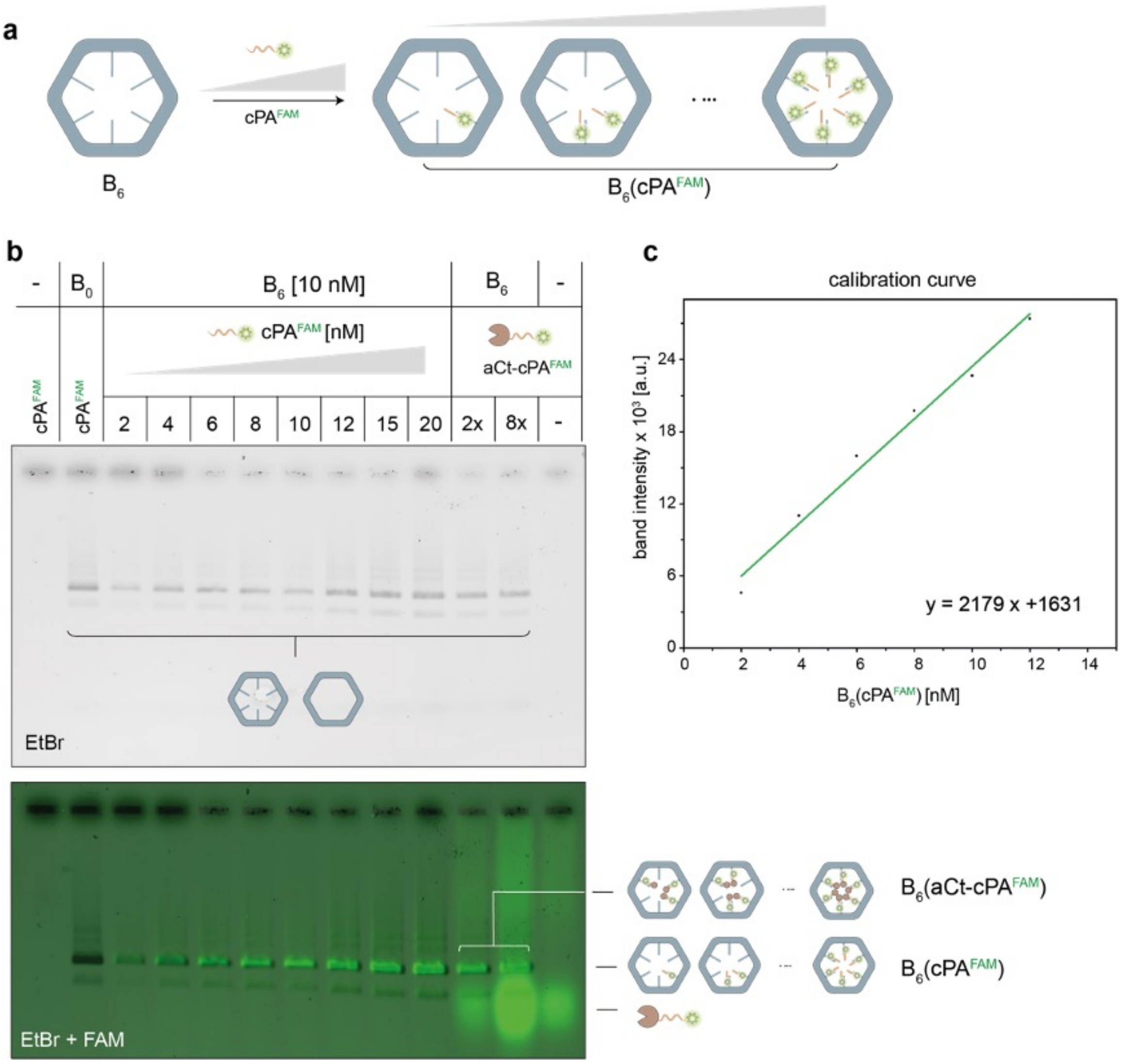

Supplementary Figure 28. AGE-based determination of aCt loading efficiency (method I). (**a**) The fluorescent signal monitored and measured in this method is given by hybridization of the cPA^{FAM} strand to one or more of the 6 complementary PAs displayed in the inner cavity of a pre-assembled DNA origami compartment (B_6). A calibration curve is generated using different concentrations of cPA^{FAM} (from 2 nM to 20 nM) added to a 10 nM solution of B_6 and measuring the intensity of the fluorescently labelled origami band after gel illumination with the appropriate exc/em filters for fluoresceine detection (**b**). In a different experiment, the aCt-cPA^{FAM} conjugate is added in 2-fold or 8-fold to a 10 nM solution of B_6. The intensity of the fluorescently labelled origami band is then used to calculate the concentration of the aCt-DNA conjugate loaded within the DNA origami cage (**c**). This procedure has been repeated for every newly synthesized aCt-cPA^{FAM} conjugate, creating a new calibration curve for each gel experiment. In average, 2-fold excess in aCt-cPA conjugate allowed to reach a loading efficiency of ca. 65 %. A higher excess of conjugate did not improve the encapsulation yield and made the removal of the unbound protein more difficult. This ensemble method provides a roughly quantitative estimation of the encapsulation yield and relies on the following assumptions: (i) every DNA origami compartment displays 6PAs, (ii) all PAs are equally accessible for hybridization and (iii) the intensity of the FAM signal is proportional to the amount of FAM molecules present within the cage *upon hybridization* and no other fluorescent phenomena are considered that may affect the observed fluorescent signal (e.g DNA-induced quenching or photobleaching). Gel running conditions: 1 % agarose in 1x TBEMg at 80 V, for 2 h, at 4 °C. The gel was scanned with a Typhoon FLA9000 (GE healthcare Life Sciences) after FAM illumination and finally stained with ethidium bromide.

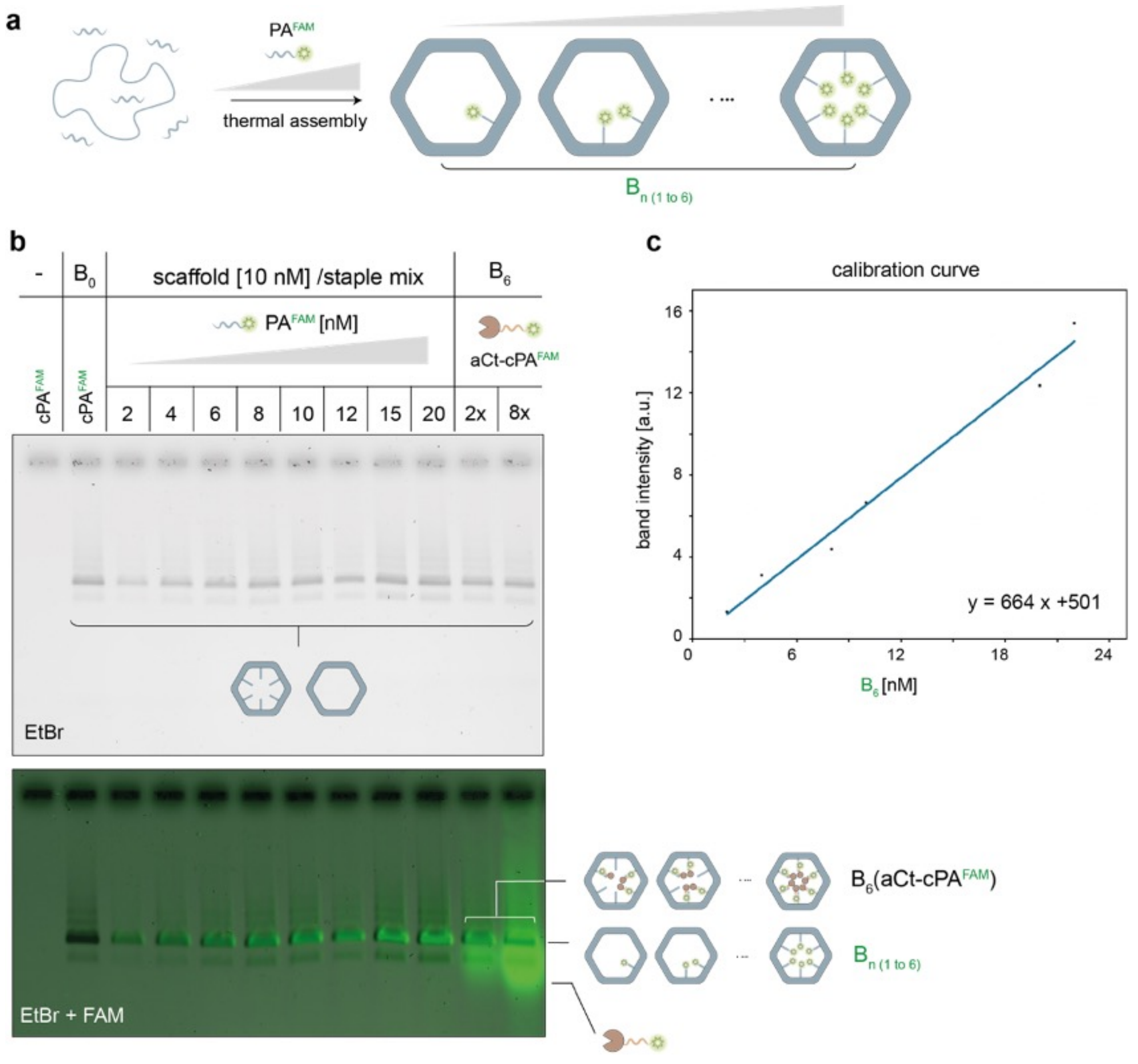

Supplementary Figure 29. AGE-based determination of aCt loading efficiency (method II). (**a**) The fluorescent signal measured in this method is given by incorporation of the PA^{FAM} strand into the DNA origami compartment during the thermally driven assembly. Contrary to method I, where the signal derives from the number of strands that are complementary to the inner PAs of a B_6 structure, here the signal derives directly from the number of fluorescently labelled PAs that incorporate into a B_n structure (*n* indicates the number of incorporated PAs). (**b**) Different concentrations of PA^{FAM} strands (from 2 nM to 20 nM) are added to an origami assembly mixture containing 10 nM scaffold (leading to 10 nM concentration of origami compartment). (**c**) The calibration curve correlates the intensity of the fluorescently labelled origami band with the concentration of PA used in the assembly mixture and thus with the number of PA incorporated into the final construct. In a different experiment, aCt-cPA^{FAM} conjugate is added in 2-fold or 8-fold to a 10 nM solution of B_6. The intensity of the fluorescently labelled origami band is then used to calculate the concentration of the aCt-cPA conjugate loaded within the DNA origami cage. This procedure has been repeated for every newly synthesized aCt-cPA^{FAM} conjugate, creating a new calibration curve for each gel experiment. This method provides a roughly quantitative estimation of the encapsulation yield and relies on the following assumptions: (i) incorporation of PAs into the B_n cage is quantitative for every value of *n*, (ii) all PAs of the B_6 construct are equally accessible for hybridization and (iii) the intensity of the FAM signal is proportional to the amount of FAM molecules *incorporated within the cage during the assembly* process and no other fluorescent phenomena are considered that may affect the observed fluorescent signal (e.g DNA-induced quenching or photobleaching). When compared to method I (Suppl. Fig. 28), this procedure leads to similar results, with a loading yield of ca. 60-70 % using a 2-fold excess aCt-cPA^{FAM} conjugate. Gel running conditions: 1 % agarose in 1x TBEMg at 80 V, for 2 h, at 4 °C. The gel was scanned with a Typhoon FLA9000 (GE healthcare Life Sciences) after FAM illumination and finally stained with ethidium bromide.

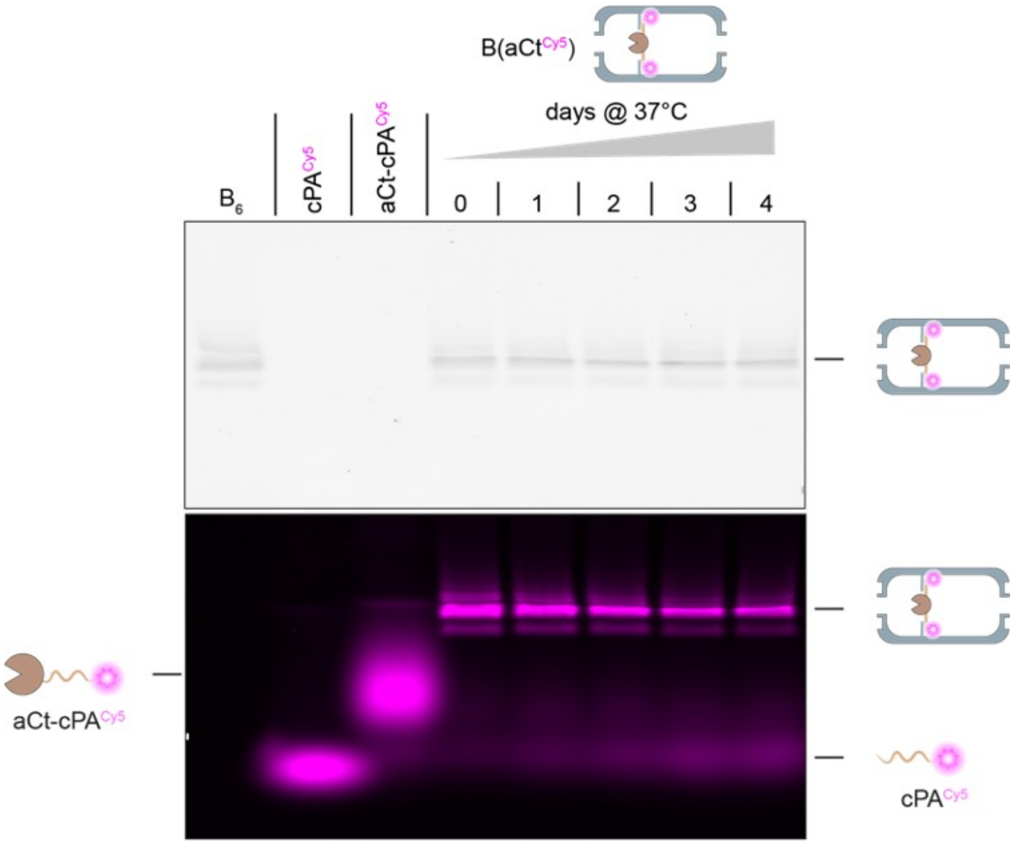

Supplementary Figure 30. Limited autoproteolytic digestion of the DNA-encaged aCt. AGE characterization of the B(aCt) construct at different time points showed that the complex was stable up to 4 days at 37 °C and that the enzyme did not escape in solution. The lack of fluorescently labelled bands with a migration rate similar to that of aCt also suggests that, once immobilized within the DNA origami compartment, the protease is protected from autoproteolysis. Gel running conditions: 1 % agarose in 1x TBEMg at 80 V, for 2.5 h, at 4 °C. The gel was scanned with a Typhoon FLA9000 (GE healthcare Life Sciences) after Cy5 illumination and finally stained with ethidium bromide.

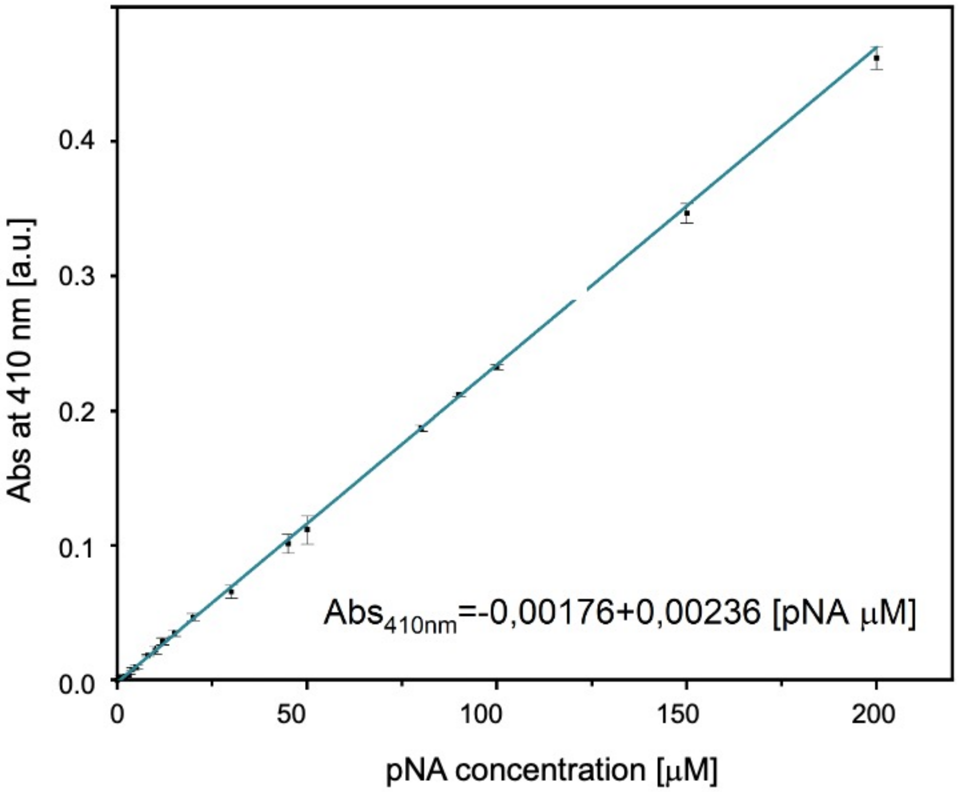

Supplementary Figure 31. Calibration curve for determination of peptide substrate concentration. The proteolytic activity of chymotrypsin was assessed using a commercially available chromogenic substrate (N-Succinyl-Ala-Ala-Pro-Phe//pNA). Upon enzymatic cleavage at the Phe residue, the concentration of the released chromogenic pNA was determined photometrically by measuring the absorbance of the solution at 410 nm. Accordingly, a standard curve was generated to relate the change in pNA absorption signal to a change in product concentration. Data are presented as mean ± SD from n = 3 technical replicates per sample.

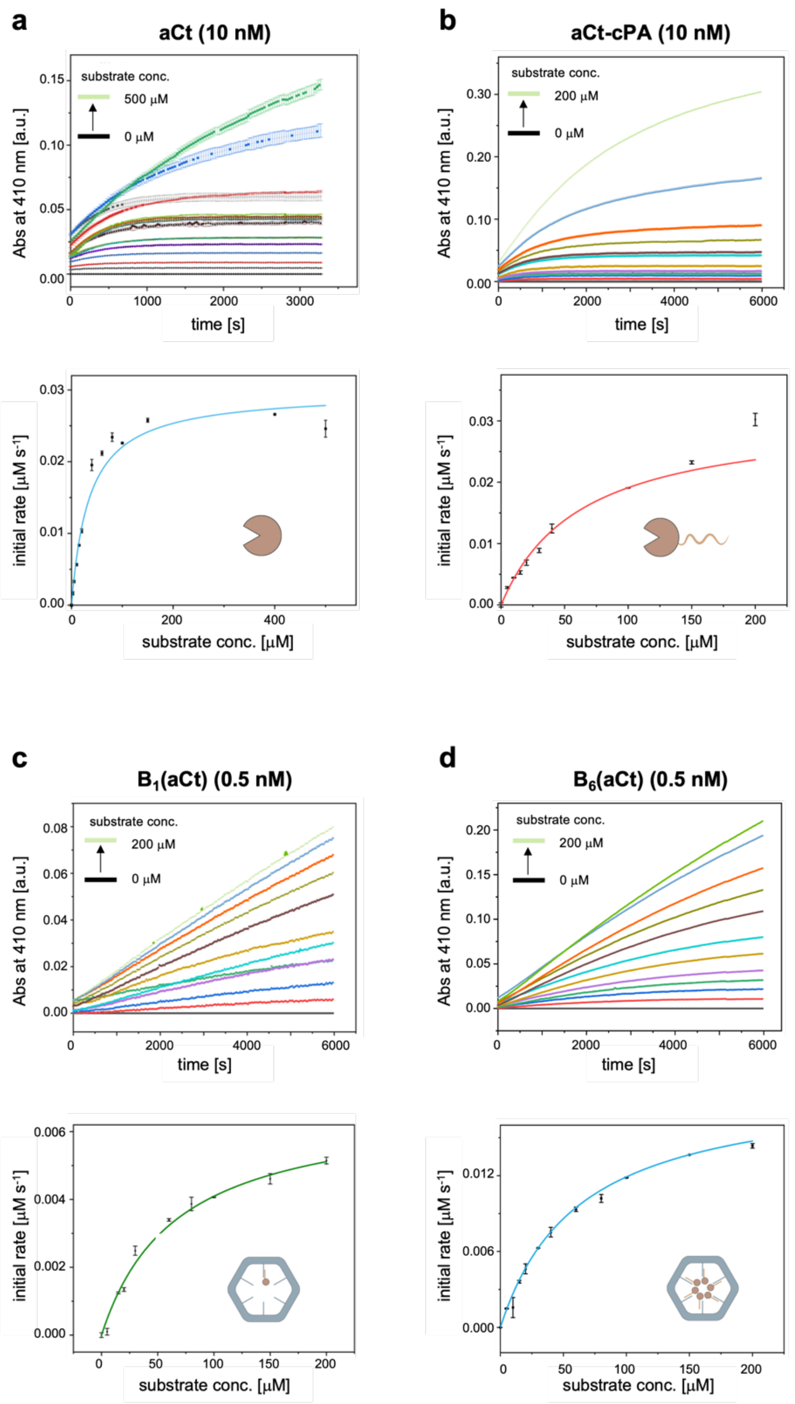

Supplementary Figure 32. Enzymatic activity of aCt and its DNA-modified forms. The enzymatic activity of native aCt (**a**), aCt-cPA conjugate (**b**) and DNA-encaged aCt (either within a DNA origami chamber containing 1 PA or 6 PAs, **c** and **d,** respectively) was measured using a commercially available chromogenic substrate (N-Succinyl-Ala-Ala-Pro-Phe//pNA). Progress curves (upper panels) were monitored at 410 nm, in TE buffer containing 5 mM $MgCl_2$ and 3 mM $CaCl_2$ at 30 °C, using aCt concentrations of 0.5 nM (encaged forms) or 10 nM (free forms), and various substrate concentrations (ranging from 0 to 200 µM or 500 µM). Progress curves are representative examples from one technical replicate. The experiments were repeated to extract the corresponding kinetic parameters of the reaction by fitting the initial rate vs substrate concentration according to the Michaelis-Menten model (lower panels). Data points in the MM curves are presented as mean ± SD from $n = 2$ or 3 biological replicates per sample (Suppl. Table 3).

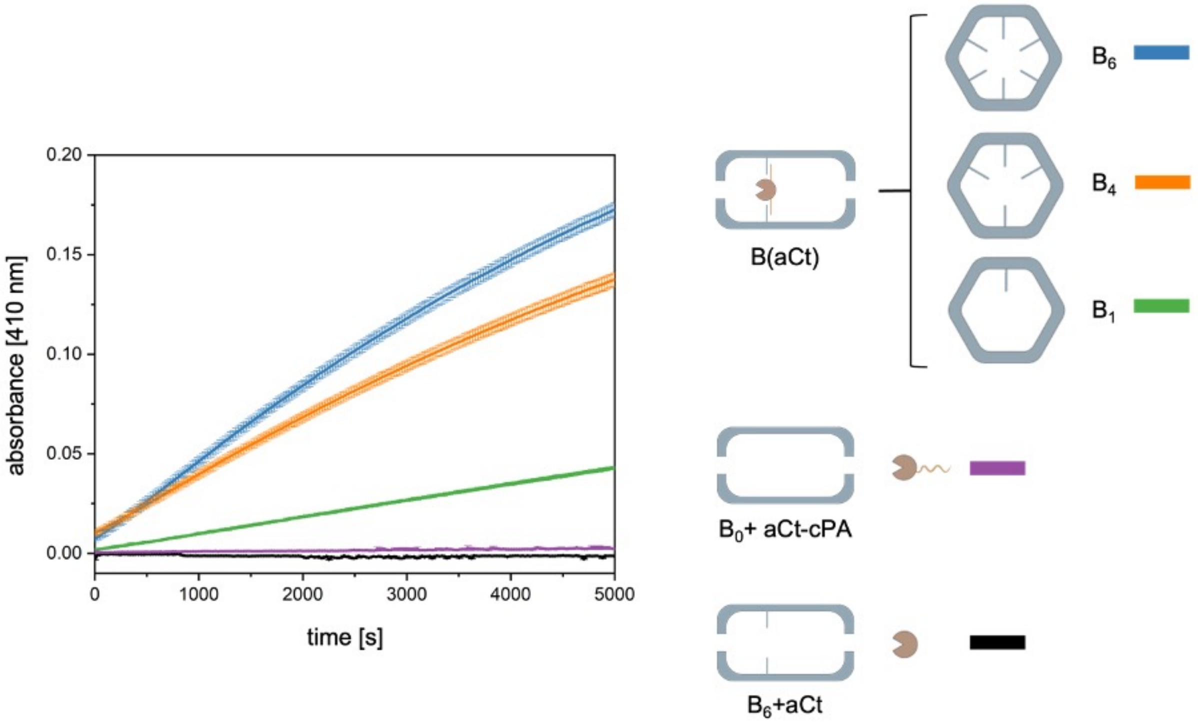

Supplementary Figure 33. Effect of aCt encapsulation on the relative initial rate of proteolysis. The rate of the enzymatic reaction enhanced upon encapsulation of the enzyme within the DNA origami compartment and was proportional, although not in a linear fashion, to the number of protruding arms pointing towards the inner cavity of the chamber. This indicates that increasing the number of strands to which the cPA tag can hybridize, correspondingly increases the number of aCt-cPA molecules trapped within the cage and thus the effective enzyme concentration. Using 6 PA, the increase in velocity was about 4.5-fold the value observed for 1 PA, in agreement with the maximal yield of protein encapsulation measured by gel assays (which results in about 4 molecules of aCt per DNA compartment). Reaction conditions: 0.5 nM aCt (either free or encaged within a DNA origami chamber); 100 µM pNA substrate. Reaction buffer and experimental conditions as in the enzymatic assays in Suppl. Fig. 32. Data are representative of a single biological replicate.

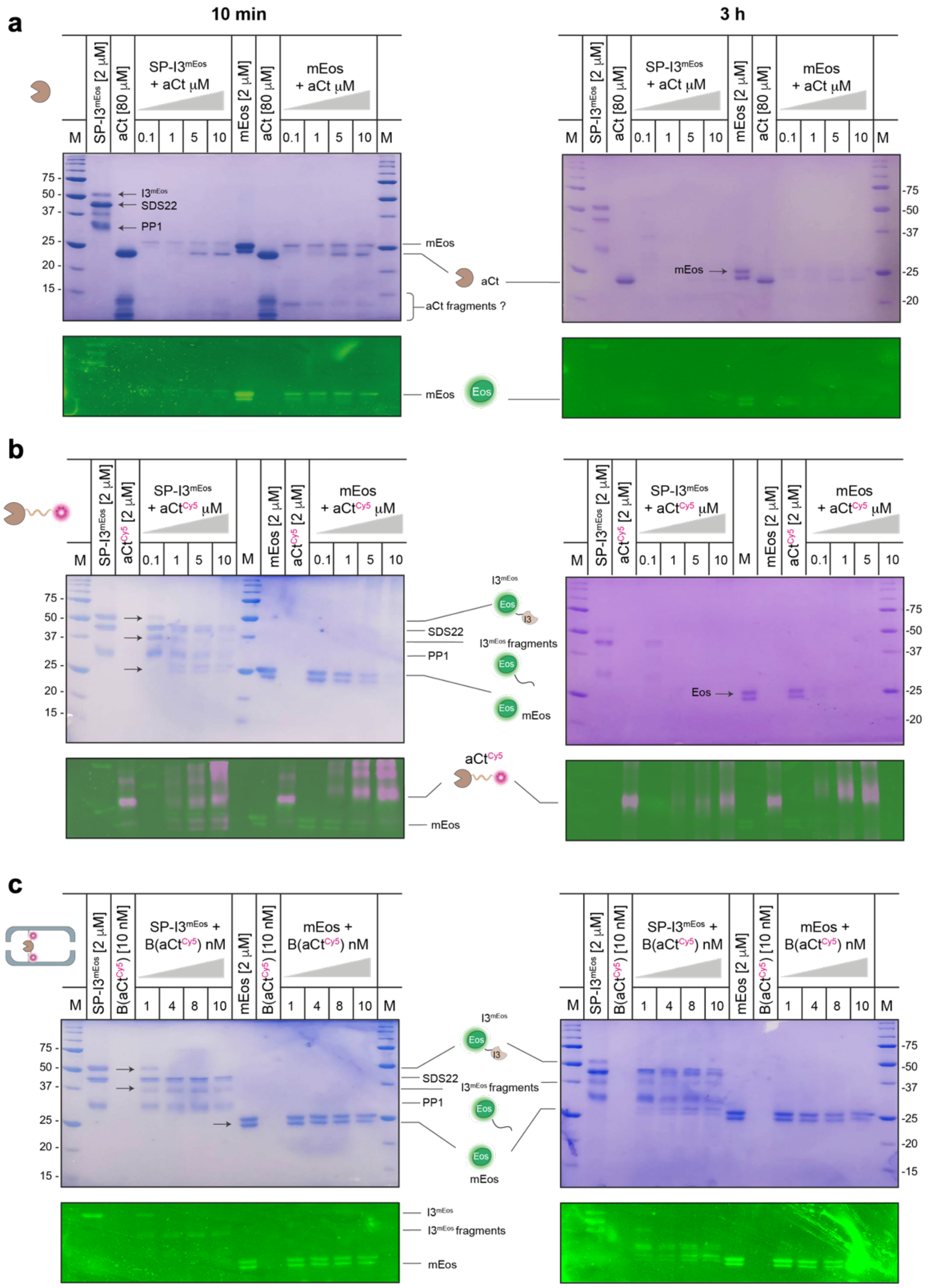

Supplementary Figure 34. Gel-electrophoretic analysis of substrate digestion. The proteolytic digestion of the green mEos protein, either alone or fused to I3 and in complex with SDS22 and PP1(forming SP-I3^{mEos}), was analyzed by SDS PAGE after 10 min or 3 hours incubation at 37 °C (left and right panels, respectively). Each solution contained 2 µM SP-I3^{mEos} or 2 µM green mEos (also loaded as references), and a varying concentration of aCt (ranging from 0.1 to 10 µM), with the enzyme available either in its free unmodified form (**a**) or conjugated to a DNA strand (**b**). A DNA-encaged aCt sample was also analyzed and showed that whereas I3 was easily degraded, the concentration of the enzyme was too low (up to 10 nM) to observe a substantial proteolytic digestion of mEos over this time window (**c**). The gel was scanned with a Typhoon FLA9000 (GE healthcare Life Sciences) at different wavelengths to visualize mEos (green channel) or fluorescently labeled aCt (Cy5 channel) (lower gel insets) and finally stained with Coomassie (upper gel insets). Gel running conditions: 15 % SDS PAGE in 1x SDS running buffer at 180 V for 1.5 h.

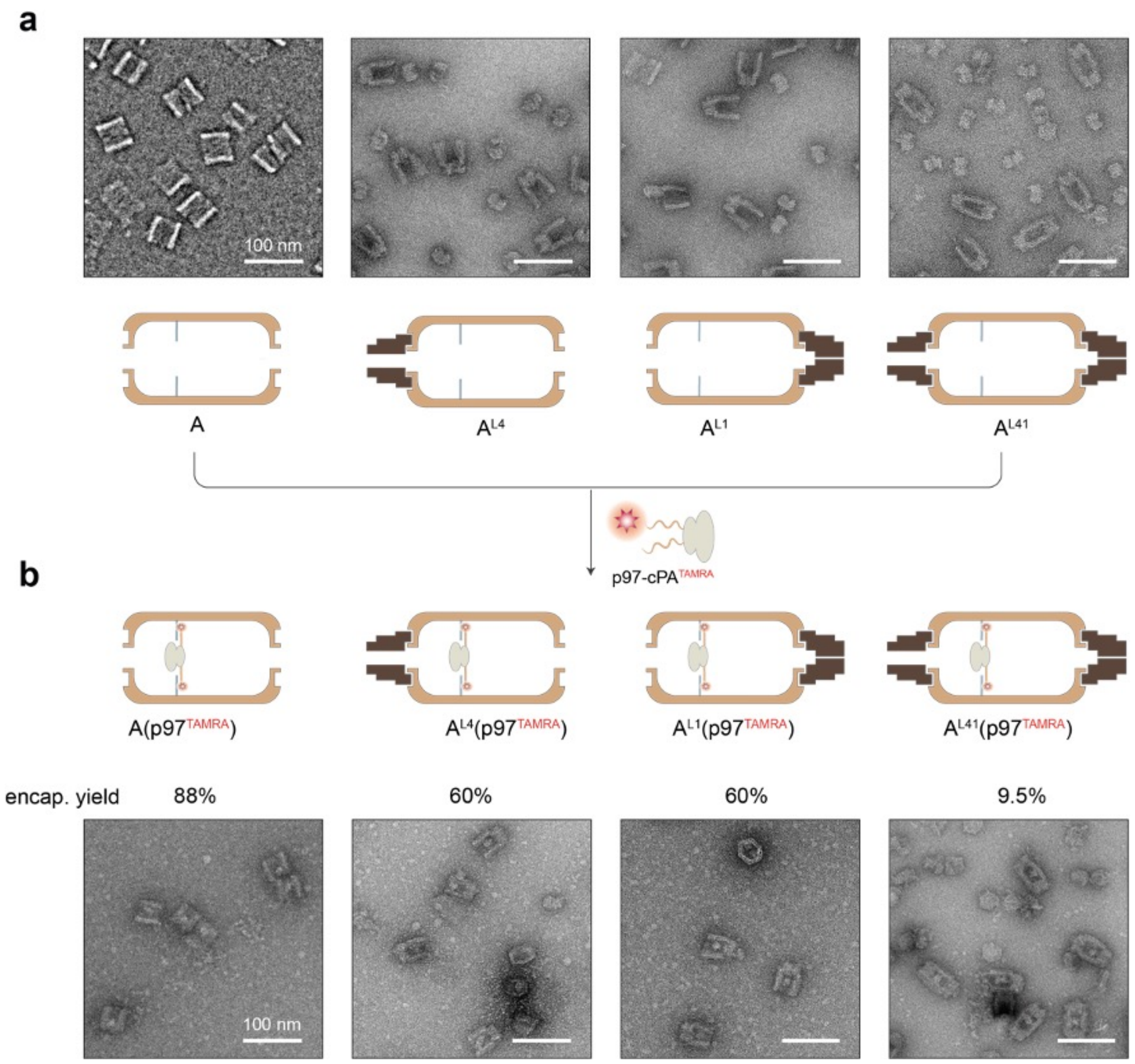

Supplementary Figure 35. Permeability of the DNA compartments to p97-cPA^{TAMRA} conjugates. The diffusion of the p97-cPA^{TAMRA} conjugate through the helical layers of a DNA origami compartment was analyzed in dependence of the accessibility of the compartment. This was either open on both sides (A) or equipped with one wide lid on the left side (A^{L4}), a narrow lid on the right side (A^{L1}), or both lids (A^{L41}). The amount of protein passing through the DNA walls of the various compartments was estimated by negative stain TEM. As expected, the presence of both lids at the entries of the chamber limited the diffusion of the large p97-HaloTag protein (ca. 780 kDa, see also Suppl. Table 1) within the DNA cavity and hindered the encapsulation yield to less than 10 %.

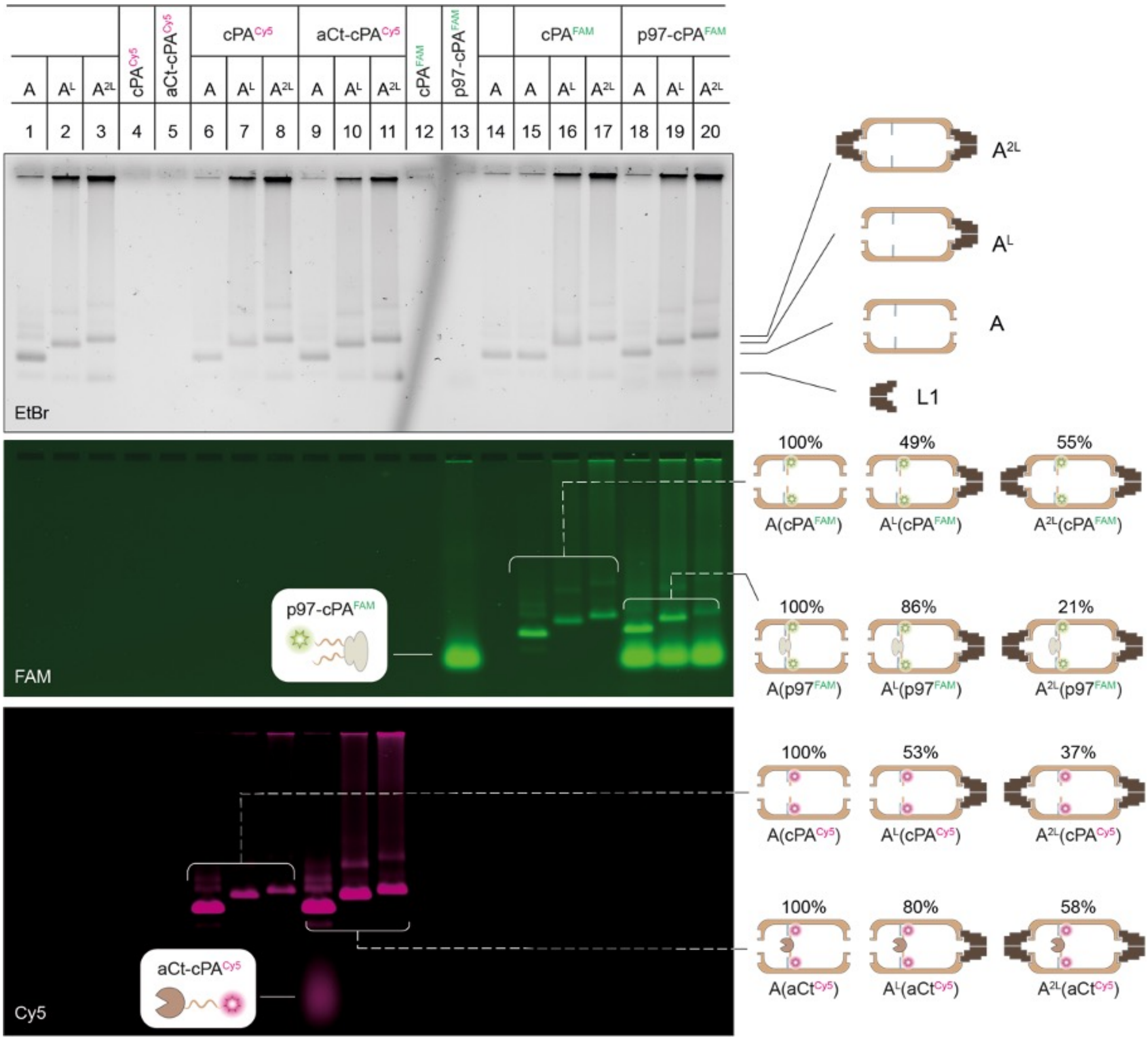

Supplementary Figure 36. Permeability of the DNA compartments to aCt-cPA^{Cy5} and p97-cPA^{FAM} conjugates. The diffusion of different molecular species through the helical layers of a DNA origami compartment was analyzed in dependence of the accessibility of the compartment (A, A^{L} and A^{2L}), the type and size of the molecular cargo (cPA strand, aCt or p97 protein) and the dye used for labelling the various species (Cy5 or FAM). Reaction products were analyzed by AGE using fluorescently labelled ssDNA (cPA^{Cy5}; lanes 6-8; or cPA^{FAM}; lanes 15-17) and their protein conjugates (aCt-cPA^{Cy5}, lanes 9-11 or p97-cPA^{FAM}; lanes 18-20). All protein species displayed one or more cPA handles which were complementary to the 6 PAs pointing towards the inner cavity of the chamber. The intensity of the fluorescently labelled band associated to the cPA sequences was assumed to be proportional to the extent of internalized cargo. Densitometric analysis of the gel bands was performed using ImageJ. The results are reported as relative intensity values with respect to the band of higher intensity. Samples containing the same molecular cargo were used for comparison. The data indicate that the interior of the DNA origami compartment is less accessible when the chamber is closed at one or both entries with a lid, with A^{2L} showing the lowest permeability to a given cargo and bulky cargos diffusing to a lower extent. The net charge of the molecular cargo and the type of dye attached to it also seem to affect the encapsulation yield; however, in a way that is still under examination and beyond the scope of this study. Gel running conditions: 1 % agarose in 1x TBEMg at 80 V, for 3 h, at 4 °C. The gel was scanned with a Typhoon FLA9000 (GE healthcare Life Sciences) at different wavelengths and finally stained with ethidium bromide.

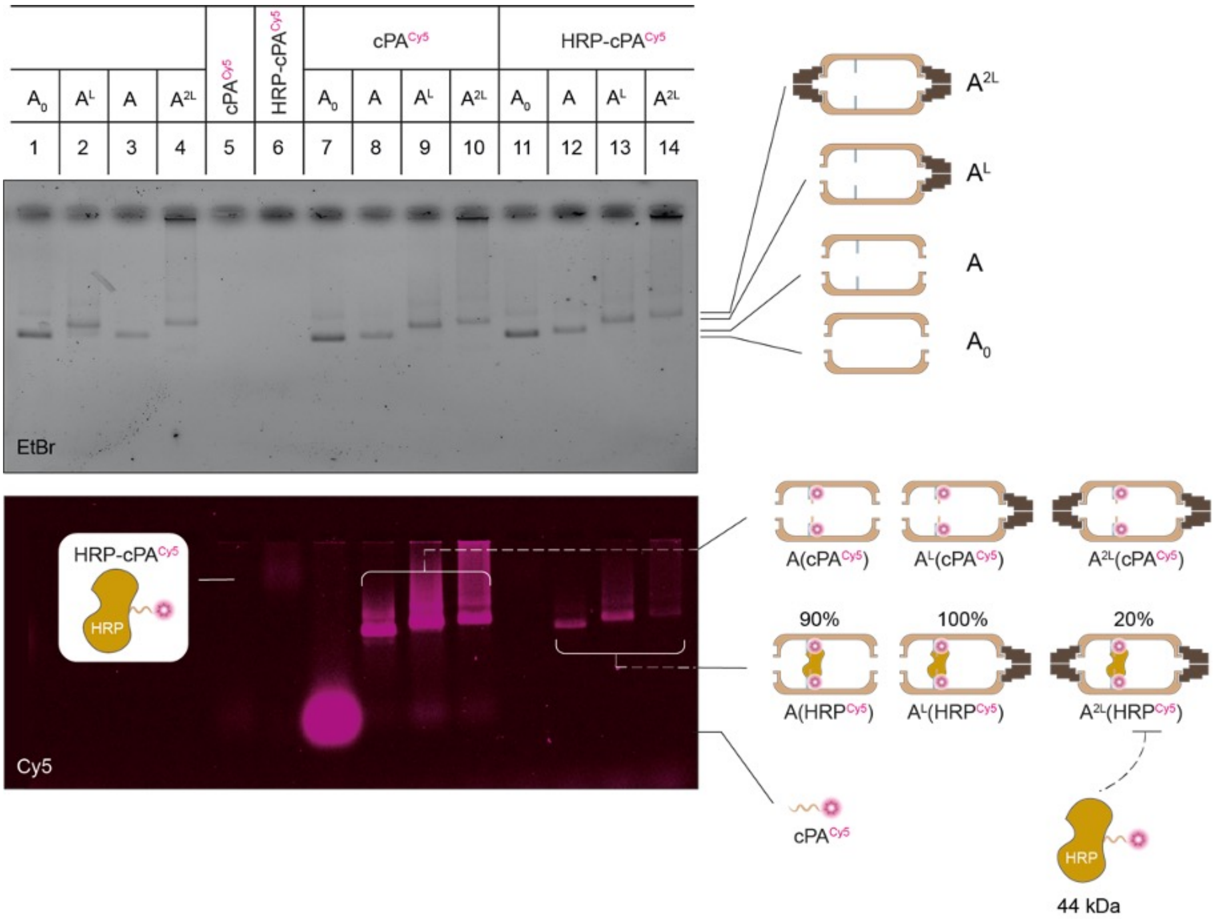

Supplementary Figure 37. Permeability of the DNA compartments to HRP-cPA^{Cy5} conjugates. The diffusion of horseradish peroxidase HRP-cPA^{FAM} through the helical layers of a DNA origami compartment was analyzed in dependence of the accessibility of the compartment (A, A^{L} and A^{2L}). All compartments contained 6 PAs which were complementary to the cPA sequences linked to the protein. A chamber containing no protruding arms in its inner cavity (A$_0$) was used as reference. Reaction products were analyzed by AGE using fluorescently labelled ssDNA (cPA^{Cy5}; lanes 7-10) and the protein conjugate (HRP-cPA^{Cy5}, lanes 11-14). The intensity of the fluorescently labelled band associated to the cPA sequences was assumed to be proportional to the extent of internalized cargo. The results showed that binding of the cPA or cPA-conjugate was specific and occured only in presence of complementary PA handles (no signal in lane 7 and 11). Moreover, whereas the ssDNA could freely diffuse through the DNA origami walls, independently of the presence or absence of the lid(s), the size of the HRP-cPA conjugate was large enough (ca. 45 kDa) to hinder its diffusion through the pores of the A^{2L} cage (lowering the encapsulation yield to about 20 % of the value obtained for the A cage; cfr. lanes 14 and 12). Gel running conditions: 0.75 % agarose in 1x TBEMg at 80 V, for 2.5 h, at 4 °C. The gel was scanned with a Typhoon FLA9000 (GE healthcare Life Sciences) at different wavelengths and finally stained with ethidium bromide.

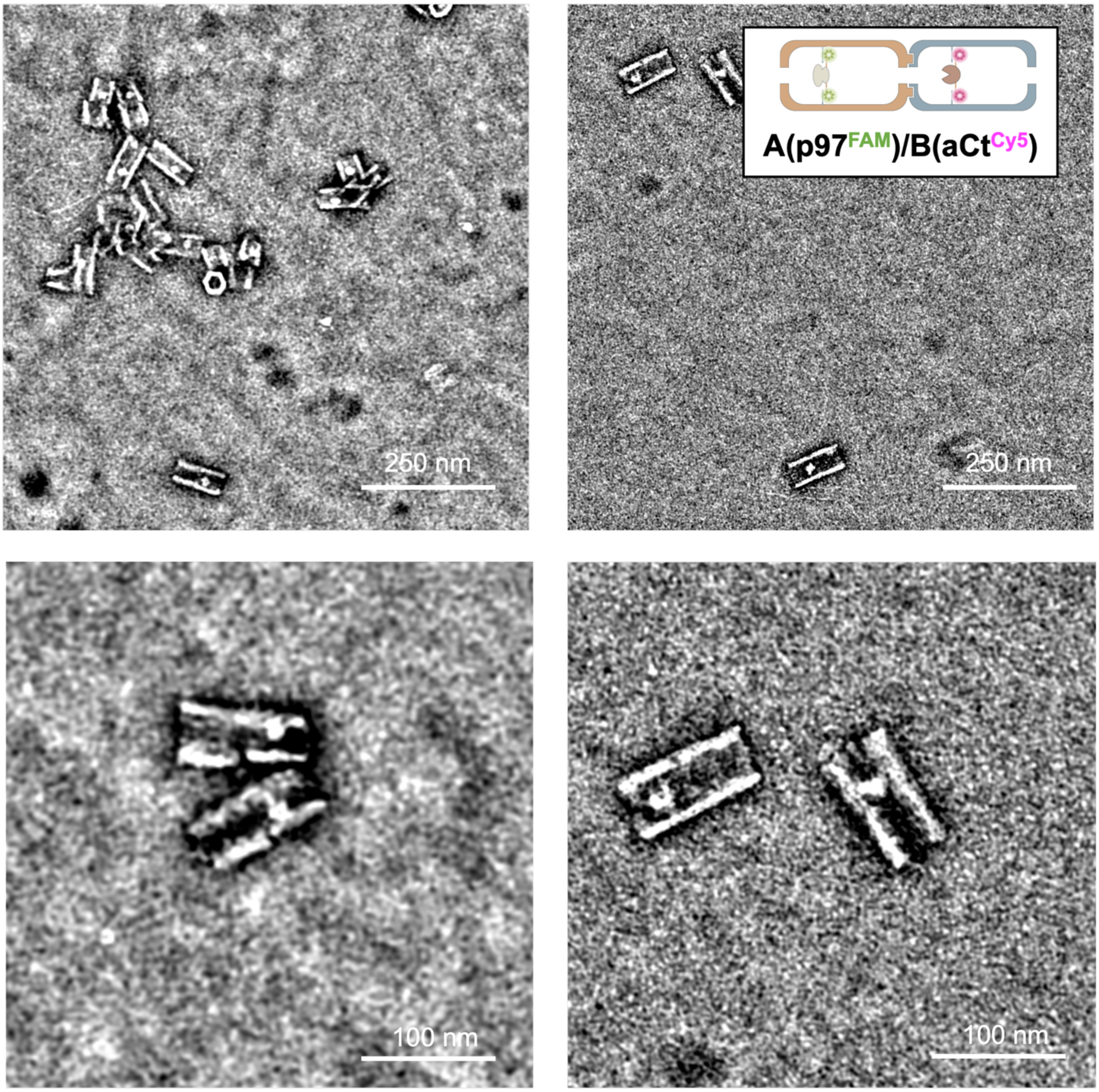

Supplementary Figure 38. TEM characterization of the A(p97)/B(aCt) construct. Successful encapsulation of the p97-cPA conjugate into the DNA origami chamber A containing 6 PAs was confirmed by negative stain TEM (ca. 40 % yield). The presence of aCt in chamber B was more difficult to observe by TEM, presumably because of its small size, which is below the resolution limit of our TEM microscope. Fluorescently labelled forms of the two proteins (p97-cPA^{FAM} and aCt-cPA^{Cy5}) however enabled to confirm the co-presence of both proteins within the dimeric AB constructs (see Fig. 5 of the main manuscript). Raw images (top rows) and enlarged views (bottom rows) are shown.

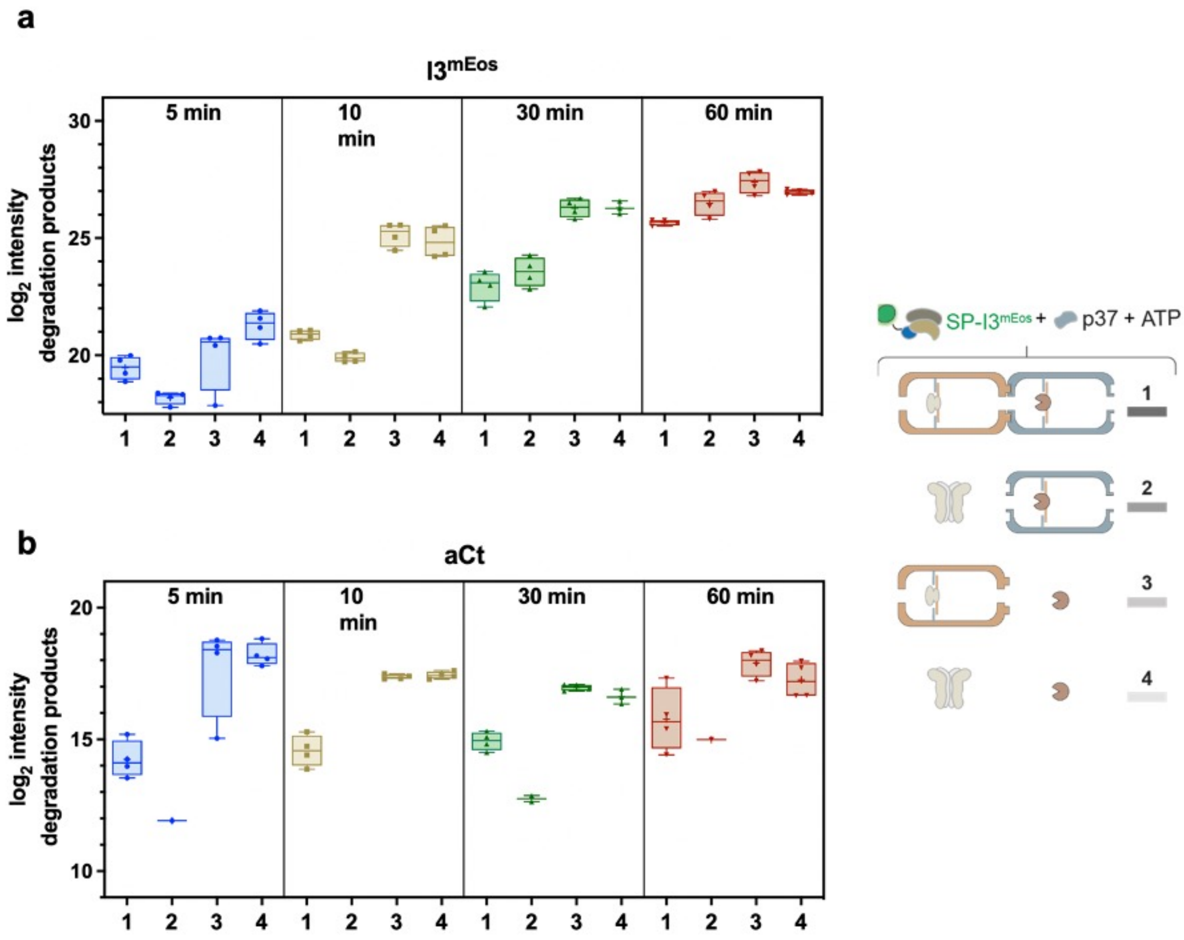

Supplementary Figure 39. LC-MS/MS analysis of the proteolytic digestion products of I3^{mEos} and aCt. Boxplot representation of quantification data (LFQ intensity in log_2 scale) for four sample settings: chimera construct (1), encaged aCt in presence of free p97 (2), encaged p97 in presence of free aCt (3) and unbound p97 and aCt enzymes in absence of DNA origami (4). Data were collected at different time points (5 min, 10 min, 30 min and 60 min) for I3^{mEos} **(a)** and aCt **(b)**. Data are presented as mean ± SD from $n = 4$ technical replicates per sample. Median values are indicated by horizontal lines for each data set. No outliers have been excluded.

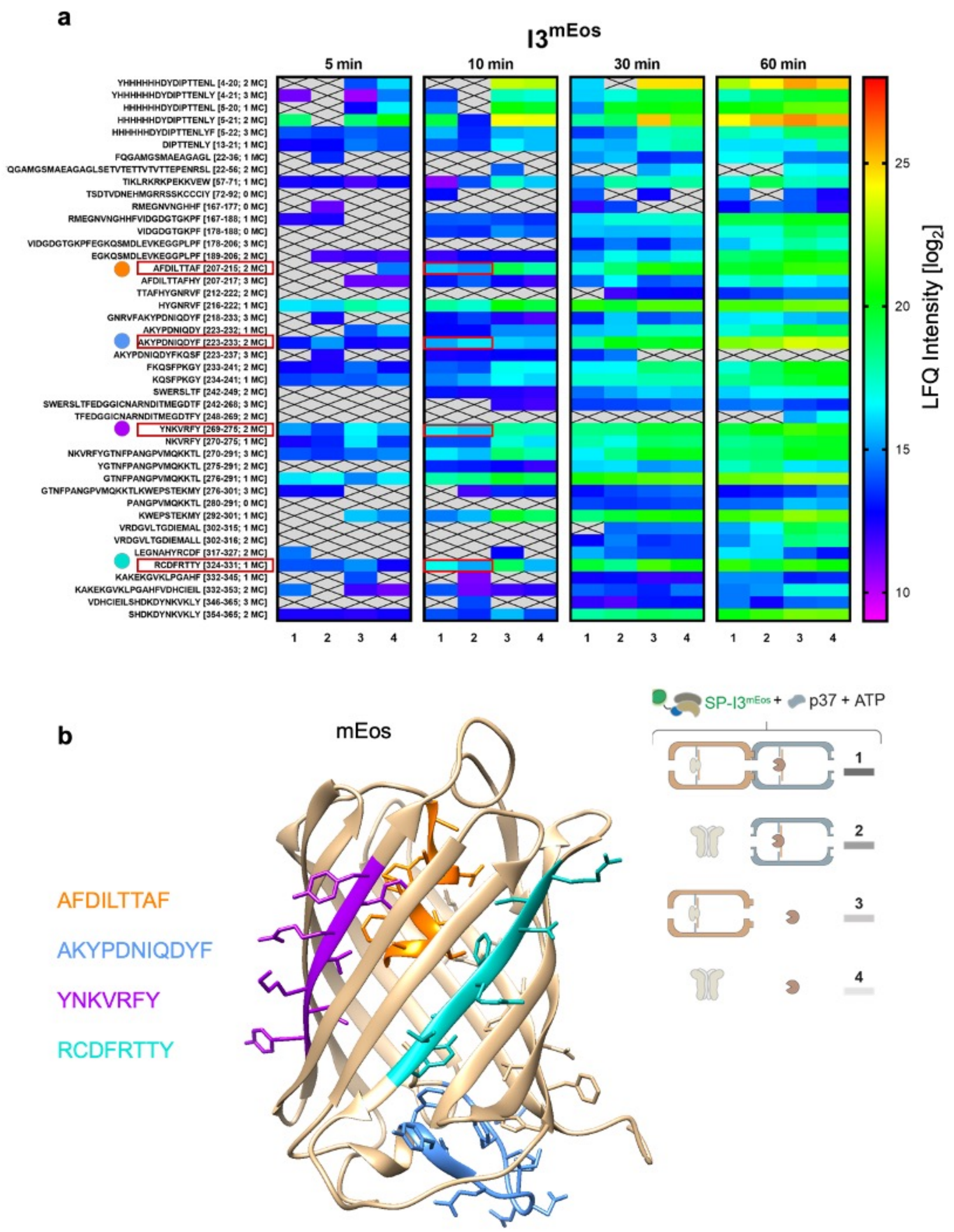

Supplementary Figure 40. Peptide fragments obtained from the proteolytic degradation of I3^{mEos}. (a) Heat map of I3^{mEos} peptides identified by LCMS-based proteomics after 5, 10, 30 or 60 min of digestion with the chimera construct (1), encaged aCt/free p97 (2), encaged p97/free aCt (3) and unbound p97 and aCt enzymes in absence of DNA origami (4). The row annotation denotes the peptide sequence in one-letter code with the amino acid position within the protein and the number of missed theoretical cleavage sites (MC) in parentheses. The respective colour of the areas represents the LFQ intensity values in a log_2 scale as a mean of $n = 4$ technical replicates as indicated in the right bar. **(b)** Representative peptide sequences of the mEos substrate that show distinct fragmentation patterns. The peptides belonging to the barrel structure (purple and cyan) or buried within it (orange) were more easily cleaved in sample 1 rather than sample 2, after 10 min (compare LFQ intensities highlighted by the red boxes). This was not true for the peptide stretch forming an exposed loop at the bottom of the structure (blue; see also Suppl. Fig. 41). This suggests that unfolding of the substrate can make some cleavage sites more accessible to proteolytic degradation.

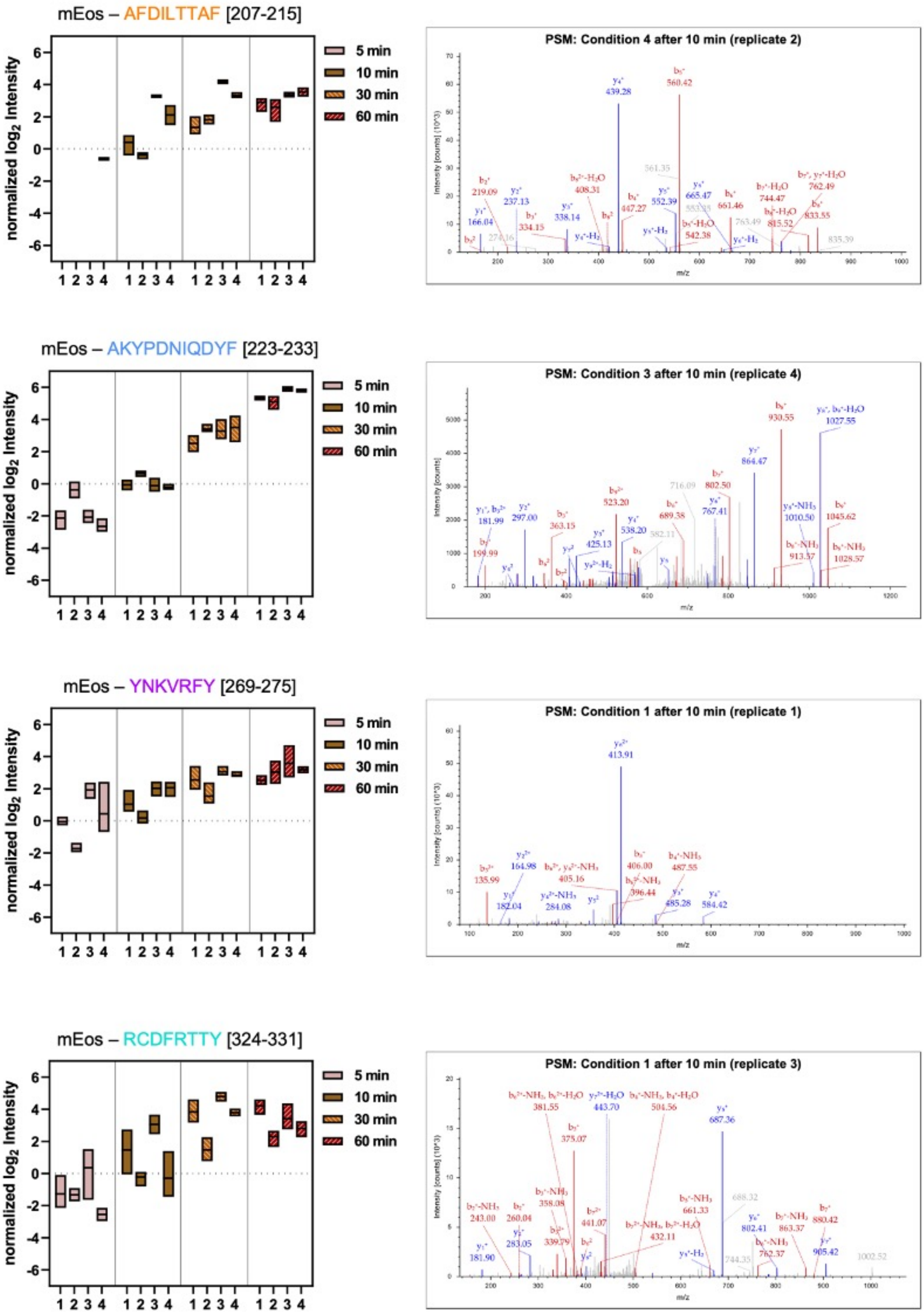

Supplementary Figure 41. Mass spectra of representative peptide fragments obtained from the proteolytic degradation of I3^{mEos}. LFQ intensities (left panels) and representative mass spectra (right panels) of the four peptide stretches identified in the folded form of mEos (Suppl. Fig. 41). In agreement with the predicted functioning of the compartmentalized p97-aCt chimera, the peptides belonging to the barrel structure (purple and cyan) or buried within it (orange) are more easily cleaved in sample 1 (p97-assisted unfolding) rather than sample 2 (encaged-aCt + free p97). On the contrary, the peptide stretch forming an exposed loop at the bottom of the structure (blue) is easily degraded by aCt also in absence of previous unfolding, probably because it is easily accessible also in the folded form of the substrate. Data are presented as mean ± SD from *n* = 4 technical replicates per sample.

Supplementary Table 1. Proteins involved in the nanoscale modular chimera.

**protein**		**MW (kDa)**
SP-I3^{mEos} (137 kDa)	SDS22	50
	PP1	37
	I3	25
	mEos 3.2	25
p37		40
p97-Halo tag hexamer		750
p97-cPA hexamer		780
aCt		25

Supplementary Table 2. Yields of structure assembly and protein loading based on counting of single particles observed by negative stain TEM. The number of particles counted is indicated in brackets. Chymotrypsin was too small to be visible by our TEM and its binding to the compartment was estimated by UV absorption upon calibration with a protein sample of known concentration (Suppl. Fig. 28-29).

	**Yield %**	
**Constructs**	**DNA origami assembly**	**protein loading**
A (or NE)	98<br>(3,065)	-
L1	98<br>(898)	
L2	95<br>(959)	
L3	97<br>(1,775)	
L4	95<br>(591)	
A^{L}	96<br>(1,477)	-
A^{2L}	98<br>(358)	-
AB	70<br>(354)	-
AB^{L}	38<br>(1,466)	-
A(p97)	98<br>(1,478)	75<br>(758)
A^{L}(p97)	98<br>(1,068)	77
B(aCt)	98	65
A(p97)/B	35<br>(787)	33
A/B(aCt)	39<br>(406)	-
A(p97)/B(aCt)	56<br>(680)	40
A(p97)/B^{L}(aCt)	36<br>(721)	-

Supplementary Table 3. Averaged Michaelis-Menten parameters of the proteolytic digestion of N-Succinyl-Ala-Ala-Pro-Phe//pNA by chymotrypsin.

	**Michaelis-Menten constant K_M (μM)**	**Turnover number k_{cat} (s^{-1})**	**k_{cat}/K_M (s^{-1} μM^{-1})**
aCt	38 ± 2	5 ± 2	0.13
aCt-cPA	53 ± 13	3 ± 2	0.06
B_1(aCt)	62 ± 6	13 ± 1	0.21
B_6(aCt)	60 ± 5	31± 11	0.52

Supplementary References

1. Martin, T.G. & Dietz, H. Magnesium-free self-assembly of multi-layer DNA objects. *Nat Commun* **3**, 1103 (2012).

Supplementary Source Data

Statistical source data for all the relevant supplementary figures are provided as Supplementary Data1. The full list of DNA sequences used for the assembly of the DNA origami structures is provided as Supplementary Data2.

Source data for Suppl. Fig. 4

Uncropped gel *Narcissus* (N)

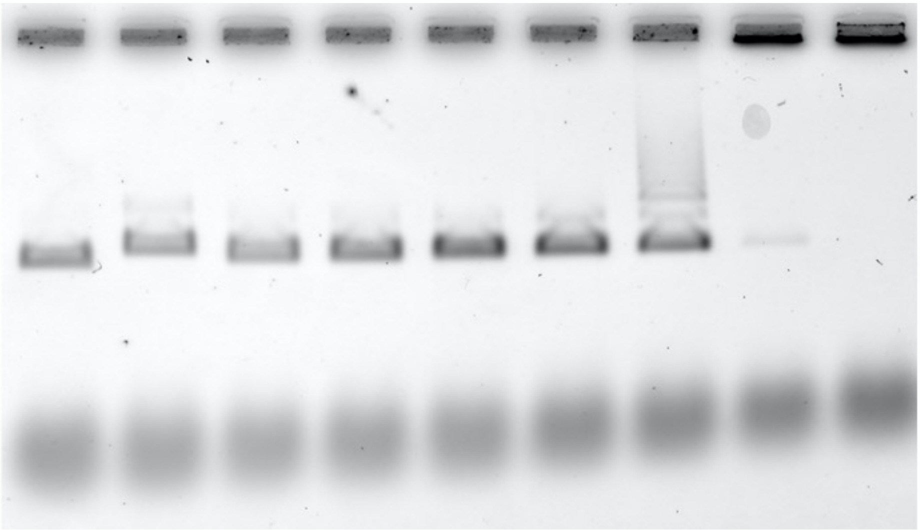

Uncropped gel *Echo* (E)

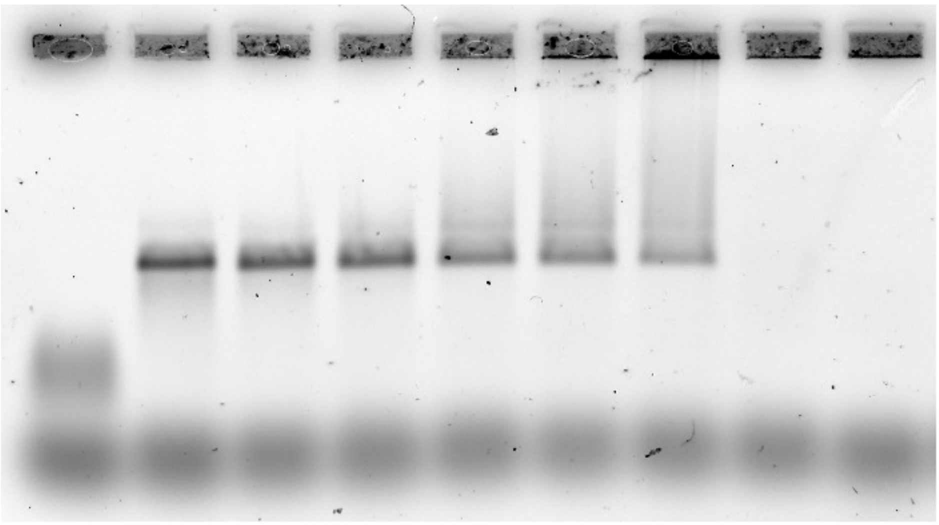

Source data for Suppl. Fig. 5

Large view AFM images (600 nm x 600 nm)

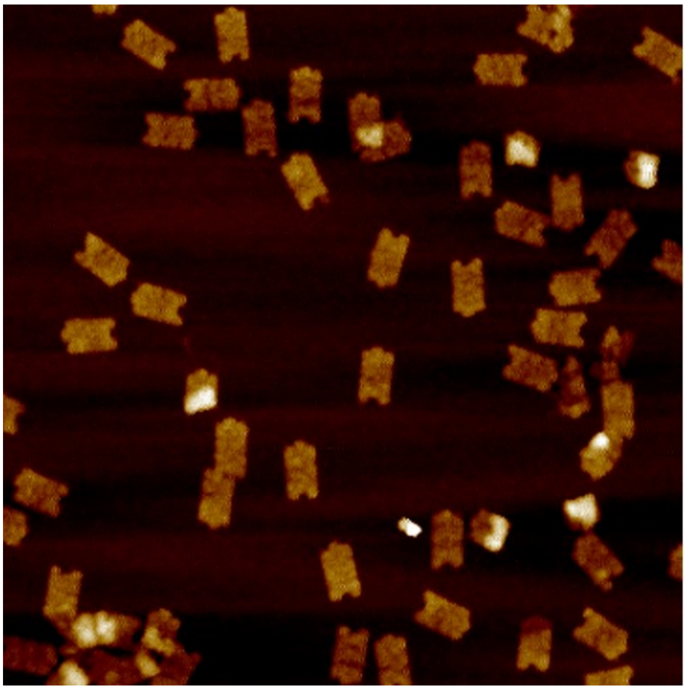

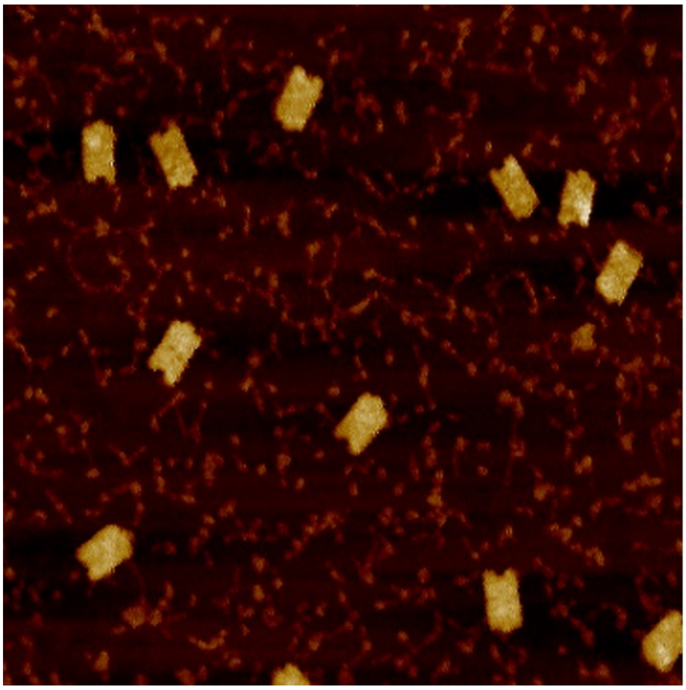

Source data for Suppl. Fig. 6

Class averages N

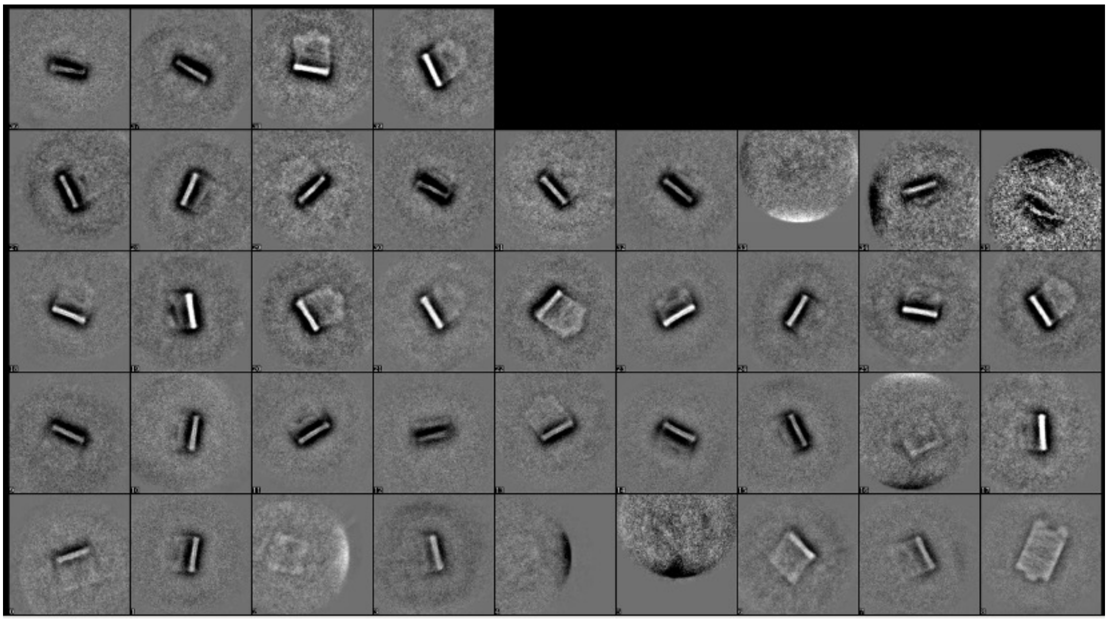

Class averages E

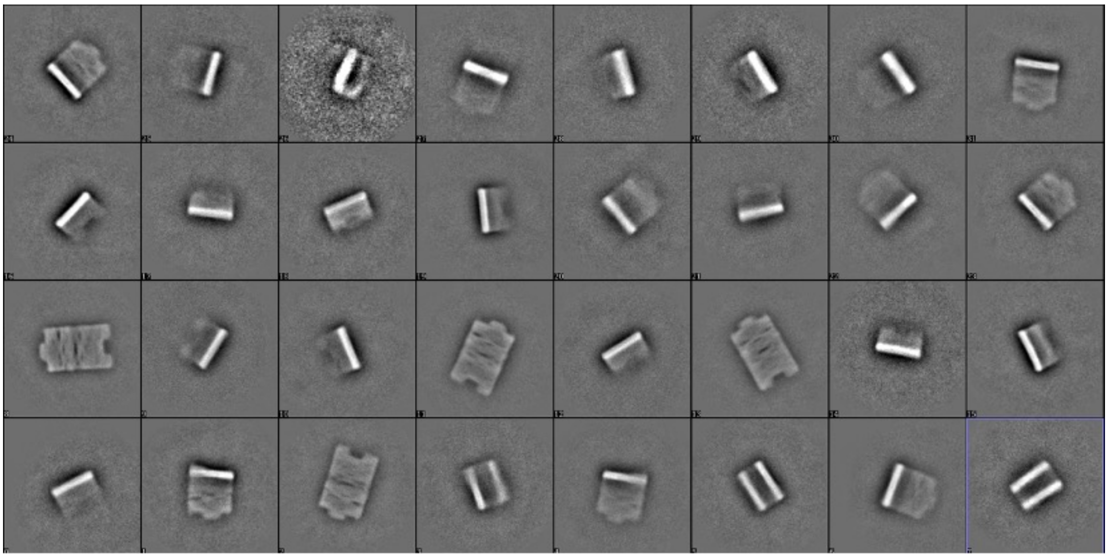

Source data for Suppl. Fig. 7

No PEG purification

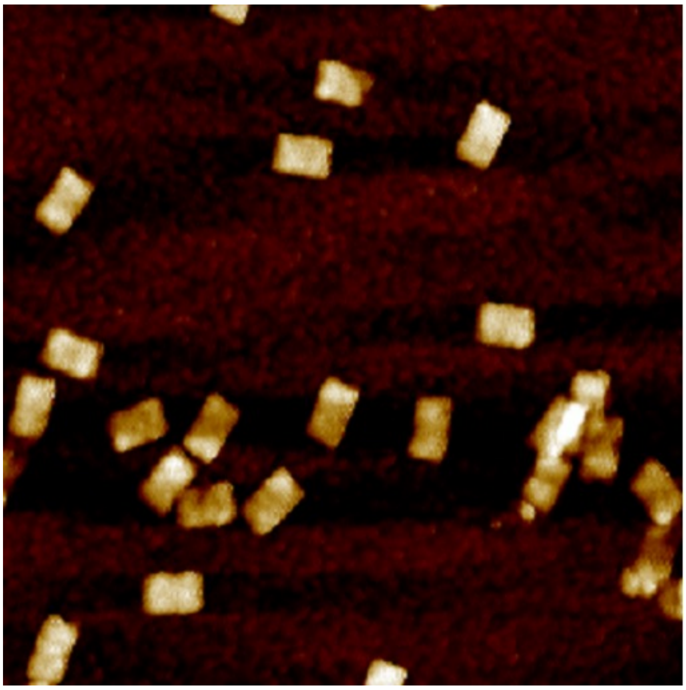

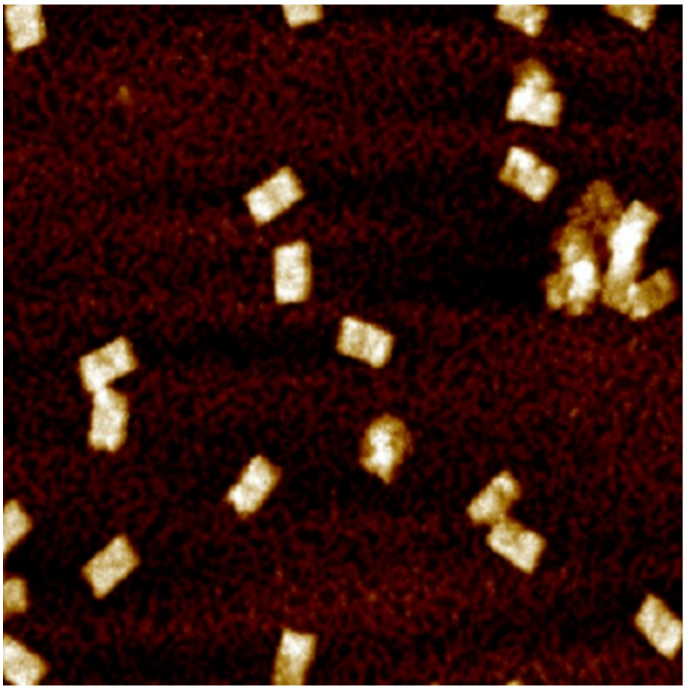

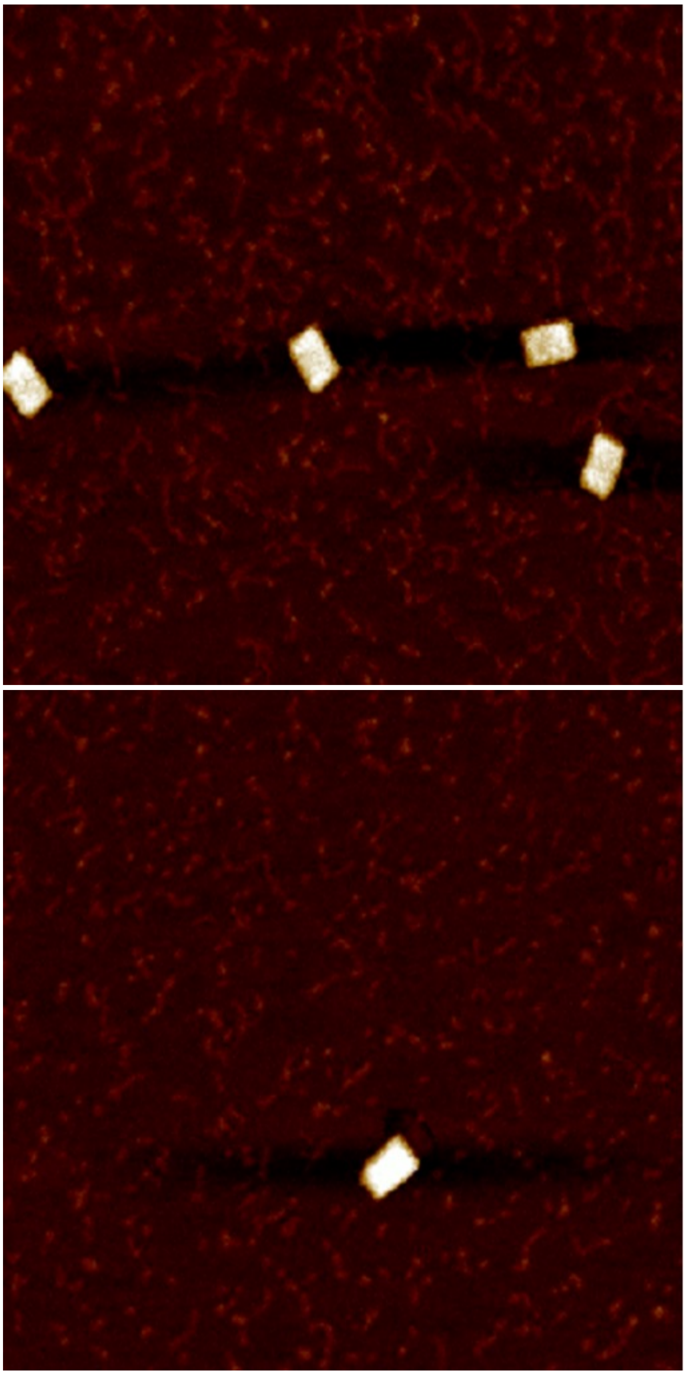

After PEG purification

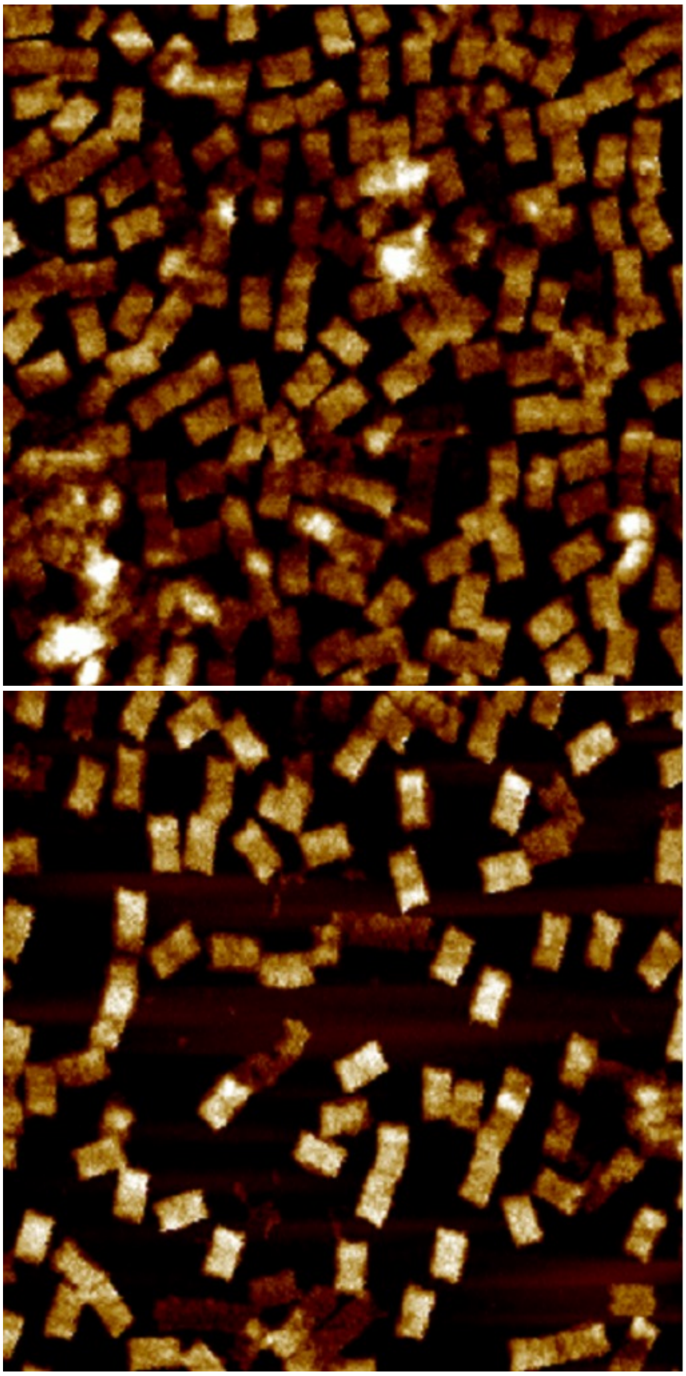

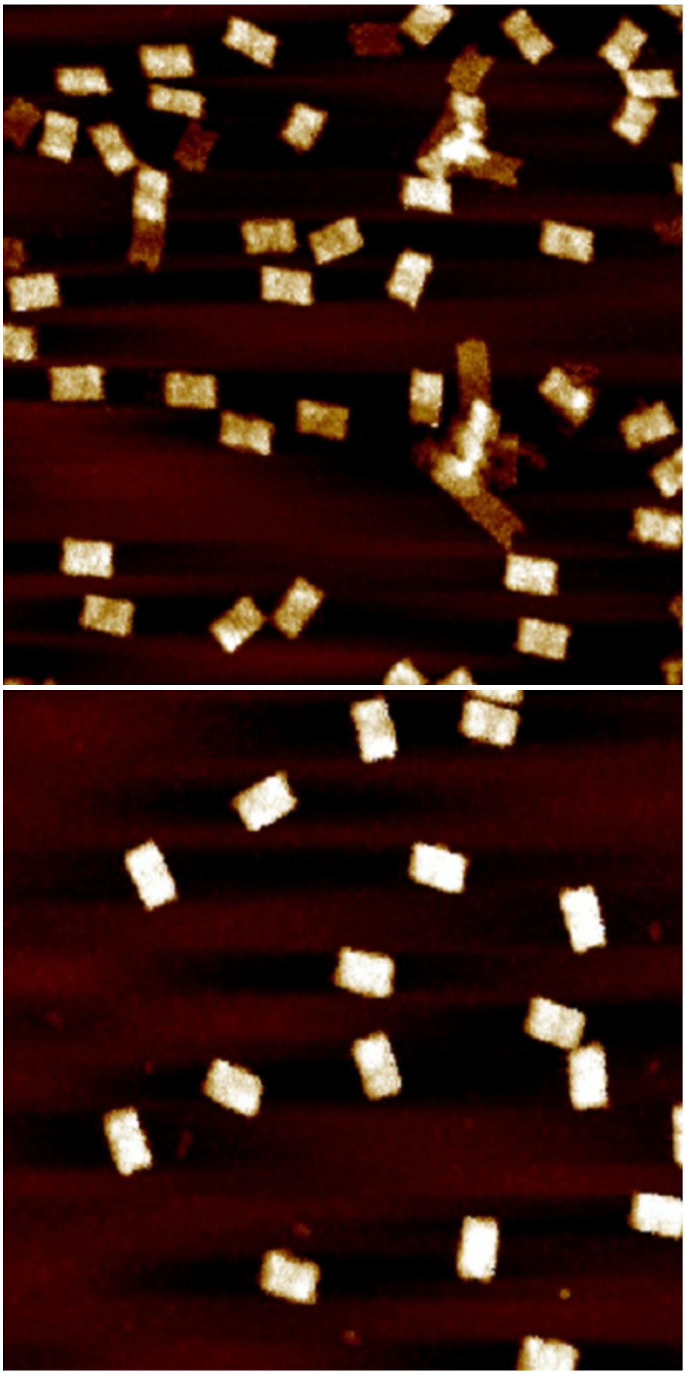

Source data for Suppl. Fig. 9

Uncropped gel after ethidium bromide staining

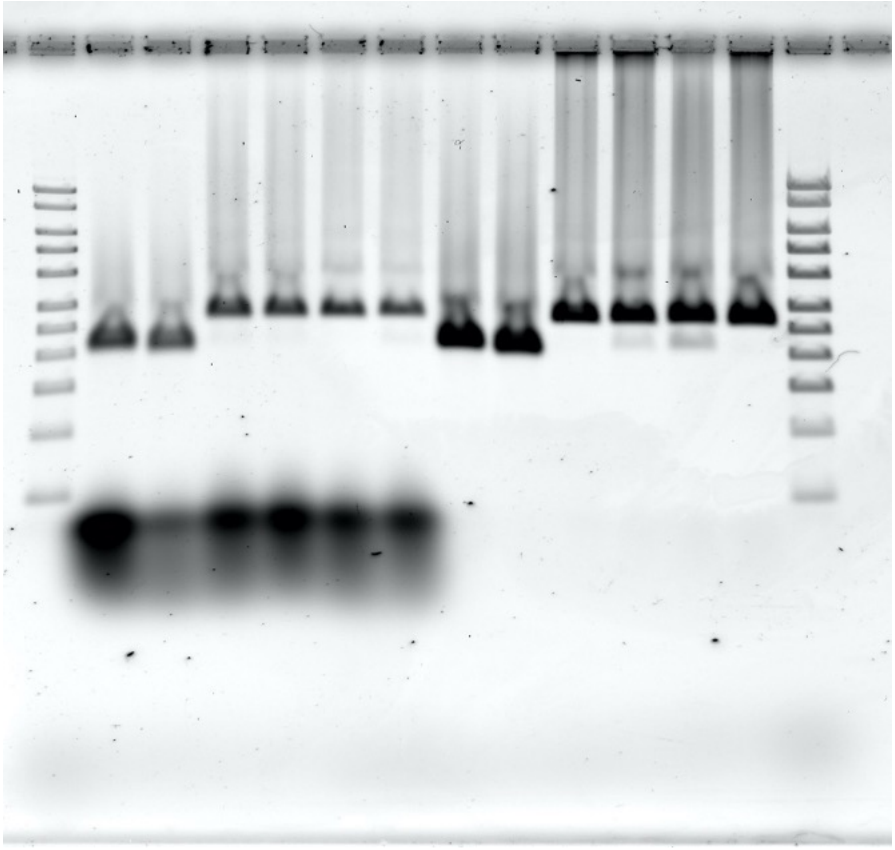

Same gel after FAM illumination

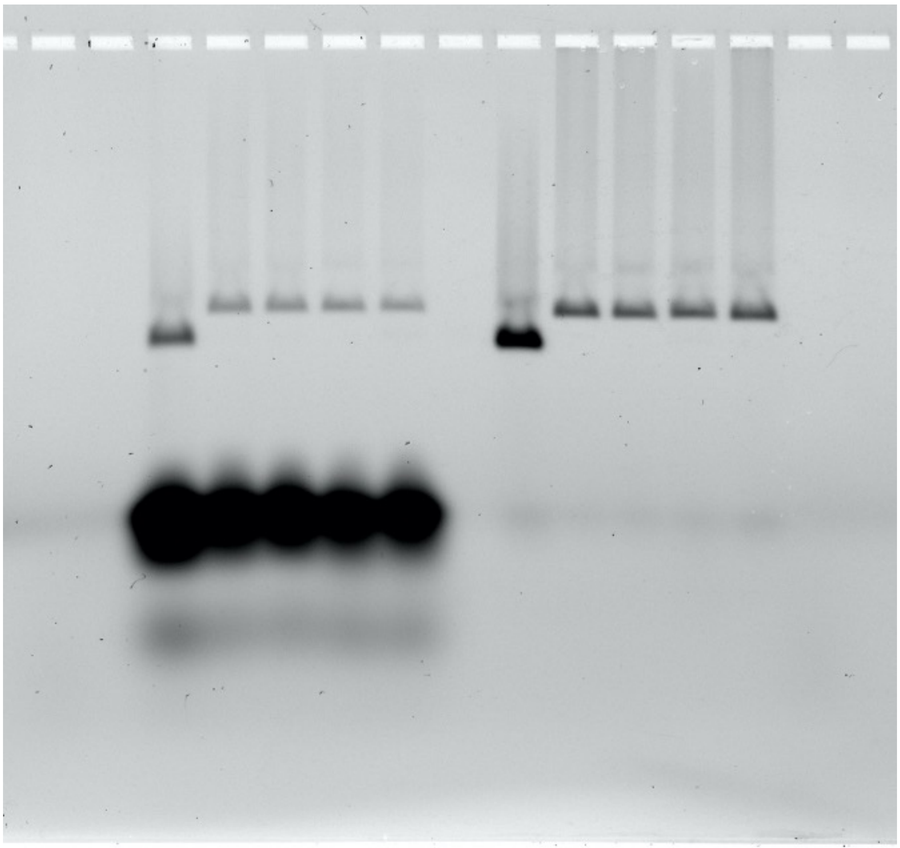

Same gel after TAMRA illumination

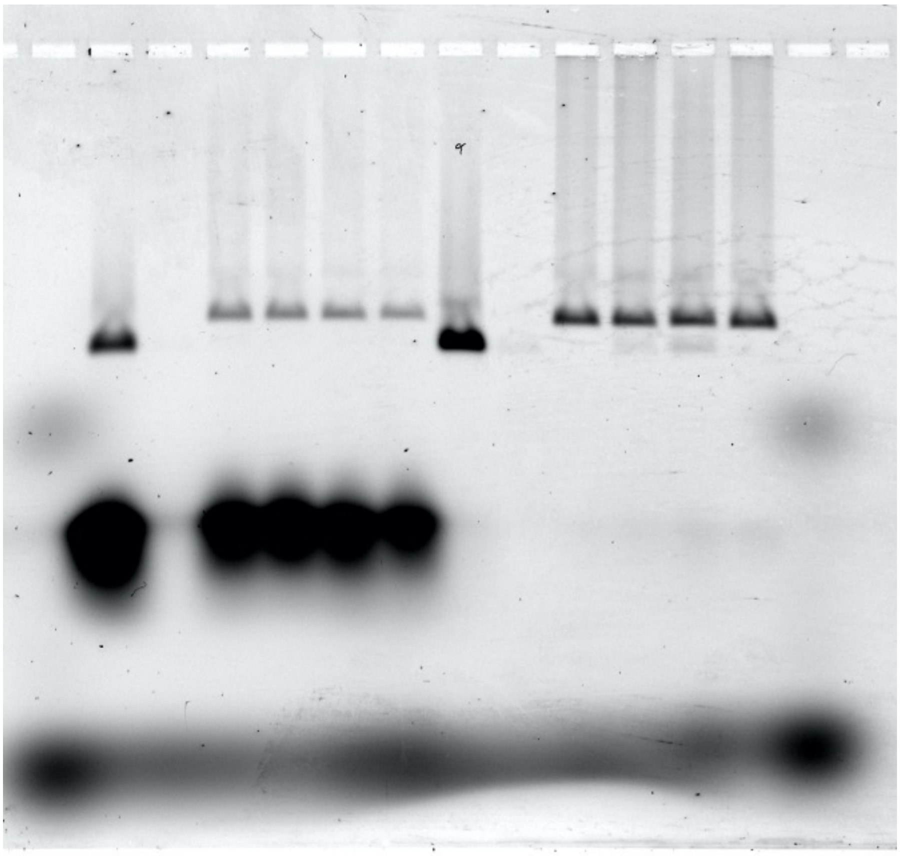

Source data for Suppl. Fig. 10

Wide-field TEM view of NE (or A)

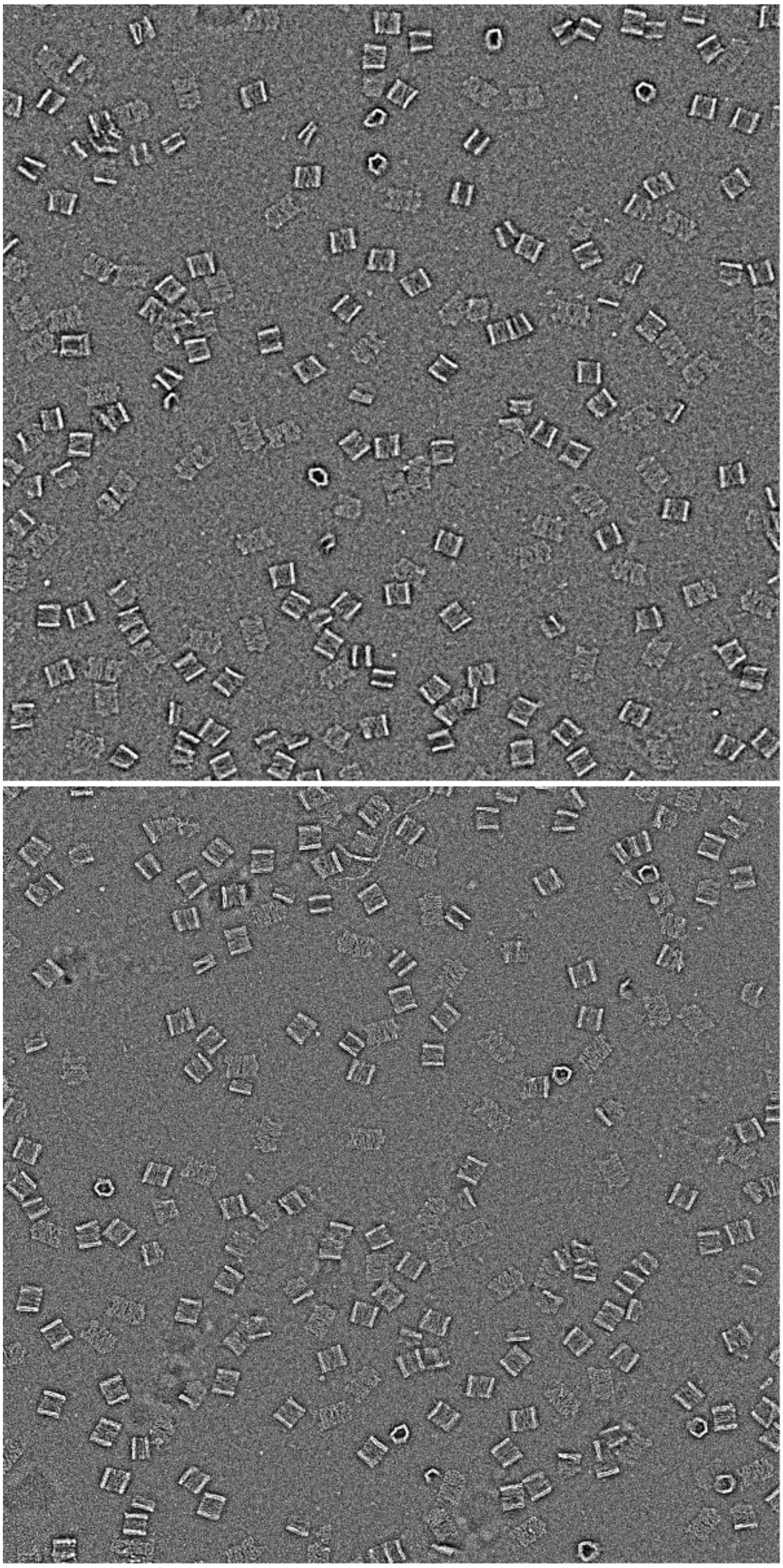

Source data for Suppl. Fig. 12

Wide-field TEM view of A^L

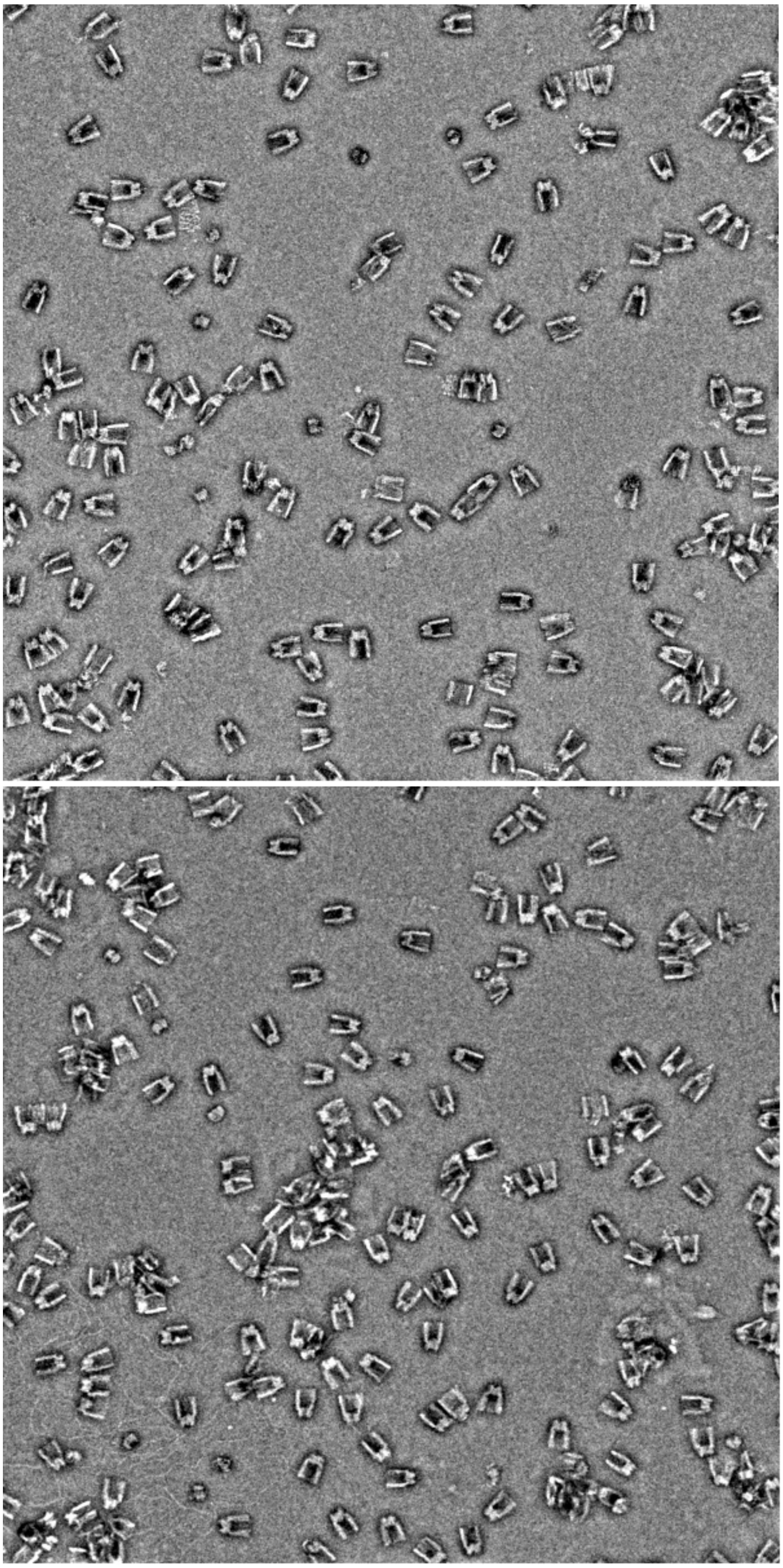

Source data for Suppl. Fig. 13

Wide-field TEM view of A^{2L}

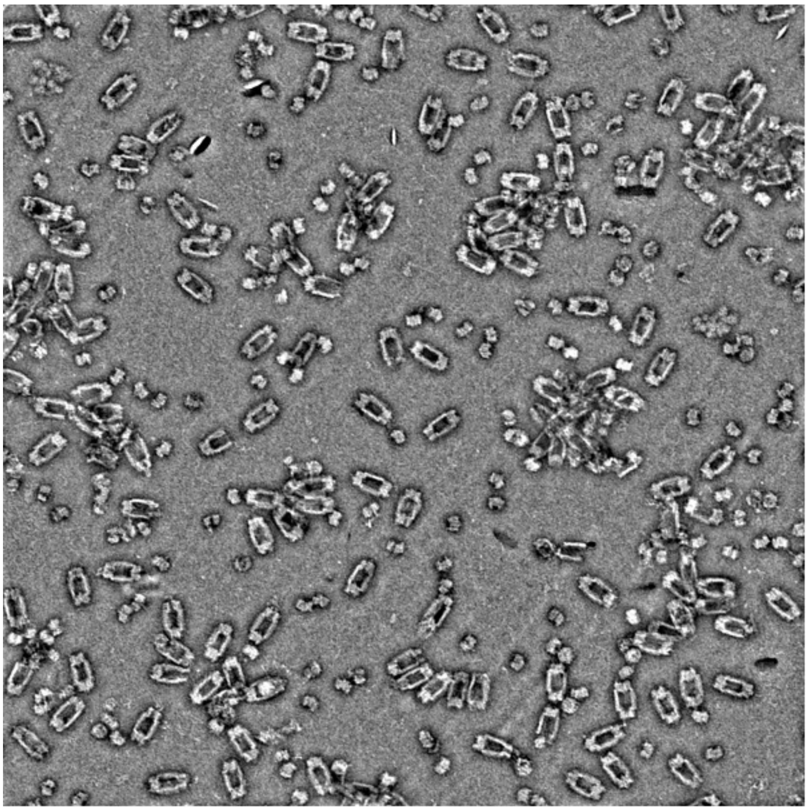

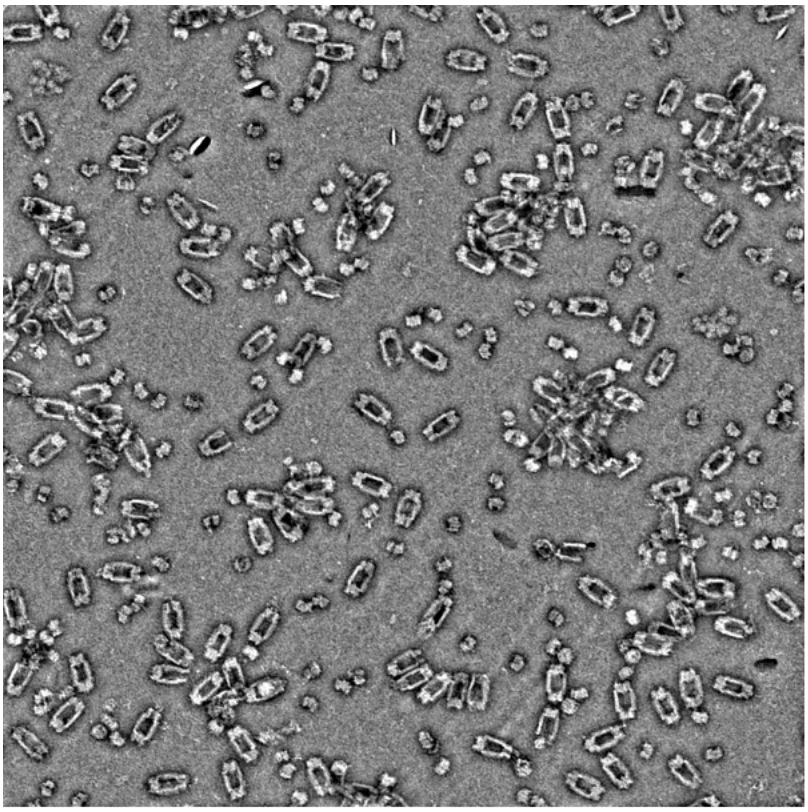

Source data for Suppl. Fig. 14

Uncropped gel B_{ex}

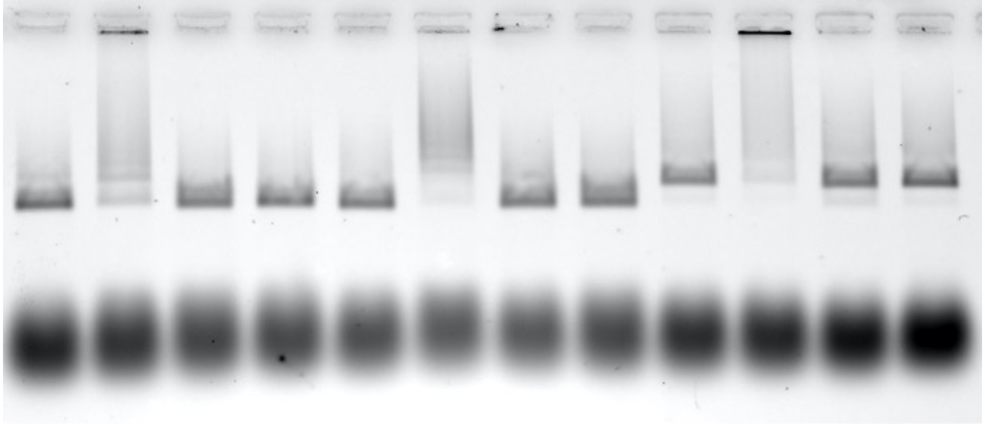

Uncropped gel B_{in}

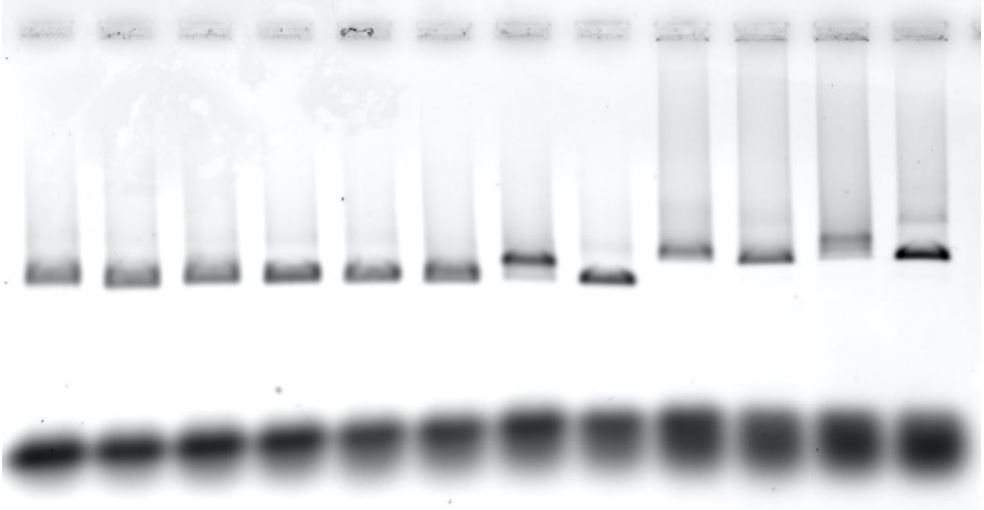

Uncropped gel B_{in+}

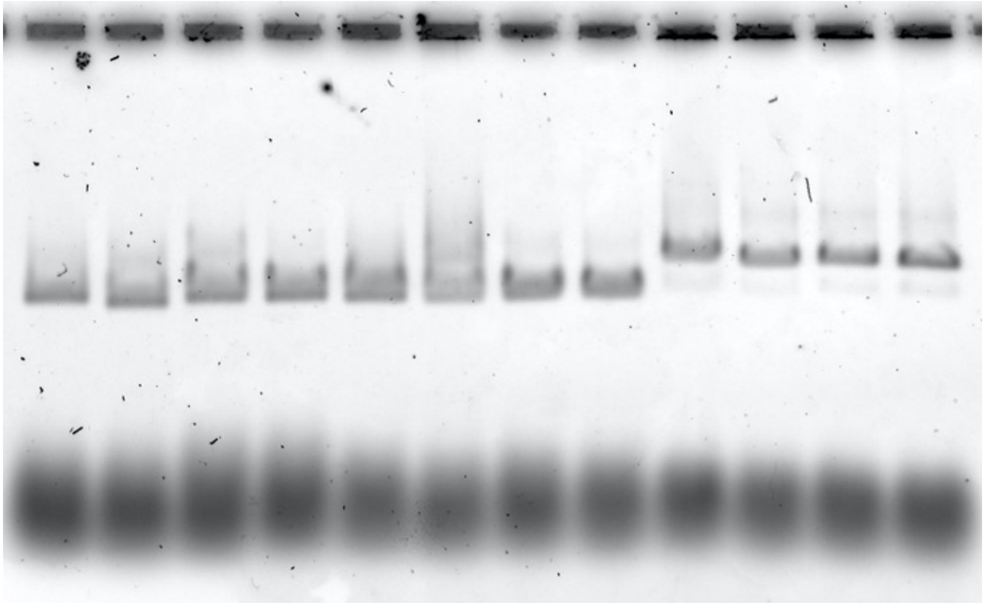

Source data for Suppl. Fig. 15

Wide-field TEM view of AB

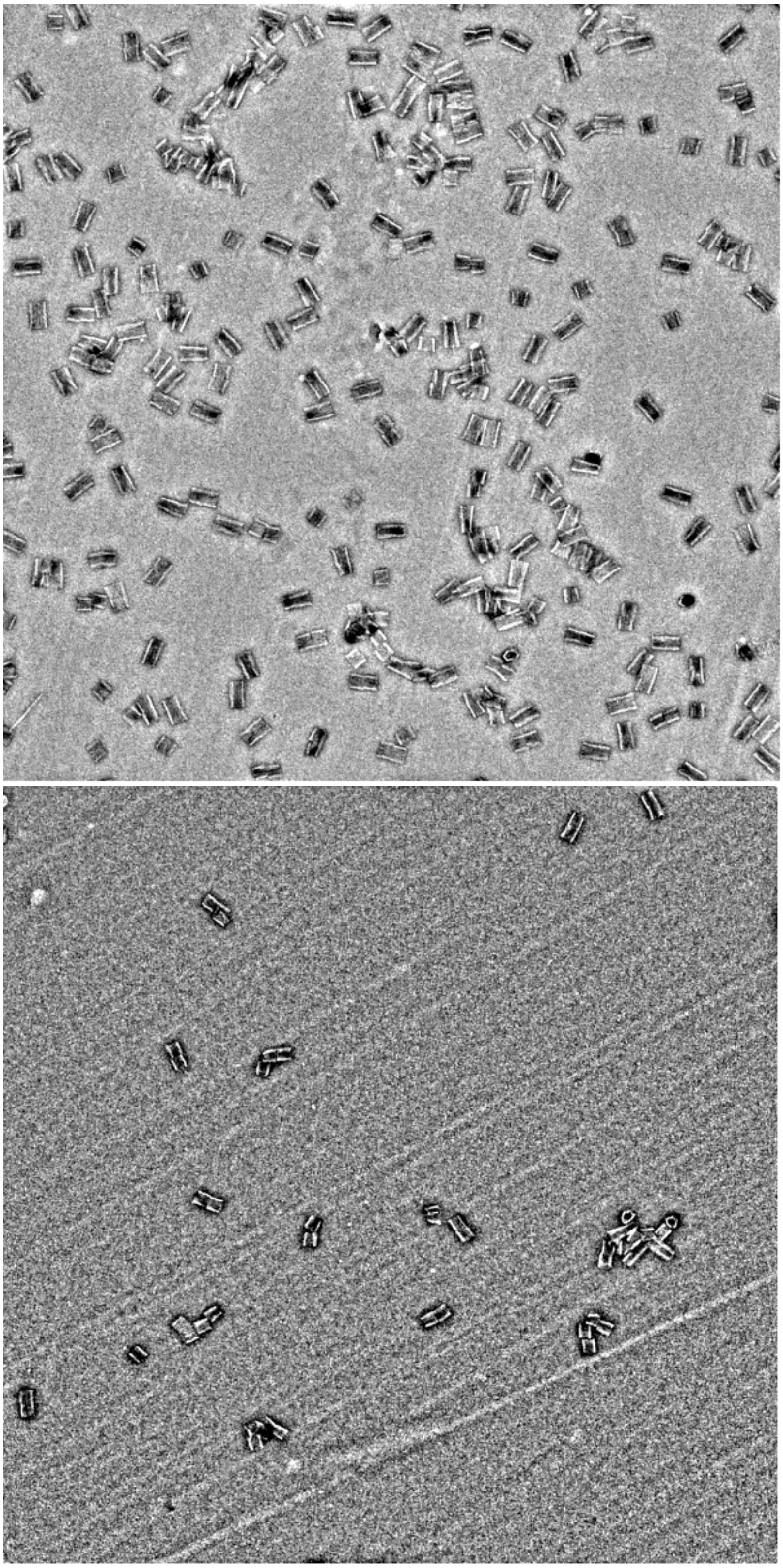

Source data for Suppl. Fig. 16

Wide-field TEM view of AB^L

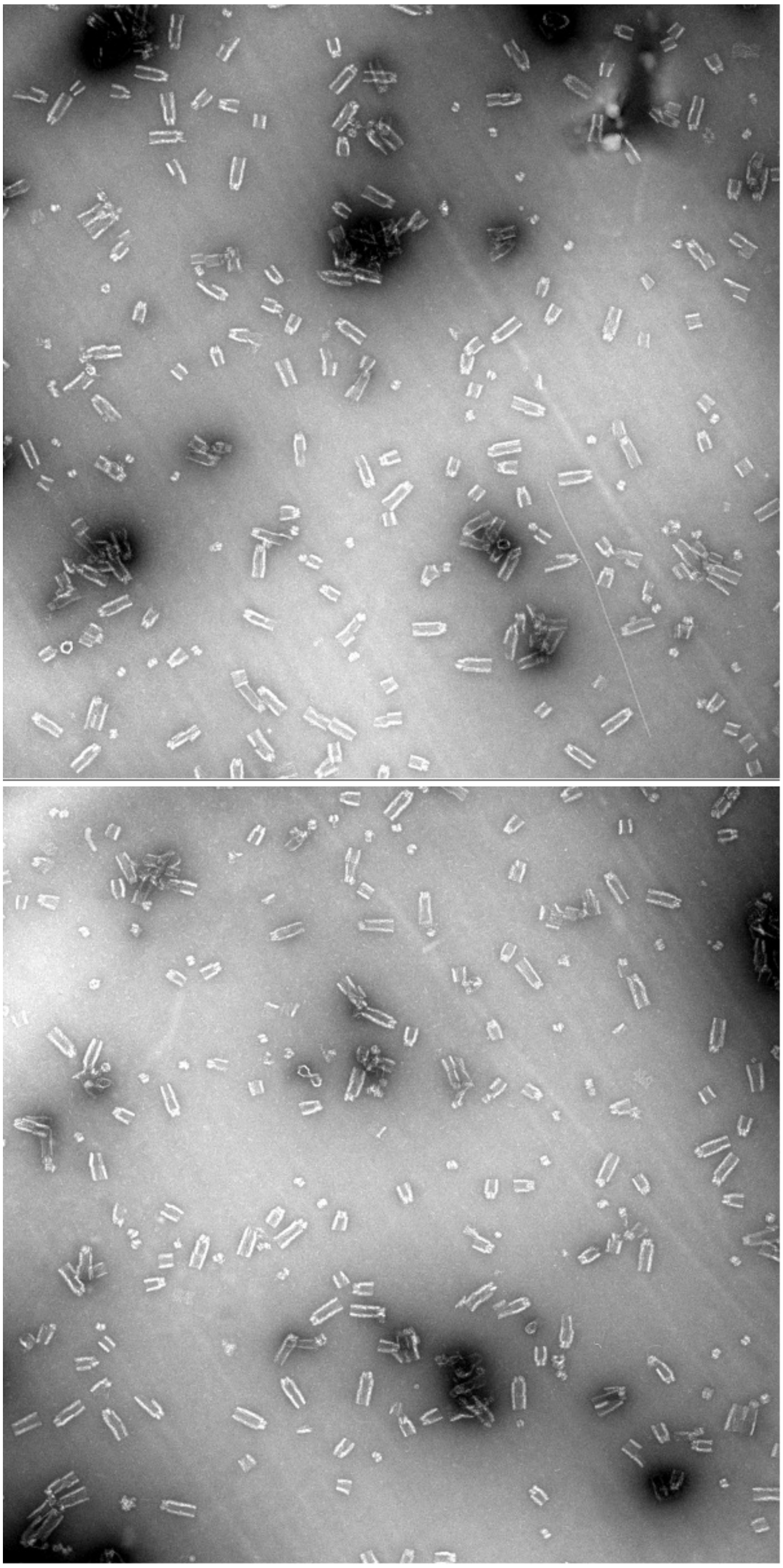

Source data for Suppl. Fig. 17

Uncropped gel 17a (lanes 1 to 7 were shown in the figure. The other lanes were controls.)

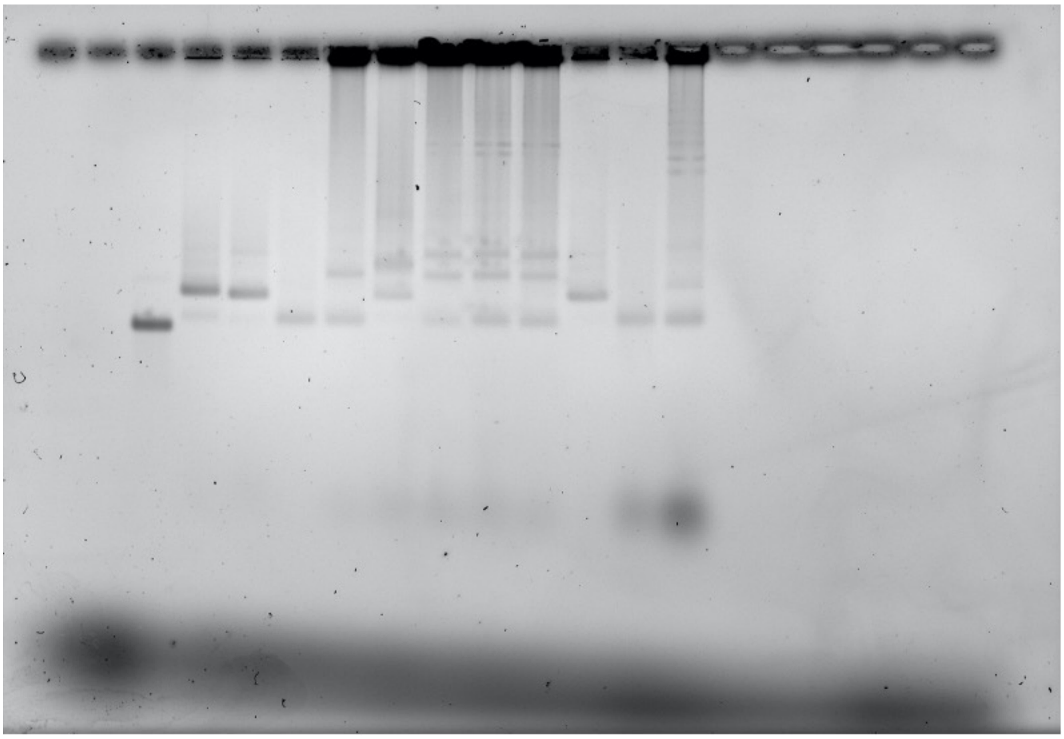

Uncropped gel 17b (last 8 lanes were shown in the figure. The other lanes were controls for reproducibility).

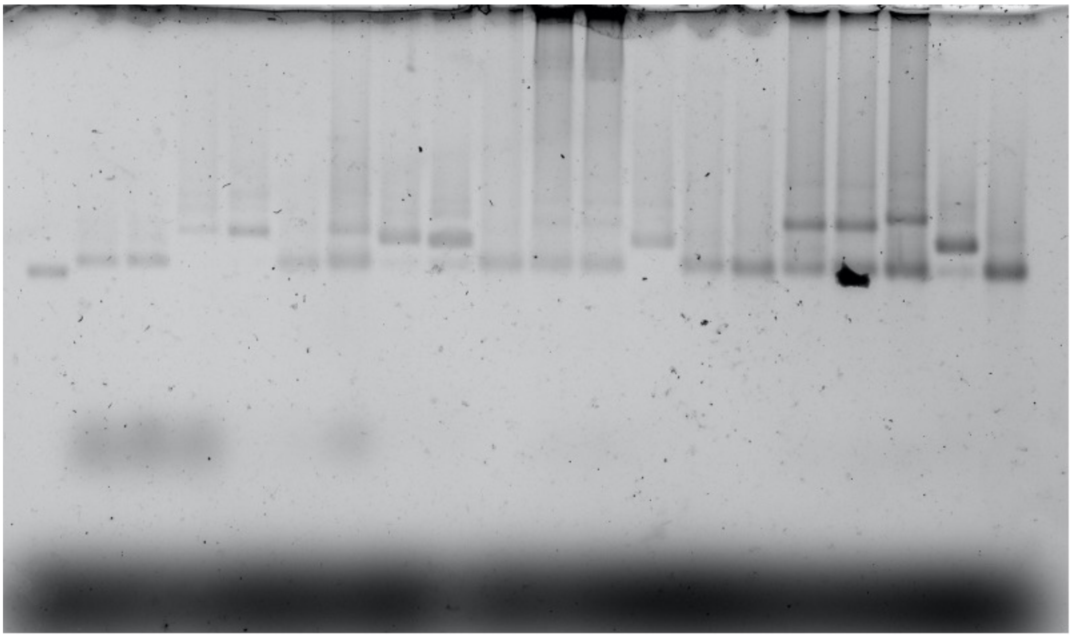

Source data for Suppl. Fig. 18

Uncropped gel 18c after FAM illumination

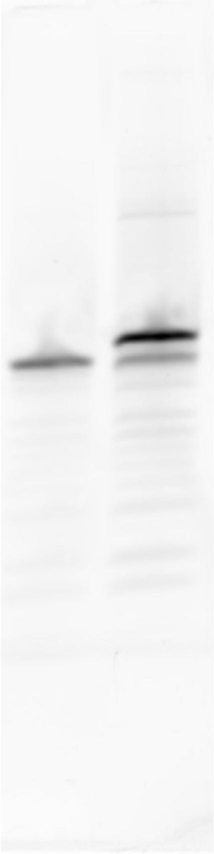

Uncropped gel 18e after Coomassie staining (left) and FAM illumination (right)

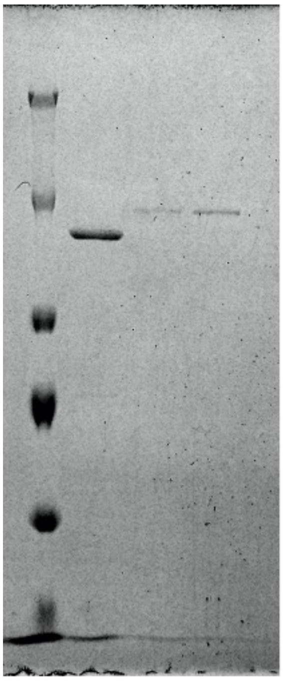

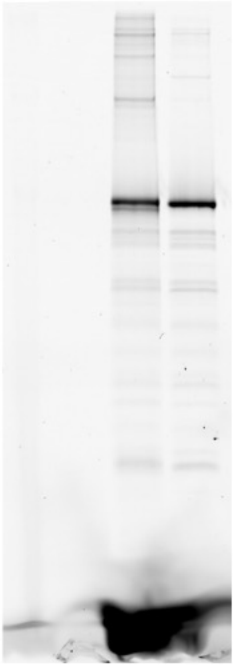

Source data for Suppl. Fig. 19

Uncropped gel 19a after Coomassie staining

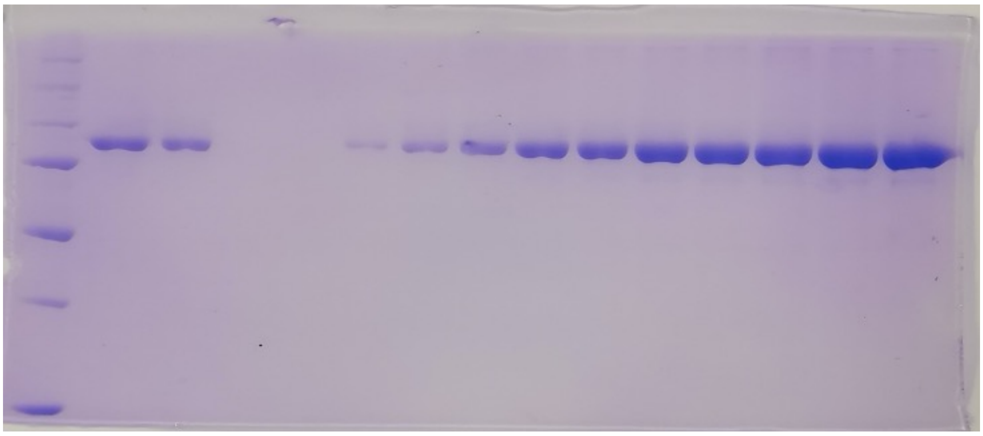

Same gel after FAM illumination

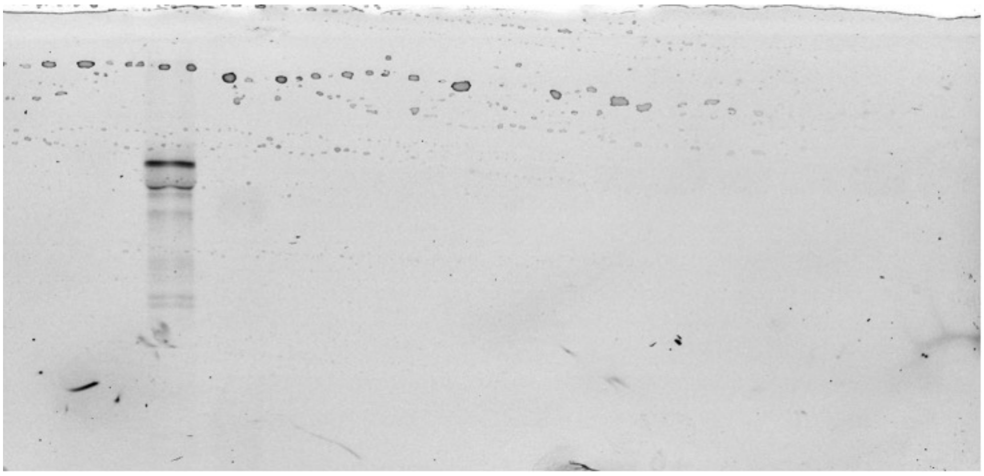

Source data for Suppl. Fig. 20

Uncropped gel 20 after Sybr green II staining

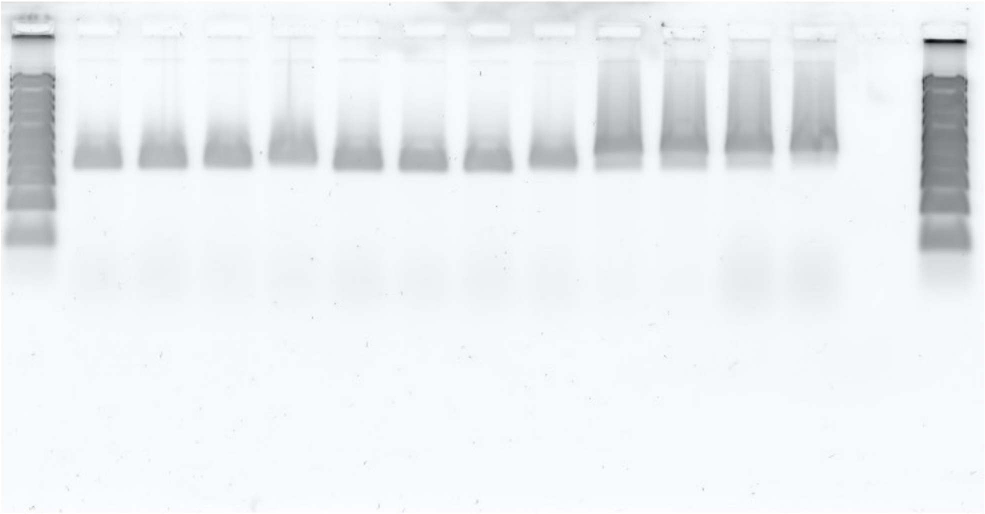

Same gel after TAMRA illumination

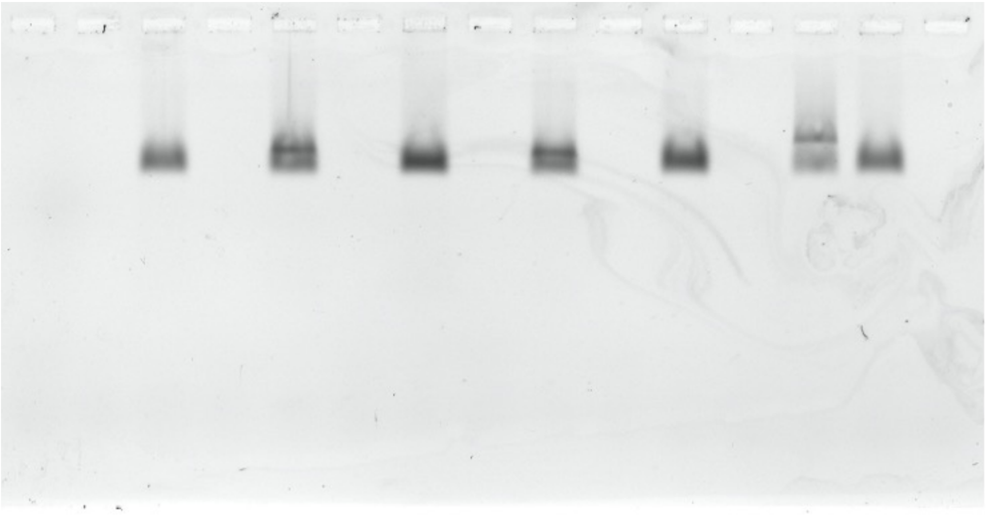

Source data for Suppl. Fig. 21

21b: Wide-field TEM view of A(p97)

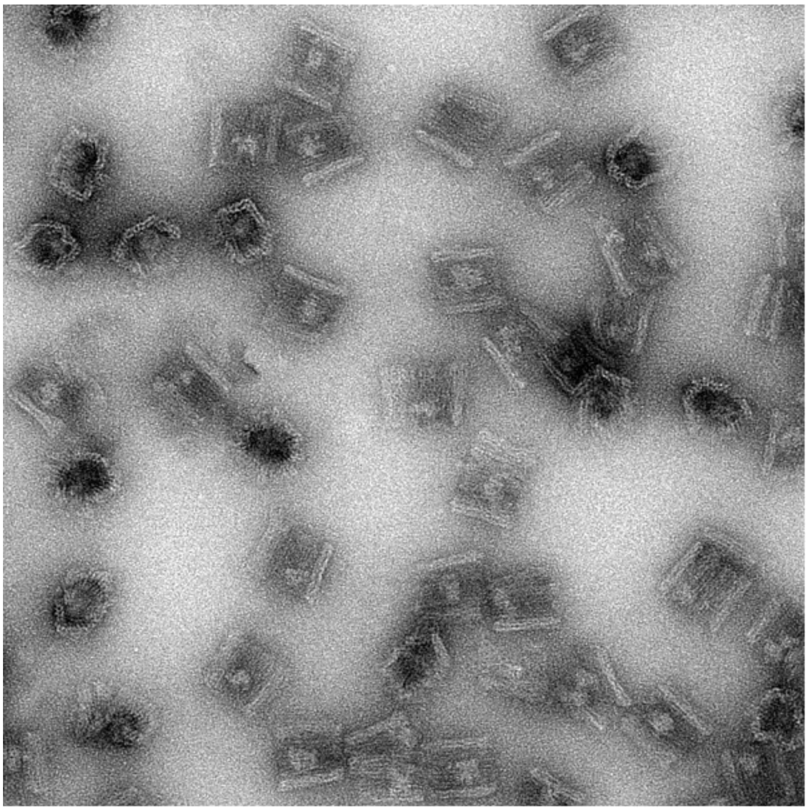

Source data for Suppl. Fig. 22

22b: Wide-field TEM view of A(p97)

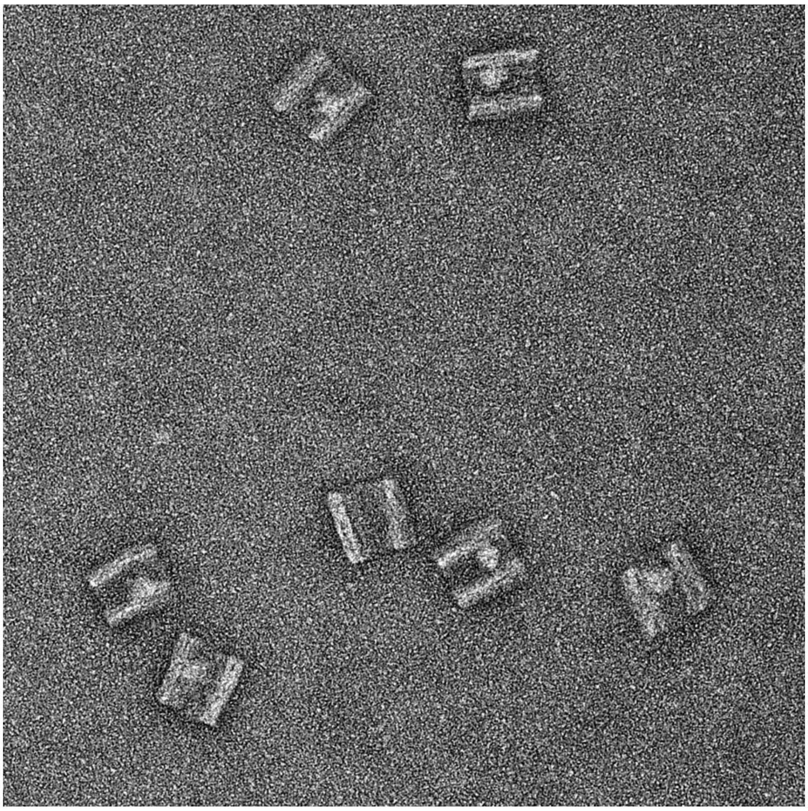

22c: Uncropped gel after ethidium bromide staining

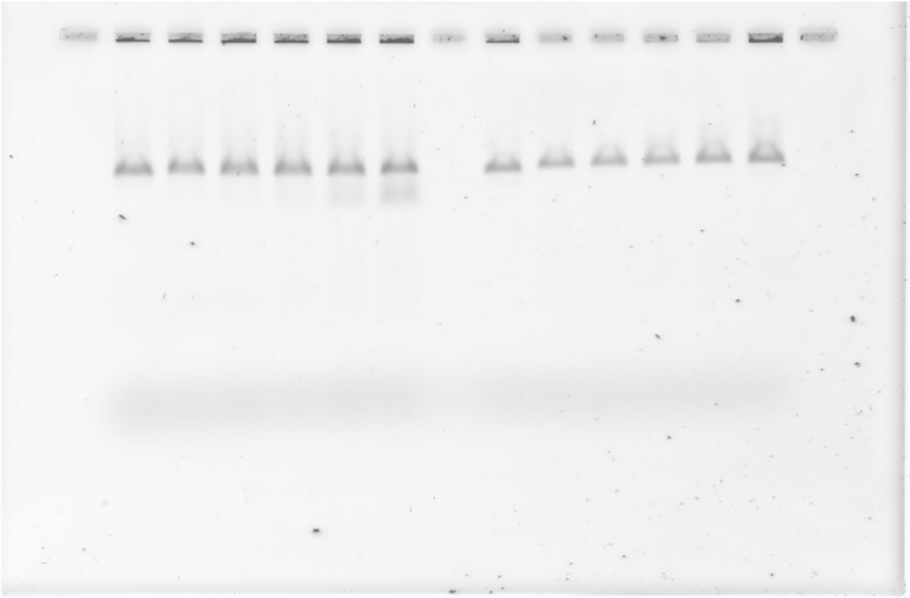

22c: same gel after FAM illumination

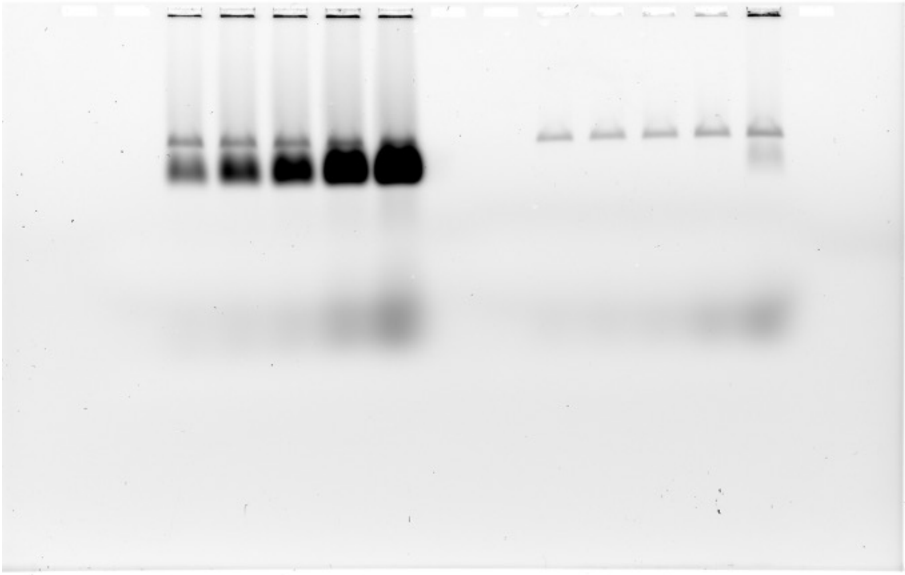

Source data for Suppl. Fig. 23

Wide-field TEM view of A(p97)

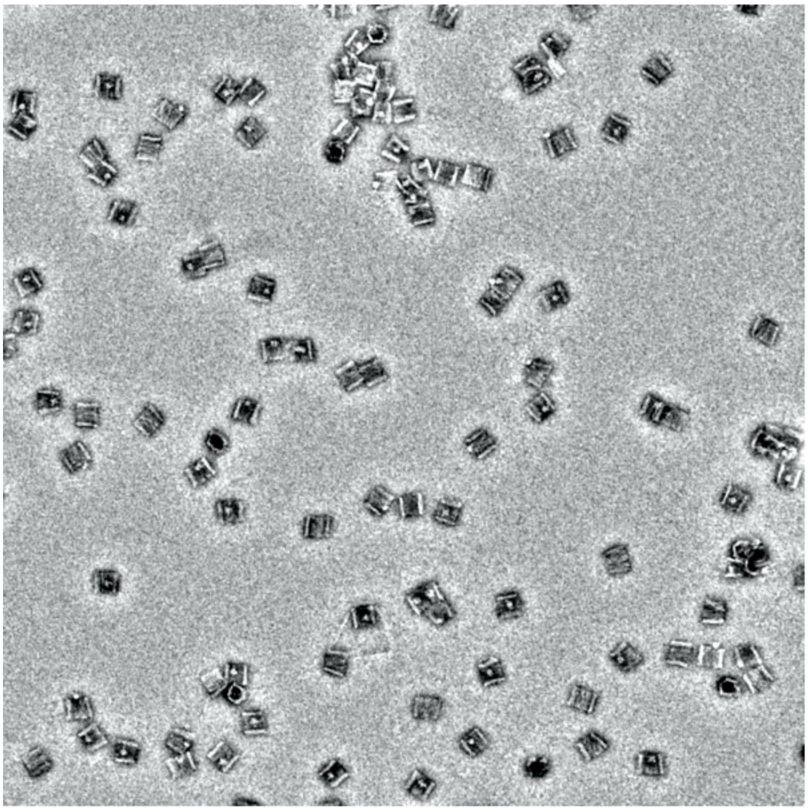

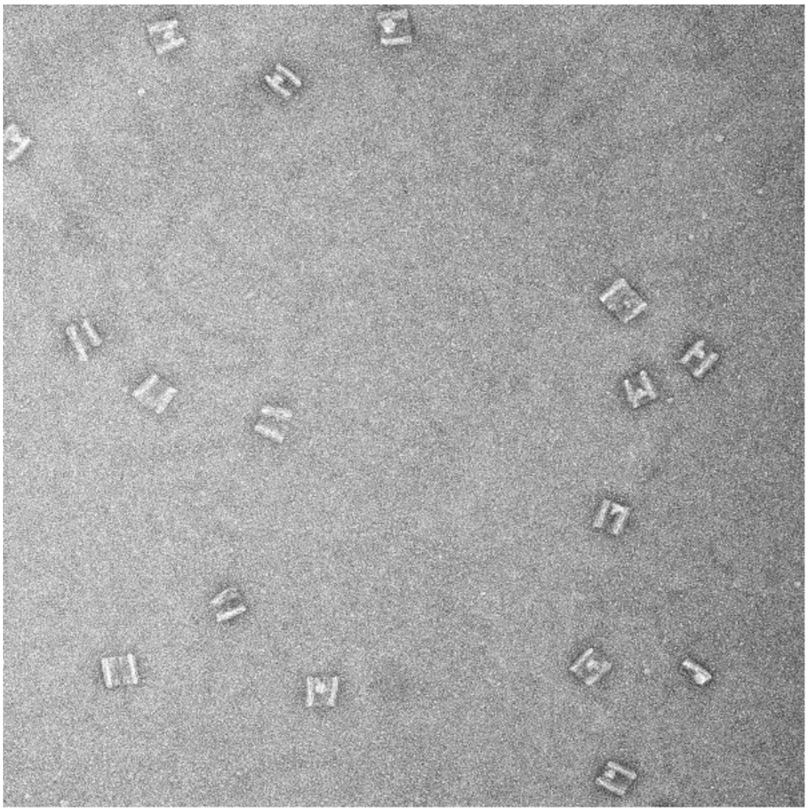

Source data for Suppl. Fig. 24

24a

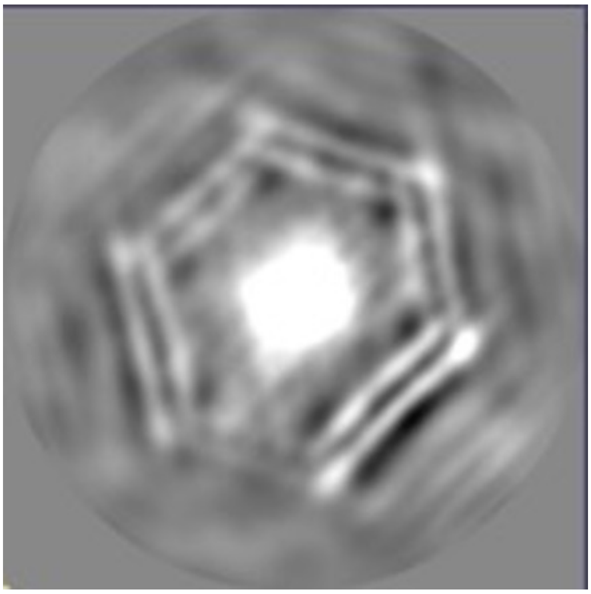

24b

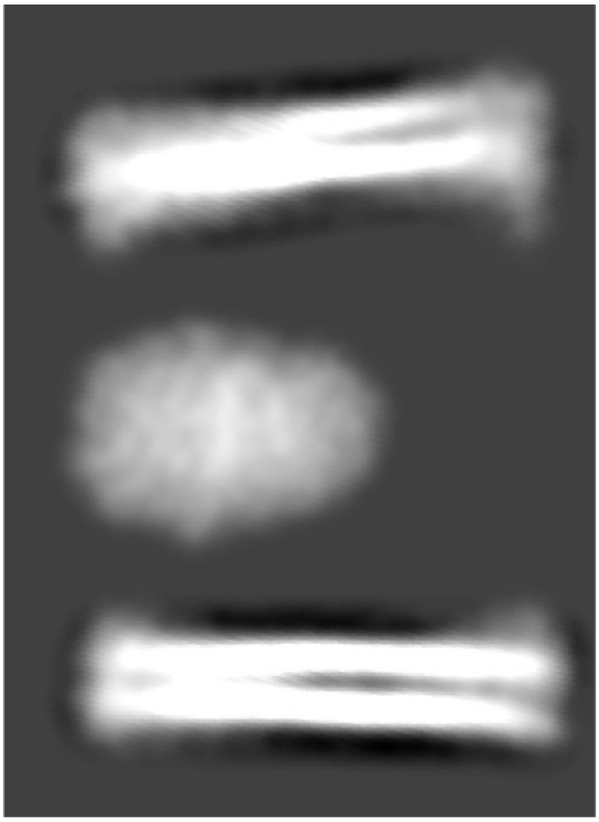

24d

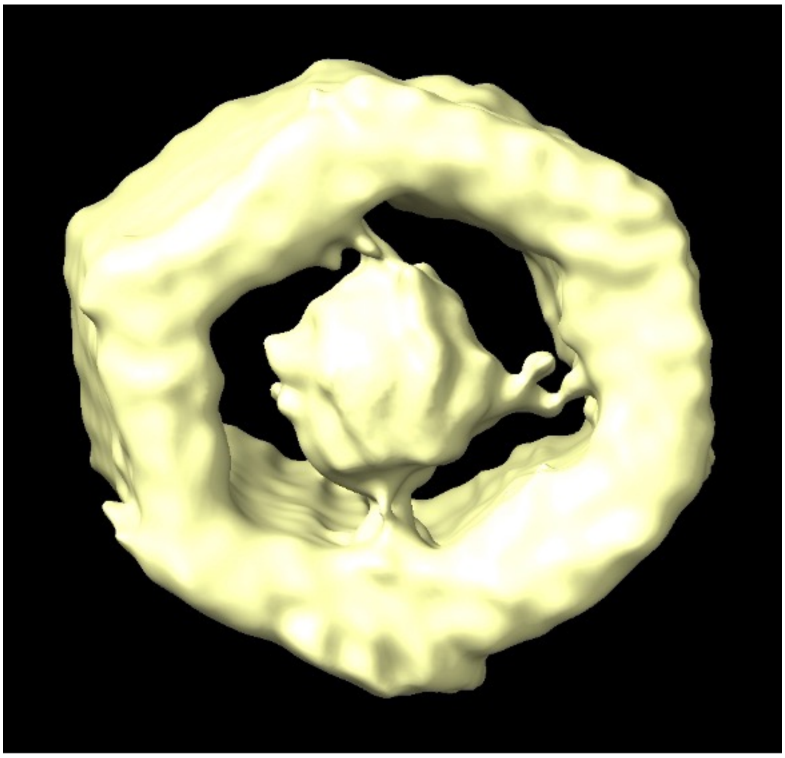

24e

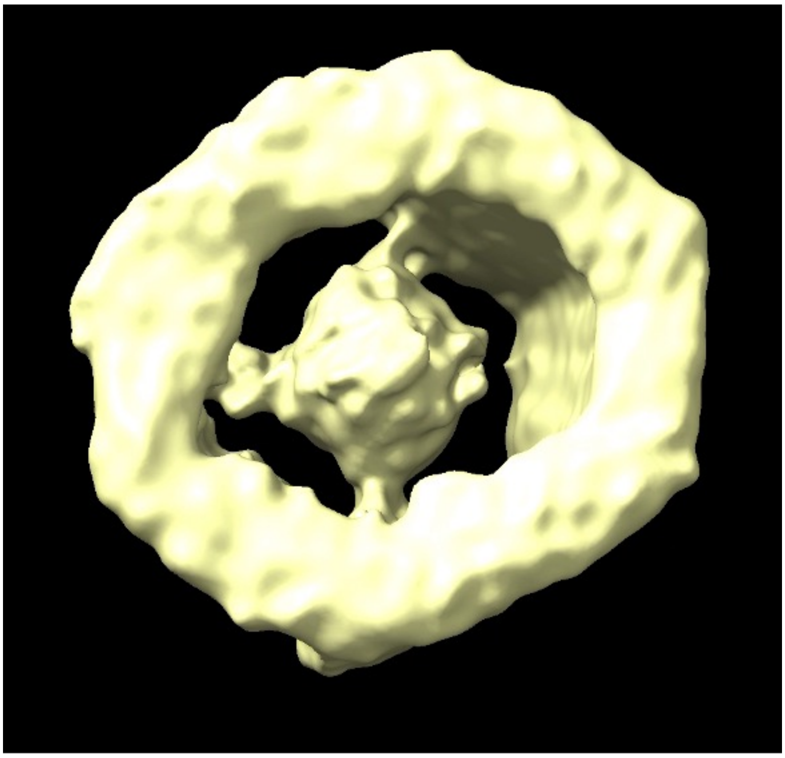

Source data for Suppl. Fig. 25

25b: Uncropped gel after ethidium bromide staining

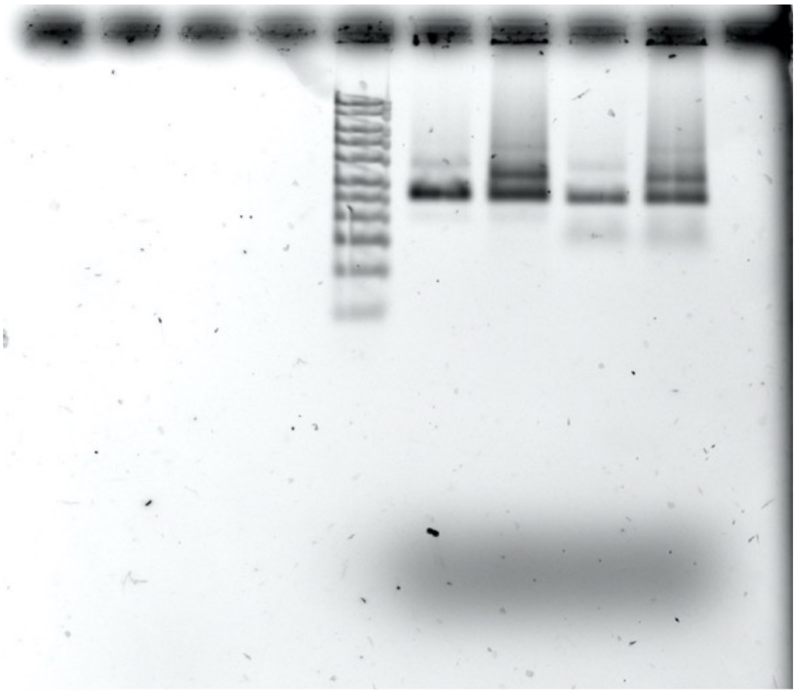

25b: same gel after TAMRA illumination

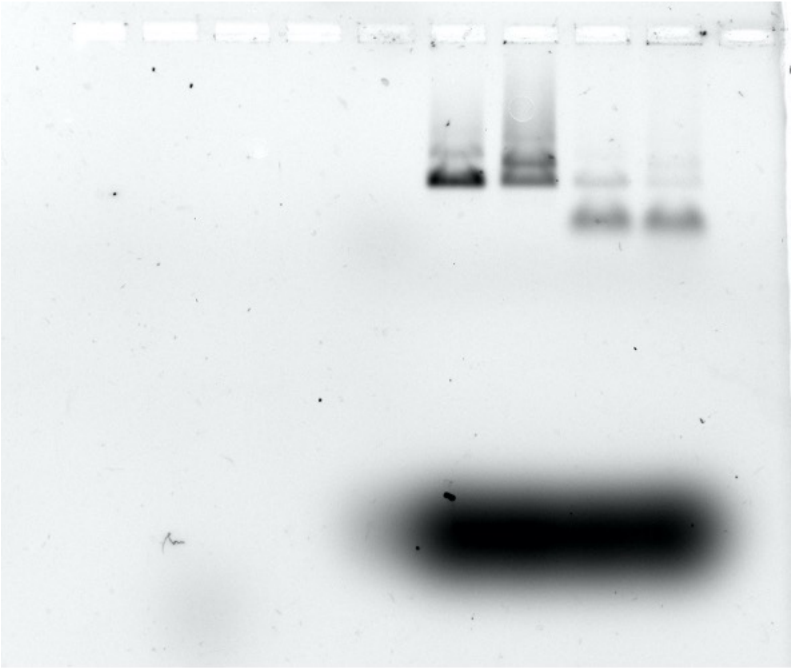

Source data for Suppl. Fig. 27

27a (left panel): Uncropped gel after ethidium bromide staining

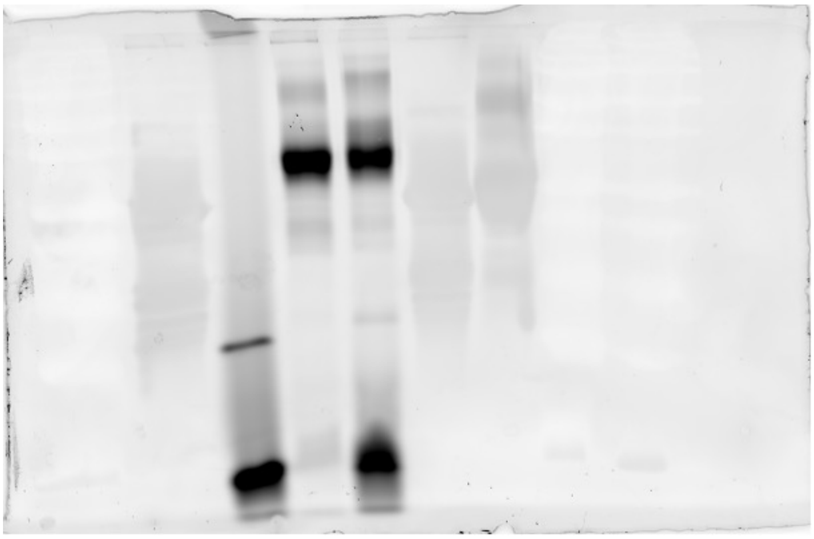

27a (right panel): same gel after FAM illumination

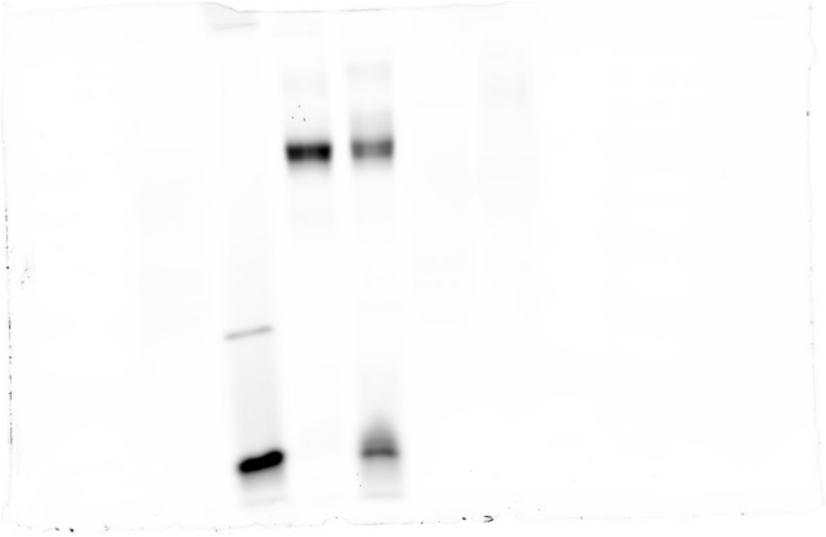

27b: same gel after Coomassie staining

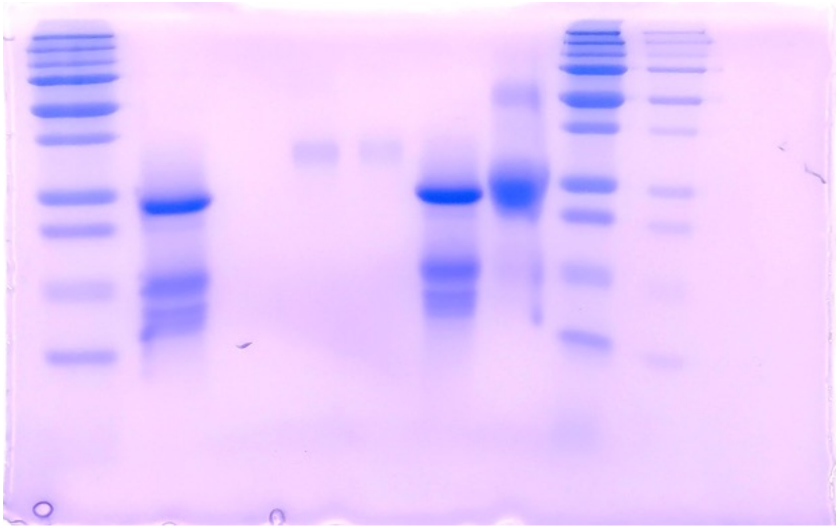

27d: uncropped gel under FAM illumination

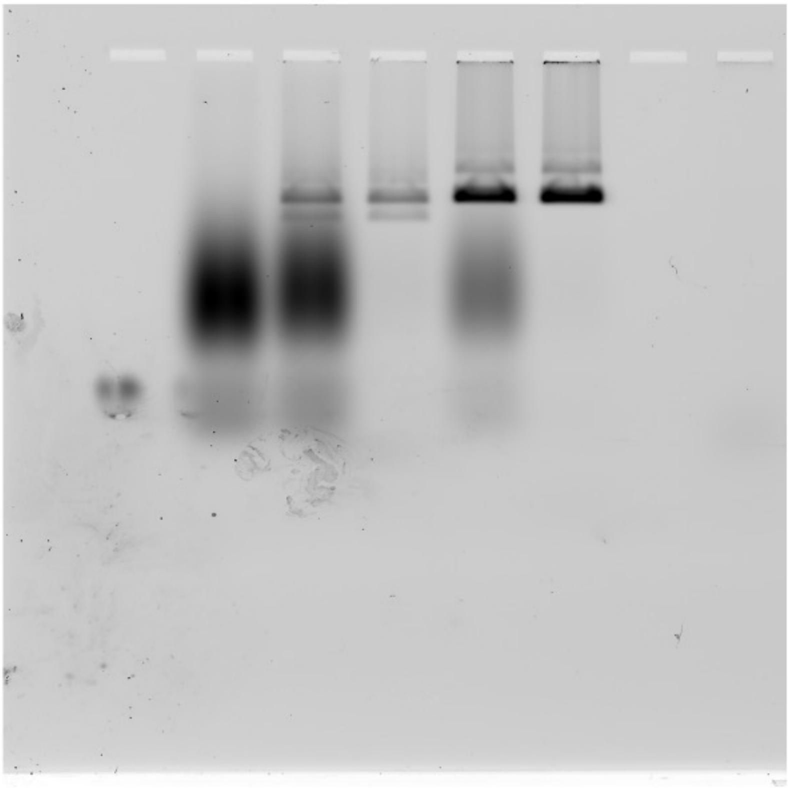

27d: same gel after ethidium bromide staining

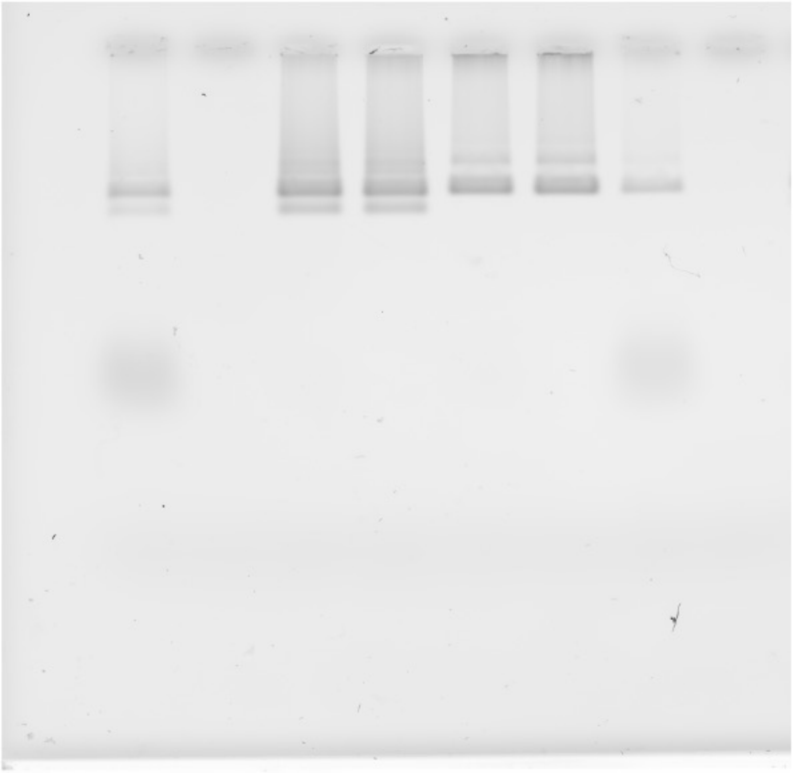

27e: wide-field TEM view and class averages of B(aCt)

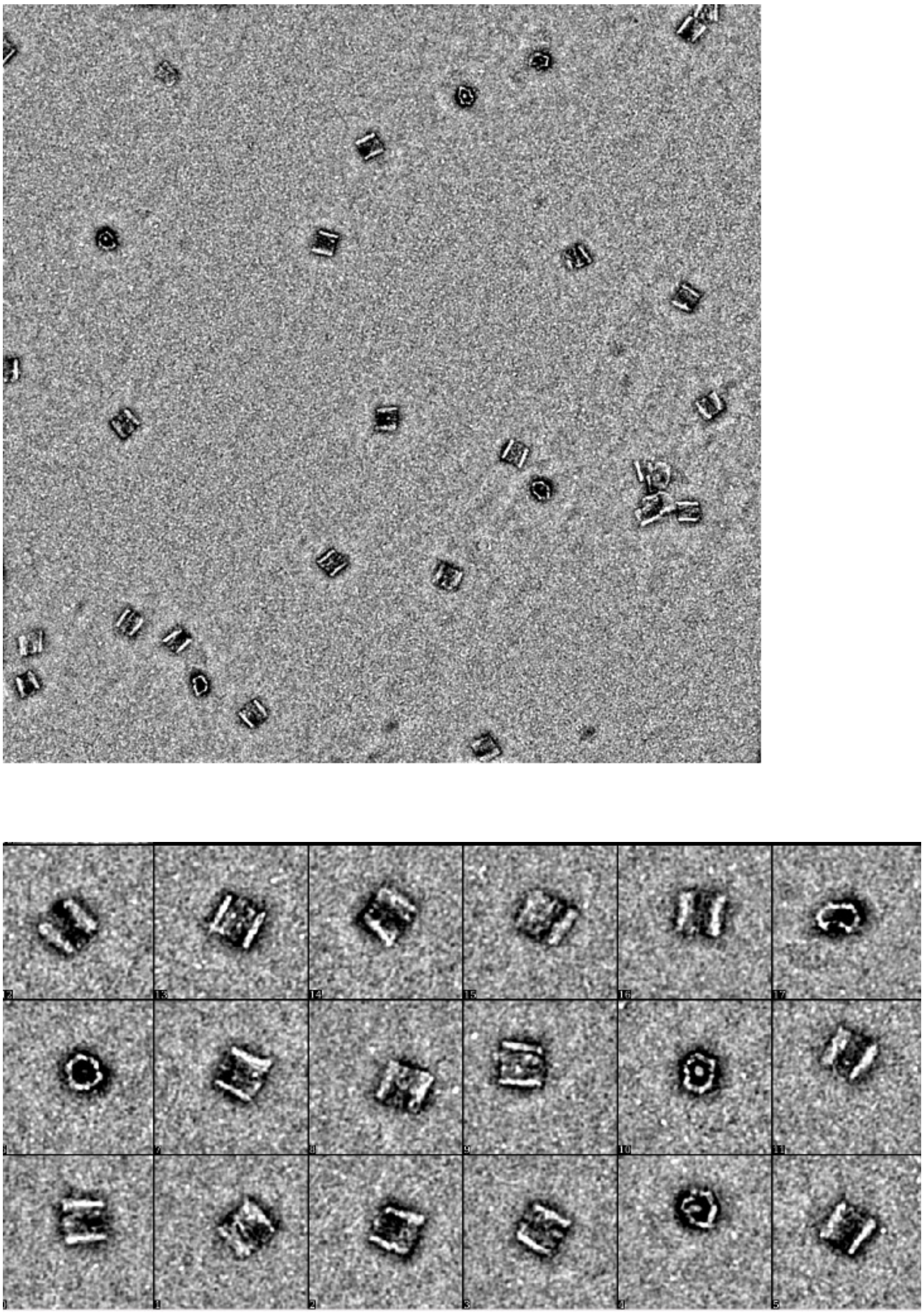

Source data for Suppl. Fig. 28

28b: Uncropped gel after FAM illumination

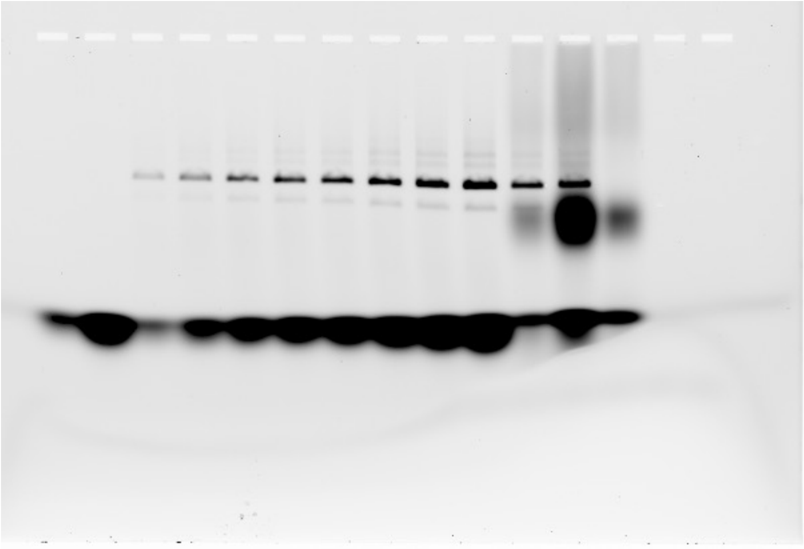

28b: same gel after ethidium bromide staining

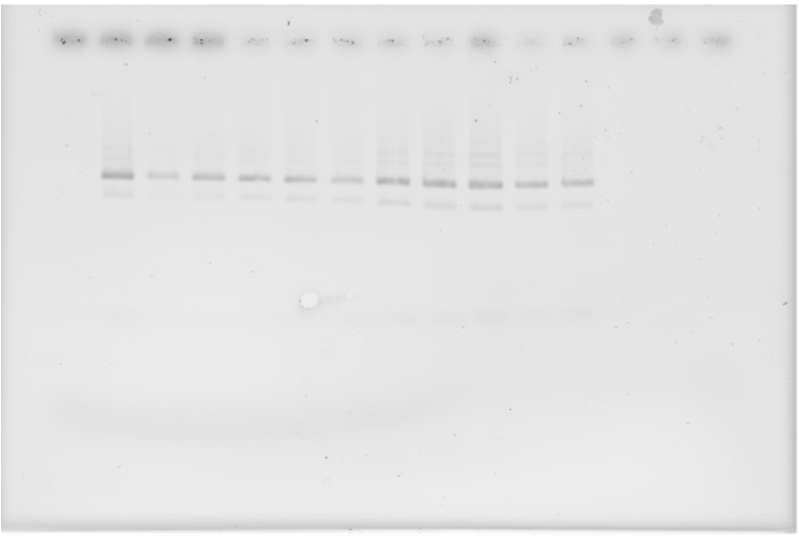

Source data for Suppl. Fig. 29

29b: Uncropped gel after FAM illumination

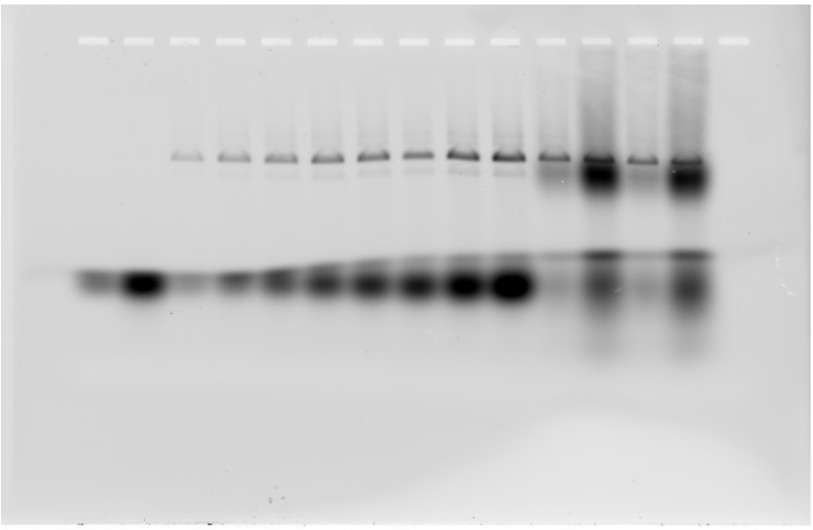

29b: Same gel after ethidium bromide staining

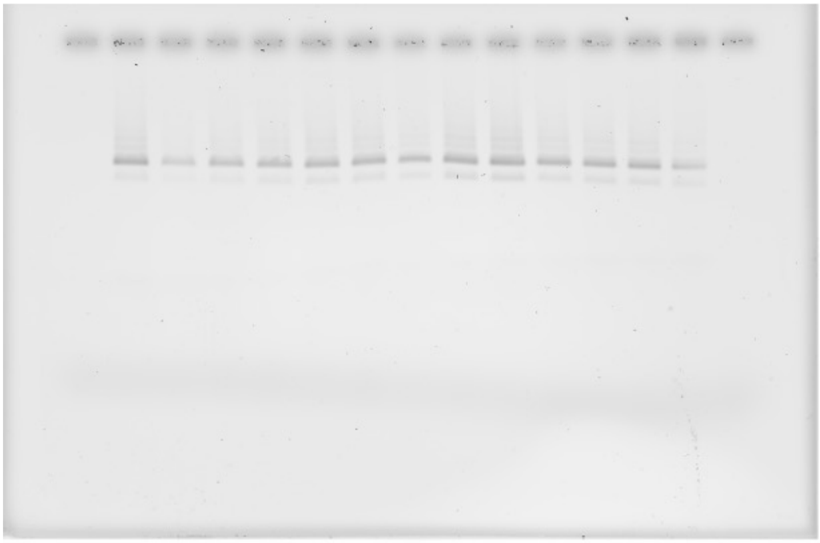

Source data for Suppl. Fig. 30

Uncropped gel after ethidium bromide staining

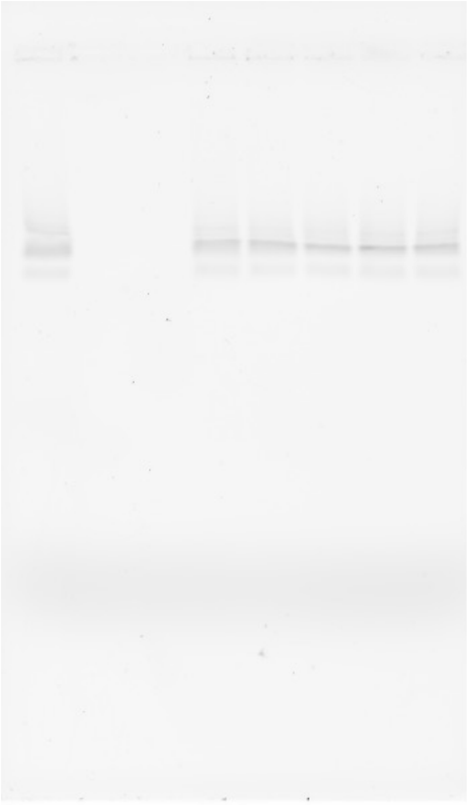

Same gel after Cy5 illumination

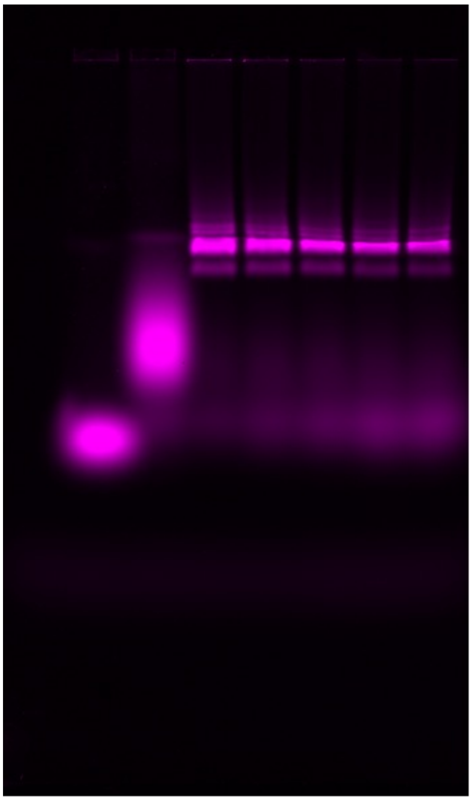

Source data for Suppl. Fig. 34

34a (10 min): Uncropped gel after Coomassie staining

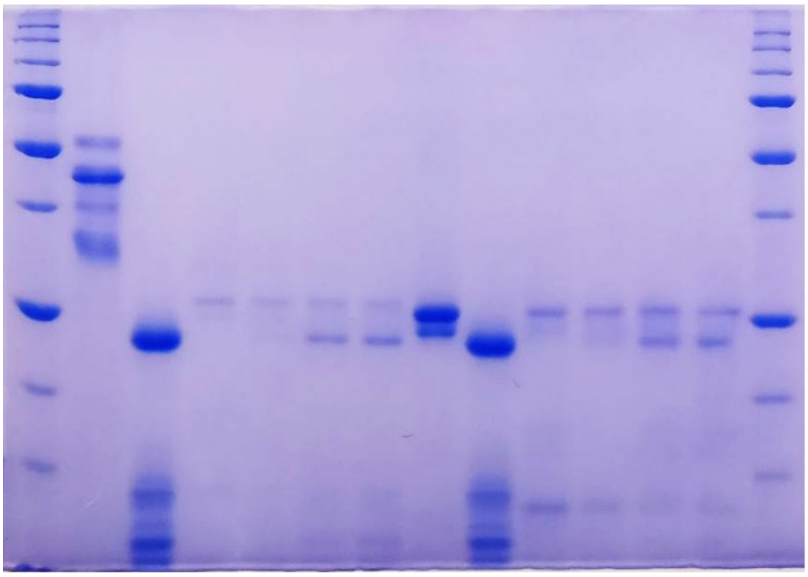

34a (10 min): same gel after FAM illumination

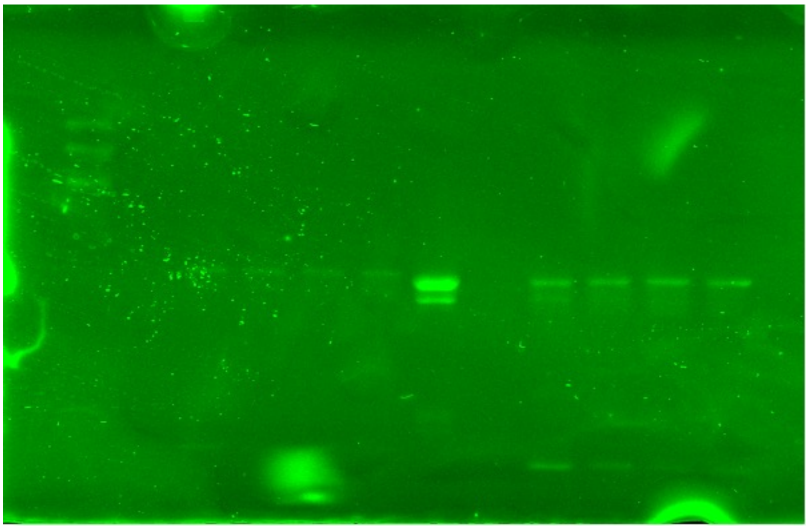

34a (3 h): uncropped gel after Coomassie staining

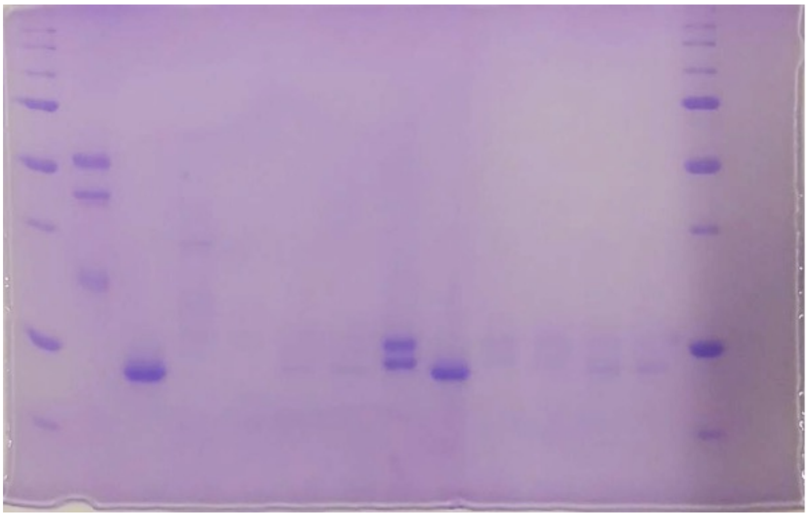

34a (3 h): same gel after FAM illumination

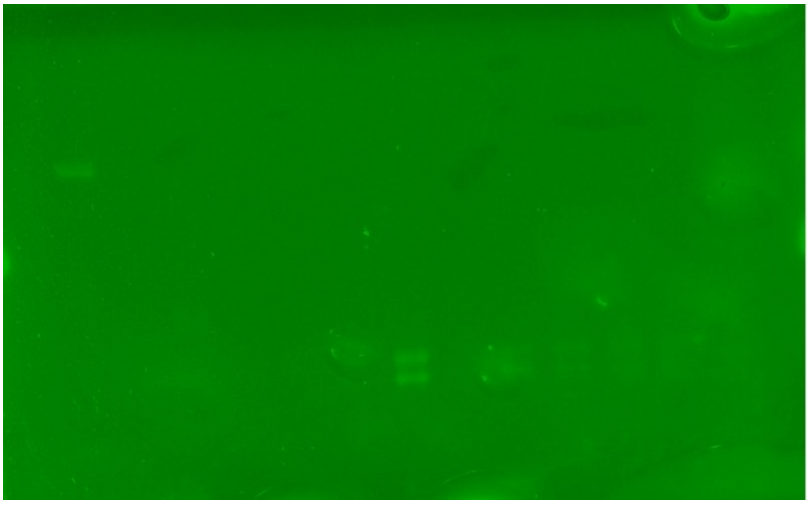

34b (10 min): Uncropped gel after Coomassie staining

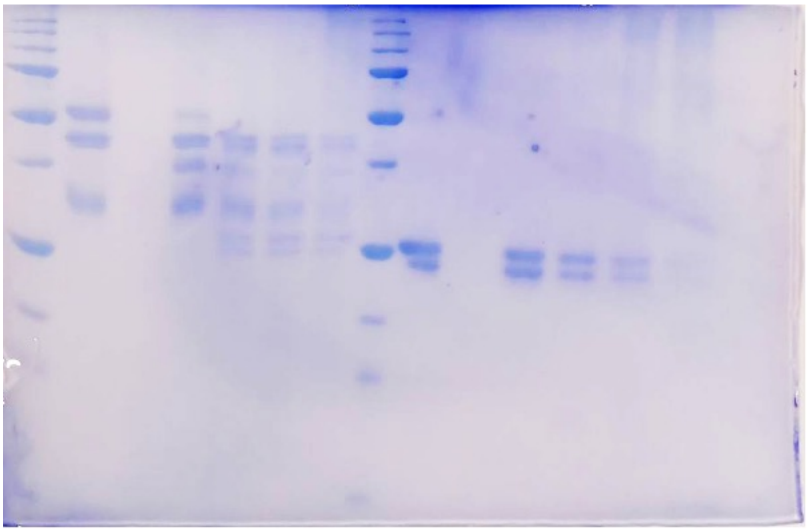

34b (10 min): same gel after FAM illumination

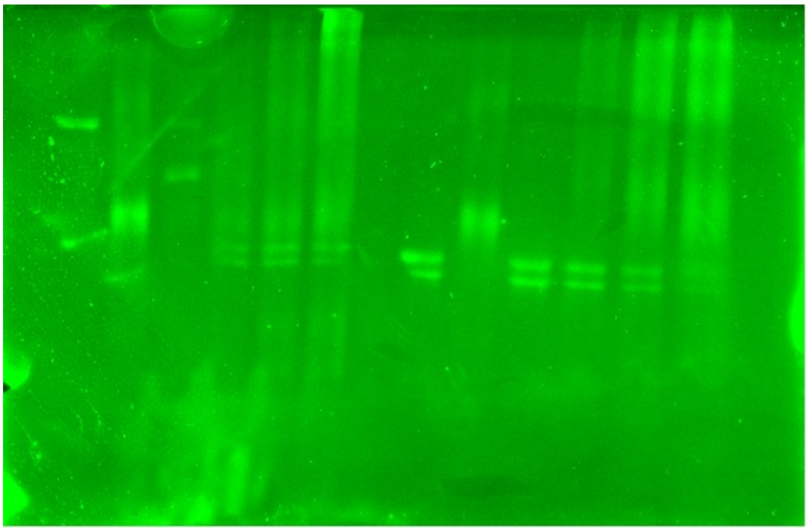

34b (10 min): same gel after Cy5 illumination

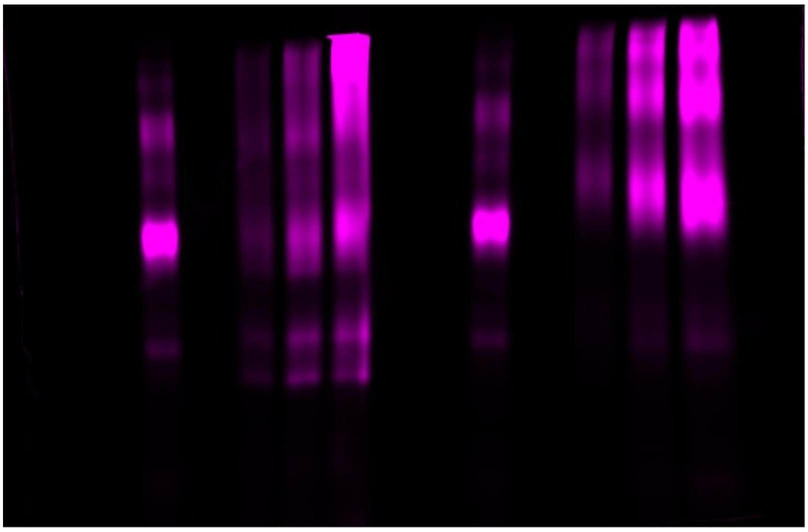

34b (3 h): Uncropped gel after Coomassie staining

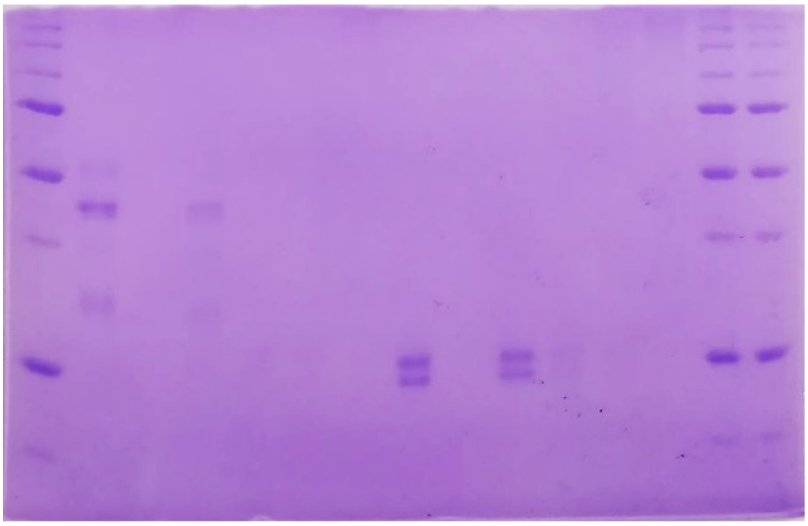

34b (3 h): same gel after FAM illumination

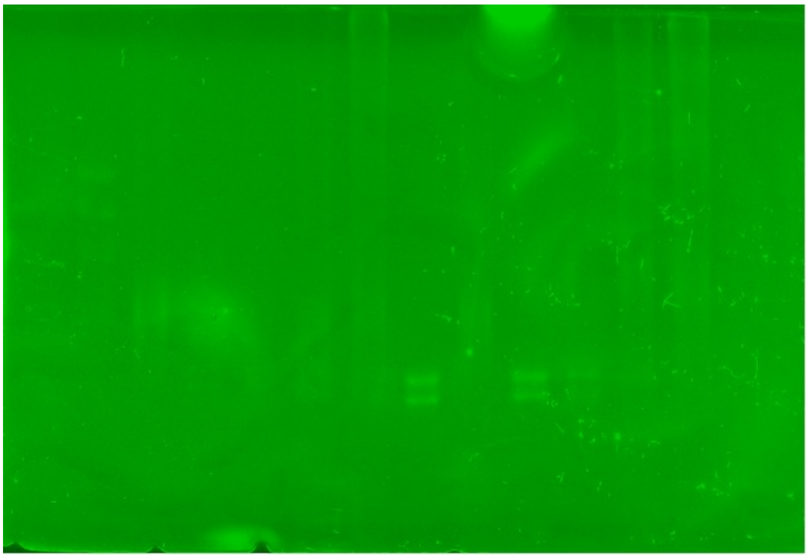

34b (3 h): same gel after Cy5 illumination

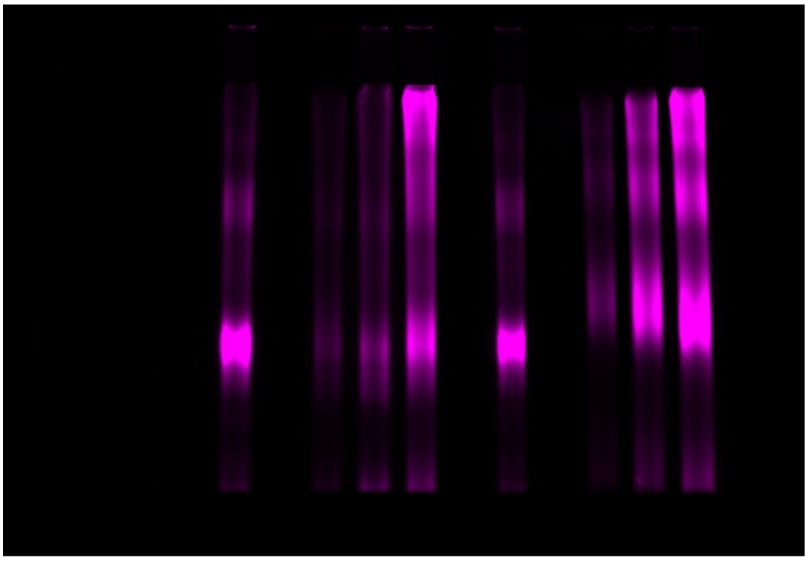

34c (10 min): Uncropped gel after Coomassie staining

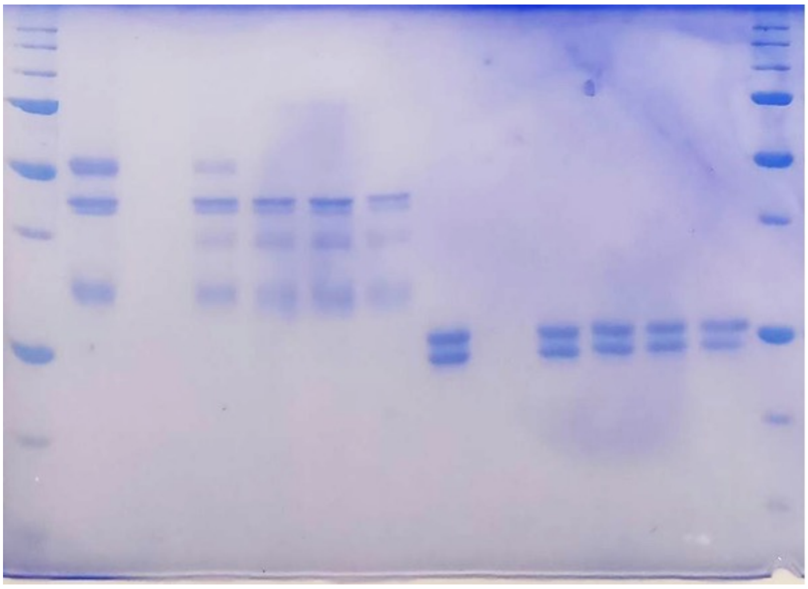

34c (10 min): same gel after FAM illumination

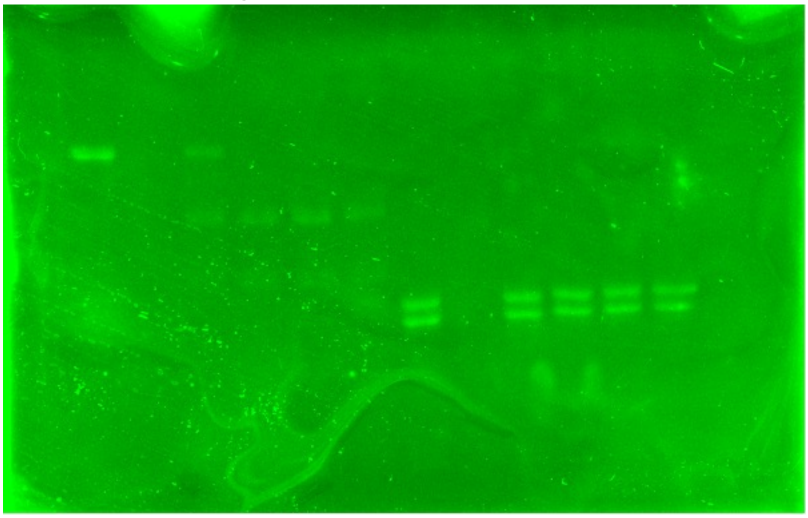

34c (3 h): Uncropped gel after Coomassie staining

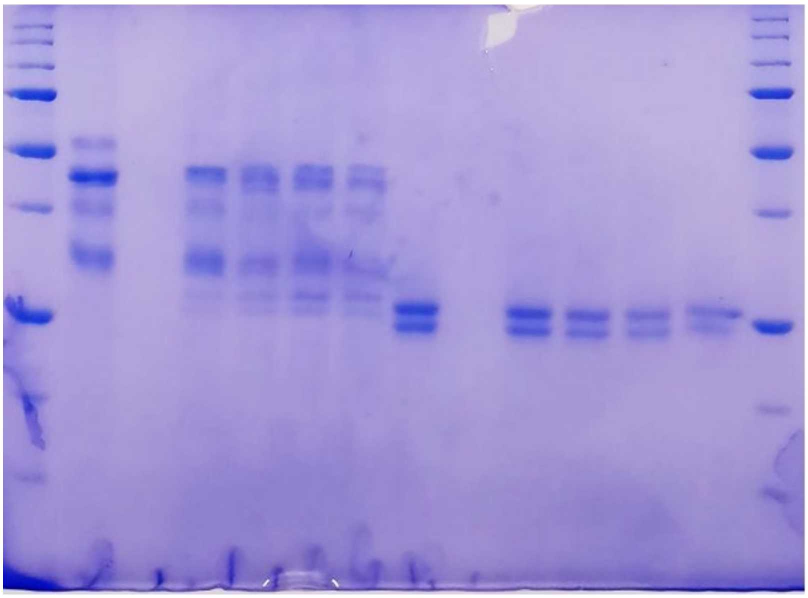

34c (3 h): same gel after FAM illumination

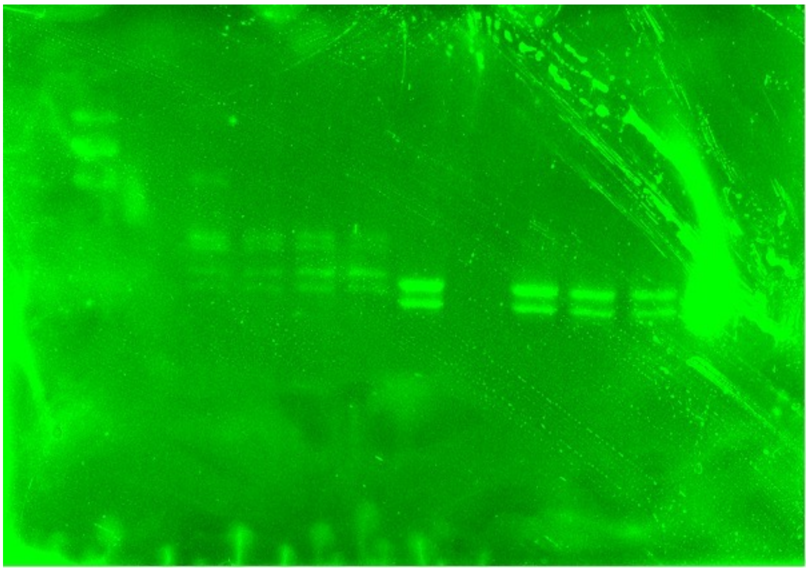

Source data for Suppl. Fig. 35

35a (from left to right)

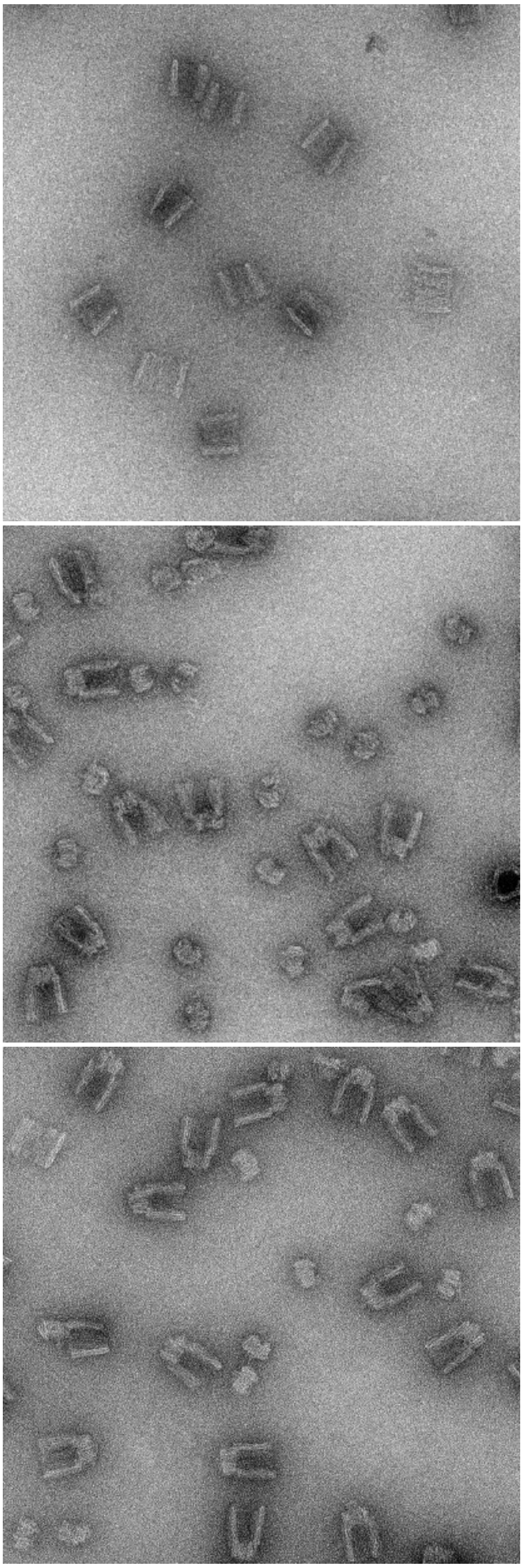

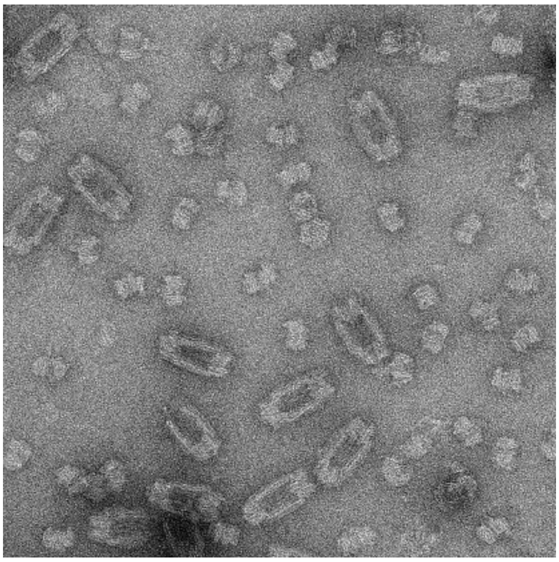

35b (from left to right)

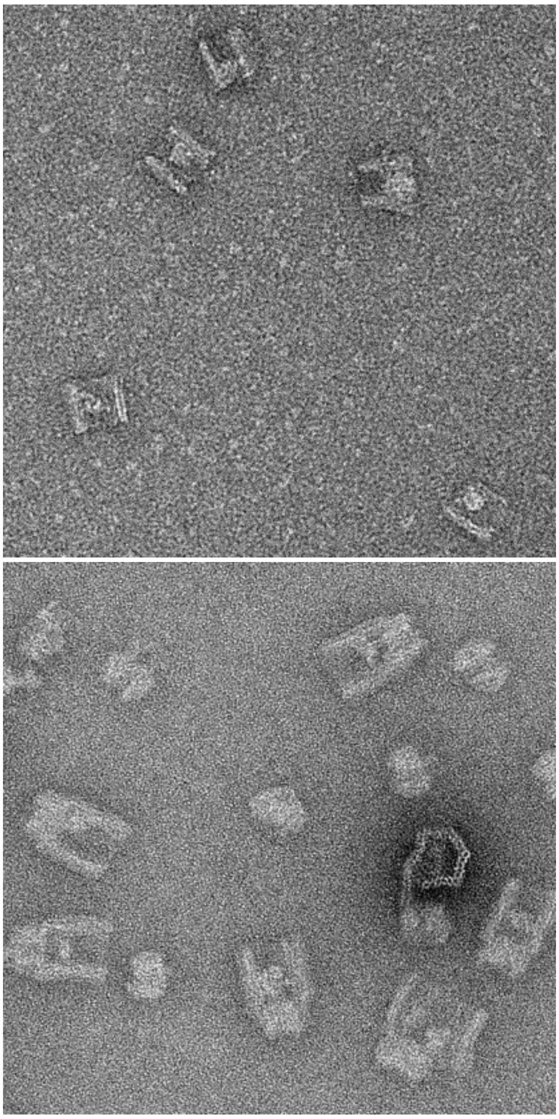

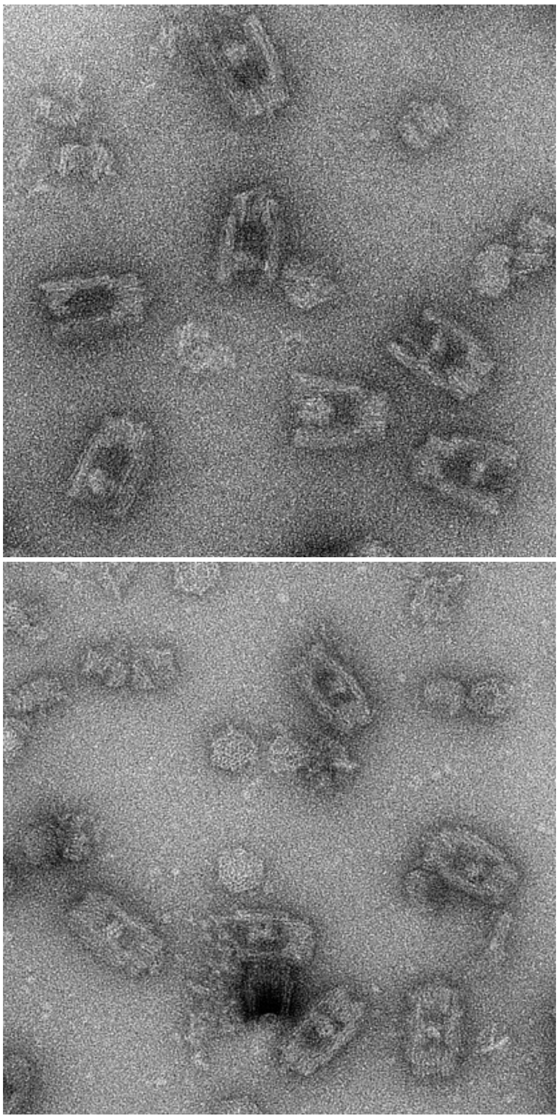

Source data for Suppl. Fig. 36

Uncropped gel after ethidium bromide staining

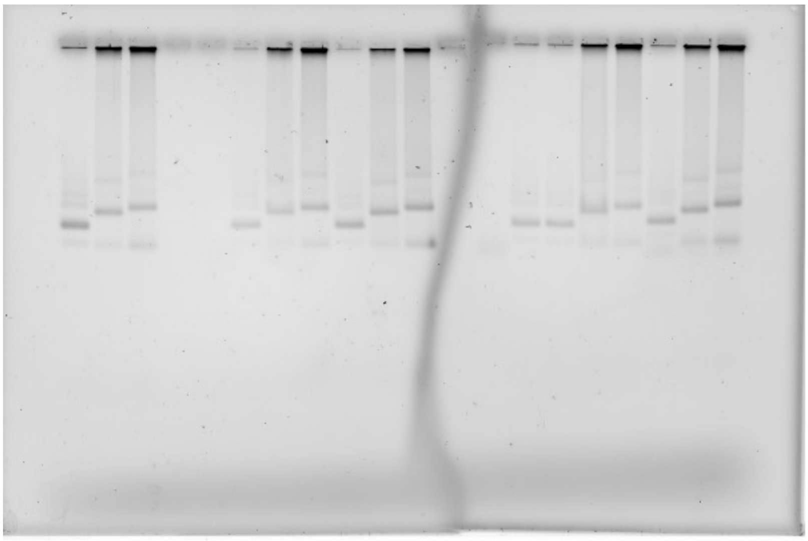

Same gel after FAM illumination

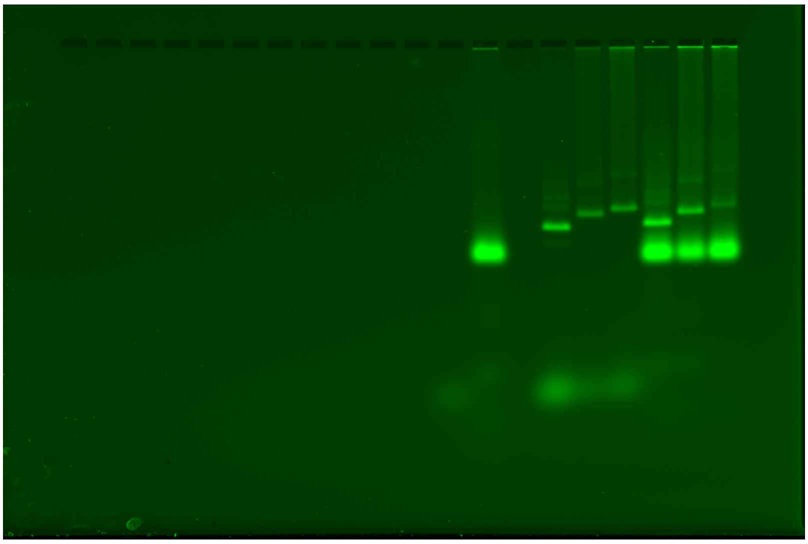

Same gel after Cy5 illumination

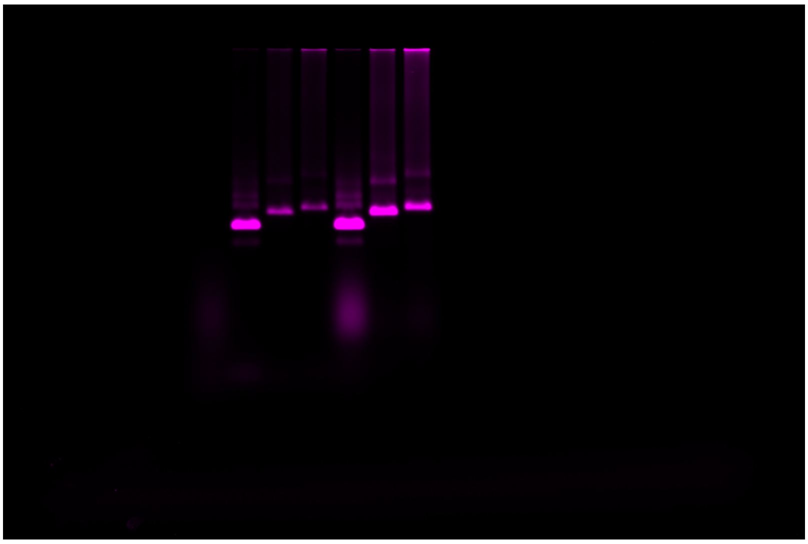

Source data for Suppl. Fig. 37

Uncropped gel after ethidium bromide staining

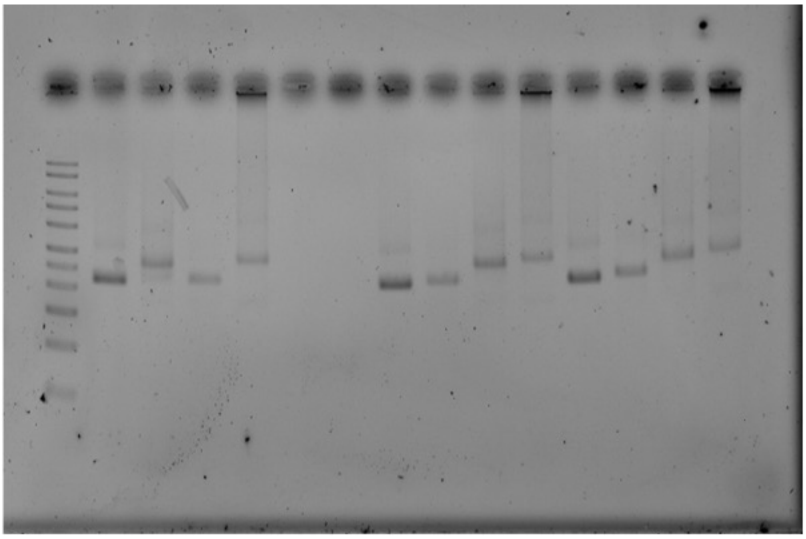

Same gel after Cy5 illumination

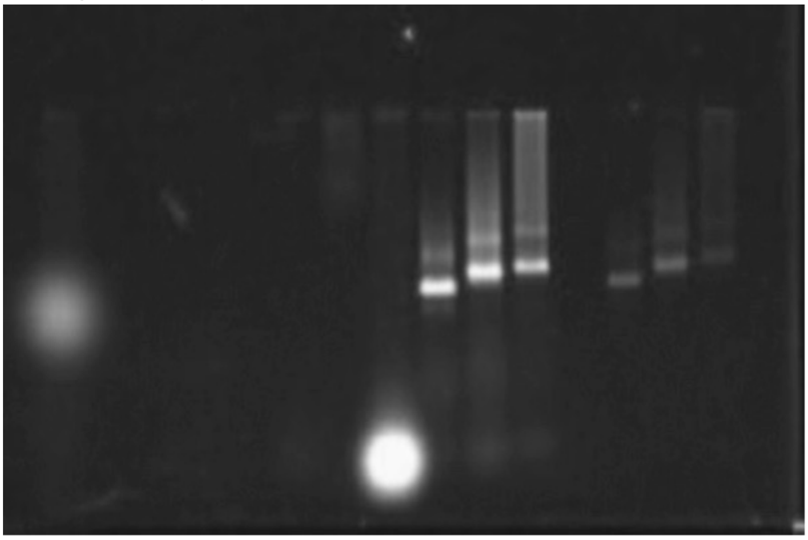

Source data for Suppl. Fig. 38

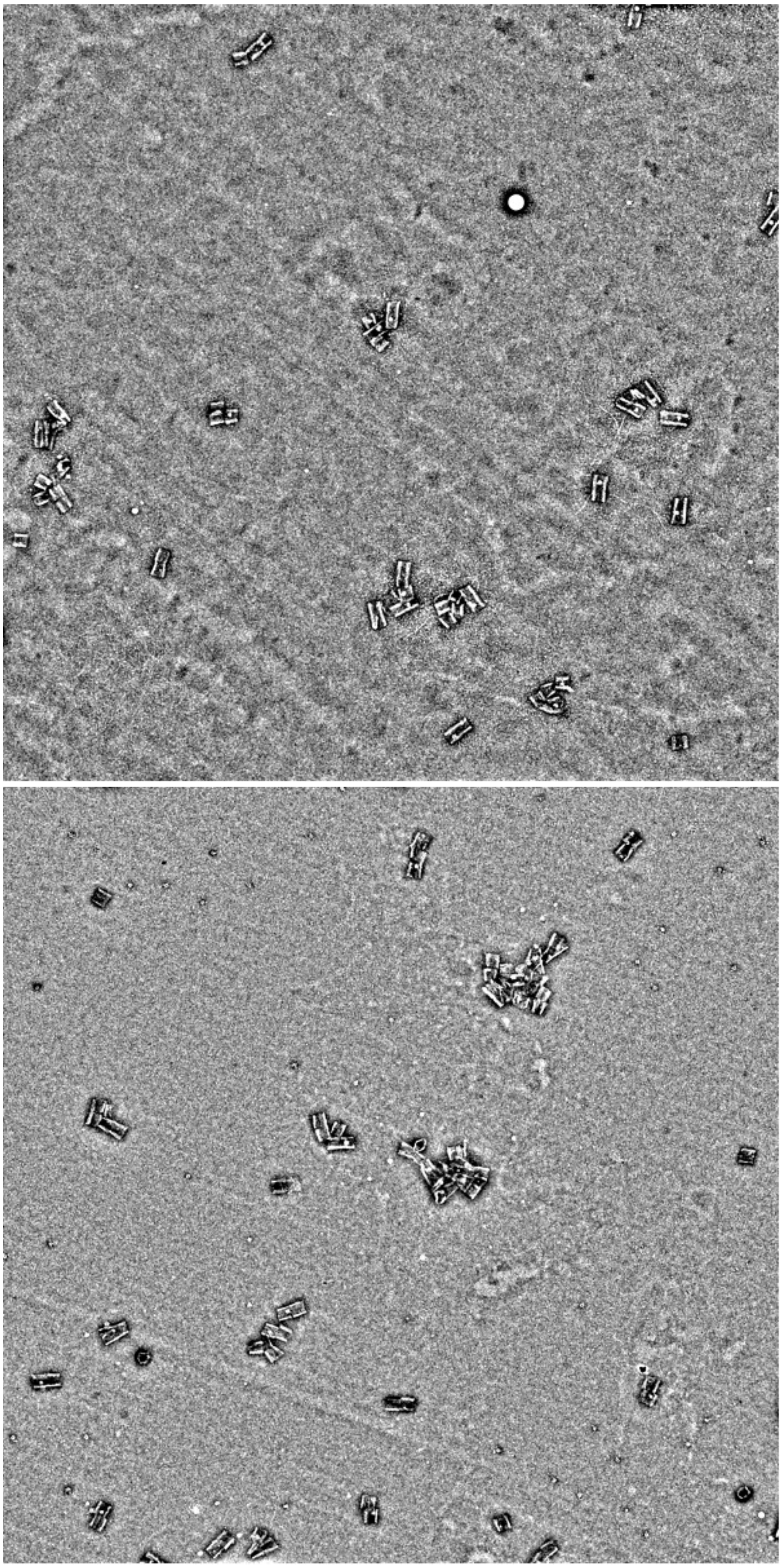

Supplement: Supplementary file 1 — Supplementary Figs. 1–41 and Tables 1–3. [file 41565_2024_1738_MOESM1_ESM.pdf]
