# Supplementary material for: A modular DNA origami nanocompartment for engineering a cell-free, protein unfolding and degradation pathway
Source: Nat Nanotechnol. 2024 Jul 29;19(10):1521–31. doi: 10.1038/s41565-024-01738-7 (PMC11486656; doi:10.1038/s41565-024-01738-7)
Supplement: Supplementary file 2 — Reporting Summary [file 41565_2024_1738_MOESM2_ESM.pdf]

nature portfolio

Corresponding author(s): Saccà Barbara

Last updated by author(s): 2024-05-14

Reporting Summary

Nature Portfolio wishes to improve the reproducibility of the work that we publish. This form provides structure for consistency and transparency in reporting. For further information on Nature Portfolio policies, see our Editorial Policies and the Editorial Policy Checklist.

Statistics

For all statistical analyses, confirm that the following items are present in the figure legend, table legend, main text, or Methods section.

n/a	Confirmed	
☐	☒	The exact sample size (n) for each experimental group/condition, given as a discrete number and unit of measurement
☐	☒	A statement on whether measurements were taken from distinct samples or whether the same sample was measured repeatedly
☒	☐	The statistical test(s) used AND whether they are one- or two-sided<br>*Only common tests should be described solely by name; describe more complex techniques in the Methods section.*
☒	☐	A description of all covariates tested
☒	☐	A description of any assumptions or corrections, such as tests of normality and adjustment for multiple comparisons
☐	☒	A full description of the statistical parameters including central tendency (e.g. means) or other basic estimates (e.g. regression coefficient) AND variation (e.g. standard deviation) or associated estimates of uncertainty (e.g. confidence intervals)
☒	☐	For null hypothesis testing, the test statistic (e.g. F, t, r) with confidence intervals, effect sizes, degrees of freedom and P value noted<br>*Give P values as exact values whenever suitable.*
☒	☐	For Bayesian analysis, information on the choice of priors and Markov chain Monte Carlo settings
☒	☐	For hierarchical and complex designs, identification of the appropriate level for tests and full reporting of outcomes
☒	☐	Estimates of effect sizes (e.g. Cohen's d, Pearson's r), indicating how they were calculated

Our web collection on statistics for biologists contains articles on many of the points above.

Software and code

Policy information about availability of computer code

Data collection	Thermocycler Mastercycler nexus gradient (Eppendorf); DS11 spectrophotometer (De Novix); autoFlex Speed (Bruker); proFIRE® (Dynamic Biosensors); Äkta pure (cytiva); Zetasizer (Malvern Analytics); Typhoon FLA9000 (GE healthcare Life Sciences); ChemoStar TS digital ECL imager (Intas); MultiModeTM (Bruker); JEOL JEM 1400Plus; Talos Arctica (Thermo Fisher); Spark 10 (Tecan) microplate reader; Vitrobot (FEI, Thermo Fisher); Evosep One UPLC; Orbitrap Elite mass spectrometer (Thermo Fisher Scientific).<br>Instrumentations used for sample manipulation and treatment are reported in the Methods section of the manuscript.
Data analysis	Excel 2022; OriginPro 2023; Fiji 2.3; ChemoStar software v.0.5.67 (Intas); NanoScope Analysis 1.5; Eman2; SerialEM; RELION 4.1; CTFFIND-4.1; ResolutionMap; ChimeraX; ColabFold; Proteome Discoverer 2.5/SequestHT search engine; Perseus 2.0.7; GPS software tool.

For manuscripts utilizing custom algorithms or software that are central to the research but not yet described in published literature, software must be made available to editors and reviewers. We strongly encourage code deposition in a community repository (e.g. GitHub). See the Nature Portfolio guidelines for submitting code & software for further information.

Data

Policy information about availability of data

All manuscripts must include a data availability statement. This statement should provide the following information, where applicable:
- Accession codes, unique identifiers, or web links for publicly available datasets
- A description of any restrictions on data availability
- For clinical datasets or third party data, please ensure that the statement adheres to our policy

All data generated and analysed during this study are included in this published article and its Supplementary Information files, Supplementary Figs. 1-41 and Supplementary Tables 1-3. Cryo-EM maps and atomic models reported in this study have been deposited in the Electron Microscopy Data Bank (EMDB) under accession codes EMD-18538. The mass spectrometry proteomics data have been deposited to the ProteomeXchange Consortium via the PRIDE partner repository (https://www.ebi.ac.uk/pride/archive/) with the dataset identifiers PXD045825 and PXD050816. All other data can be provided by the corresponding authors on request.

Research involving human participants, their data, or biological material

Policy information about studies with human participants or human data. See also policy information about sex, gender (identity/presentation), and sexual orientation and race, ethnicity and racism.

Reporting on sex and gender	n.a.
Reporting on race, ethnicity, or other socially relevant groupings	n.a.
Population characteristics	n.a.
Recruitment	n.a.
Ethics oversight	n.a.

Note that full information on the approval of the study protocol must also be provided in the manuscript.

Field-specific reporting

Please select the one below that is the best fit for your research. If you are not sure, read the appropriate sections before making your selection.

☒ Life sciences ☐ Behavioural & social sciences ☐ Ecological, evolutionary & environmental sciences

For a reference copy of the document with all sections, see nature.com/documents/nr-reporting-summary-flat.pdf

Life sciences study design

All studies must disclose on these points even when the disclosure is negative.

Sample size	No statistical method was used to predetermine sample-size. For unfolding assays, sample size was at least n > 6 (the exact number of technical and/or biological replicates is reported in the legends to the Figures). For TEM imaging, sample sizes were at least n > 350 (exact sizes are reported in Suppl. Table 1).
Data exclusions	No data were excluded from the analyses.
Replication	We verified the reproducibility of the experimental findings by repeating the experiments at least twice using n > 3 technical replicates each time. Electrophoretic gel analysis of the same samples was executed several times in various settings and (in some cases) by distinct experimenters. Reproducibility was observed in all cases.
Randomization	Randomization was not relevant to our study. Therefore no randomization measures were applied.
Blinding	Blinding was not applicable nor relevant to our study. Investigators were not blinded to group allocation during data collection and/or analysis.

Reporting for specific materials, systems and methods

We require information from authors about some types of materials, experimental systems and methods used in many studies. Here, indicate whether each material, system or method listed is relevant to your study. If you are not sure if a list item applies to your research, read the appropriate section before selecting a response.

Materials & experimental systems

n/a	Involved in the study	
☐	☒	Antibodies
☒	☐	Eukaryotic cell lines
☒	☐	Palaeontology and archaeology
☒	☐	Animals and other organisms
☒	☐	Clinical data
☒	☐	Dual use research of concern
☒	☐	Plants

Methods

n/a	Involved in the study	
☒	☐	ChIP-seq
☒	☐	Flow cytometry
☒	☐	MRI-based neuroimaging

Antibodies

Antibodies used	SDS22 antibody B-6 (Santa Cruz Biotechnologies)<br>UBXN-2 antibody (PMID 23649807)<br>PP1γ antibody (E-9; Santa Cruz Biotechnology)<br>Anti-I3 (PMID 25298395)<br>Alexa Fluor 647-modified phosphotyrosine antibody (P-Tyr-01; ThermoFisher)
Validation	SDS22 antibody B-6 (Santa Cruz Biotechnologies); https://datasheets.scbt.com/sc-398864.pdf<br>UBXN-2 antibody (PMID 23649807); https://pubmed.ncbi.nlm.nih.gov/23649807/<br>PP1γ antibody (E-9; Santa Cruz Biotechnology); https://datasheets.scbt.com/sc-7482.pdf<br>Anti-I3 (PMID 25298395); https://pubmed.ncbi.nlm.nih.gov/25298395/<br>Alexa Fluor 647-modified phosphotyrosine antibody (P-Tyr-01; ThermoFisher); https://www.thermofisher.com/order/genome-database/dataSheetPdf?producttype=antibody&productsubtype=antibody_primary&productId=MA5-18172&version=Local

Plants

Seed stocks	n.a.
Novel plant genotypes	n.a.
Authentication	n.a.
