# Supplementary material for: A modular DNA origami nanocompartment for engineering a cell-free, protein unfolding and degradation pathway
Source: Nat Nanotechnol. 2024 Jul 29;19(10):1521–31. doi: 10.1038/s41565-024-01738-7 (PMC11486656; doi:10.1038/s41565-024-01738-7)

Source Data Images for Figures and Extended Data Figures

Source Data Fig. 1b: Wide-field TEM images of p97

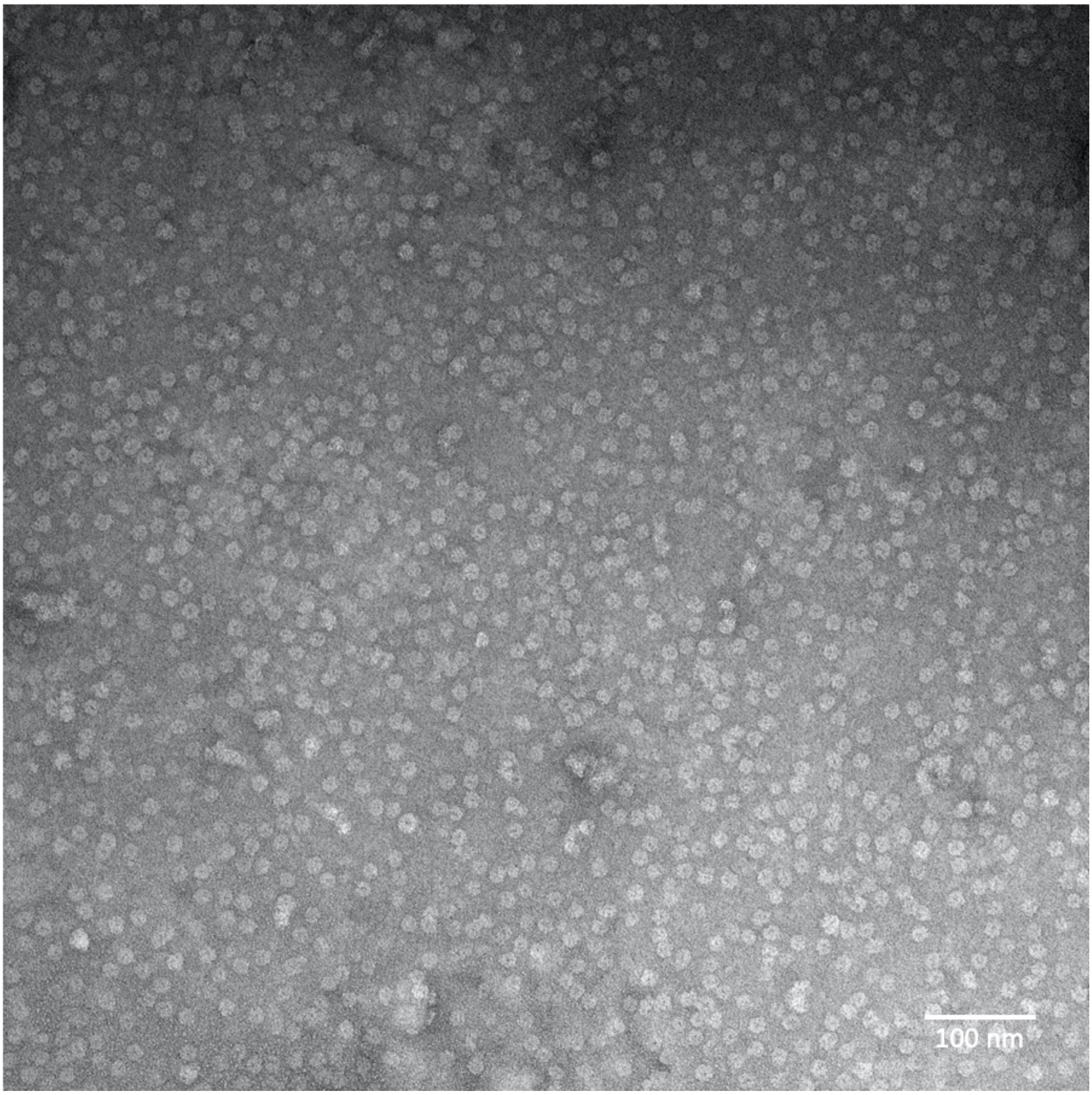

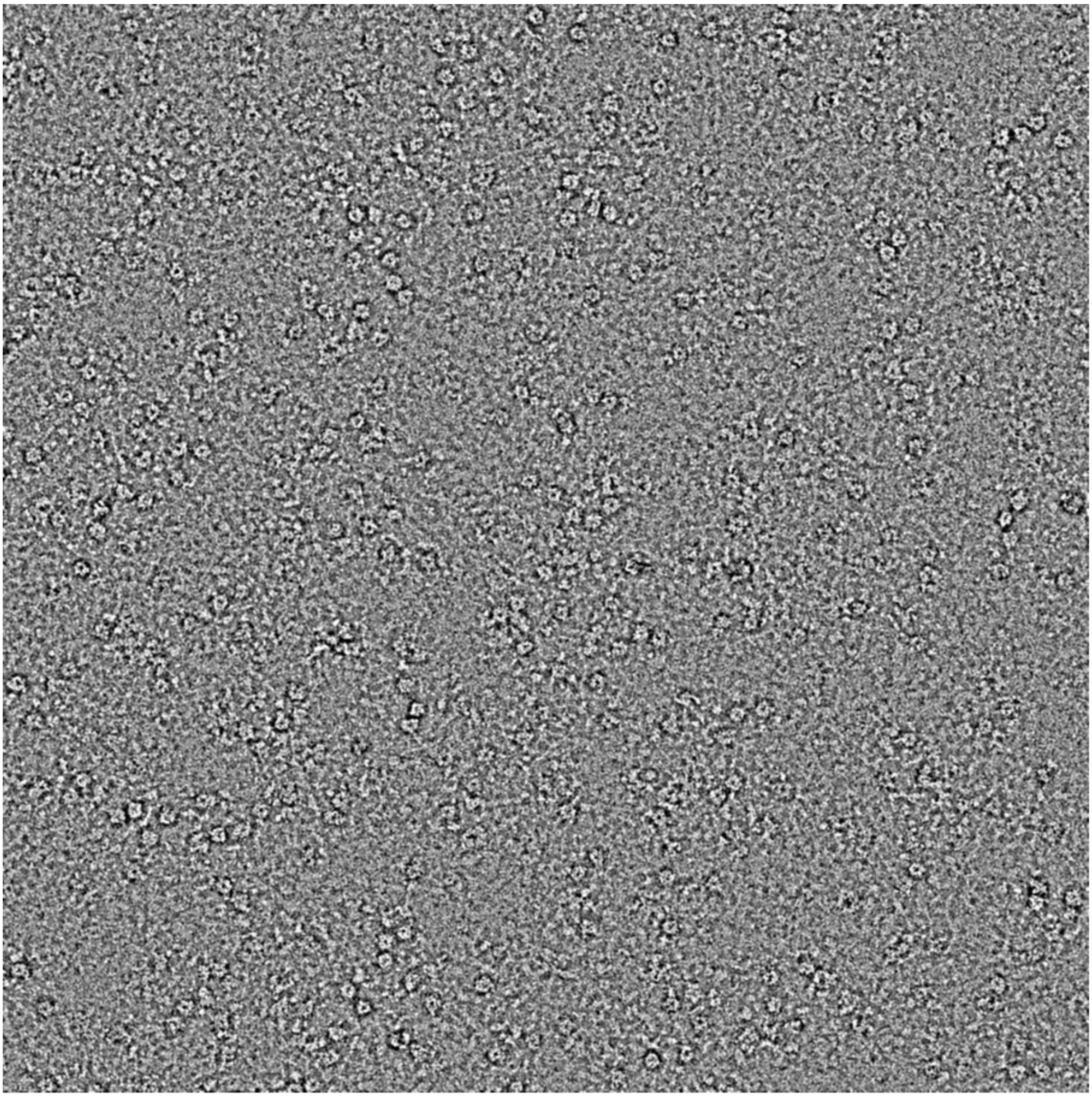

Source Data Fig. 2a: TEM averaged classes of N and E

Class averages of N

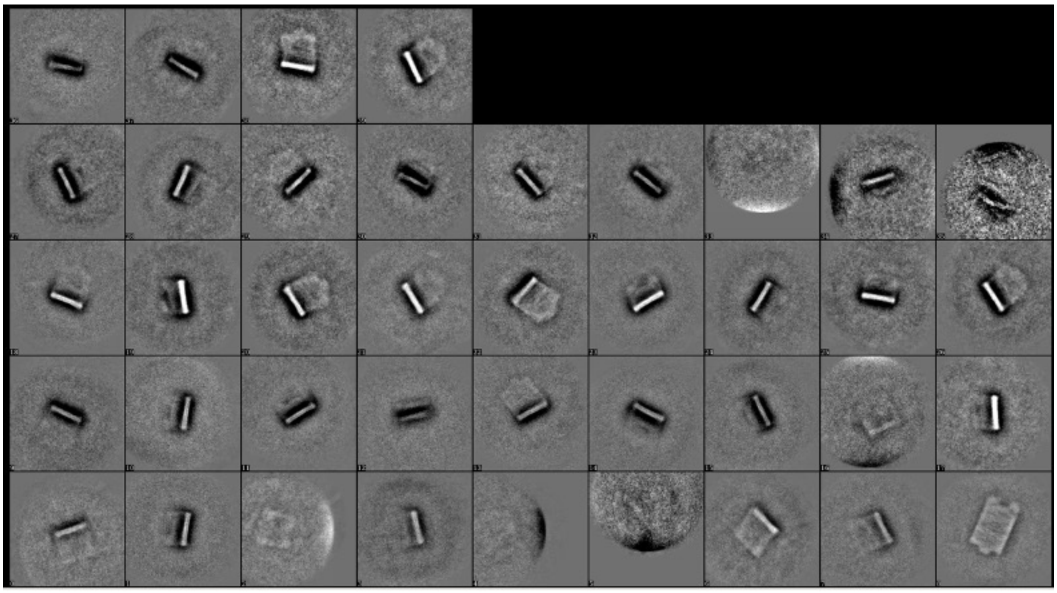

Class averages of E

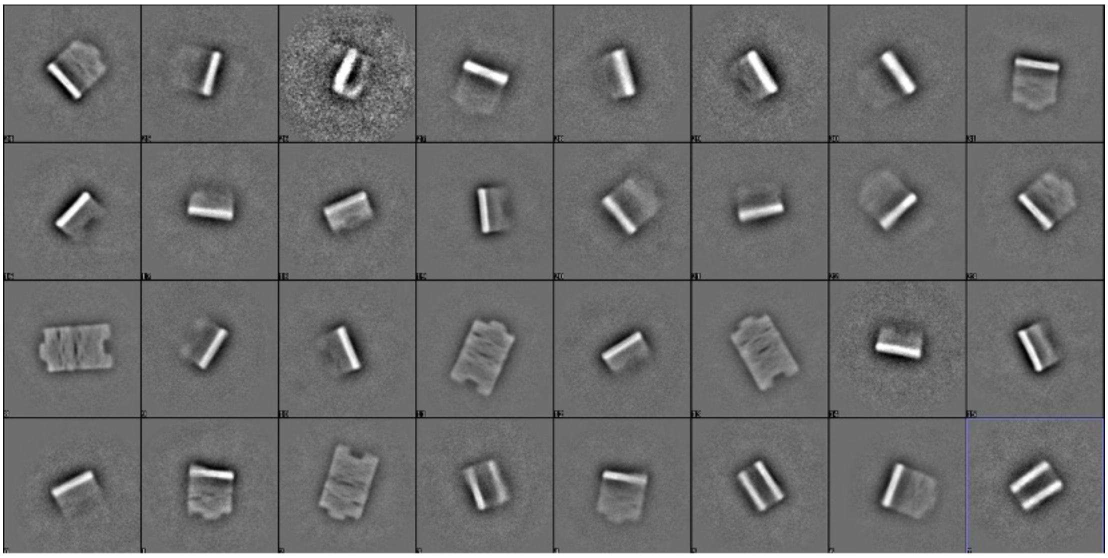

Source Data Fig. 2b: Wide-field TEM images of NE

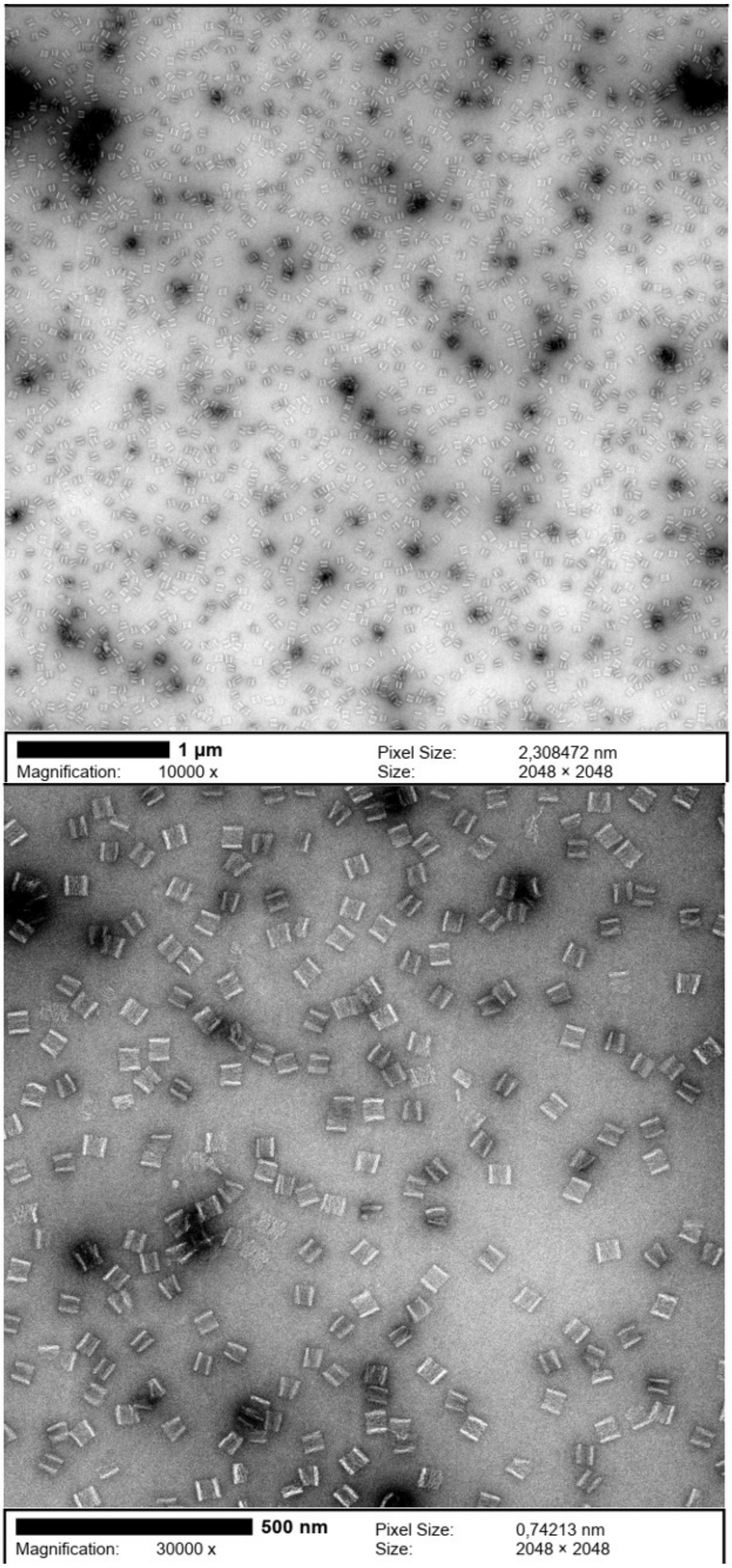

Source Data Fig. 2c: Class averages of NE

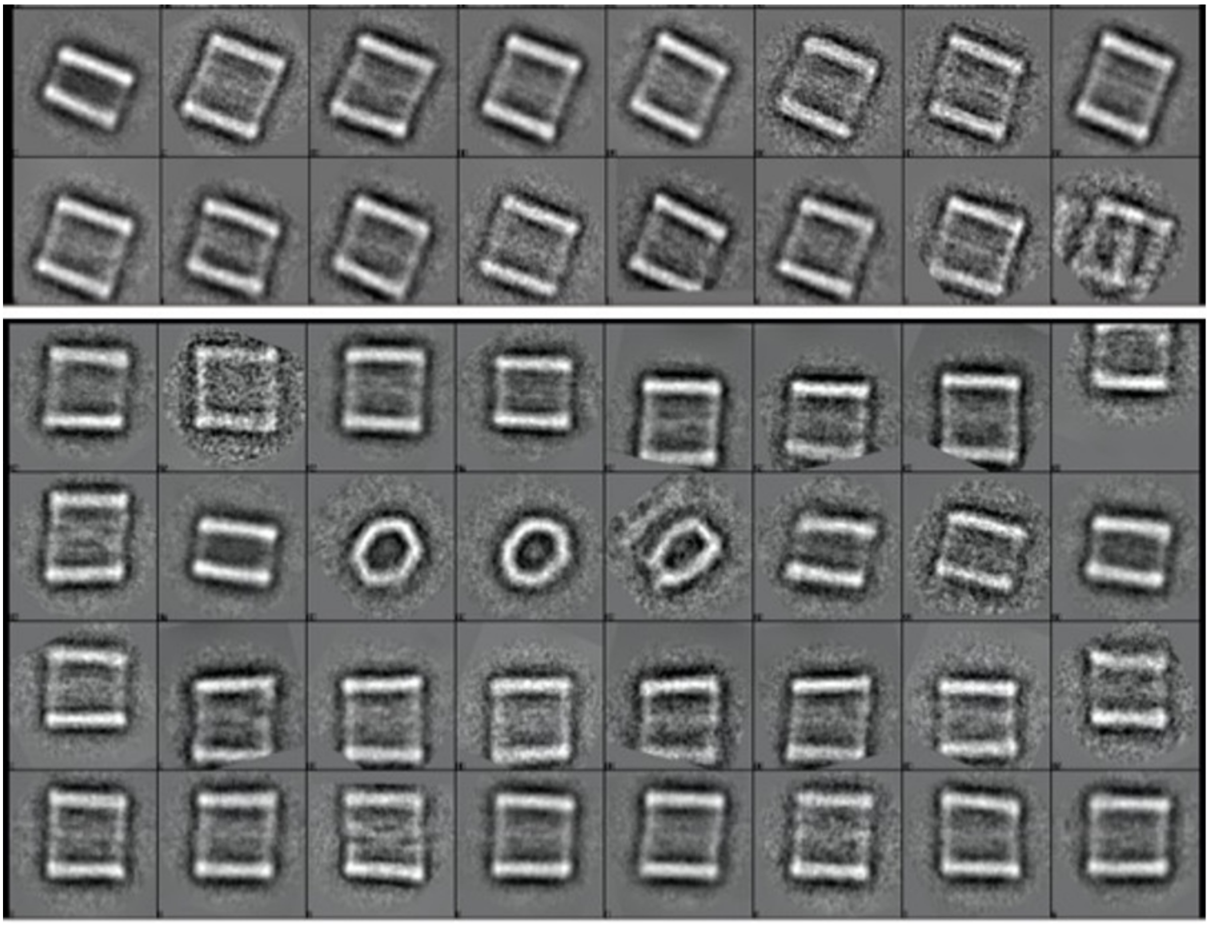

Source Data Fig. 2e: TEM images of L

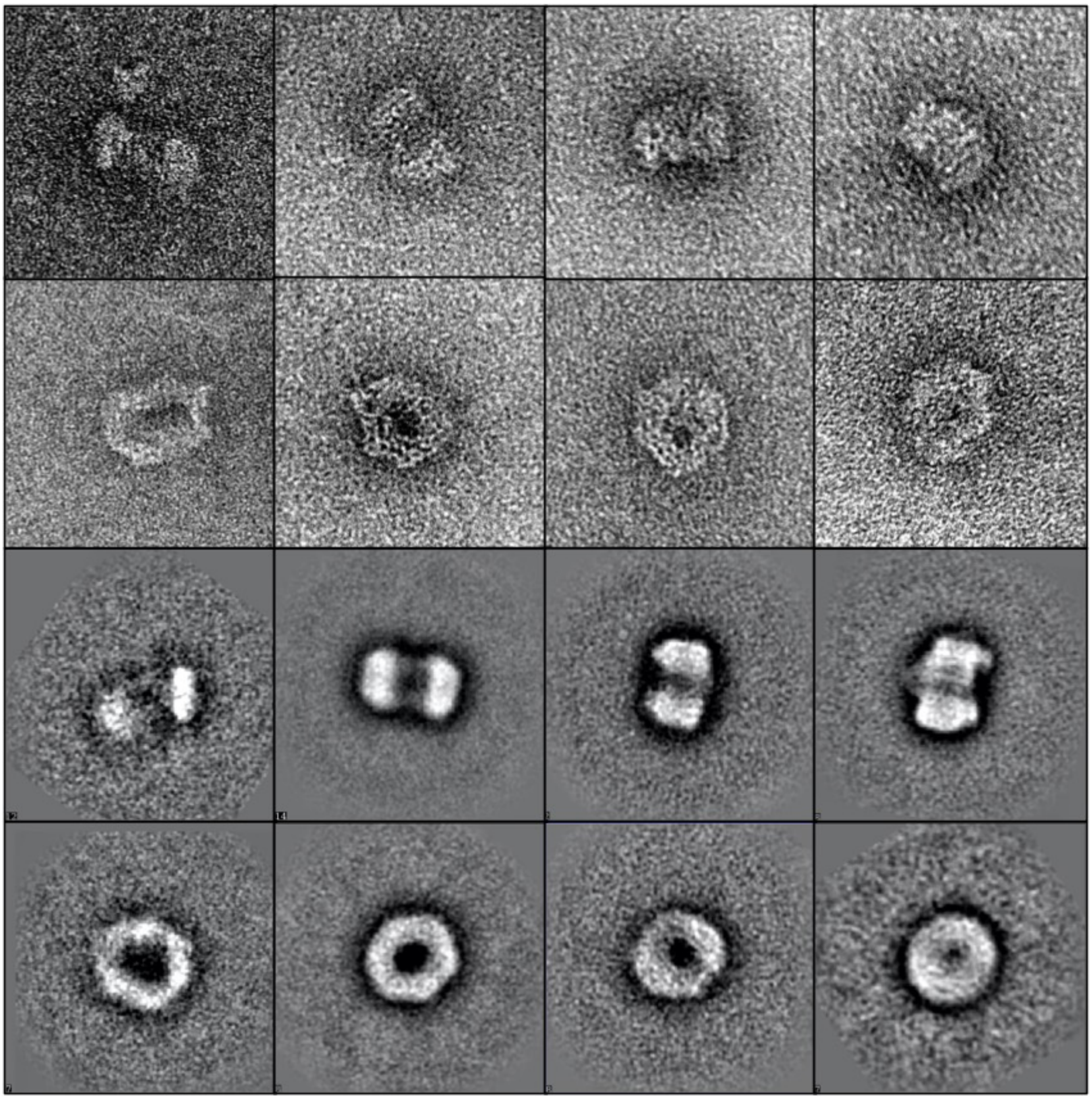

Source Data Fig. 2g: Wide-field TEM images of A^L

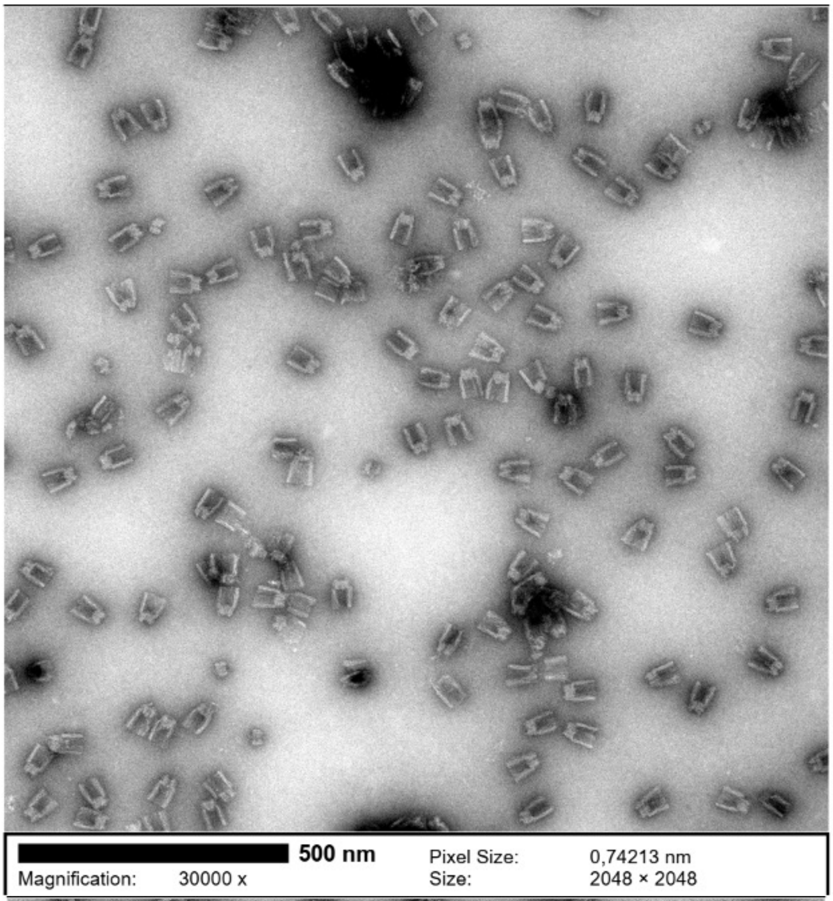

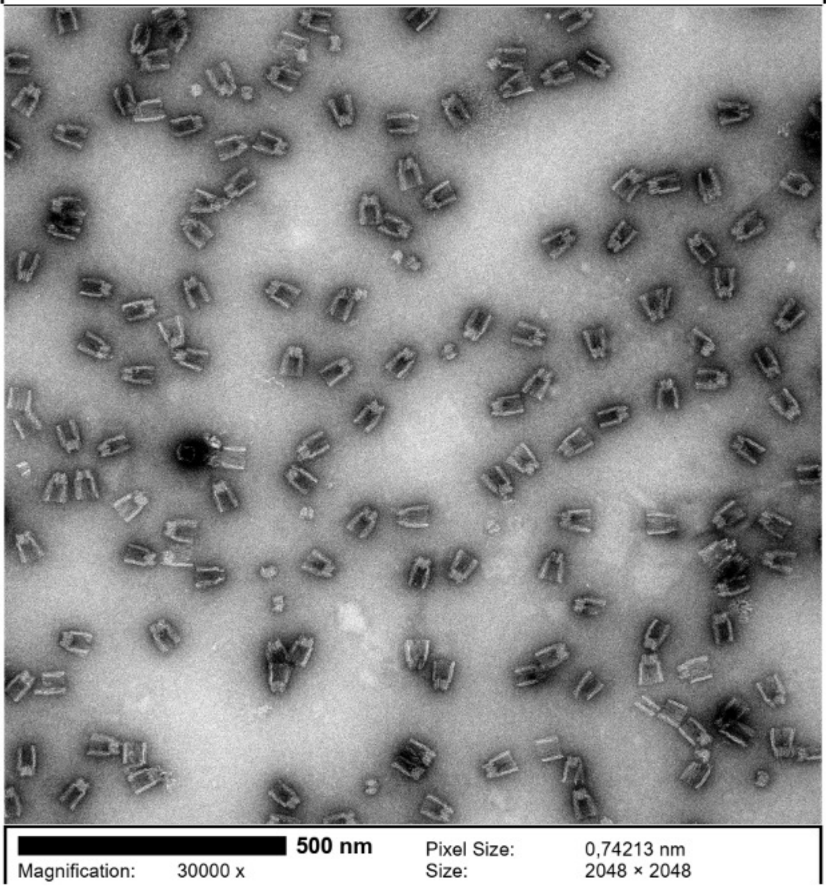

Source Data Fig. 2h: Wide-field TEM images and class averages of A^{2L}

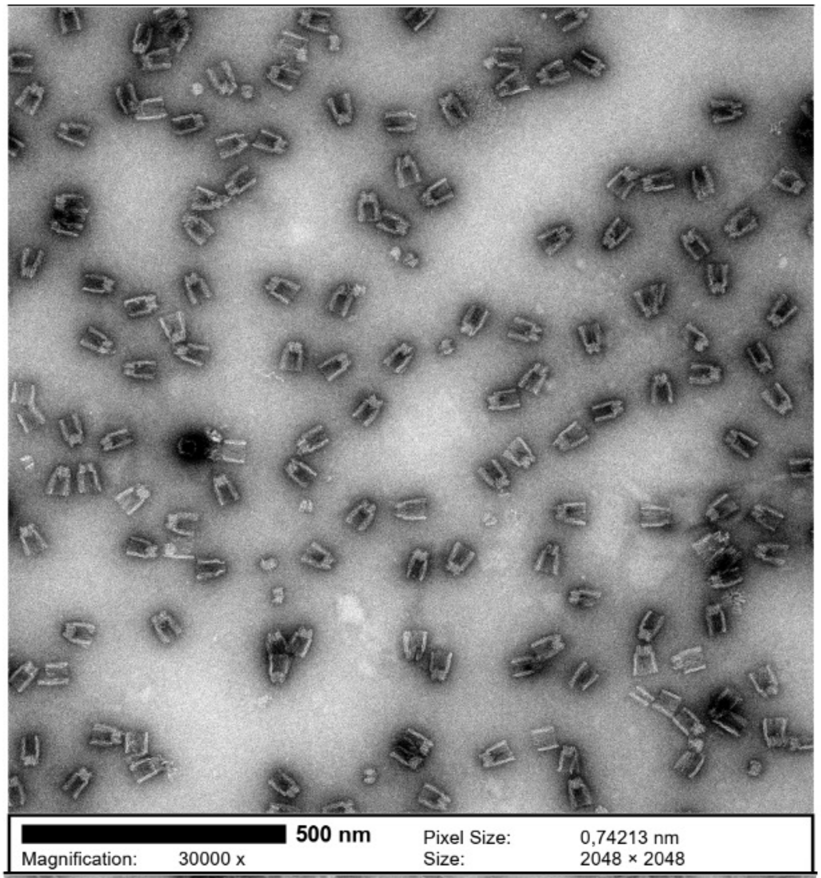

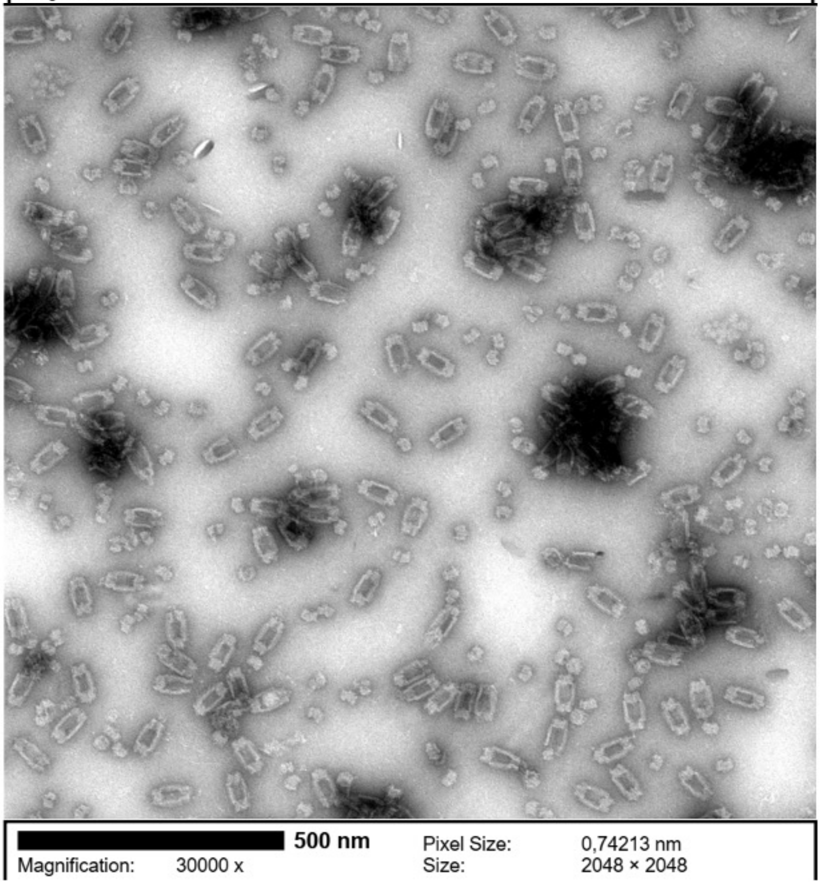

Class averages of A^{2L}

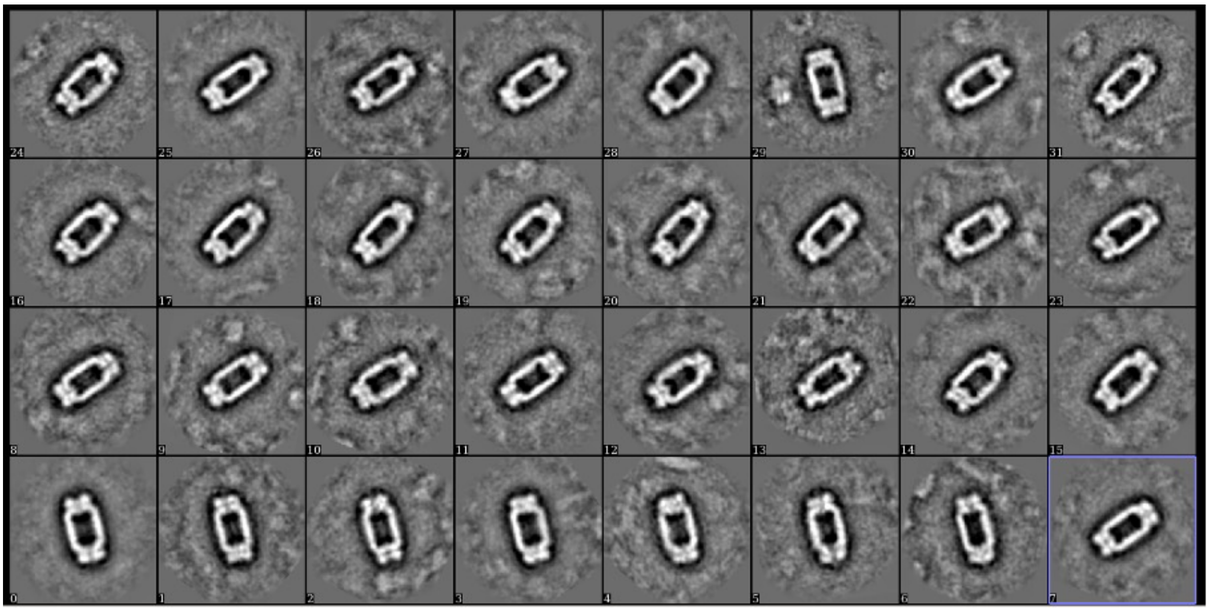

Source Data Fig. 2j: Wide-field TEM images and class averages of AB

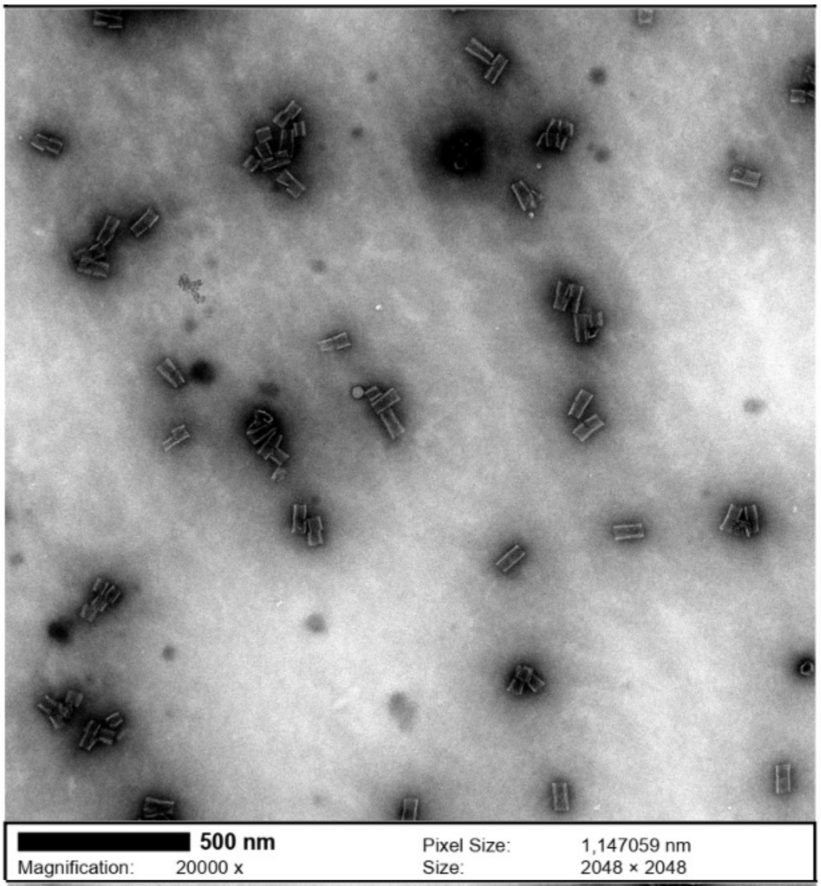

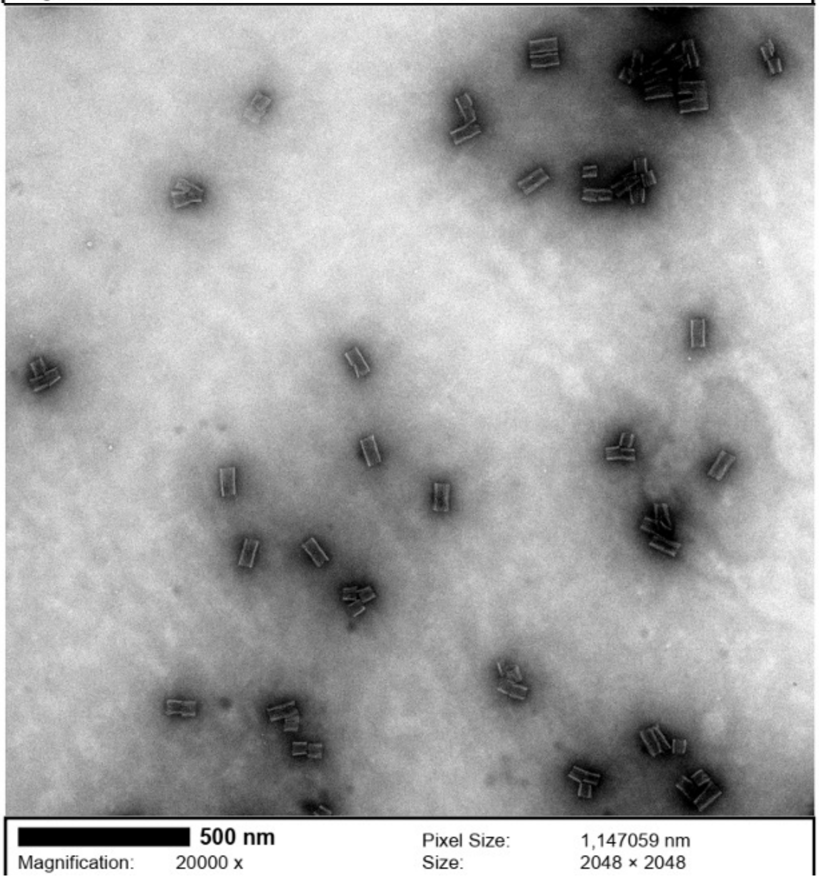

Class averages of AB

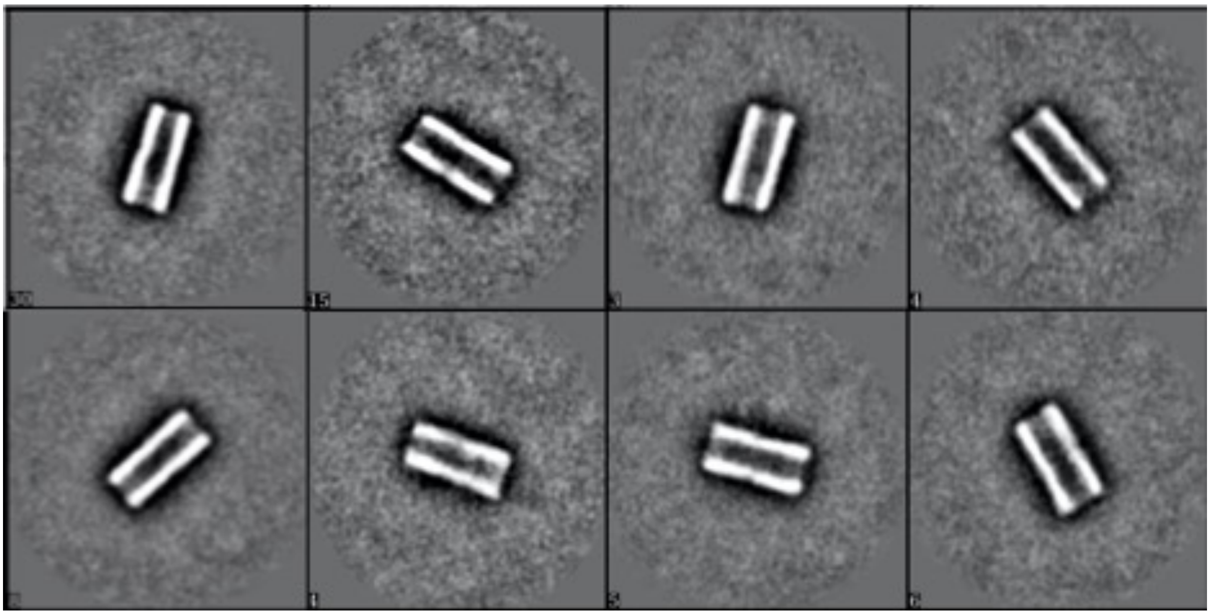

Source Data Fig. 2k: TEM images of ABC

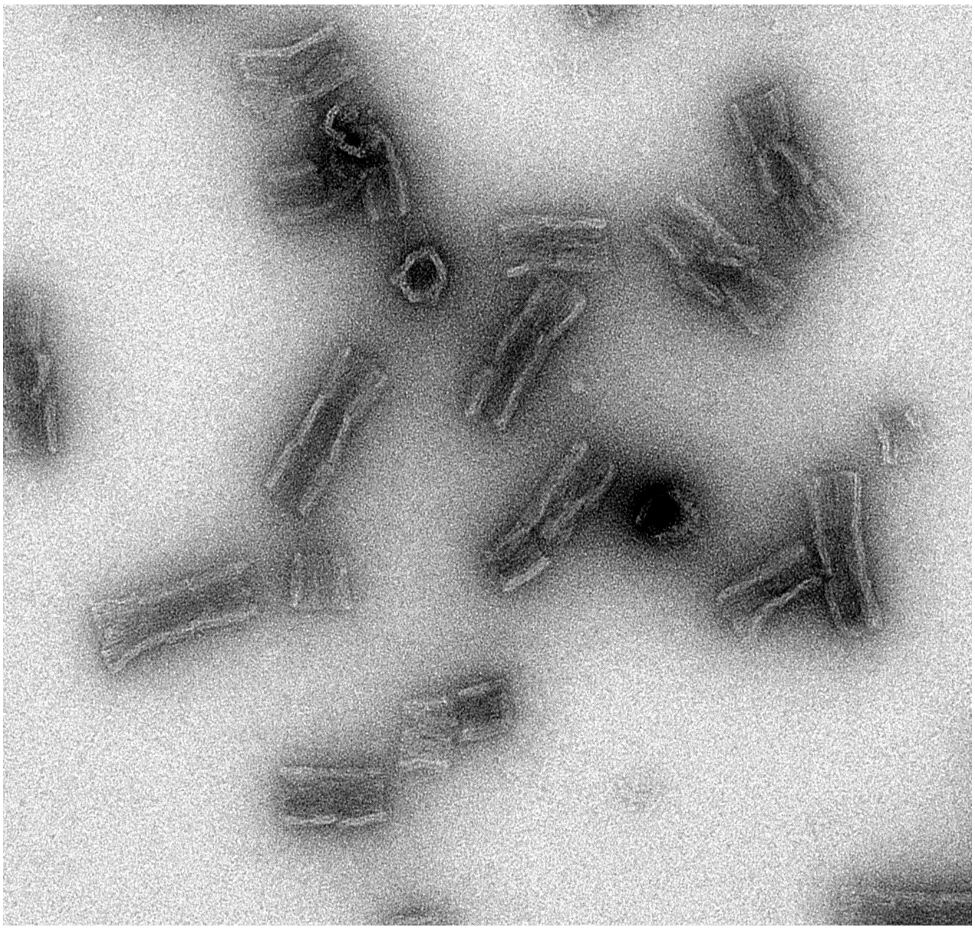

Source Data Fig. 3b: Unprocessed AGE of A(p97)

Ethidium bromide staining

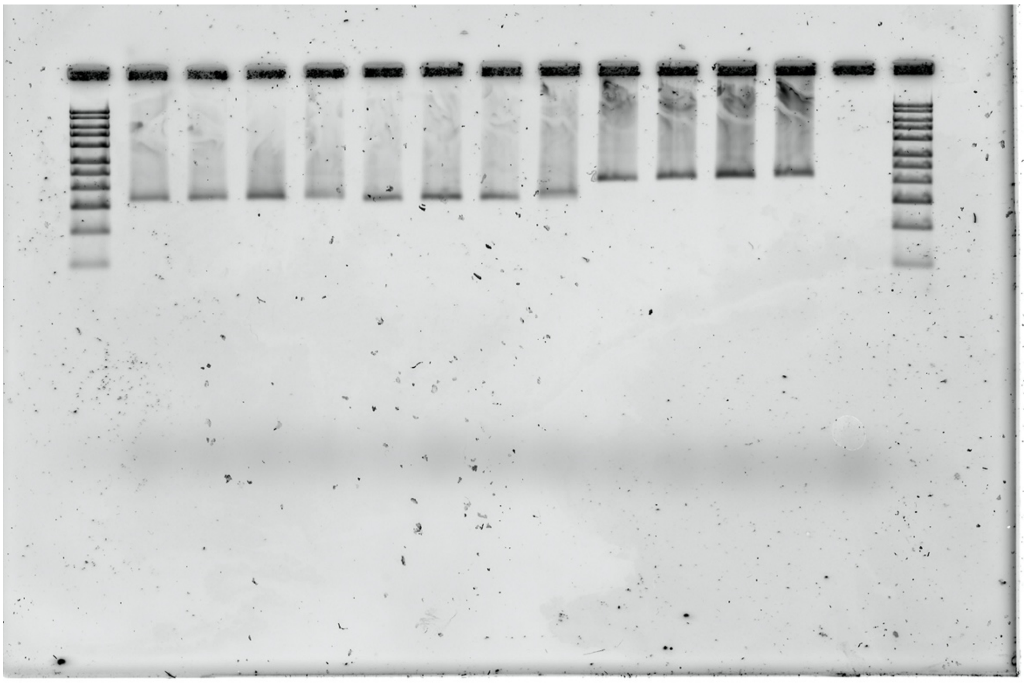

FAM illumination

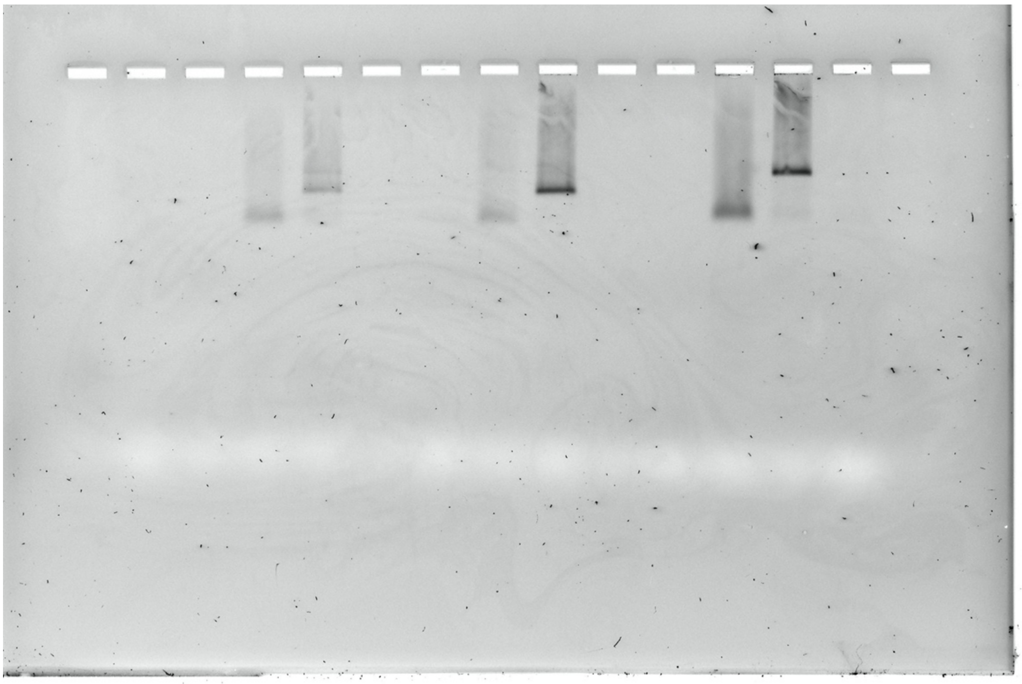

Source Data Fig. 3c: Wide-field TEM images of A(p97)

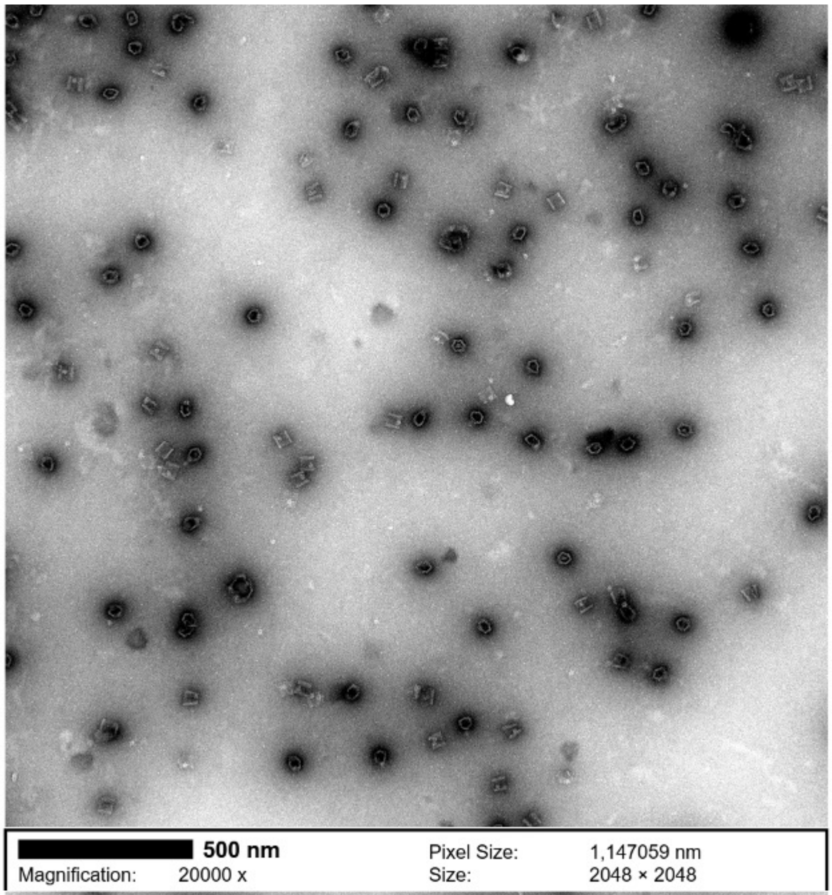

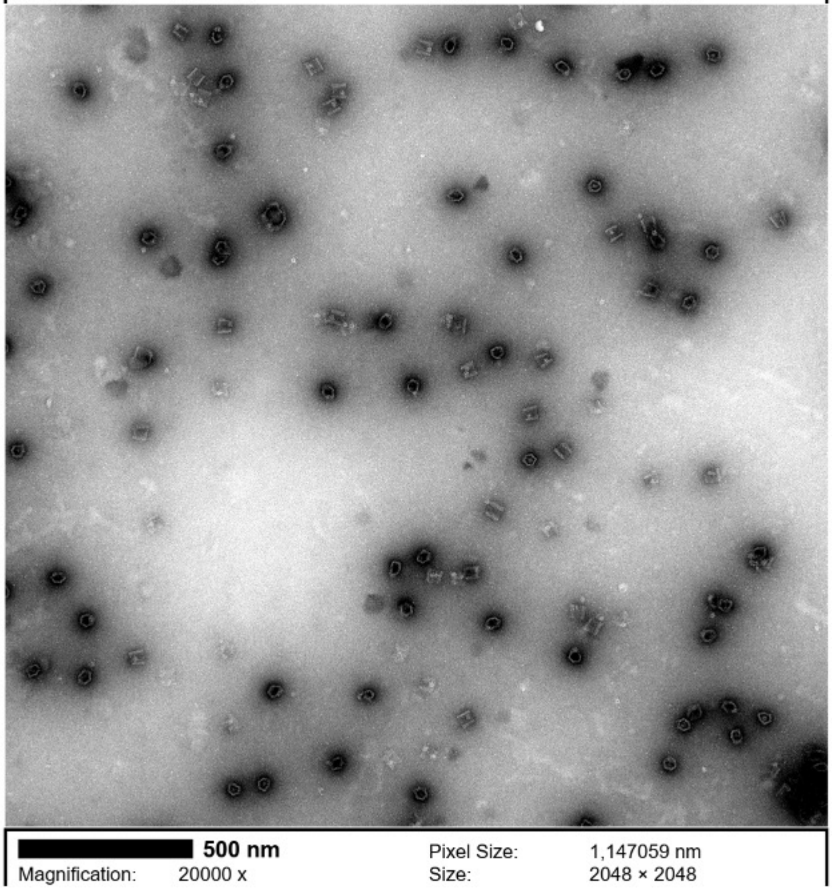

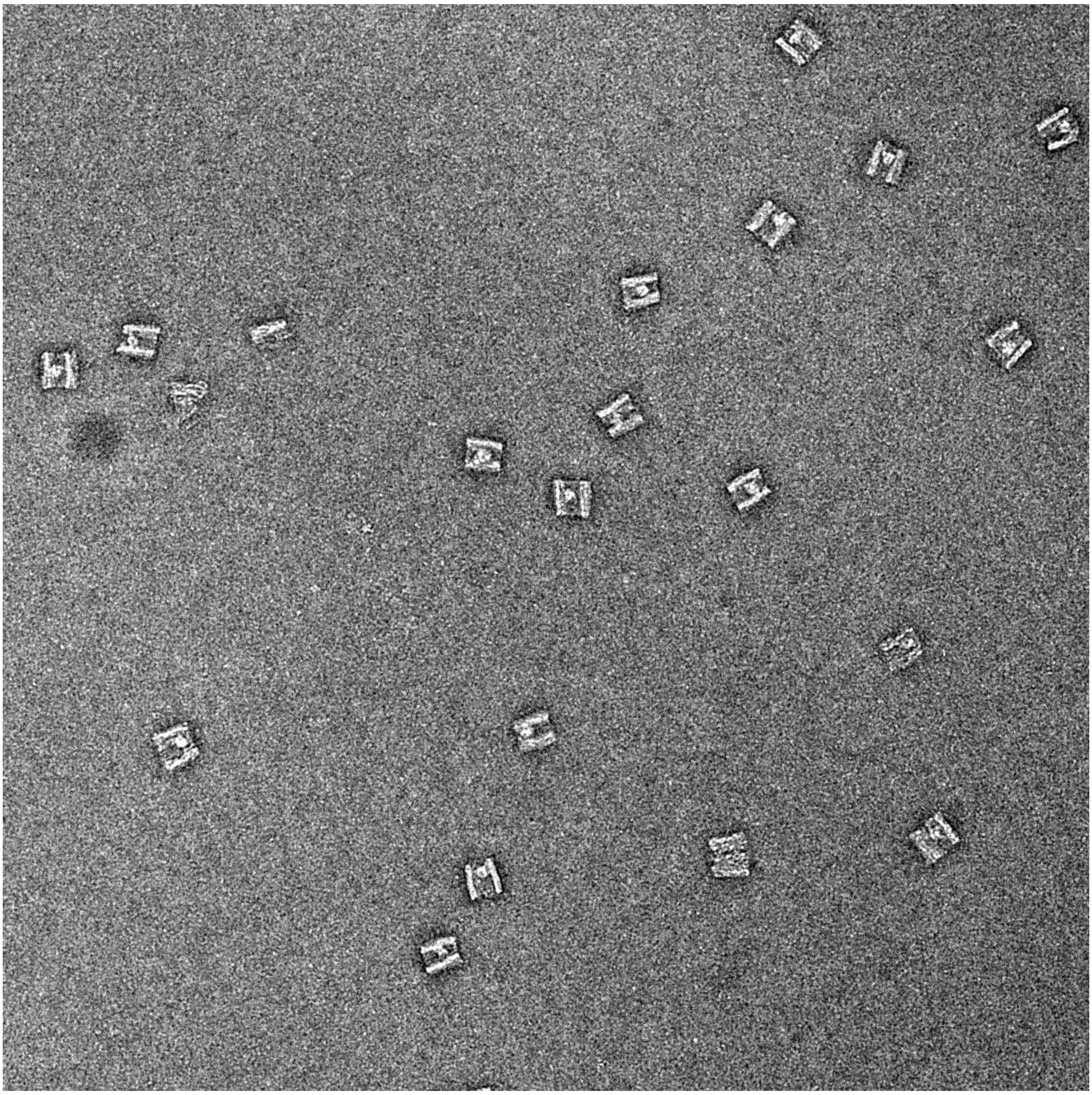

Source Data Fig. 3e: Slices of the 3D cryoEM density map

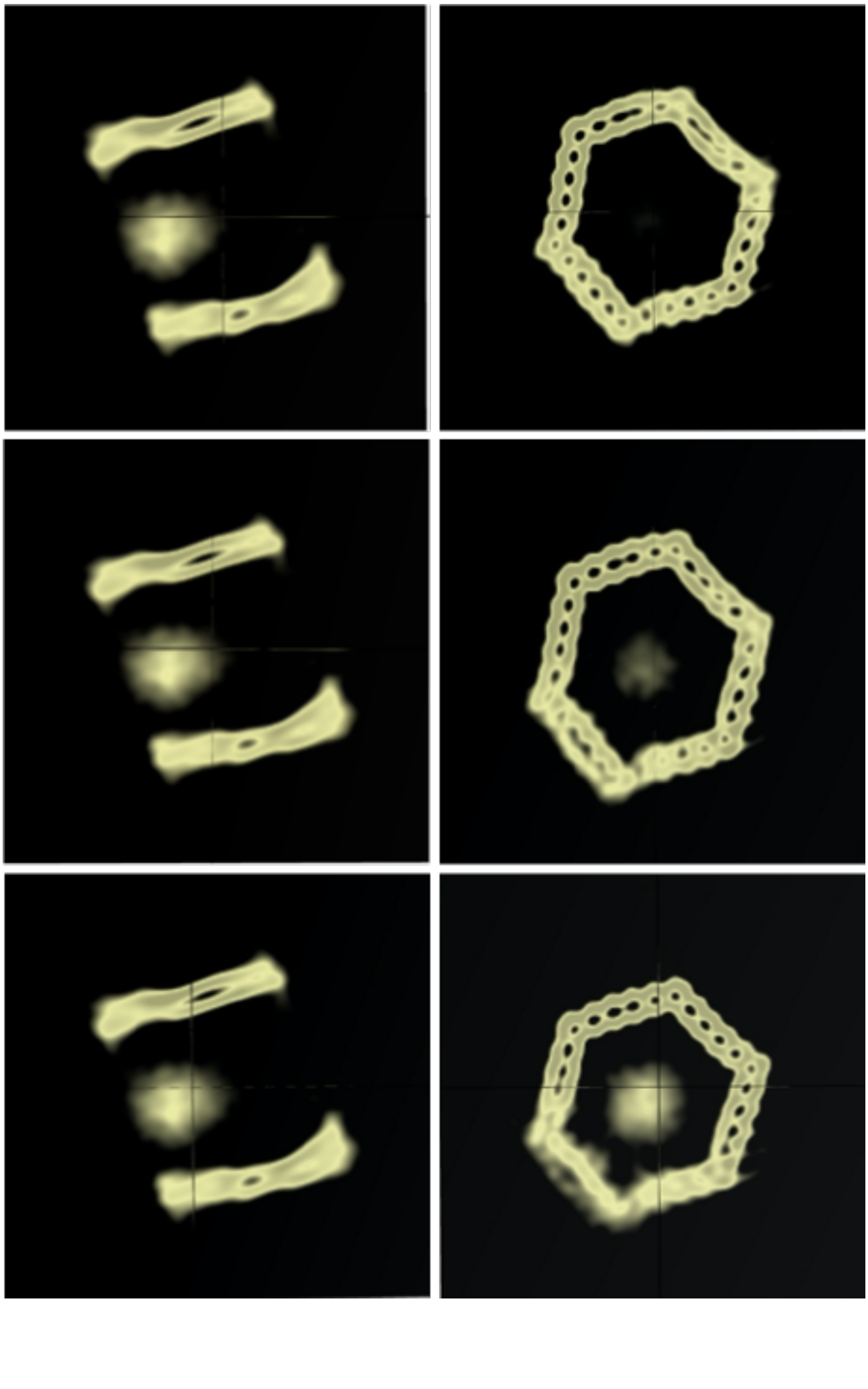

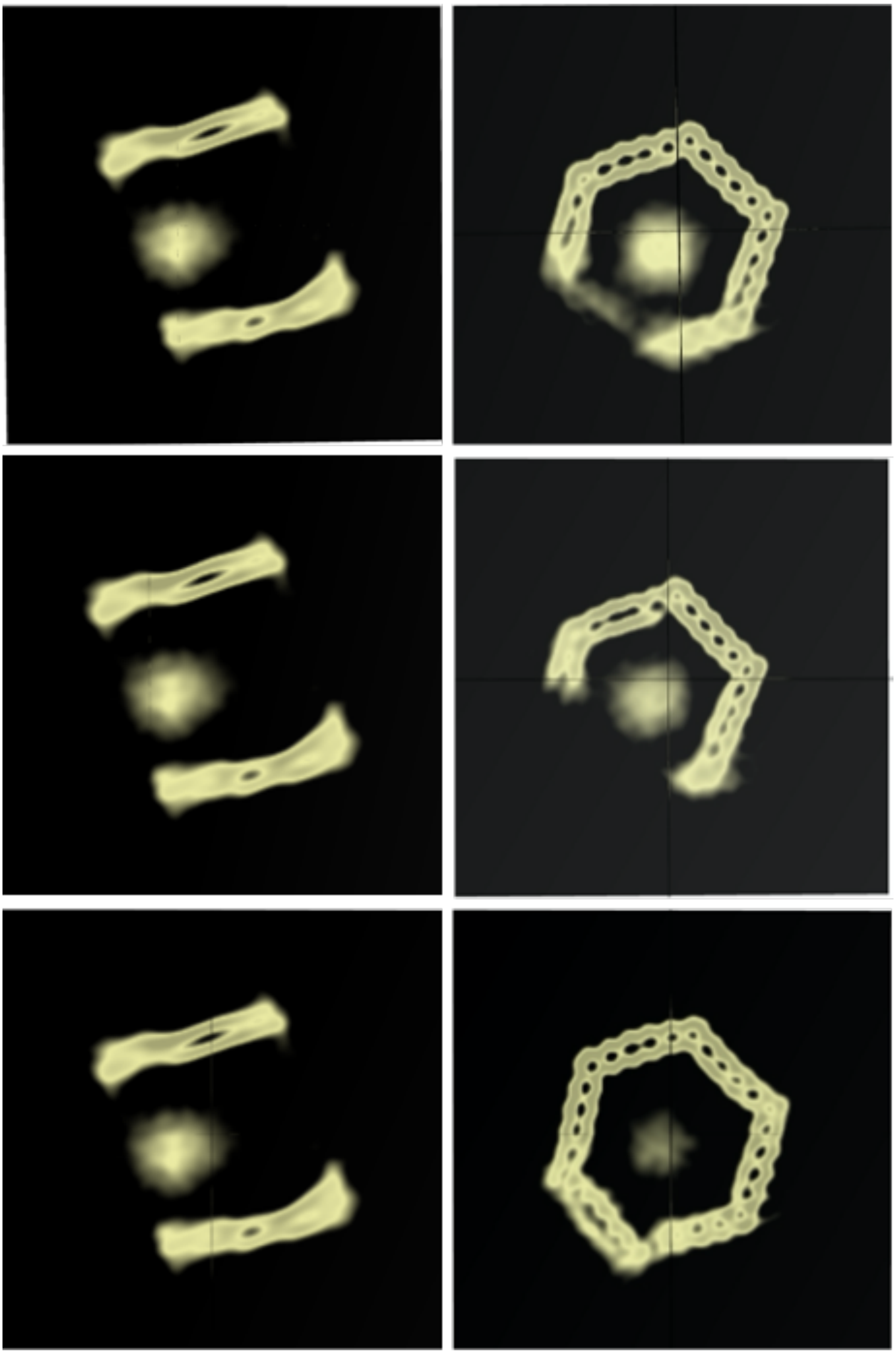

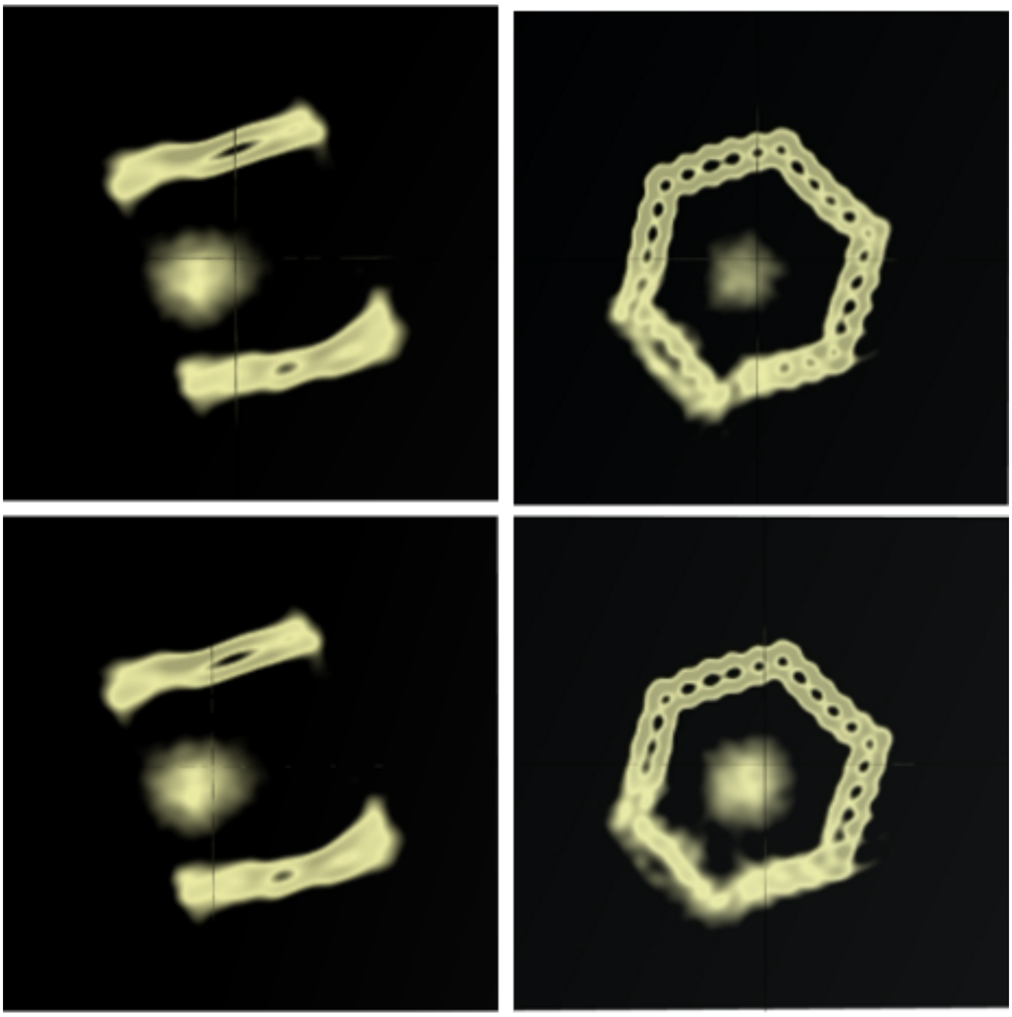

Source Data Fig. 4a: Wide-field TEM images of A

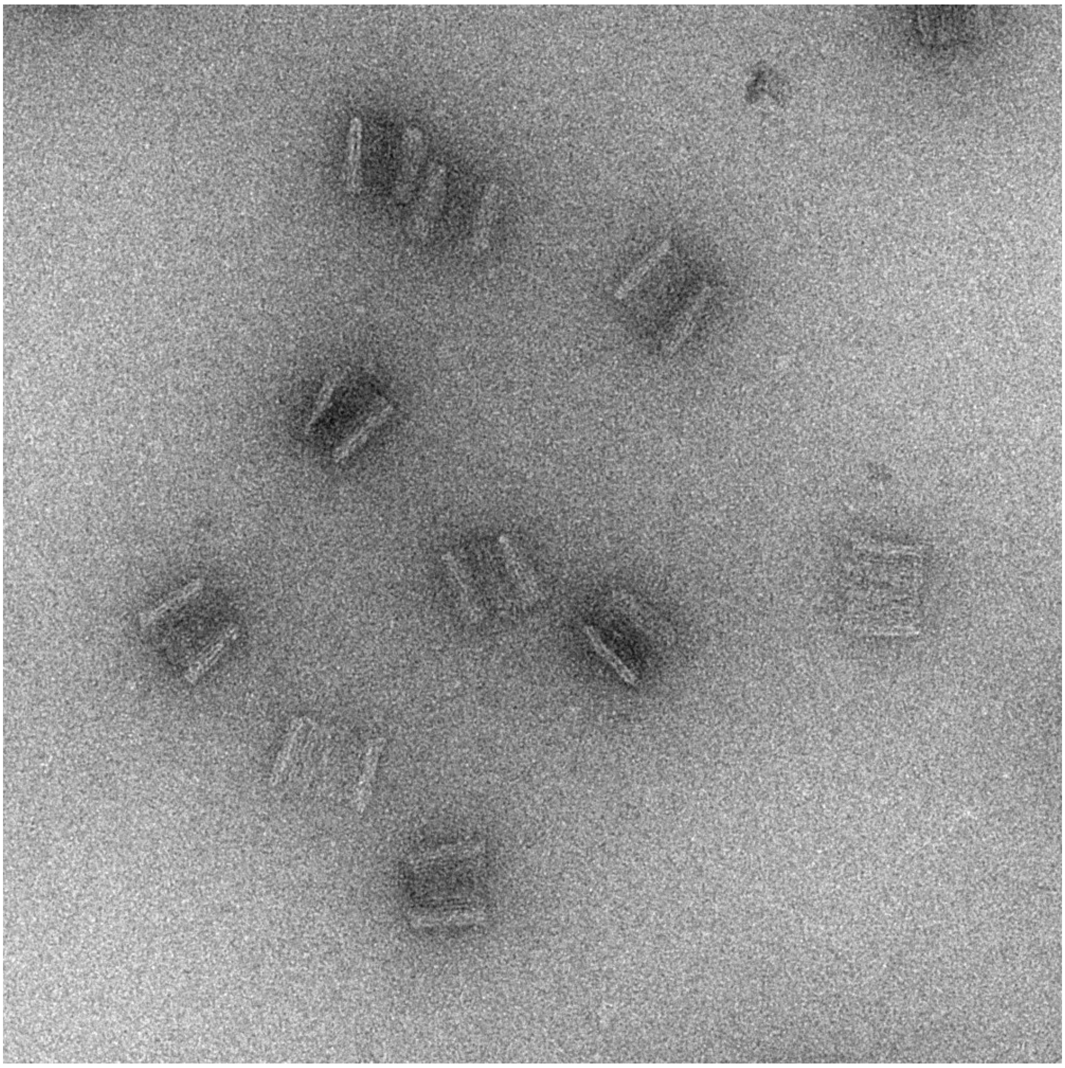

Source Data Fig. 4b: Wide-field TEM images of A(p97)

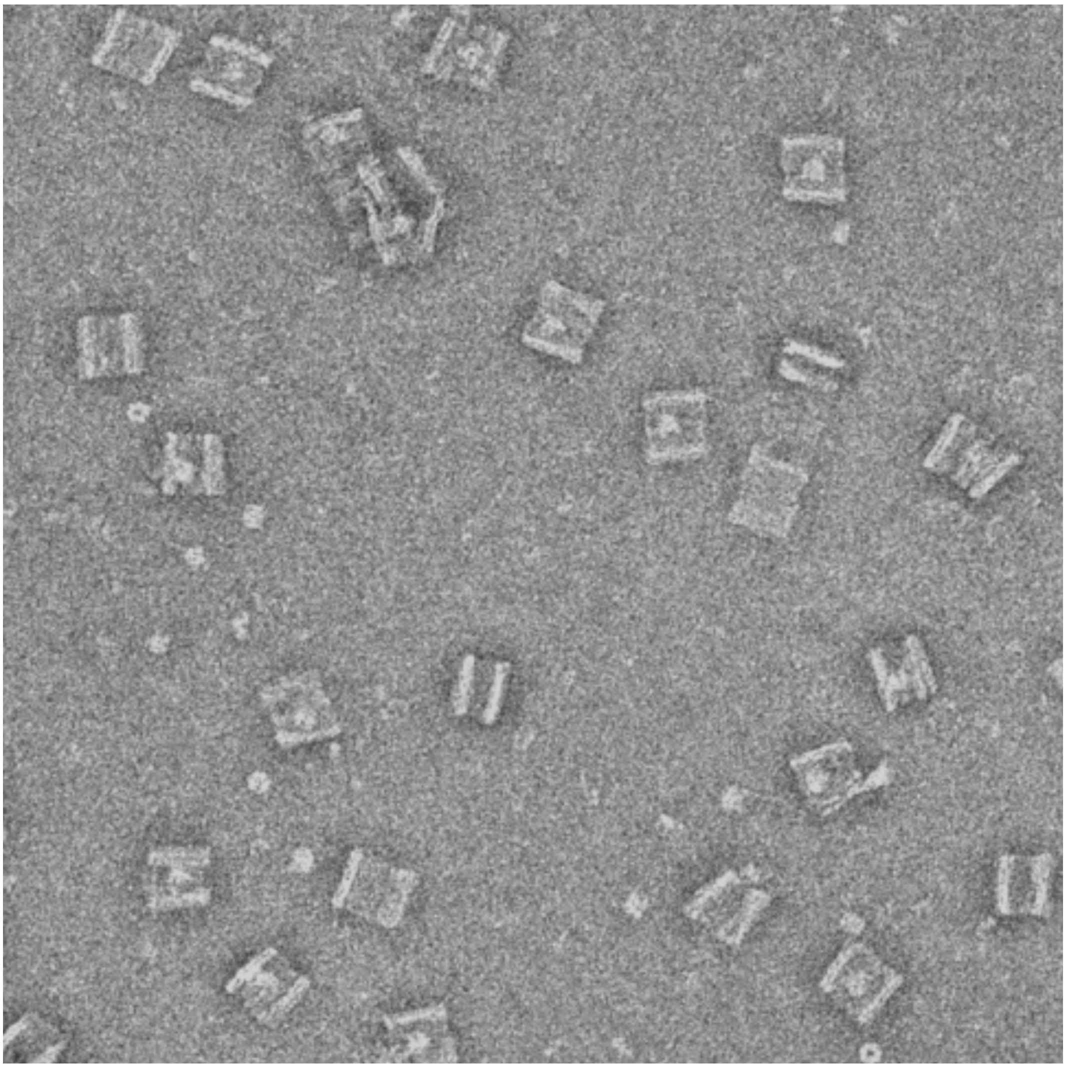

Source Data Fig. 4c: Wide-field TEM images of A_{rel}

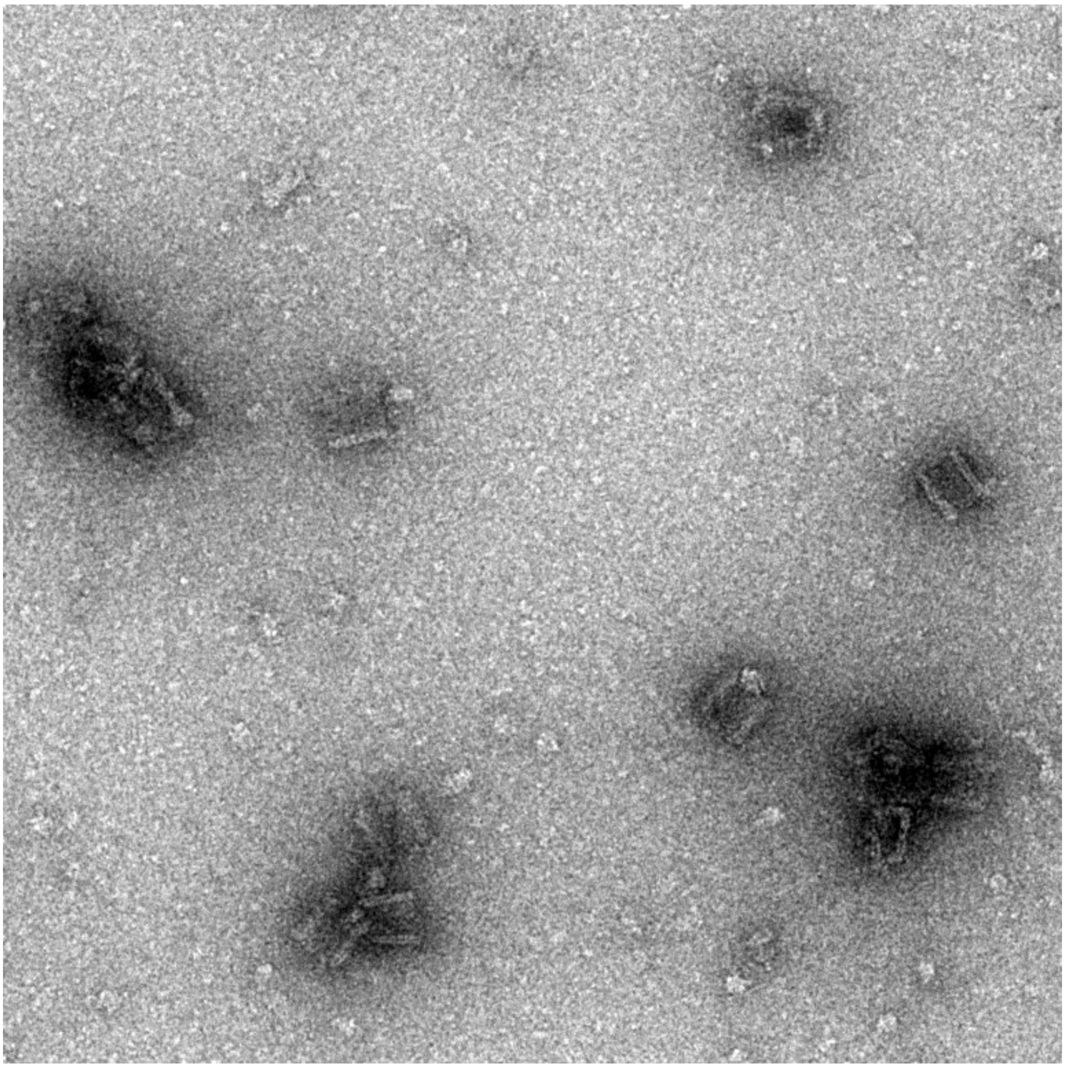

Source Data Fig. 4d: Wide-field TEM images of A^{L4}(p97)

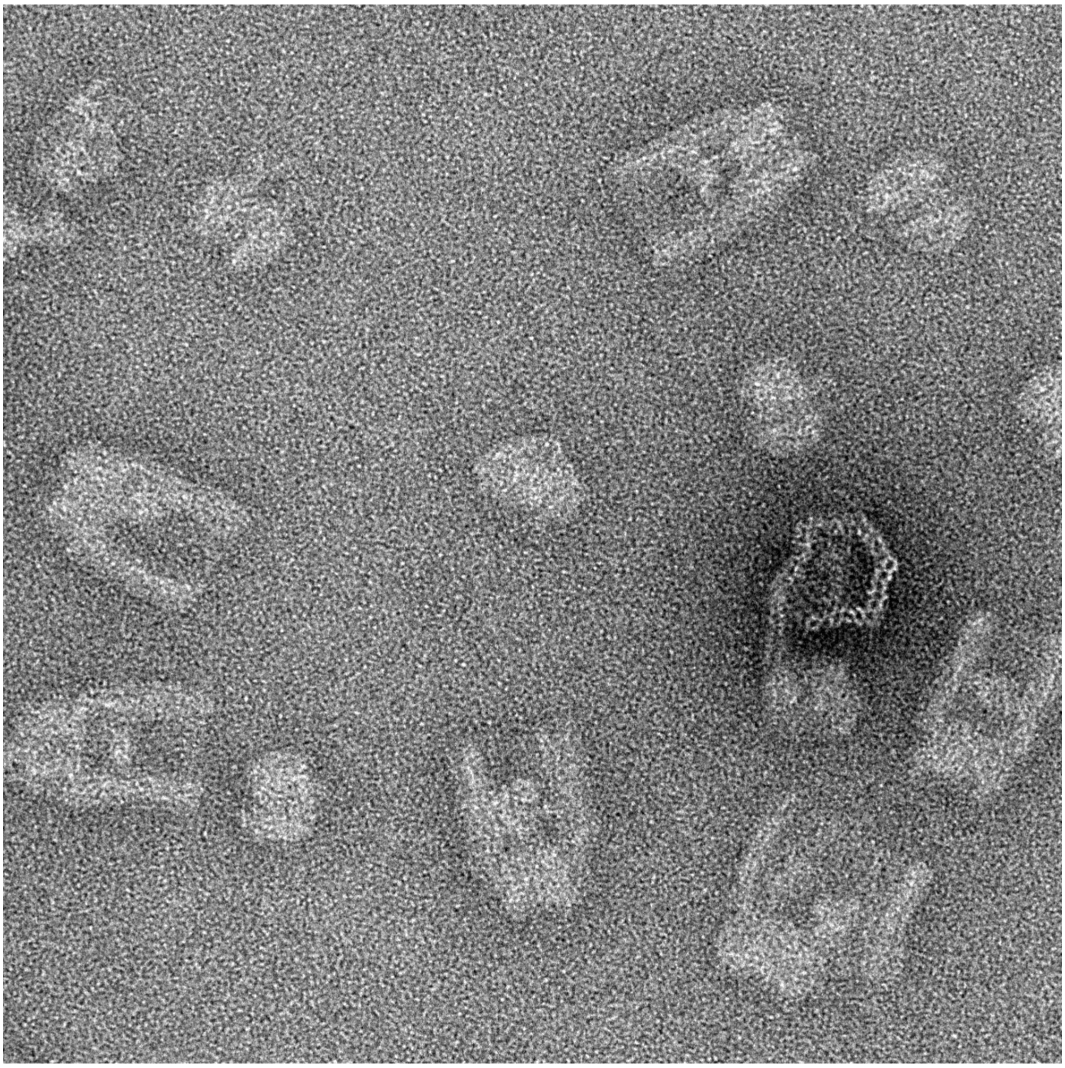

Source Data Fig. 4e: Wide-field TEM images of A^{L1}(p97)

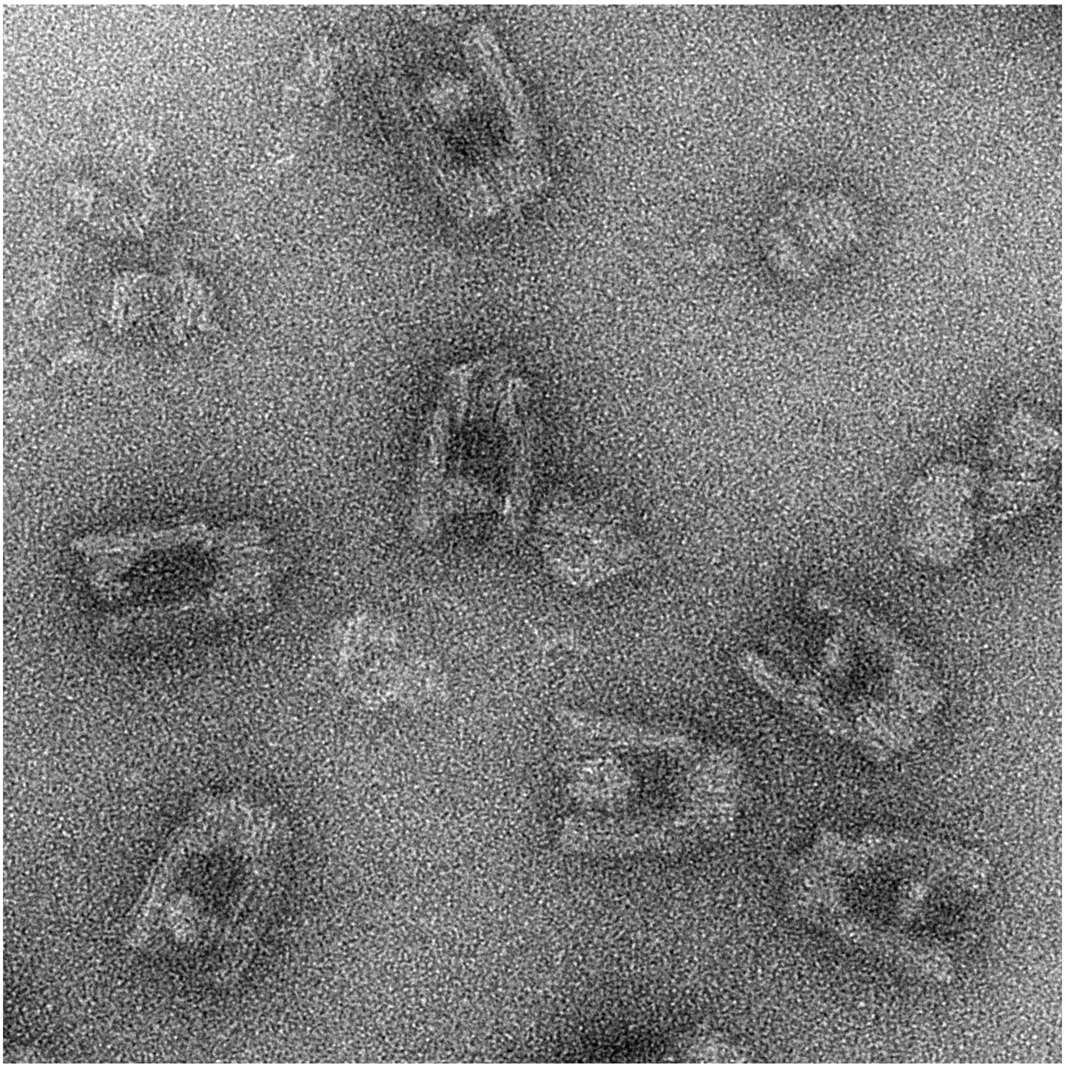

Source Data Fig. 4f: Wide-field TEM images of A^{L41}(p97)

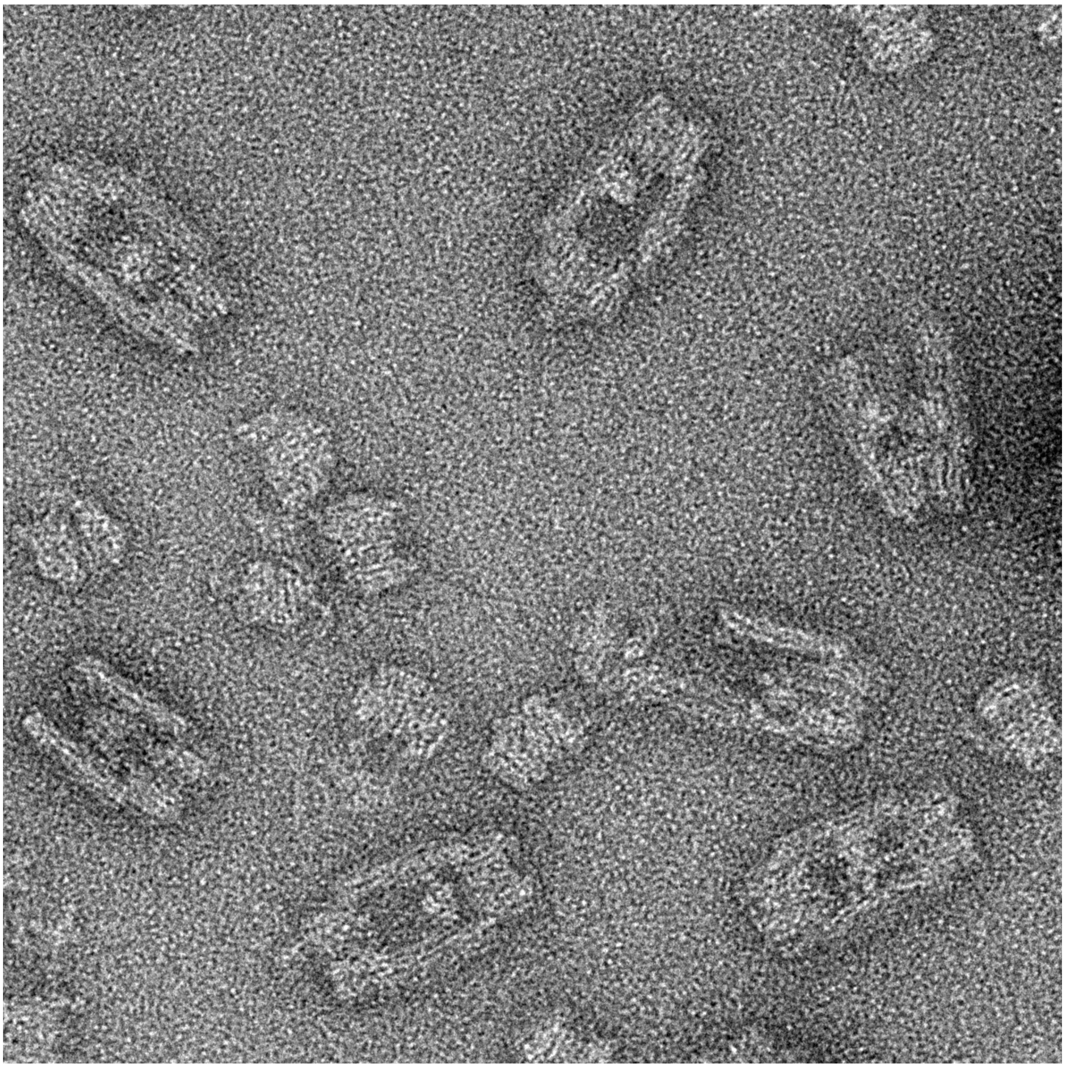

Source Data Fig. 4j: SDS gel B(aCt)

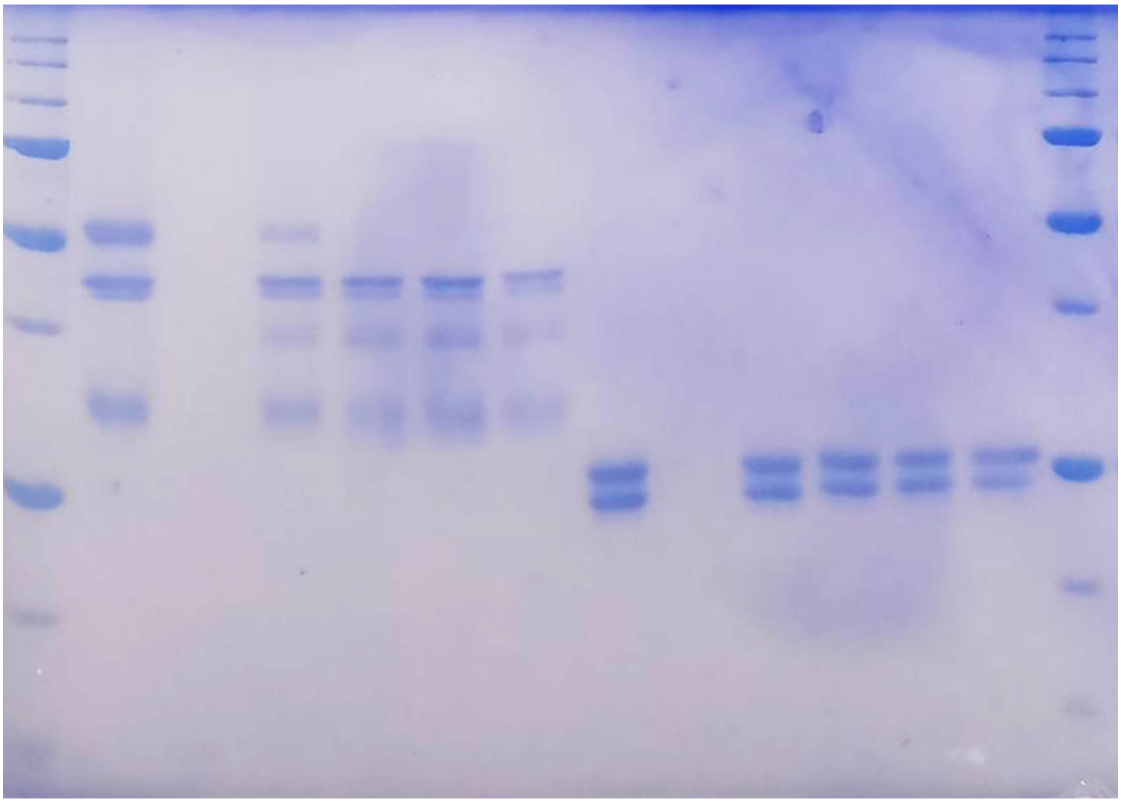

Source Data Fig. 5c: Unprocessed gel A(p97)/B(aCt) chimera

Ethidium Bromide staining

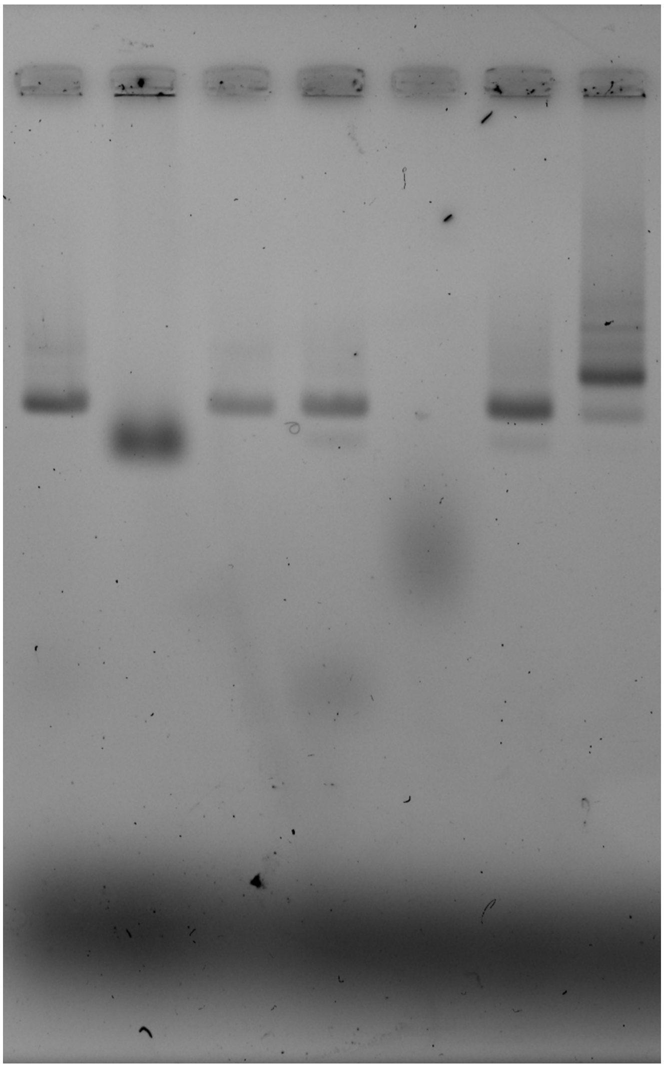

Cy5 illumination

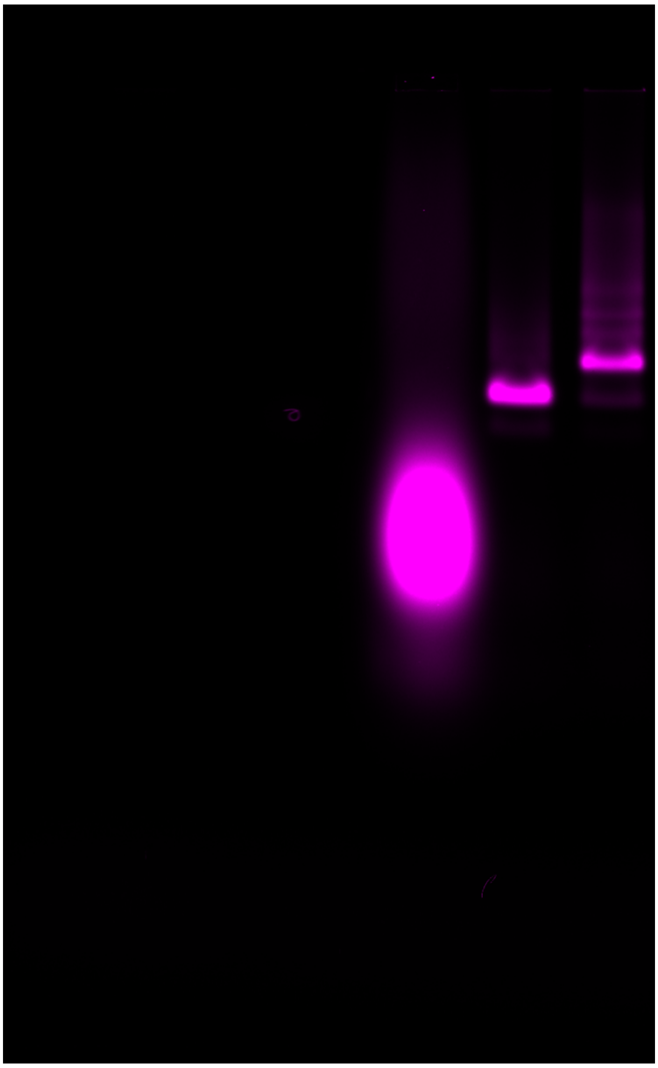

FAM illumination

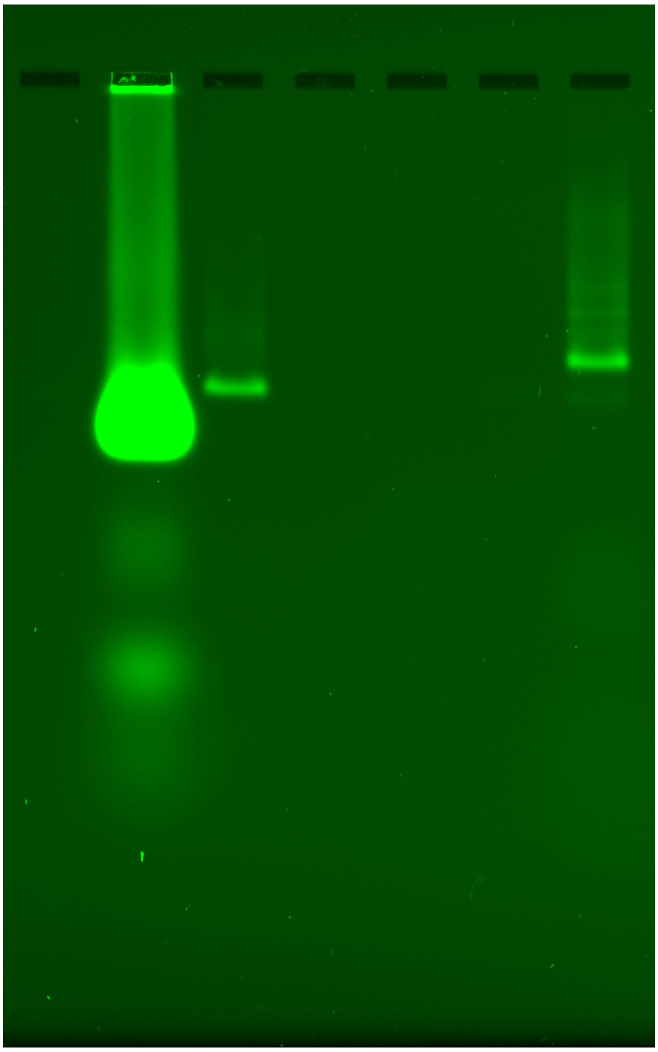

Source Data Fig. 5c: insets TEM

Class averages of AB

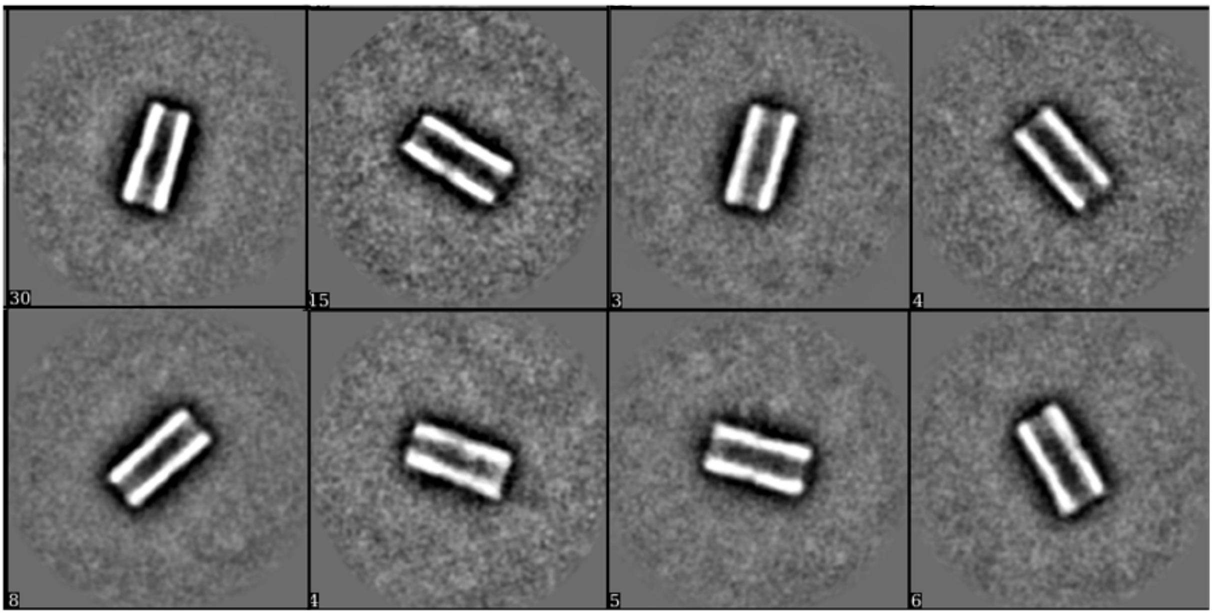

Class averages of A(p97)/B(aCt)

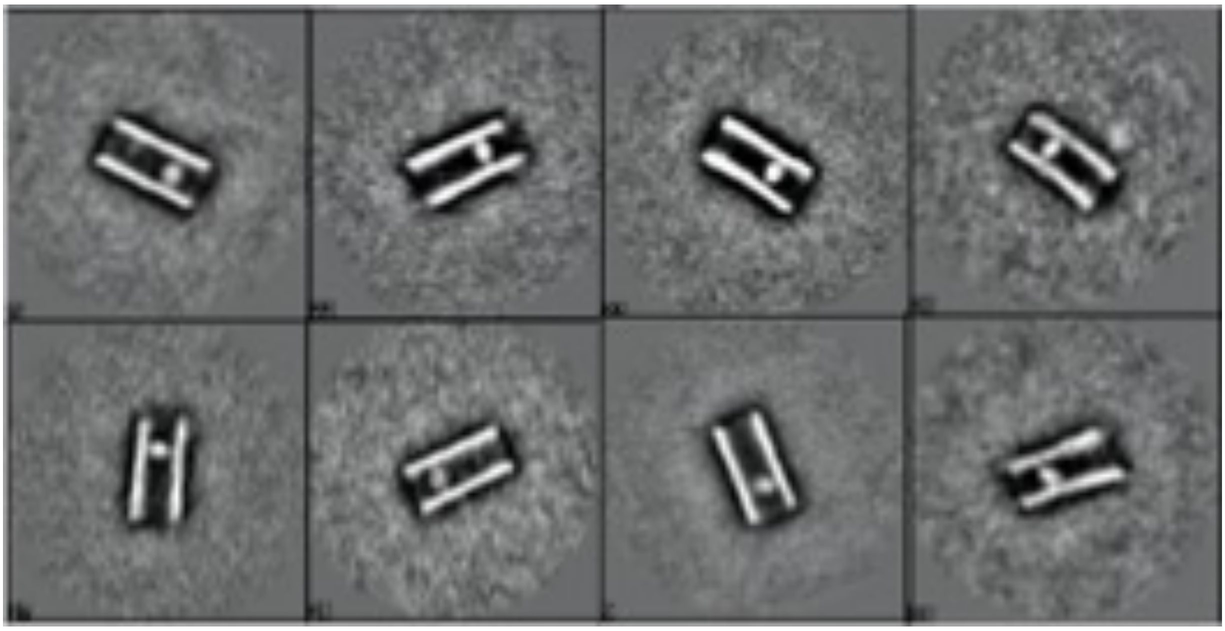

Source Data ED Fig. 3c: Unprocessed SDS gel and blots A(p97)/B(Src)
lanes 2 to 5 were shown in the figure

pTyr antibody

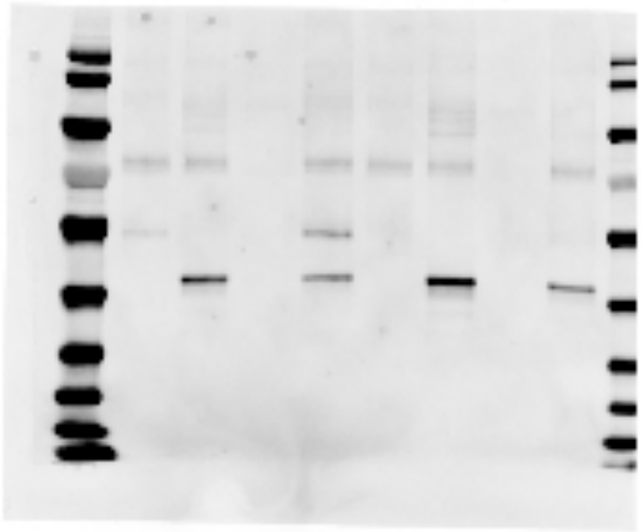

I3 antibody

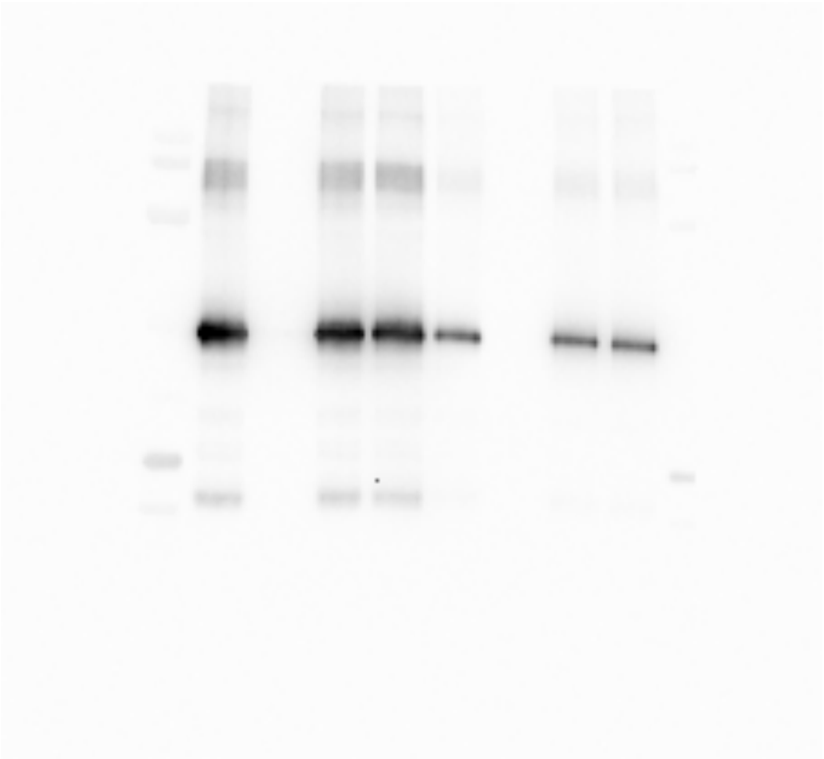

PP1 antibody

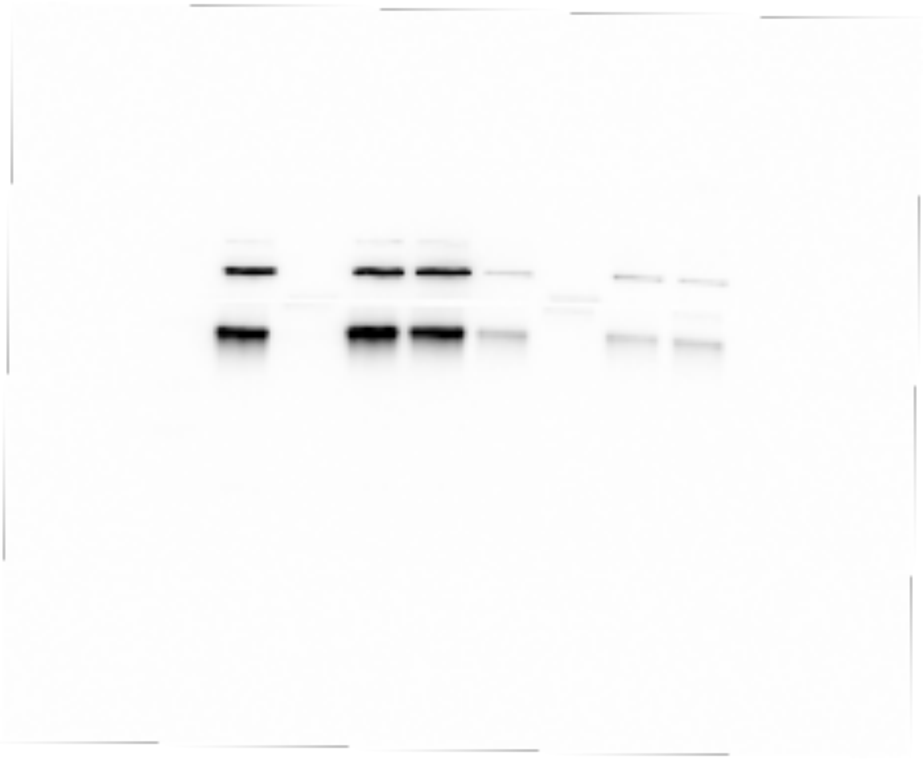

SDS22 antibody

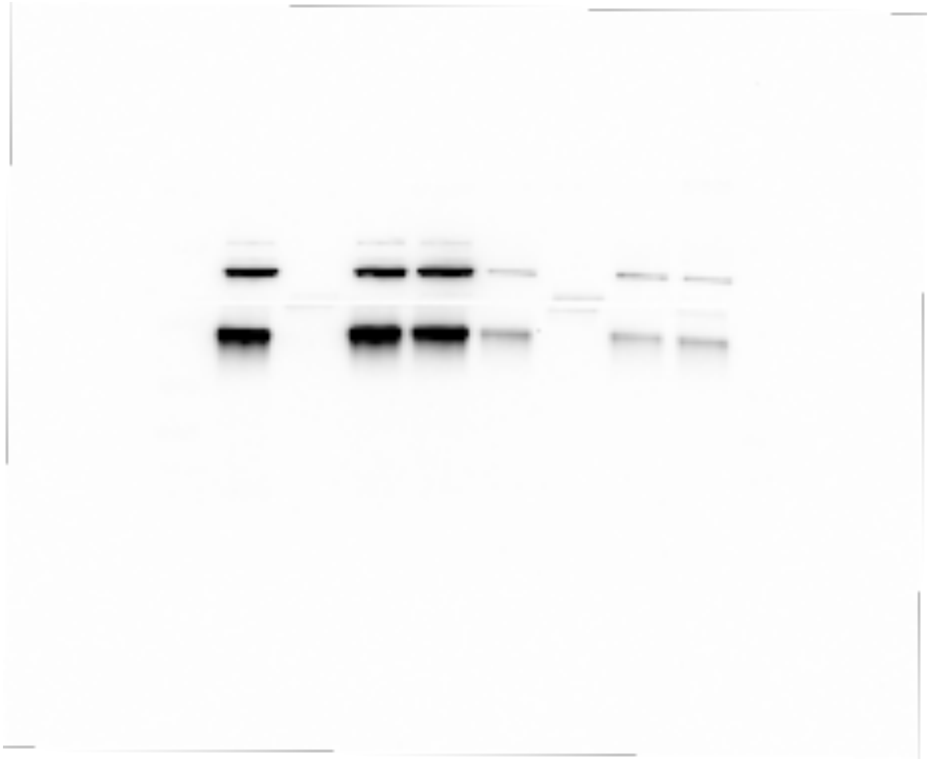

p37 antibody

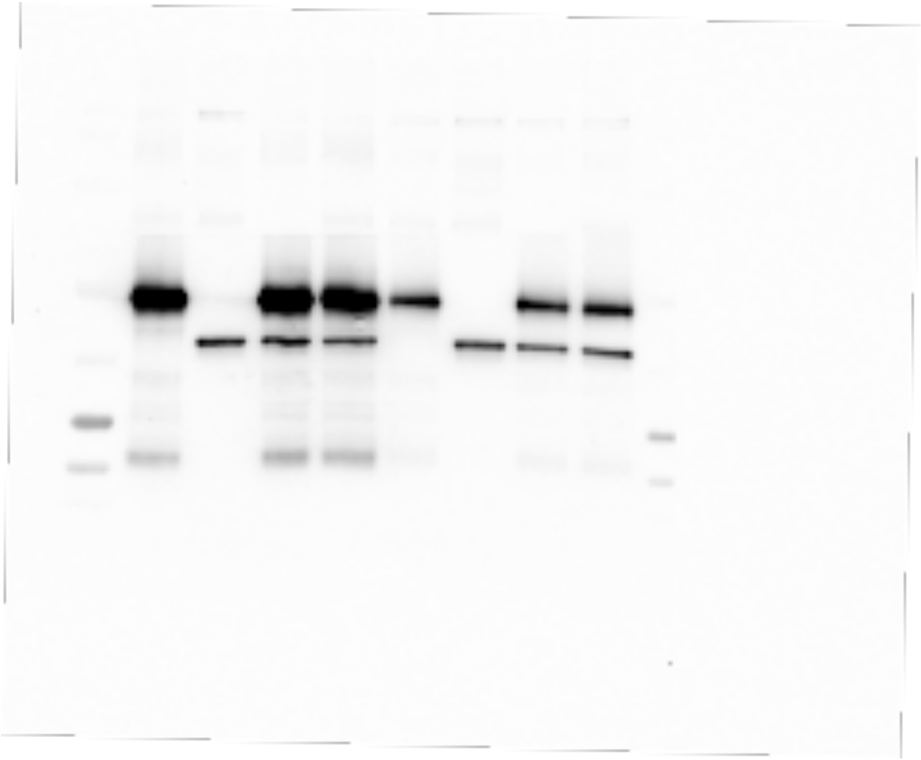

Supplement: Supplementary file 6 — Figure 1b: wide-field TEM images of p97. Figure 2a: TEM-averaged classes of N and E. Figure 2b: wide-field TEM images of NE. Figure 2c: TEM-averaged classes of NE. Figure 2e: TEM images of L. Figure 2g: wide-field TEM images of AL. Figure 2h: wide-field TEM images and class averages of A2L. Figure 2j: wide-field TEM images and class averages of AB. Figure 2k: TEM images of ABC. Figure 3b: unprocessed AGE of A(p97). Figure 3c: wide-field TEM images of A(p97). Figure 3e: slices of the 3D cryo-EM density map. Figure 4a: wide-field TEM images of A. Figure 4b: wide-field TEM images of A(p97). Figure 4c: wide-field TEM images of Arel. Figure 4d: wide-field TEM images of AL4(p97). Figure 4e: wide-field TEM images of AL1(p97). Figure 4f: wide-field TEM images of AL41(p97). Figure 4j: SDS gel B(aCt). Figure 5c: unprocessed gel A(p97)/B(aCt) chimera. Figure 5c (inset) TEM class average of AB. Extended Data Fig. 3c: unprocessed SDS gel and blots A(p97)/B(Src). [file 41565_2024_1738_MOESM6_ESM.pdf]
